# Precise dating of the Middle-to-Upper Paleolithic transition in Murcia (Spain) supports late Neandertal persistence in Iberia

**DOI:** 10.1016/j.heliyon.2017.e00435

**Published:** 2017-11-16

**Authors:** João Zilhão, Daniela Anesin, Thierry Aubry, Ernestina Badal, Dan Cabanes, Martin Kehl, Nicole Klasen, Armando Lucena, Ignacio Martín-Lerma, Susana Martínez, Henrique Matias, Davide Susini, Peter Steier, Eva Maria Wild, Diego E. Angelucci, Valentín Villaverde, Josefina Zapata

**Affiliations:** a Institució Catalana de Recerca i Estudis Avançats (ICREA), Passeig Lluís Companys 23, 08010 Barcelona, Spain; b Universitat de Barcelona, Departament d’Història i Arqueologia, Facultat de Geografia i Història, c/Montalegre 6, 08001 Barcelona, Spain; c UNIARQ – Centro de Arqueologia da Universidade de Lisboa, Faculdade de Letras de Lisboa, Universidade de Lisboa, Alameda da Universidade, 1600-214 Lisboa, Portugal; d Università degli Studi di Trento, Dipartimento di Lettere e Filosofia, via Tommaso Gar 14, 38122 Trento, Italy; e Parque Arqueológico do Vale do Côa, Fundação Côa Parque, Rua do Museu, 5150-610 Vila Nova de Foz Côa, Portugal; f Universitat de València, Departament de Prehistòria, Arqueologia i Història Antiga, Av. Blasco Ibañez 28, 46010 València, Spain, Av. Blasco Ibañez 28, 46010 València, Spain; g Department of Anthropology, Rutgers University, Biological Sciences Building, 32 Bishop Street, New Brunswick, NJ, 08901, USA; h University of Cologne, Institute of Geography, Albertus-Magnus-Platz, 50923 Cologne, Germany; i Universidad de Murcia, Área de Prehistoria, Facultad de Letras, Campus de La Merced, 30071 Murcia, Spain; j Università di Siena, Dipartimento di Scienze fisiche, della Terra e dell'Ambiente, Strada Laterina 8, 53100 Siena, Italy; k VERA (Vienna Environmental Research Accelerator) Laboratory, Faculty of Physics – Isotope Research and Nuclear Physics, University of Vienna, Währingerstraße 17, 1090 Wien, Austria; l Universidad de Murcia, Área de Antropología Física, Facultad de Biología, Campus Universitario de Espinardo, 30100 Murcia, Spain

**Keywords:** Archaeology

## Abstract

The late persistence in Southern Iberia of a Neandertal-associated Middle Paleolithic is supported by the archeological stratigraphy and the radiocarbon and luminescence dating of three newly excavated localities in the Mula basin of Murcia (Spain). At Cueva Antón, Mousterian layer I-k can be no more than 37,100 years-old. At La Boja, the basal Aurignacian can be no less than 36,500 years-old. The regional Middle-to-Upper Paleolithic transition process is thereby bounded to the first half of the 37th millennium Before Present, in agreement with evidence from Andalusia, Gibraltar and Portugal. This chronology represents a lag of minimally 3000 years with the rest of Europe, where that transition and the associated process of Neandertal/modern human admixture took place between 40,000 and 42,000 years ago. The lag implies the presence of an effective barrier to migration and diffusion across the Ebro river depression, which, based on available paleoenvironmental indicators, would at that time have represented a major biogeographical divide. In addition, (a) the Phlegraean Fields caldera explosion, which occurred 39,850 years ago, would have stalled the Neandertal/modern human admixture front because of the population sink it generated in Central and Eastern Europe, and (b) the long period of ameliorated climate that came soon after (Greenland Interstadial 8, during which forests underwent a marked expansion in Iberian regions south of 40°N) would have enhanced the “Ebro Frontier” effect. These findings have two broader paleoanthropological implications: firstly, that, below the Ebro, the archeological record made prior to 37,000 years ago must be attributed, in all its aspects and components, to the Neandertals (or their ancestors); secondly, that modern human emergence is best seen as an uneven, punctuated process during which long-lasting barriers to gene flow and cultural diffusion could have existed across rather short distances, with attendant consequences for ancient genetics and models of human population history.

## 1 Introduction

In the Aquitaine basin and the Pyrenees, the Middle Paleolithic (MP) Mousterian culture is followed, in succession, by the Châtelperronian, the Protoaurignacian and the Aurignacian I (a.k.a. Early Aurignacian). In Iberia, these initial phases of the Upper Paleolithic (UP) are represented in the Cantabrian strip and in Catalonia but remain unknown to the South of the Ebro basin. Based on these observations, the “Ebro Frontier” model hypothesizes that (a) in Valencia, Murcia, Andalusia, Gibraltar, the Mesetan hinterland, and Portugal, the corresponding chronostratigraphic slot is occupied by a late-persisting Mousterian and (b) the pattern is explained by the major biogeographical divide that the Ebro basin would have been at that time (Zilhão, 1993; Zilhão, 2000; Zilhão, 2006a; Zilhão, 2009).

The paleontological and ancient DNA (aDNA) evidence indicates that, in Europe, extensive admixture occurred at the time of contact between aboriginal Neandertals and in-dispersing groups of modern humans, resulting in the former’s eventual assimilation (Smith et al., 2005; Trinkaus, 2007; Pääbo, 2015). The authorship of the Châtelperronian, the Protoaurignacian, and the other so-called “transitional” industries from this time remains debated (Higham et al., 2010; Caron et al., 2011; Hublin et al., 2012; Trinkaus and Zilhão, 2013; Zilhão, 2013; Zilhão et al., 2015; Welker et al., 2016). In Western Eurasia, however, the Mousterian is exclusively associated with the Neandertals, while the Aurignacian I and the succeeding Aurignacian II (a.k.a. Evolved Aurignacian), which extend from Asturias in the West to northern Israel in the East, are associated with modern humans only (Verna et al., 2012). In this context, the broader paleoanthropological significance of the “Ebro Frontier” model resides in the implication that Neandertals persisted in Southern and Western Iberia longer than everywhere else.

Within the model, the chronological boundaries of the Middle Paleolithic/Neandertal persistence pattern are given by the difference in age between the earliest archeological cultures (or their phases) that, on each side of the Ebro divide, are unambiguously associated with modern humans: to the North, the Aurignacian I; to the South, the Aurignacian II. Given the currently accepted dating of these assemblage types (Higham et al., 2011; Banks et al., 2013a; Banks et al., 2013b), the lag implicated (i.e., the duration of the “Ebro Frontier” pattern) is, at the least, of three millennia, between 40,000 and 37,000 years ago.

The number of occurrences substantiating that Iberian regions to the South of the Ebro divide were occupied by a late-persisting Mousterian while those to the North were occupied by the Aurignacian I is, however, limited. This paucity of occurrences has led to alternative readings of the evidence whereby the late persistence is apparent. In such readings, the “Ebro Frontier” pattern would stem from insufficient information on the early Upper Paleolithic, aggravated by (a) Middle Paleolithic-associated radiocarbon dating results that would be inaccurately young, and (b) ambiguity in the definition of the stone tool assemblages implicated (Wood et al., 2013).

Conversely, it has been argued that no Aurignacian exists in Southern and Western Iberia, their Upper Paleolithic beginning with the Gravettian (de la Peña, 2013). Such views imply that (a) the Mousterian persisted even longer (Finlayson et al., 2006; Finlayson et al., 2008), or (b) after a Neandertal extinction event, Southern and Western Iberia remained uninhabited until modern human reoccupation (Bradtmöller et al., 2012; Galván et al., 2014). In these scenarios, the role of biogeographical divide played by the Ebro basin under certain climatic and environmental conditions would not have contributed to observed patterns in any significant manner.

Re-dating and critical examination of old sites and collections (Kehl et al., 2013; Wood et al., 2013) have advanced these debates. The scope of the many empirical issues involved, however, requires the excavation of new sites with the potential to settle the key points of contention. Here, we report on the progress made in that direction resulting from a decade of fieldwork in Murcia, Southeast Spain.

When specifically cited, individual radiocarbon results are given as provided by the dating laboratory, i.e., expressed in uncalibrated radiocarbon years Before Present (BP). Throughout, however, the discussion is framed in calendar terms, i.e., in years or thousands of years (ka) before the time of measurement for U-series and luminescence dates, and in calibrated years or thousands of years BP for radiocarbon dates.

## 2 Results

### 2.1 Site formation and dating

**Fig. 1:**
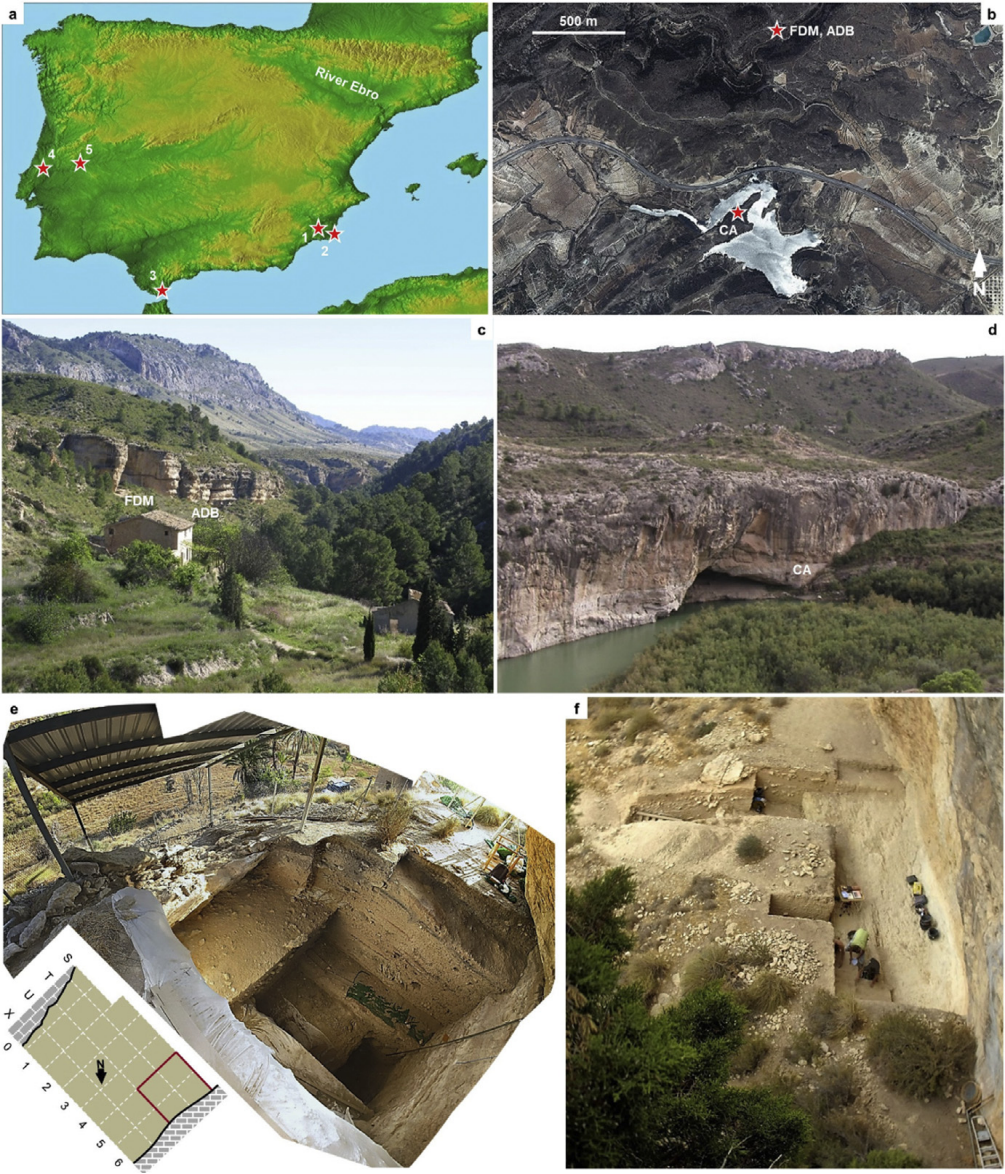
The Mula basin sites. a. Location of the late Middle Paleolithic sites of Southern and Western Iberia relative to the Ebro basin (1. Cueva Antón; 2. Sima de las Palomas; 3. Gorham’s Cave; 4. Gruta da Oliveira; 5. Foz do Enxarrique). b. Location of the Mula basin sites in a 2013 orthophoto.

Source: http://cartomur.imida.es/visorcartoteca/; CA, Cueva Antón; FDM, Finca Doña Martina; ADB, Abrigo de La Boja); as the crow flies, the distance between Cueva Antón and the Rambla Perea rock-shelters is 1670 m. c. The Rambla Perea rock-shelters from upstream (2009). d. The tail of the La Cierva reservoir, with Cueva Antón seen from Northeast (2007), after (Zilhão et al., 2016), with permission from Elsevier. e. La Boja at the end of the 2016 field season; the red lines in the excavation grid denote the reference cross-sections in Fig. 4. f. Finca Doña Martina’s excavation trench at the end of the 2016 field season.

We excavated three localities <2 km apart within the Mula basin (Angelucci et al., 2017). The Supplementary Information (SI) Appendix provides a succinct geographical description of the area, as well as extensive monographic presentations of the sites’ stratigraphic sequences, dating, human occupation features, and stone tool assemblages. The sites are: Cueva Antón (CA; 38°03′51.84″N, 01°29′47.20″W), Finca Doña Martina (FDM; 38°04′43.21″N, 01°29′25.13″W), and Abrigo de La Boja (ADB; 38°04′43.37″N, 1°29′23.17″W) (Fig. 1; Figs. S1.1–S1.2).

**Fig. 2:**
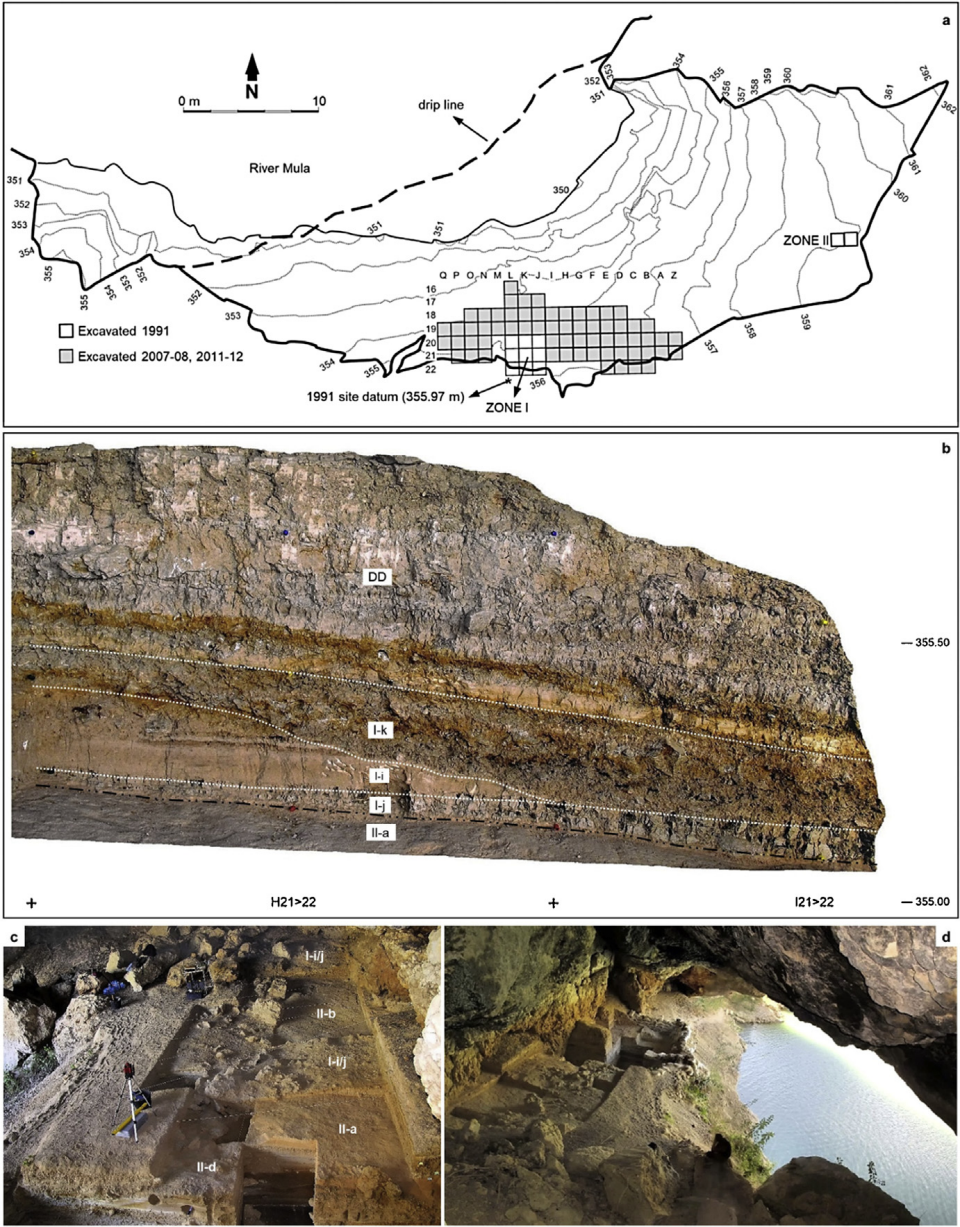
Cueva Antón. a. Site plan and excavation grid. b. Cross-section illustrating the position of layer I-k — sandwiched between the DD reservoir-inundation silts and the basal alluvium of sub-complex AS1 (here represented by layers I-i, I-j and II-a). c. View from the West at the end of the 2011 field season; the layer labels designate the units whose surface is exposed in each sector. d. View from the East at the end of the 2012 field season. Elevations are in m asl. Figs. 2a, 2c and 2d after (Zilhão et al., 2016), with permission from Elsevier.

Cueva Antón (SI appendix, chapter 2; Fig. 2) is a cave located in the valley of River Mula (Zilhão et al., 2010a; Angelucci et al., 2013; Zilhão et al., 2016). Sandwiched between basal palustrine deposits (complex FP) and well-bedded inundation silts and sands accumulated in recent times during periods of submersion by the reservoir of the La Cierva dam (complex DD), the site contains a thick Upper Pleistocene succession (complex AS). The base of this succession (sub-complexes AS2-AS5) is an alluvial fill of MIS (Marine Isotope Stage) 5 age that features discrete anthropogenic lenses recording short-lived occupation episodes — the last of which is layer II-l. After an erosional hiatus, broadly coincident with MIS 4, the accumulation of alluvium inside the cave — represented by the basal layers (I-i, I-j, II-a, II-c and II-b; Fig. 2) of the AS1 sub-complex — resumed briefly in MIS 3. Layer I-k, an archeologically fertile breccia made-up of wall degradation debris, caps the AS1 deposit, whose surface is erosional. Previous work has placed the basal MIS 5 alluvium in the 72–85 ka age range (Burow et al., 2015; Zilhão et al., 2016) and the MIS 3 alluvium and breccia in the 35.1–37.7 ka age range (Table 1; Zilhão et al., 2016). Here, the focus lies on layer I-k’s site formation process and stone tool assemblage composition, upon which lie its assignment to the Middle Paleolithic.

**Table 1:** Cueva Antón. ABOx-SC radiocarbon dating results for sub-complex AS1 (after Zilhão et al., 2016). The ages have been calibrated against IntCal13 (Reimer et al., 2013) in Calib 7.0.4 (Stuiver and Reimer, 1993); the calibrated ages are given as 95.4% probability intervals.

Sample	Taxon	Field unit	Layer	OxA	δ^13^C [‰]	Yield (mg)	% Yld	% C	Age BP	Age cal BP
I20-3	Conifer	I-k	I-k top	26346	−22.3	4.7	4.1	66.9	31790 ± 270	35067–36245
G21-4	*Juniperus* sp.	dec 4	I-k base	22625	−21.0	8.6^a^	8.7^a^	77.9	32330 ± 250	35627–36826
E21-11	*Juniperus* sp.	dec 5a	II-a	22019	−22.7	6.43	6.0	75.6	32390 ± 280	35594–37055
J19-7	*Pinus* sp.	I-k/II-d	II-b	21244	−22.3	11.7^a^	12.1^a^	88.4	32890 ± 200	36314–37714

a These values are estimated as only approximately half of the sample remaining after the wet chemistry was pre-combusted.

**Fig. 3:**
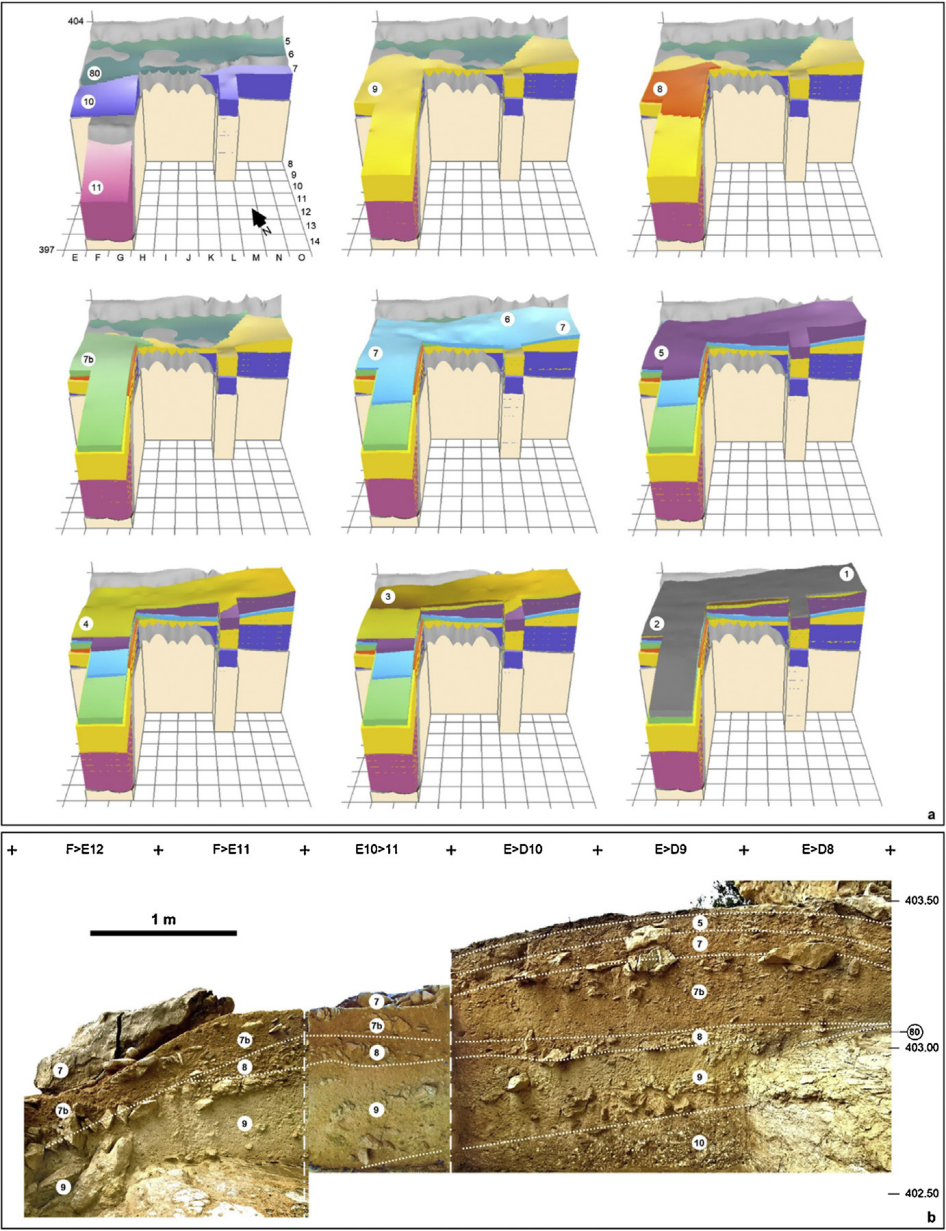
Finca Doña Martina. a. 3D model of the accumulation (for an extended discussion, see the SI appendix); the labels denote the different stratigraphic units recognized. b. The stratigraphic succession in the trench’s western wall. Elevations are in m asl.

**Fig. 4:**
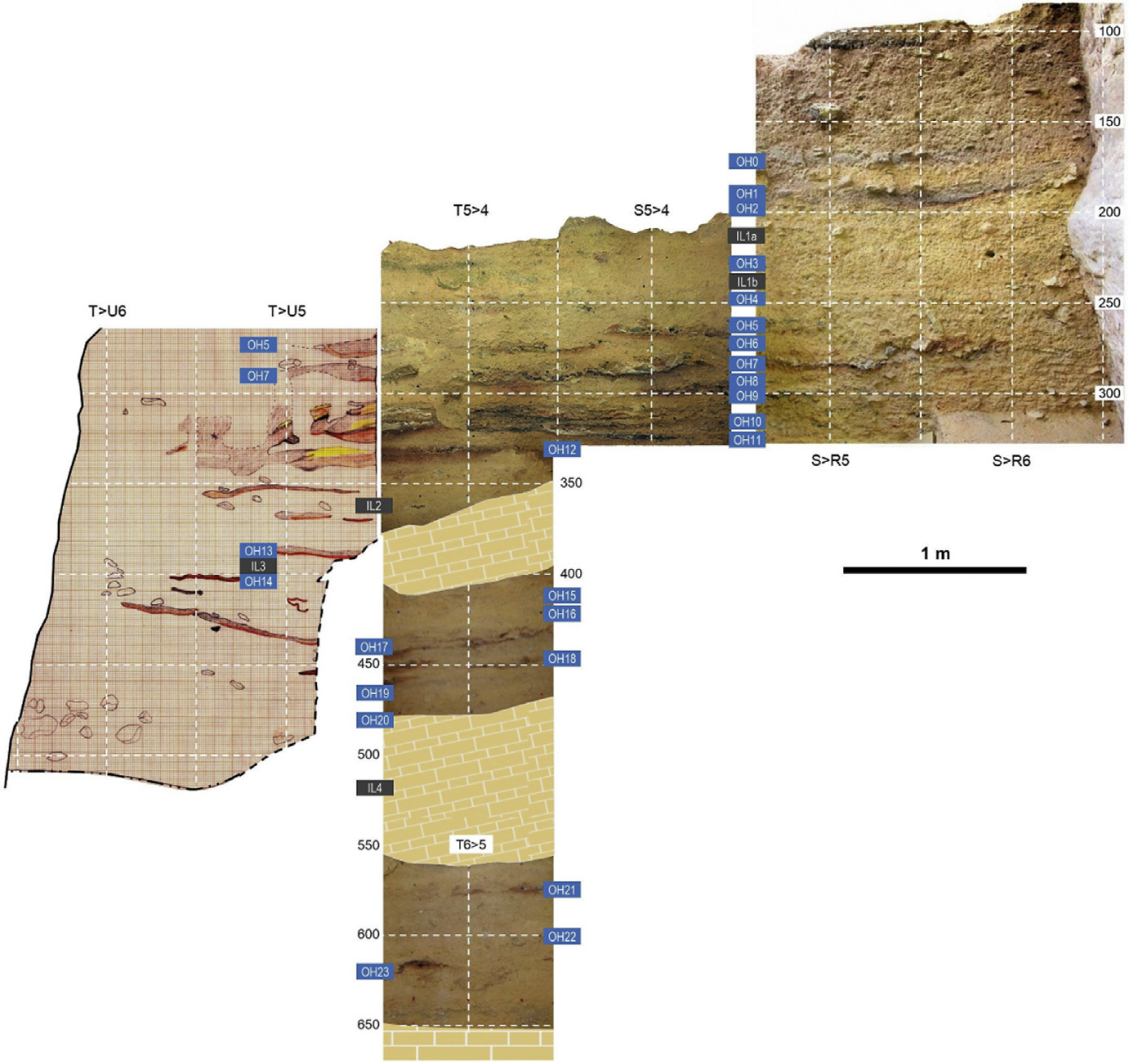
La Boja. The archeo-stratigraphic sequence. Trench cross-sections as recorded at the end of the 2013 field season (for an extended discussion, see the SI appendix). Elevations are in cm below datum.

**Fig. 5:**
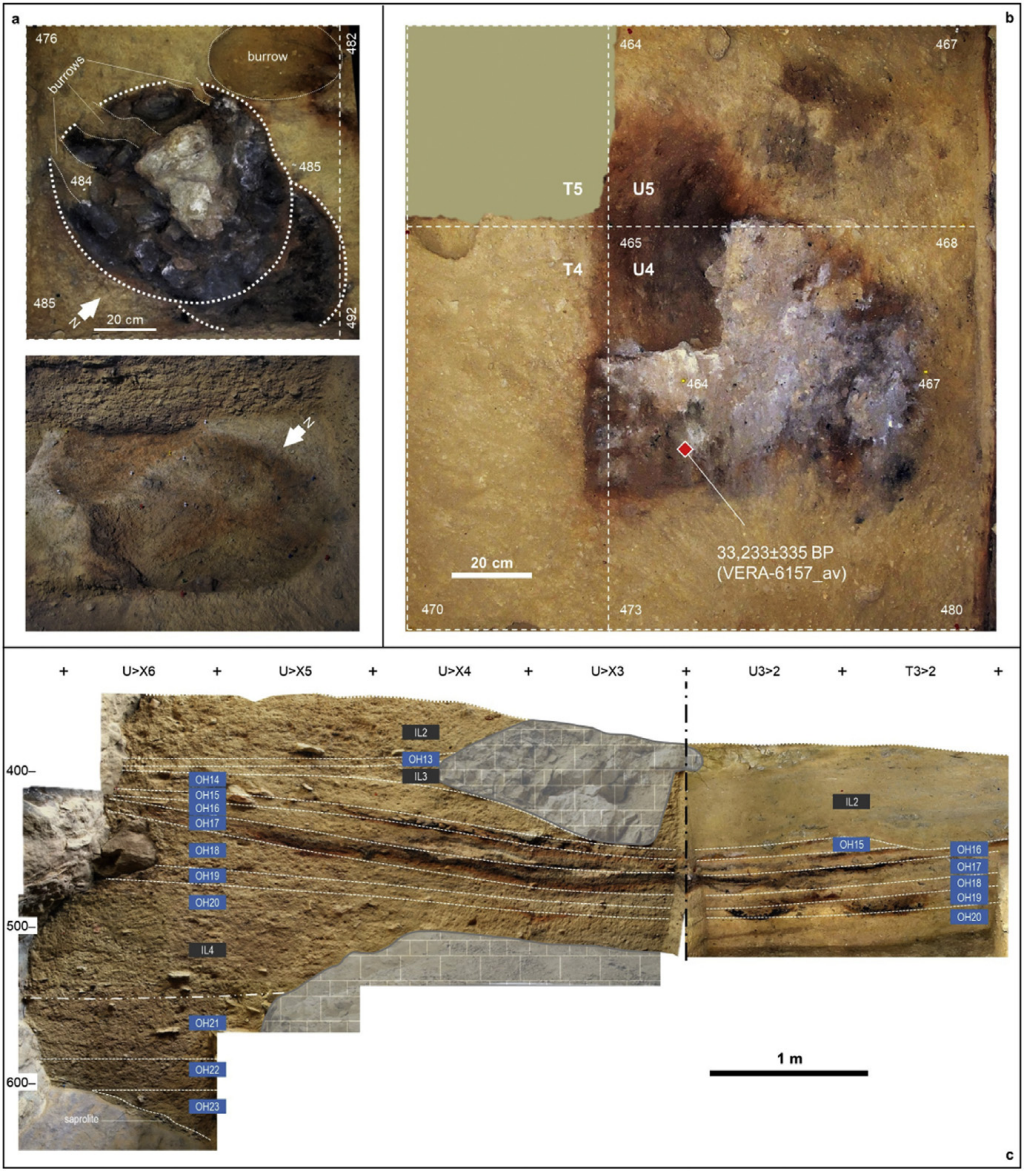
The basal, Mousterian and Aurignacian sections of the La Boja sequence. Elevations are in cm below datum. a. The OH19 double hearth in grid unit T3 at exposure of the feature’s top (above, orthorectified plan view) and base (below, oblique view from the opposite angle). b. Orthorectified plan view of the OH19 hearth in grid unit U4; the provenience of the sample that established this horizon’s radiocarbon age is indicated by the red diamond. c. Stratigraphic cross-sections representing the basal parts of the sequence extant at the end of the 2014 field season; the preservation of intact hearths and/or extensive lenses of anthropized sediment allows sub-centimeter discrimination of occupation floors (OH) separated by intermediate levels (IL); the latter are sterile or only contain post-depositionally intruded items (OH21-23 are Mousterian, OH15-20 are Aurignacian, OH13-OH14 are Early Gravettian).

Finca Doña Martina (SI appendix, chapter 3; Fig. 3) and La Boja (SI appendix, chapter 4; Fig. 4, Fig. 5) are rock-shelters located in the Rambla Perea (Zilhão et al., 2010b; Lucena et al., 2012). In the regional landscape, this tributary of River Mula likewise communicates the lowlands of the Murcia littoral with the plateaus and mountain ranges extending northward to the Mesetan hinterland. Both sites feature stratigraphic successions where a basal Middle Paleolithic is overlain by long Upper Paleolithic sequences. The preservation is good for shell but poor-to-nil for bone, and charcoal is abundant — even though, at Finca Doña Martina, chemically weathered (leading to radiocarbon results that are minimum ages only; Tables S3.1-S3.2).

Layer 8 of Finca Doña Martina yielded a lithic assemblage whose Aurignacian affinities (Figs. S3.31-S3.32) are consistent with the layer’s stratigraphic position between Mousterian layer 9 and Gravettian layers 7b and 6/7. At La Boja, the excellent preservation of charcoal and the sub-centimeter precision with which most archeo-stratigraphic units — designated OH (Occupation Horizons) — could be separated provided for a large series of radiocarbon results that, a burrow sample excepted, are in full stratigraphic order (Table 2; Table S4.1; Fig. S4.8). The basal Mousterian dates beyond 44 ka and is buried under a thick, multi-ton, roof-collapsed slab. The site was re-occupied, in the Aurignacian, once this slab was covered by the accumulation of the sediment forming the IL (Intermediate Level) 4 unit. Otherwise archeologically sterile, IL4 includes some post-depositionally intruded material and yielded a date of ca.41 ka. This date provides a *terminus post quem* for the ca.75 cm-thick Aurignacian sequence, which is sealed by another large, roof-collapsed slab. Radiocarbon dating places the three basal Aurignacian horizons (OH18-OH20) within the 34.9–38.2 ka interval and the three upper ones (OH15-OH17) within the 33.9–35.6 ka interval.

**Table 2:** La Boja. Radiocarbon dating results. Calibration used Calib 7.0.4 against IntCal13 (Stuiver and Reimer, 1993, Reimer et al., 2013). Unless otherwise stated, samples were ABA-pretreated. The VERA lab δ^13^C values were determined for the graphitized samples with the AMS system. See Table S4.1 for additional detail.

Horizon	Sample	Lab #	Age BP	Age cal BP (2σ)	δ^13^C [‰]	Observations
burrow	2008-775	OxA-20116	6959 ± 33	7694–7918	−23.72	*Olea europaea*
OH1	2010-27	VERA-5363	12605 ± 45	–	−21.2 ± 1.1	*Juniperus* sp.
		VERA-5363_2	12585 ± 40	–	−20.5 ± 1.1	repeat
		VERA-5363_av	12594 ± 30	14745–15136	–	average
OH1/OH2	2008-774	VERA-5212a	12965 ± 40	15295–15706	−21.4 ± 0.7	*Pinus nigra*
OH3	2013-868	VERA-5937	13290 ± 40	15793–16156	−24.9 ± 1.5	*Pinus nigra/sylvestris*
OH4	2014-846	VERA-6080	15390 ± 50	–	−20.3 ± 1.5	*Juniperus* sp.
		VERA-6080ABOx	15320 ± 45	–	−19.3 ± 1.2	ABOx, no stepped combustion
		VERA-6080_av	15351 ± 33	18522–18740	–	average
OH5	2012-385	VERA-5788	16580 ± 70	19755–20228	−20.5 ± 0.9	*Juniperus* sp.
OH6	2010-183	VERA-5364a	16990 ± 70	20255–20704	−19.5 ± 0.5	*Juniperus* sp.
		VERA-5364b	17430 ± 70	20801–21310	−15.1 ± 0.7	*Juniperus* sp.
OH7	2010-225	VERA-5365	19390 ± 100	–	−20.9 ± 0.6	*Juniperus* sp.
		VERA-5365_2	19240 ± 90	–	−19.0 ± 0.9	repeat
		VERA-5365_av	19307 ± 67	22996–23509	–	average
OH9	2014-1270	VERA-6081	20440 ± 90	–	−19.2 ± 1.6	*Juniperus* sp.
		VERA-6081ABOx	20350 ± 90	–	−21.8 ± 1.0	ABOx, no stepped combustion
		VERA-6081_av	20395 ± 64	24252–24840	–	average
	2012-1522	VERA-5850	20580 ± 100	24434–25155	−22.0 ± 0.9	*Juniperus* sp.
OH10	2010-316	VERA-5366	20980 ± 120	25031–25617	−21.5 ± 0.6	*Juniperus* sp.
		VERA-5366_2	20830 ± 110	–	−22.0 ± 0.5	repeat
		VERA-5366_av	20898 ± 81	–	–	average
		VERA-5366HS	20640 ± 110	–	−20.9 ± 0.6	humic acids
OH11	2008-760	VERA-5213	20980 ± 110	24976–25511	−25.4 ± 0.9	*Juniperus* sp.
		VERA-5213HS	21060 ± 110	–	−22.7 ± 0.5	humic acids
	2014-2578	VERA-6152	20754 ± 105	24577–25343	−20.9 ± 0.9	*Juniperus* sp.
		VERA-6152HS	20457 ± 105	–	−21.3 ± 1.1	humic acids
burrow	2012-178	VERA-5851	20610 ± 110	–	−23.7 ± 1.0	*Juniperus* sp.
		VERA-5851_2	20720 ± 100	–	−19.5 ± 3.7	repeat
		VERA-5851_av	20670 ± 74	24551–25215	–	average
OH12	2012-175	VERA-5852	23530 ± 150	27434–27899	−23.7 ± 1.0	*Juniperus* sp.
		VERA-5852HS	21870 ± 130	–	−19.6 ± 1.2	humic acids
OH13	2012-622	VERA-5789	27260 ± 230	30895–31483	−21.9 ± 0.8	*Juniperus* sp.
		VERA-5789HS	26760 ± 230	–	−21.8 ± 0.7	humic acids
OH15	2014-2903	VERA-6153	30548/+363/−347	33891–35137	−20.3 ± 1.8	*Juniperus* sp.
OH16	2014-3046	VERA-6154	30686/+355/−340	33989–35289	−22.9 ± 1.4	*Juniperus* sp.
OH17	2012-1518	VERA-5853HS	29300/+300/−290	–	−21.0 ± 1.4	humic acids
	2014-3129	VERA-6155HS	29230/+298/−287	–	−17.7 ± 1.7	humic acids
	2014-3184	VERA-6156	30918/+359/−343	34165–35561	−26.8 ± 1.6	*Juniperus* sp.
OH18	2012-1352	VERA-5854	32080/+420/−400	34948–37011	−20.9 ± 1.0	*Juniperus* sp.
		VERA-5854HS	30090/+320/−310	–	−23.2 ± 1.2	humic acids
OH19	2014-3348	VERA-6157	33290/+494/−466	–	−22.4 ± 1.6	*Juniperus* sp.
		VERA-6157ABOxSC	33179/+482/−455	–	−23.2 ± 1.4	ABOx, stepped combustion
		VERA-6157_av	33233 ± 335	36491–38396	–	average
	2014-3421	VERA-6158HS	32331/+439/−417	–	−26.1 ± 1.9	*Juniperus* sp.
OH20	2012-1382	VERA-5855	32890/+430/−410	–	−22.6 ± 1.4	*Juniperus* sp.
		VERA-5855ABOxSC	33170/+470/−450	–	−24.4 ± 2.2	ABOx, stepped combustion
		VERA-5855_av	33017 ± 310	36321–38191	–	average
		VERA-5855HS	31490/+370/−350	–	−23.5 ± 1.2	humic acids
IL4	2012-1481	VERA-5856	37160/+680/−620	–	−25.9 ± 1.4	*Juniperus* sp.
		VERA-5856ABOxSC	37154/+710/−660	–	−19.6 ± 1.5	ABOx, stepped combustion
		VERA-5856_av	37157 ± 472	40794–42356	–	average
		VERA-5856HS	31960/+670/−620	–	−22.2 ± 1.2	humic acids
OH22	2013-384	VERA-5899	46500/+2400/−1800	beyond curve	−24.1 ± 4.8	*Pinus nigra/sylvestris*
		VERA-5899HS	40820/+1090/−960	–	−24.5 ± 1.3	humic acids
	2013-330	VERA-5900	46900/+2400/−1800	beyond curve	−21.1 ± 2.9	*Pinus nigra/sylvestris*
		VERA-5900HS	45700/+2100/−1700	–	−26.9 ± 1.8	humic acids
OH23	2013-258	VERA-5901	43300/+1600/−1300	44181–49611	−23.3 ± 1.5	*Juniperus* sp.
		VERA-5901HS	46200/+2200/−1700	–	−19.7 ± 1.2	humic acids
	2013-361	VERA-5902HS	42800/+1400/−1200	–	−21.4 ± 3.1	*Pinus nigra/sylvestris;* humic acids

**Fig. 6:**
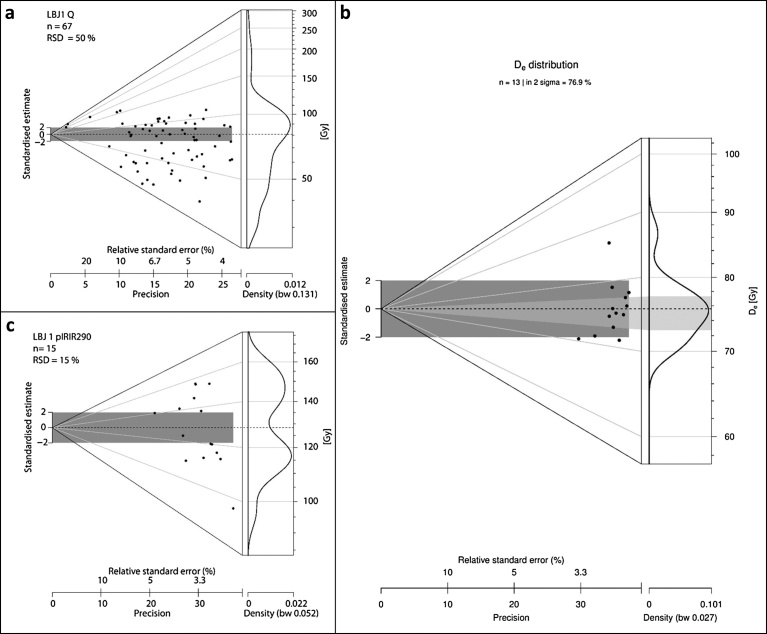
La Boja OSL dating. Representative equivalent dose distributions of the dated quartz and feldspar samples. The distributions, displayed as abanico plots (Dietze et al., 2016), which combine a scatter plot with a kernel density estimate, are for sample C-L3901, taken at the base of the sequence, immediately below OH23. The dashed line is the arithmetic mean equivalent dose. The plots were generated using R Luminescence package version 0.7.3 (Dietze and Kreutzer, 2017). a. quartz. b. feldspar (IR_50_). c. feldspar (pIRIR_290_).

**Fig. 7:**
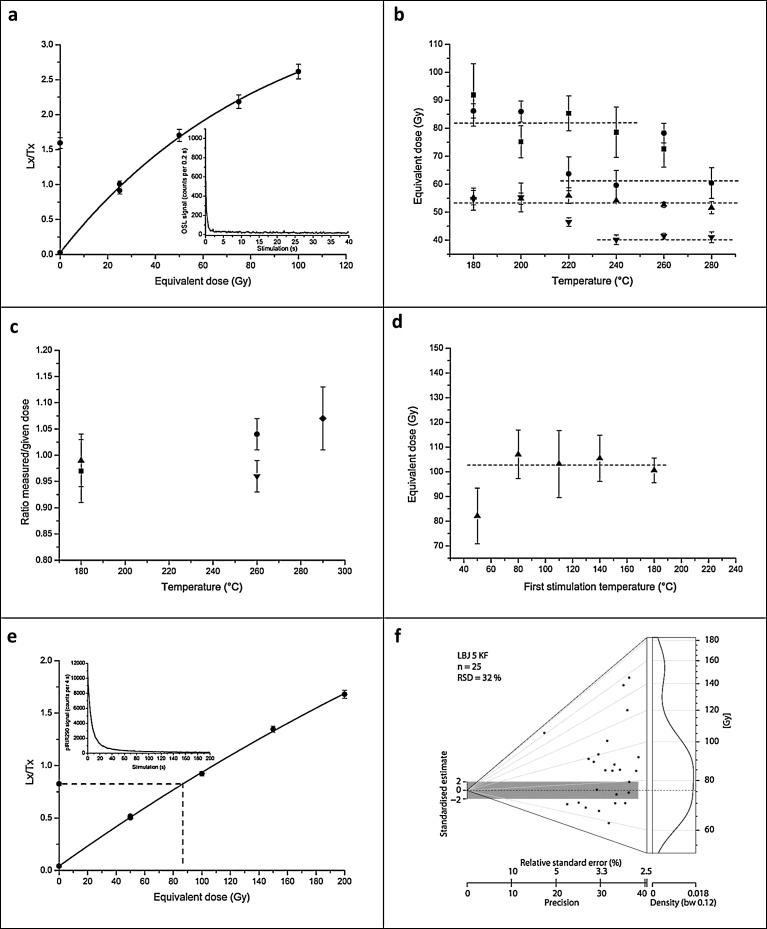
La Boja OSL dating. Analytical data. a. Representative quartz dose response and decay curve for sample C-L3905. b. Preheat plateau tests indicating that the equivalent dose is independent from temperature treatment between: 180 and 240 °C (C-L3901, square); 220 and 280 °C (C-L3904, circle); 180 and 280 °C (C-L3905, triangle); 240 and 280 °C (C-L3906, inverted triangle). c. Dose recovery tests showing that a laboratory given dose was best recovered using a temperature of 180 °C for samples C-L3901 and C-L3905 and of 260 °C for samples C-L3904 and C-L3906. d. Prior IR stimulation temperature tests carried out for feldspar sample C-L3905 indicating a plateau between 80 and 180 °C; 80 °C was chosen as prior-IR stimulation temperature. e. Representative feldspar pIRIR290 dose response and decay curves of sample C-L3905. f. Dose distribution of feldspar sample C-L3905 displayed as abanico plot; the dashed line is the MAM equivalent dose.

**Fig. 8:**
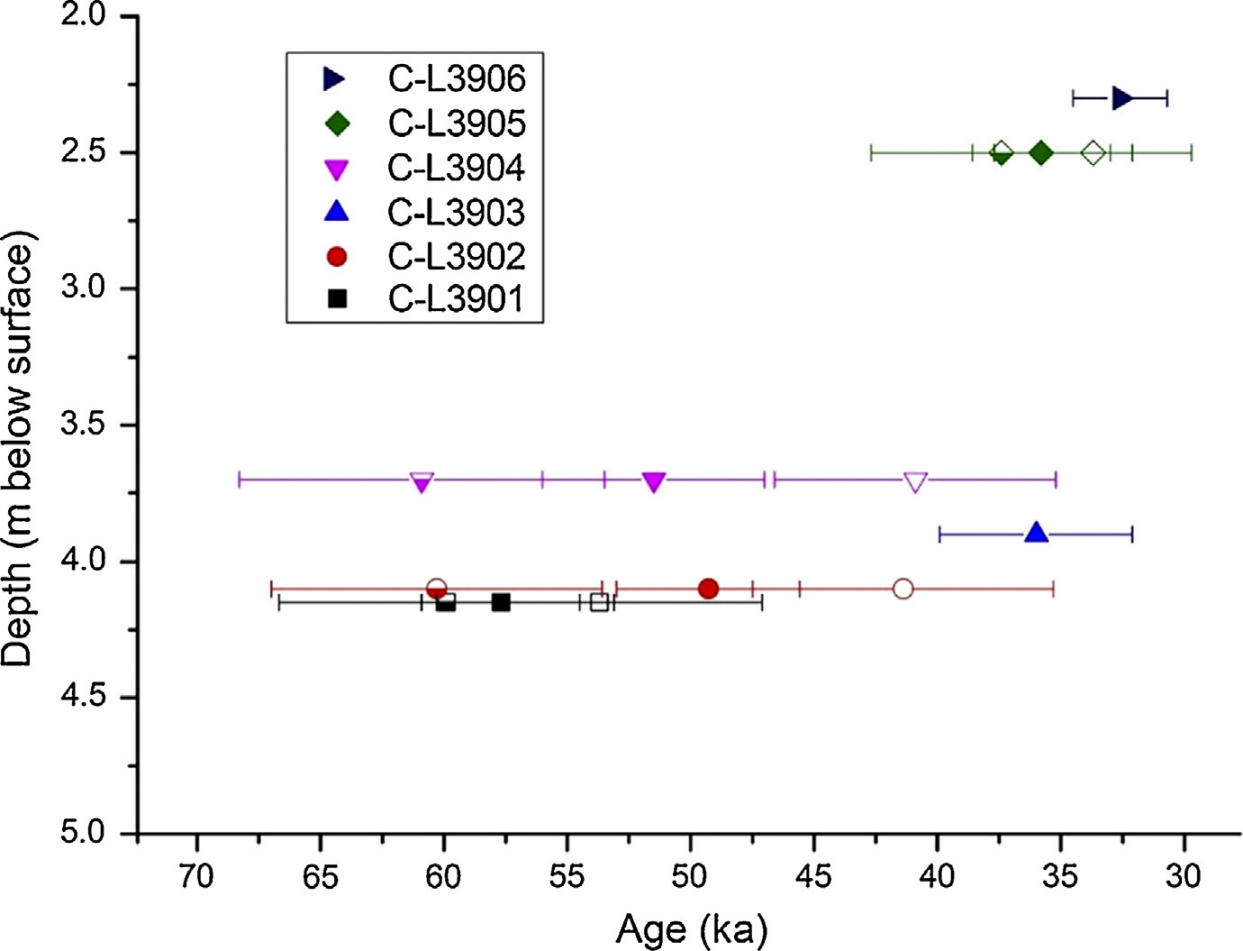
La Boja OSL dating. Age (±1σ) vs depth plot of luminescence dates. Filled symbols: quartz OSL results. Open symbols: feldspar IR_50_ results. Half-open symbols: feldspar pIRIR_290_ results.

Sediment samples from the Mousterian (OH21-OH23) and the Aurignacian (OH17-OH18) of La Boja were also dated by Optically Stimulated Luminescence (OSL) (Table 3; Figs. 6–8 ; Fig. S4.9). The multiple-grain dating of the quartz and feldspar minerals places the sequence between 32.6 ± 1.9 ka (C-L3906), for OH17, and 59.9 ± 6.8 ka (C-L3901), for the base of the deposit, below OH23. These luminescence ages are in complete agreement with the radiocarbon results for the corresponding Aurignacian and Mousterian horizons.

**Table 3:** La Boja. Dose rate data, equivalent dose values and luminescence ages. The cosmic dose was calculated after Prescott and Hutton (1994); the conversion factors of Guérin et al. (2011 and an assumed water content of 5 ± 2% were used. The internal beta dose rate contribution of the feldspar samples was calculated by assuming a potassium content of 12.5 ± 0.5%, after Huntley and Baril (1997), and an a-value of 0.12 ± 0.02.

Lab code	Mineral	Grain size (μm)	Accepted/measured aliquots (N)	U (ppm)	Th (ppm)	K (%)	Dose rate (Gy/ka)	RSD (%)	Age model	De (Gy)	Age (ka)
Sample LBJ6 (2.3 m below surface of cross-section); OH17											
C-L3906	Quartz	100–150	55/56	3.14 ± 0.16	1.71 ± 0.15	0.37 ± 0.01	1.35 ± 0.04	16	AM	43.9 ± 2.3	32.6 ± 1.9
Sample LBJ5 (2.5 m below surface of cross-section); OH18											
C-L3905	Quartz	100–150	39/40	3.09 ± 0.16	1.53 ± 0.14	0.33 ± 0.01	1.28 ± 0.04	30	AM	45.9 ± 3.2	35.8 ± 2.8
	K-F IR_50_	100–200	25/25	3.09 ± 0.16	1.53 ± 0.14	0.33 ± 0.01	2.02 ± 0.21	32	AM	51.1 ± 3.9	33.7 ± 4.0
	K-F pIRIR_290_	100–200	25/25	3.09 ± 0.16	1.53 ± 0.14	0.33 ± 0.01	2.02 ± 0.21	32	AM	91.8 ± 7.5	45.4 ± 5.6
									MAM	75.5 ± 7.5	37.4 ± 5.3
Sample LBJ4 (3.7 m below surface of cross-section); OH21											
C-L3904	Quartz	100–150	40/45	3.54 ± 0.18	1.44 ± 0.12	0.30 ± 0.01	1.33 ± 0.04	24	AM	68.4 ± 5.6	51.5 ± 4.5
	K-F IR_50_	100–200	12/12	3.54 ± 0.18	1.44 ± 0.12	0.30 ± 0.01	2.11 ± 0.20	13	AM	65.9 ± 4.1	40.9 ± 5.7
	K-F pIRIR_290_	100–200	21/21	3.54 ± 0.18	1.44 ± 0.12	0.30 ± 0.01	2.16 ± 0.21	26	AM	131.2 ± 10.0	60.9 ± 7.4
Sample LBJ3 (3.9 m below surface of cross-section); OH22											
C-L3903	Quartz	100–150	31/32	3.39 ± 0.18	1.51 ± 0.14	0.30 ± 0.01	1.30 ± 0.04	51	AM	46.7 ± 4.9	36.0 ± 3.9
Sample LBJ2 (4.1 m below surface of cross-section); OH23											
C-L3902	Quartz	100–150	103/131	3.36 ± 0.17	1.61 ± 0.13	0.32 ± 0.01	1.31 ± 0.04	47	AM	64.6 ± 4.4	49.3 ± 3.7
	K-F IR_50_	100–200	13/13	3.36 ± 0.17	1.61 ± 0.13	0.32 ± 0.01	2.09 ± 0.20	20	AM	59.9 ± 4.5	41.4 ± 6.1
	K-F pIRIR_290_	100–200	15/15	3.36 ± 0.17	1.61 ± 0.13	0.32 ± 0.01	2.03 ± 0.20	14	AM	128.7 ± 7.4	60.3 ± 6.7
Sample LBJ1 (4.1 m below surface of cross-section); basal											
C-L3901	Quartz	100–150	19/20	3.55 ± 0.19	1.58 ± 0.14	0.30 ± 0.01	1.33 ± 0.04	20	AM	80.6 ± 6.6	57.7 ± 3.2
	K-F IR_50_	100–200	13/13	3.55 ± 0.19	1.58 ± 0.14	0.30 ± 0.01	2.11 ± 0.20	6	AM	75.5 ± 4.0	53.7 ± 6.6
	K-F pIRIR_290_	100–200	15/15	3.55 ± 0.19	1.58 ± 0.14	0.30 ± 0.01	2.16 ± 0.21	15	AM	129.6 ± 8.1	59.9 ± 6.8

F = feldspar; K = Potassium; Th = Thorium; U = Uranium; AM = Arithmetic Mean; De = equivalent dose; IR_50_ = infrared stimulated luminescence signal at 50 °C; MAM = Minimum Age Model; pIRIR_290_ = post-infrared infrared stimulated luminescence signal at 290 °C; RSD = relative standard deviation.

**Fig. 9:**
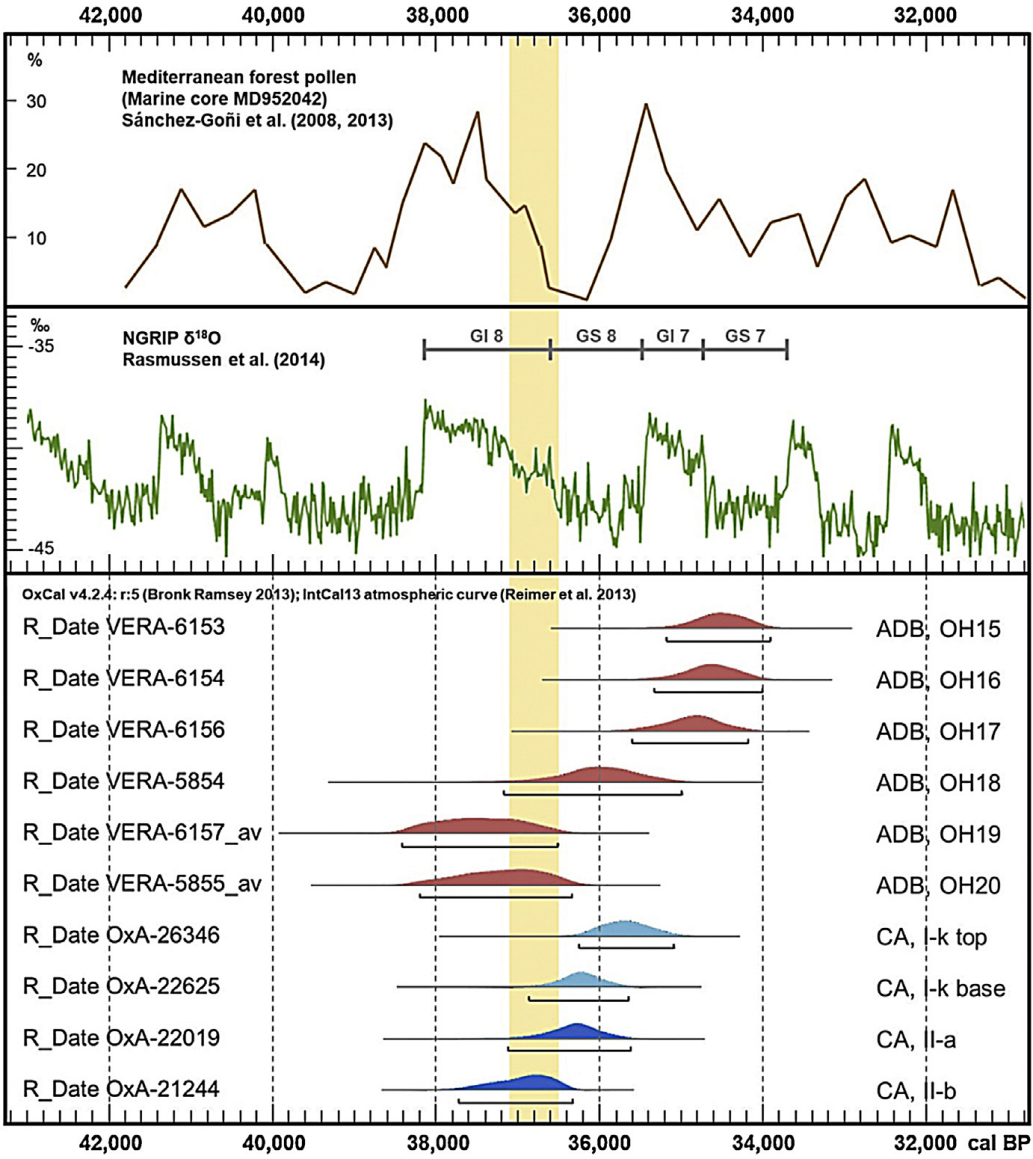
Chronology of the Middle-to-Upper Paleolithic transition in the Mula basin sites. Plot of calibrated radiocarbon dates (95.4% probability intervals) for the Aurignacian of La Boja and for the Mousterian (layer I-k) and immediately underlying alluvium (layers II-a and II-b) of Cueva Antón. The vertical yellow band denotes the interval during which the transition took place: between 36.5 ka, the youngest possible age of La Boja’s Aurignacian in OH19-20, and 37.1 ka, the oldest possible age of the Cueva Antón Mousterian as provided by the layer II-a *terminus post quem*. The comparison with the global proxies (Rasmussen et al., 2014, Sánchez-Goñi et al., 2008, Sánchez-Goñi et al., 2013) shows that, in the Mula basin, the transition coincides with the end of a long and mild temperate phase, Greenland Interstadial 8.

The ages of the Late Mousterian in layer I-k of Cueva Antón and of the Evolved Aurignacian in OH18-OH20 of La Boja overlap (Fig. 9). As the occupation events recorded at these sites are of short duration, a possible interpretation of this pattern is that the two assemblage types coexisted in the region for an extended period, during which their makers would have made infrequent, alternating incursions into the River Mula and Rambla Perea valleys. If so, Middle Paleolithic material ought to exist within the basal Aurignacian of La Boja as (a) discrete, interstratified lenses, or (b) isolated elements mixed in the OH18-OH20 assemblages. As neither is the case, the regional contemporaneity between the bearers of the two kinds of stone tool technologies must have been short-lived. Therefore, the dating overlap must primarily reflect the statistical uncertainty inherent to radiometric dating. Under these priors, CA/I-k and ADB/OH18-OH20 can be treated as two consecutive phases of the regional chrono-stratigraphic sequence.

Whether the charcoal found in layer I-k of Cueva Antón is anthropogenic, environmentally accumulated, or both, cannot be ascertained. However, the basal AS1 alluvium consists of lenses of fine, sandy-silty alluvium deposited in quick succession during low-energy inundation events; such kinds of events are also largely responsible for the matrix of the I-k breccia (Angelucci et al., 2013). This record’s resolution implies that any temporal difference that may have existed between human occupation and charcoal deposition must be negligible. Nevertheless, to be conservative, the age of the Late Mousterian in layer I-k is best constrained using the *terminus post quem* represented by the underlying units, layers II-a and II-b.

**Fig. 10:**
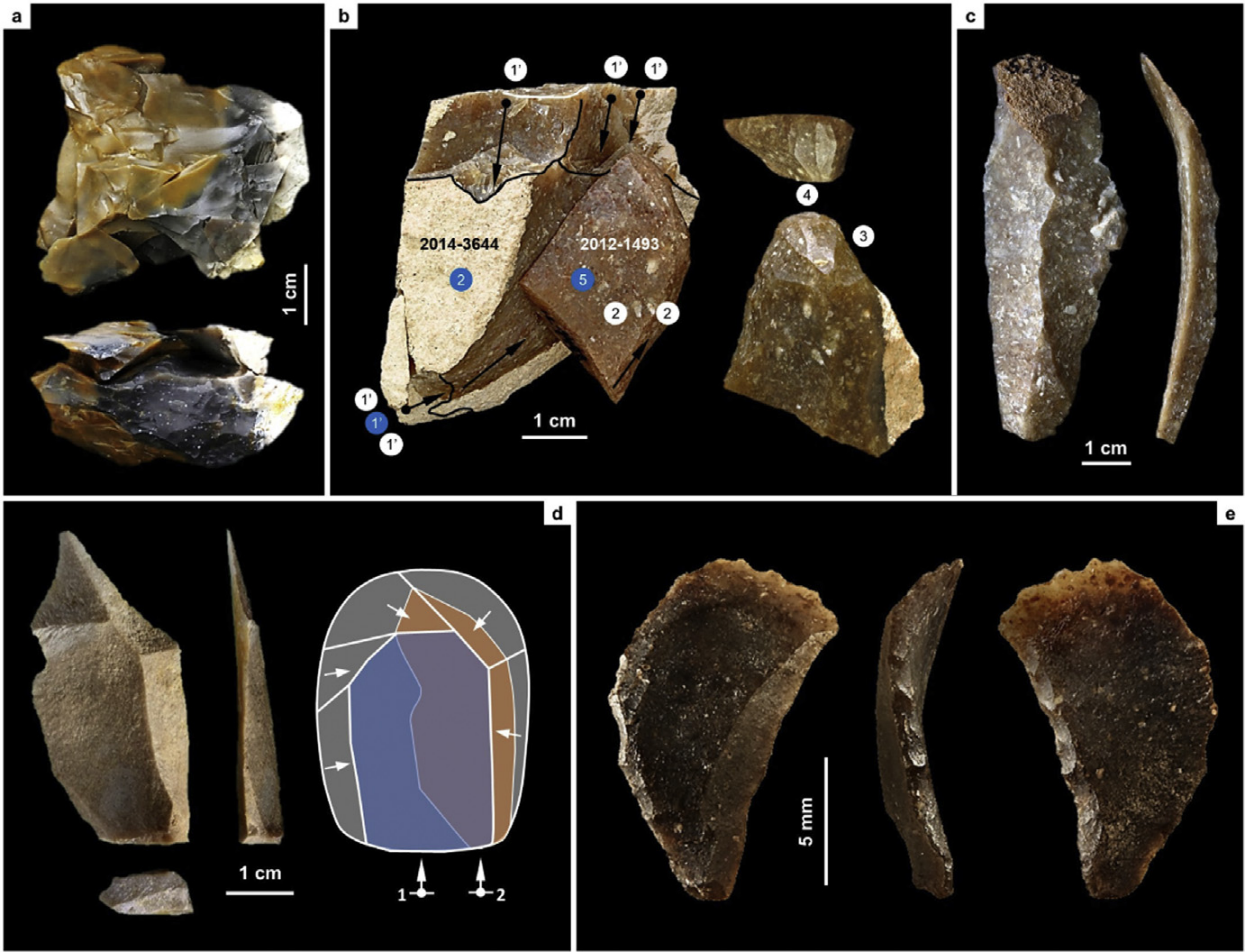
Blank production and diagnostic stone tools across the Middle-to-Upper Paleolithic transition in the the Mula basin sites. a. Centripetal core for small flakes, with refits (Cueva Antón, layer I-k, Mousterian). b. Multi-step reduction sequence for the production of bladelets (La Boja, OH20, Aurignacian): preparation (1) or re-preparation (1′) of a prismatic core for the extraction of long, thick blades (2), followed by preparation of such laminar blanks as carinated or nosed “scrapers” (3), extraction of bladelets from the “scraper front” (4), and eventual discard of the exhausted “scraper”/core (5); the blue circles denote steps represented in the refit, the white circles denote steps represented by removal scars or among the block’s unrefitted material. c. long blade with minor, proximal break (La Boja, OH20, Aurignacian). d. Laminar Levallois flake, representing a lateral removal after the extraction of a preferential flake in a Levallois recurrent reduction sequence (Cueva Antón, layer I-k, Mousterian). e. Characteristically twisted Dufour bladelet of the Roc-de-Combe subtype extracted from a carinated or nosed “scraper”/core (La Boja, OH17, Aurignacian).

That layers II-a and II-b provide indeed a robust maximum age for the human occupation of layer I-k is intimated by the archeological sterility of the basal AS1 alluvium, to which those two layers belong. Such sterility precludes interpreting the artefact assemblage in overlying layer I-k as inherited via some sort of local post-depositional process. In addition, (a) the stratigraphic integrity of the AS1 package is accredited by the absence of disturbance features across its total thickness and entire excavated extent, and (b) the mode of accumulation of layer I-k implies that its artefact content cannot have been inherited via fluvial transport from an earlier Middle Paleolithic site located elsewhere in the landscape. The stone tool refits (Fig. 10; Fig. S2.18), which document on-site production, corroborate the homogeneity, integrity, and in situ nature of both the artefact assemblage and its stratigraphic context. There can be no doubt, therefore, that, at Cueva Antón, the human activity recorded in layer I-k post-dates the time of deposition of layers II-a and II-b.

At La Boja, the age of the successful, hearth-collected sample from OH19 (2014-3348; 33,233 ± 335 BP, VERA-6157_av) is statistically indistinguishable from that obtained for immediately underlying OH20 and represents a direct record of human activity. OH19 and OH20 both contain diagnostically Upper Paleolithic, specifically Aurignacian, tool-kits. Thus, their dating sets an unambiguous *terminus ante quem* for the end of the region’s latest Middle Paleolithic.

Under this reasoning, the earliest possible age of Cueva Antón’s latest Mousterian is 37.1 ka, and the youngest possible age of La Boja’s Aurignacian is 36.5 ka, in calendar years. The yellow band in Fig. 9 represents the interval bounded by these dates. It was within this interval that, after a coexistence and interaction period of unknown duration, the region’s Neandertal-associated Late Mousterian was replaced by the modern human-associated Evolved Aurignacian.

### 2.2 Composition of the artefact assemblages

Jarama VI, a cave site in the Iberian hinterland once thought to span the MP-UP transition, illustrates well how issues of definition are as much implicated in the Neandertal persistence debate as those of dating accuracy and sample association: upon closer examination, the “Upper Paleolithic” stone tools retrieved in the levels capping the site’s Pleistocene succession turned out to be of Mousterian affinities instead (Kehl et al., 2013). Clearly, the robustness of the Mula basin’s chronology also depends on whether the artefact assemblages associated with the dated samples do represent the two sides of the regional transition.

**Fig. 11:**
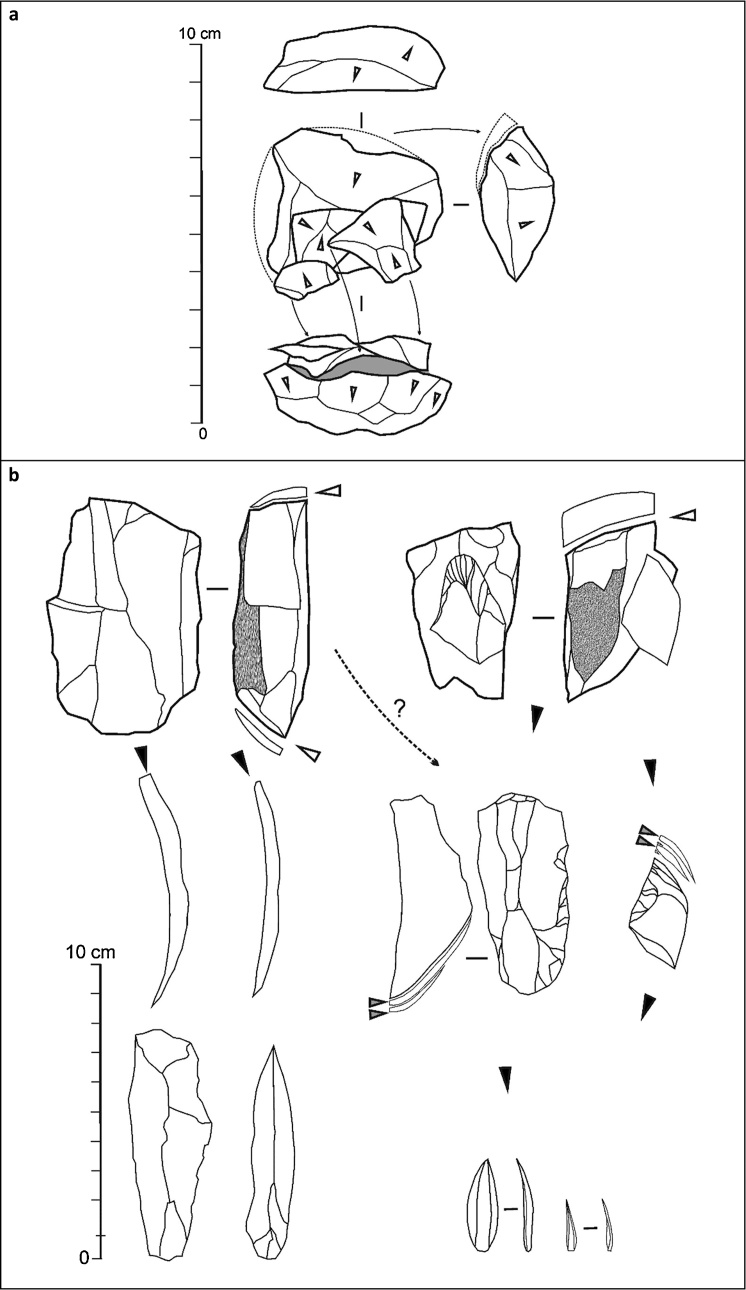
Core reduction methods across the Middle-to-Upper Paleolithic transition in the Mula basin sites. a. Simplified, schematic rendition of the approach to core reduction represented by the refitted material from Mousterian layer I-k of Cueva Antón (Fig. 10a); the refitting unit documents the endpoint, prior to discard, of the centripetal production of small flakes from a core previously exploited for similar blanks and in similar manner (as indicated by the shape and radial patterning of the flaking scars). b. Simplified, schematic rendition of the core reduction methods represented in the Evolved Aurignacian (OH20) of La Boja (Fig. 10b-c); two types of blades are extracted from prismatic cores — thin, to be used as a tool or as a blank for a retouched tool, and thick, to be used as a blank for bladelet cores of the carinated or nosed kind; thus, the latter’s intended end-products are bladelets obtained separately, not at the end of a continuous, blade-then-bladelet core reduction sequence.

Fig. 10, Fig. 11 illustrate the key aspects of lithic technology supporting our assignments: method of core reduction, and type of blank that production is designed for.

In layer I-k of Cueva Antón, the following methods, which are exclusive to the Middle Paleolithic, are found (Figs. S2.17-S2.19): Centripetal, Levallois or Discoid, core reduction, represented by a core, refitted flakes, and debris; Discoid, represented by imported core-trimming, or deliberately overshot, naturally backed flakes bearing notched or denticulated edges; Kombewa, represented by a core discarded in an initial stage of the reduction; and Levallois, represented by an imported laminar flake.

In La Boja OH18-OH20, only two methods, both unknown in the regional Middle Paleolithic, are found (Figs. S4.39-S4.43): prismatic for the extraction of blades and bladelets, represented by cores, débitage, and refitted sets; and carinated/nosed “scraper” reduction, also including refitted sets and represented by all steps of the sequence (initial large core for long-and-thick blades used as blanks for the extraction of the intended bladelets, the abandoned bladelet cores, the bladelets themselves, and the waste produced as the “scraper” front was reduced, trimmed and reconfigured). The Dufour bladelet in Fig. 10 is a typical example of the Roc-de-Combe subtype, an index fossil of the Evolved Aurignacian. It comes from OH17, but this and other subtypes of Dufour bladelets occur through the OH15-OH20 sequence (Figs. S4.41-S4.43). They are also present, alongside the characteristic carinated/nosed “scrapers”/cores, in layer 8 of Finca Doña Martina (Figs. S3.31-S3.32). In OH15 and OH16 of La Boja, backed microliths (Fig. S4.43, nos. 4–5) appear for the first time alongside these characteristic Aurignacian items, suggesting that the emergence of the succeeding Gravettian likely corresponds to a technological transition with no major discontinuity in population, demography, or settlement.

Well-stratified Portuguese examples show that specialized site occupancy may generate lithic assemblages that, despite their Upper Paleolithic age, lack the period’s diagnostics. This evidence questions automatic assignment to the Middle Paleolithic of similar assemblages, the more so if they are small (Wood et al., 2013). However, unlike layer I-k of Cueva Antón, those Portuguese assemblages also lack Middle Paleolithic diagnostics: they contain no items (either cores or blanks) indicating that the Discoid, Levallois and Kombewa reduction methods were in use at the time of production. A case in point is the assemblage from the EE15 occupation surface of the Lagar Velho rock-shelter (N = 593) (Almeida et al., 2009). Here, the idiosyncrasy relates to the situational context (reduction of immediately available quartzite cobbles for the expedient production of cutting edges used in carcass-processing tasks), and is of no wider chrono-stratigraphic consequence.

The mutually exclusive presence/absence of diagnostic technologies in the Mula basin sites stands despite differences in assemblage size of up to two orders of magnitude, and is consistently seen across time (Table 4). In this regard, the Late Mousterian in layer I-k of Cueva Antón is no different from the Middle Paleolithic assemblage of MIS 5 age found in the site’s layer II-l (Tables S2.2–S2.5). Likewise, the equivalently small size of the Early Gravettian assemblages in OH13-OH14 of La Boja (Tables S4.22–S4.25) is no impediment for their fully Upper Paleolithic nature to manifest itself through such diagnostics as bladelets extracted from both prismatic and “burin” core-types, the “burins” themselves, and even the technocomplex’s index fossil (a microgravette point). Much the same applies to La Boja’s Aurignacian assemblages (Tables S4.10–S4.21). At Finca Doña Martina, the lower resolution of the stratigraphic sequence means that each unit samples, and averages out, much longer time intervals. Yet, it remains that (a) Levallois and Discoid cores and blanks, sidescrapers, and denticulates are found together in this site’s basal layer 9 (Tables S3.3–S3.5, Figs. S3.29–S3.30) but not in overlying layers 8, 7b and 6/7, while (b) the reverse is true of prismatic, carinated/nosed “scraper” and “burin” core-types, endscrapers, or bladelet tools (Tables S3.7–S3.12; Figs. S3.31–S3.33).

**Table 4:** Cueva Antón and La Boja stone tools. Assemblage size versus representation of the diagnostic lithics.

		CA		ADB^a^							
Categories^b^	Diagnostics	II-l	I-k	OH20	OH19	OH18	OH17	OH16	OH15	OH14	OH13
Cores											
MP	Kombewa	–	1	–	–	–	–	–	–	–	–
	centripetal	1	1	–	–	–	–	–	–	–	–
UP	carinated/nosed	–	–	2	2	1	2	–	–	–	–
	burin	–	–	–	–	1	2	–	1	–	1
	prismatic	–	–	4	2	3	6	11	1	2	–
Unretouched blanks											
MP	Kombewa	1	–	–	–	–	–	–	–	–	–
	Levallois	1	1	–	–	–	–	–	–	–	–
UP	blades	–	–	18	–	–	2	7	8	–	–
	bladelets	–	–	37	6	9	42	51	18	6	3
Formal tools											
MP	sidescrapers	6	–	–	–	–	–	–	–	–	–
	denticulates	–	1	–	–	–	–	–	–	–	–
UP	endscrapers	–	–	1	–	1	–	1	–	–	–
	bladelet tools	–	–	3	2	1	1	14	7	1	–
Total^c^		26	14	179	59	69	285	371	77	22	14
Total^d^		34	20	453	146	202	923	1543	231	82	35

a OH15-OH20, Aurignacian, OH13-OH14, Early Gravettian (IL4 and IL3 items counted under OH20 and OH13, respectively).

b MP = Middle Paleolithic diagnostics; UP = Upper Paleolithic diagnostics.

c Debris (chippage and chunks), manuports and hammerstones excluded.

d Debris included.

The variation in the size and composition of these assemblages is primarily due to local factors. At Cueva Antón, the patches of dry sediment available for settlement inside the cave during the time of formation of layers II-l and I-k were restricted and surrounded by inundated or boggy riverside terrain (Figs. S2.11, S2.16). As shown by the taphonomy of the abundant rabbit bone, the site functioned as an eagle-owl roost throughout, which is inconsistent with frequent or intensive human presence (Sanchis, 2012; Zilhão et al., 2016). Likewise, the spatial restrictions to habitation caused by a massive roof collapse explain the small size of the artefact scatter around the hearth in La Boja’s OH13 horizon (Fig. S4.21).

The spectrum of activities reflected in the use-wear data for layer I-k of Cueva Antón is limited to wood-working (Table S2.5; Fig. S2.19), which is in keeping with the highly transient nature of the occupation(s). In the Rambla Perea sites, raw-material economy patterns indicate no significant change in site function across the transition. In the residential versus logistical balance of hunter-gatherer settlement-subsistence systems — as gauged by the relative importance of domestic- versus hunting-related stone tools — the scales were somewhat tipped in favor of the latter in the Early Gravettian and the Aurignacian of Finca Doña Martina, but not in the Aurignacian of La Boja (SI appendix, chapters 3–4).

**Fig. 12:**
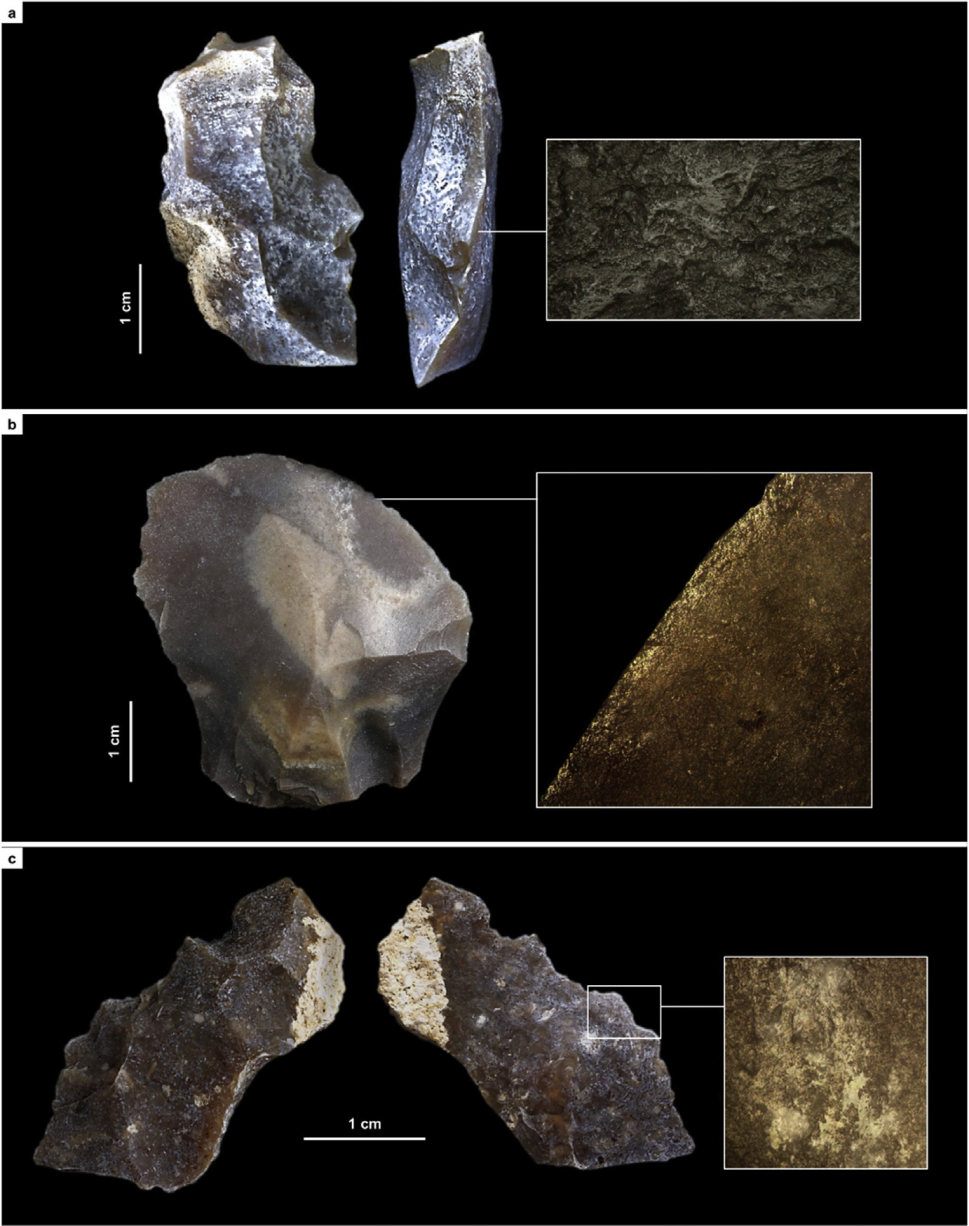
Middle Paleolithic wood-working tools in the Mula basin sites. a. Denticulate from Cueva Antón (layer I-k). b. Unretouched blank from La Boja (OH23). c. Denticulate from La Boja (OH23). The insets show characteristic microscopic polish. Note the similarity of the two denticulates, both made on orange-segment or discoid-overshot blanks; denticulates of this kind are entirely absent from top to bottom of the long and complete Upper Paleolithic sequences of La Boja and Finca Doña Martina (for additional detail, see the SI appendix).

**Fig. 13:**
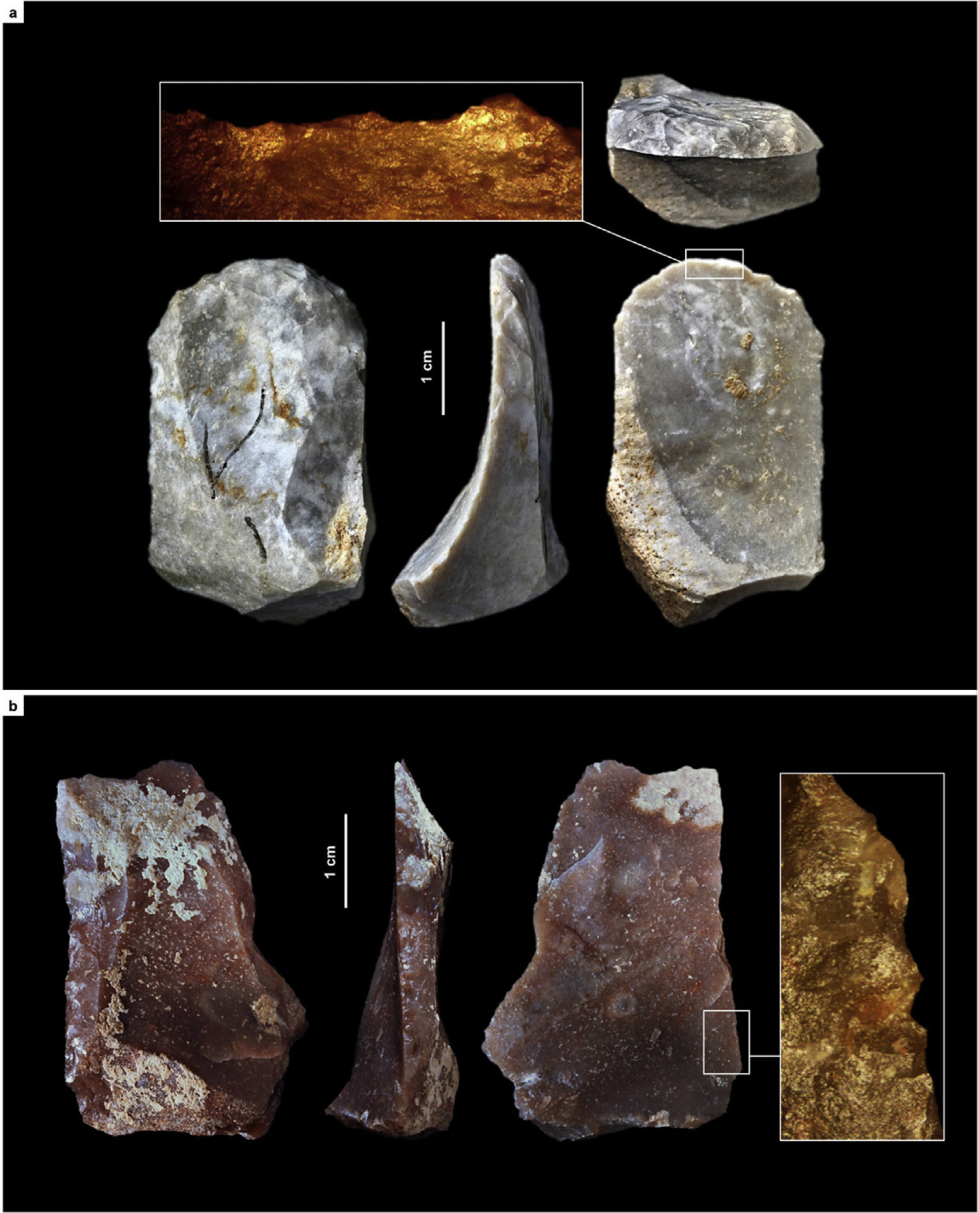
Hide-working tools across the Middle-to-Upper Paleolithic transition at Finca Doña Martina. a. Endscraper from Aurignacian layer 8. b. Sidescraper from Mousterian layer 9. The insets show characteristic microscopic polish (for additional detail, see the SI appendix).

**Fig. 14:**
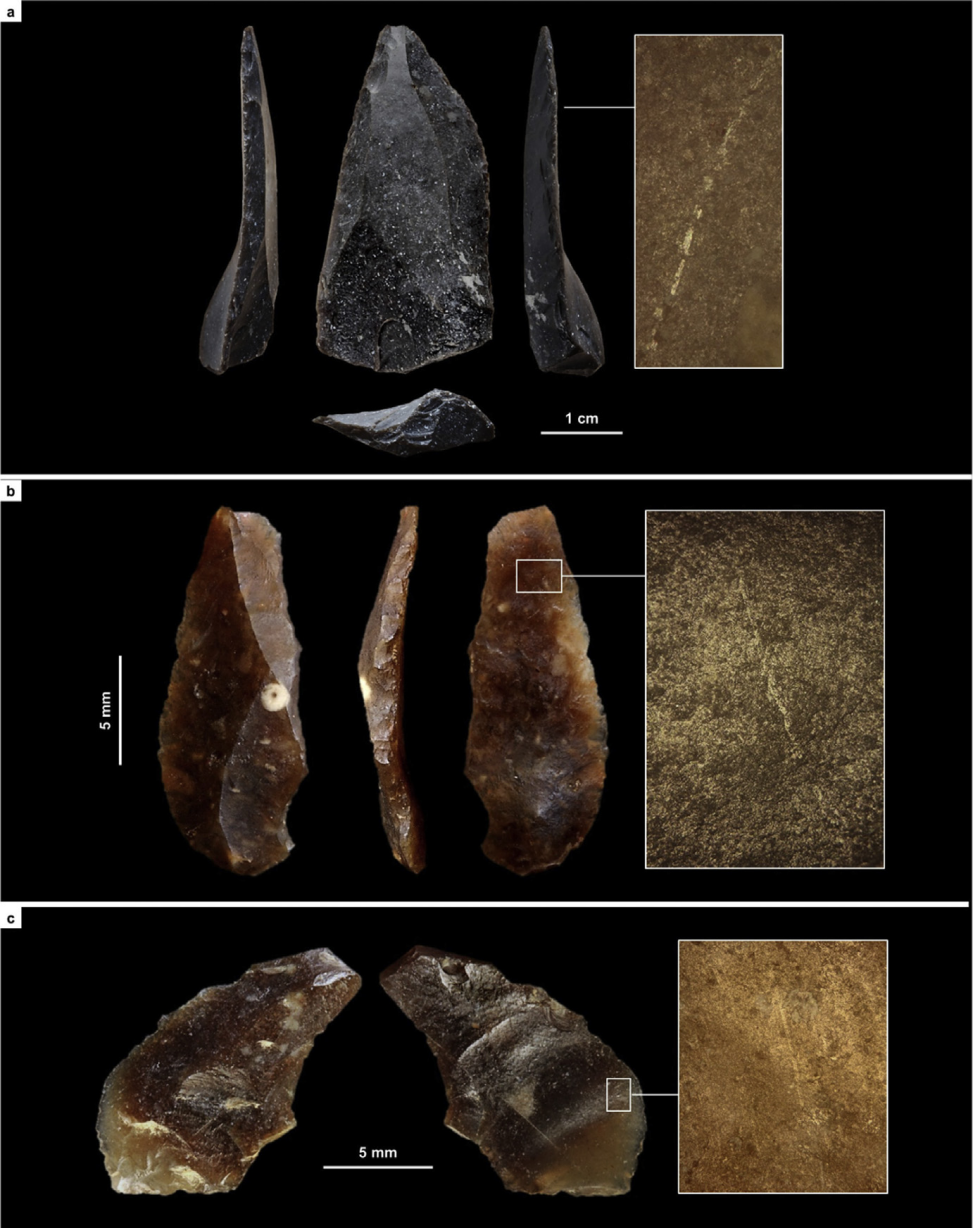
Projectile technology across the Middle-to-Upper Paleolithic transition in the Mula basin sites. Axial points in the Mousterian, composite points armed with cutting, laterally mounted, microlithic elements in the Aurignacian. a. Mousterian point from Finca Doña Martina (layer 9). b. marginally backed bladelet from La Boja (OH16). c. Dufour bladelet from Finca Doña Martina (layer 8). The insets show characteristic microscopic striations generated by impact (for additional detail, see the SI appendix).

For the Rambla Perea rock-shelters, lateral variation between two adjacent archeological sites that, in the living past, must have functioned as a single, spatially extensive locus of human activity, suffices to explain the contrasts between coeval lithic assemblages. Through time, across the regional MP-UP transition, the use-wear evidence shows that the differences are primarily of a techno-typological nature. Hide-working, wood-working, defleshing and the use of projectiles are documented in both the Mousterian and the Aurignacian (Tables S3.6, S4.9; Fig. 12, Fig. 13, Fig. 14 ; Figs. S3.30–S3.32, S4.37–S4.38, S4.41). However, (a) hides were processed with sidescrapers in the Mousterian but with endscrapers in the Aurignacian, and (b) projectiles were armed with single, axially-mounted points in the Mousterian but with multiple, laterally-mounted microlithic elements in the Aurignacian. In short, synchronic functional variability cannot explain the differences in lithic technology upon which we have assigned the stone tool assemblages of the Mula basin sites to either the Middle or the Upper Paleolithic.

**Fig. 15:**
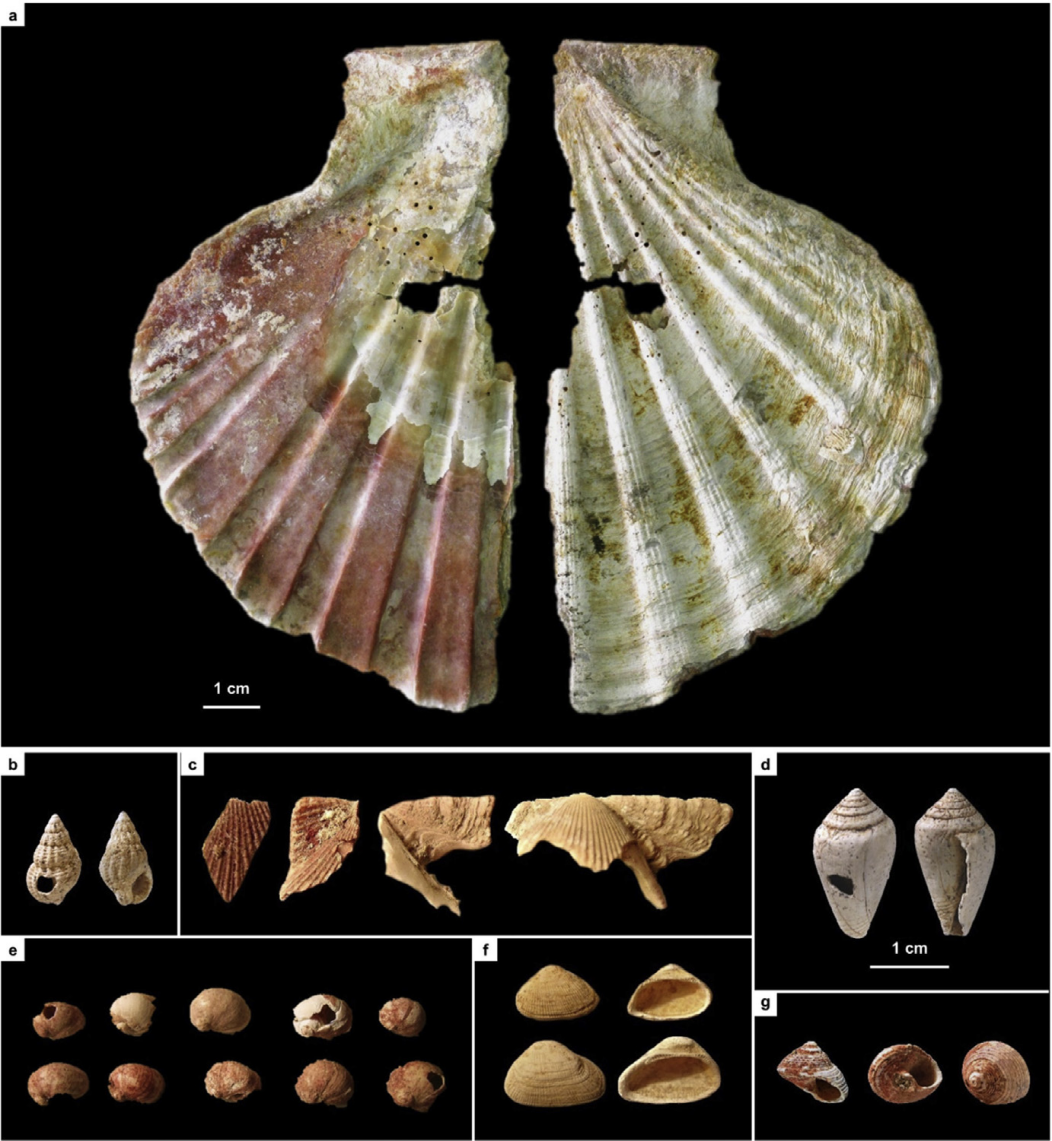
Ornamental shell across the Middle-to-Upper Paleolithic transition in the Mula basin sites. a. *Pecten* half-valve from Middle Palaeolithic layer I-k of Cueva Antón (after Zilhão et al., 2010a); the reddish color of the internal side is natural; remnants of an orange colorant made of goethite and hematite are visible in the side that was painted (the external, whitish one). b–g. perforated and/or ochre-stained bivalve and gastropod shell (all at the same scale) from the Aurignacian of La Boja (for additional detail and taxonomic identifications, see the SI appendix).

Ochre is often involved in the processing of hides, as documented by residue on a Mousterian sidescraper from Finca Doña Martina (Fig. S3.30). No such residues were found in the lithics from layer I-k of Cueva Antón. Thus, the pigment cover of the associated scallop shell (Fig. 15; Fig. S2.20) cannot represent accidental or post-depositional staining by iron oxides brought in for hide-processing tasks or locally produced by diagenetic processes. Much the same applies to the ornamental shell assemblage of quite distinct composition found in the Aurignacian of La Boja (Table S4.2; Fig. 15; Figs. S4.32, S4.34). This assemblage features ubiquitous red ochre staining even though none was found in the 78 stone tools from OH15-OH20 examined for use-wear (Table S4.9). These findings further strengthen the symbolic interpretation previously advanced for Cueva Antón’s ochred scallop (Zilhão et al., 2010a).

## 3 Discussion

### 3.1 Dating accuracy

At La Boja, the archeological sequence’s radiocarbon chronology is independently supported by the OSL dating of the basal Mousterian and of the Aurignacian. At Cueva Antón, layer I-k could not be OSL-dated for two main reasons: (a) prior to 20th-century burial by silts accumulated during intermittent periods of submersion under the La Cierva reservoir the layer was exposed as a surface for an undetermined amount of time, implying significant uncertainty with regards to environmental radiation parameters; and, (b) coupled with its limited thickness in the cross-sections exposed at the time of sampling, its high stone content (layer I-k is a clast-supported breccia with few fines) made this layer inappropriate for luminescence dating (Burow et al., 2015).

From within the radiocarbon method itself, the Cueva Antón and La Boja charcoal samples passed all the reliability tests currently available. The dates allowing us to bound the Mula basin’s Mousterian-to-Aurignacian transition belong to long series of results that are fully stratigraphically consistent, both internally (within each site) and externally (across sites and with the broader, regional and supra-regional framework).

At La Boja, the humic fraction was also measured to assess the potential impact of contamination. The accuracy of the chronology obtained on the fraction processed with the ABA (Acid-Base-Acid) treatment is supported by (a) the identical results obtained whenever the dating of individual samples was repeated, and (b) the lack of statistical difference between the results obtained for individual samples processed with both ABA and ABOx-SC (Acid-Base-Oxidation with Stepped Combustion) (based on Bird et al., 1999).

At Cueva Antón, the ABA protocol was found to slightly underestimate the age of the samples, and the success rate of ABOx (26%; five out of 19) was lower than at La Boja (Zilhão et al., 2016). However, the Cueva Antón samples surviving the ABOx-SC pretreatment had a high %C, which, following Rebollo et al. (2011), is a good indicator that the material that survived was well preserved. In addition, given the aggressiveness of the treatment, the percentage of failed samples is not unexpected; similar rates have been reported when using ABOx-SC for samples derived from contexts dated to broadly the same time interval (Brock and Higham, 2009).

### 3.2 The latest Middle Paleolithic south of the Ebro

**Fig. 16:**
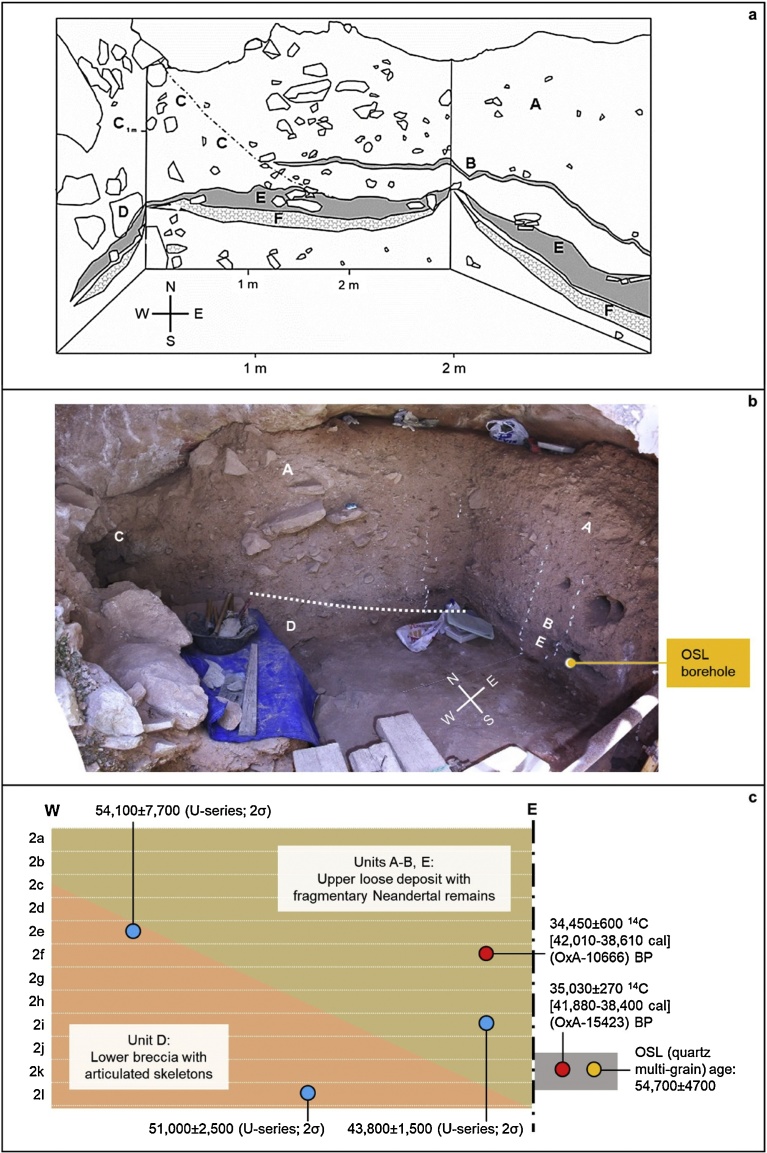
Sima de las Palomas de Cabezo Gordo, Upper Cutting. a. Schematic drawing of the stratigraphy [after (Walker et al., 2008) (Walker et al., 2012), modified]. b. Composite mosaic view over the north and east walls of the Upper Cutting excavation trench during the 2007 field season. c. Schematic position of the radiocarbon- and U-series-dated samples relative to stratigraphy and arbitrary horizontal spits of provenience (2a-to-2l).

The dating work carried out at the site of Sima de las Palomas (Fig. 1, no. 2), on the coast of Murcia, ca.60 km to the Southeast of Cueva Antón, provides further support for the late persistence of the Middle Paleolithic in the region — in this case, with diagnostic Neandertal remains found stratigraphically together with the lithics (Walker et al., 2008; Trinkaus and Walker, 2017). Correct understanding of the significance of the dates obtained at this key site is hindered by the samples’ provenience notations referring to arbitrary horizontal spits that do not reflect the stratigraphic layout of the sequence — something misunderstood by Wood et al. (2013) and Santamaría and de la Rasilla (2013), although explicitly stated in Walker et al. (2008). When the actual stratigraphy is considered, the dating results— obtained by radiocarbon on burnt bone treated with the ABA protocol, U-series on bone using Diffusion/Adsorption (D/A) assumptions, and multi-grain quartz OSL on sediments — are mutually consistent (Fig. 16).

The U-series dates for Sima de las Palomas show that (a) the accumulation of the lower cemented deposit containing articulated Neandertal skeletons (unit D) took place prior to 46.4 ka, (b) provide a *terminus post quem* of 53.5 ka for the accumulation of the overlying deposit containing fragmentary Neandertal remains (units A-B and E), and (c) suggest for the base of the latter an age younger than 45.3 ka. The OSL result is less precise and, because of the existence of remnants of an older sedimentary fill brecciated against the walls and roof of the cave, could be affected by incomplete bleaching; even so, when its 95.4% probability interval (45.3–64.1 ka) is considered, it agrees with the U-series results.

Taken together, the OSL and U-series dates are in turn consistent with the two radiocarbon dates from samples retrieved at the same stratigraphic elevation or higher up in units A-B and E. The uppermost radiocarbon result (OxA-10666) is from a faunal fragment cemented to a diagnostic Neandertal mandible that was (a) found half-way through the unit A deposit and (b) overlain by ca.50 cm of sediment containing nothing but diagnostic Middle Paleolithic stone tools and diagnostic Neandertal remains. As OxA-10666 translates into a calibrated age within the 38.6–42.0 ka interval, the Sima de las Palomas evidence strongly indicates, in line with the Cueva Antón pattern, that the Middle Paleolithic persisted in the region well beyond 40–42 ka. In addition, it shows that such a late-persisting Mousterian is indeed a Neandertal-associated technocomplex. There is no reason, therefore, to question that the association pertains in those other parts of Iberia where stratigraphy and dating support persistence of the Middle Paleolithic into the same time range: Gibraltar and Portugal.

At Gorham’s Cave (Gibraltar; Fig. 1, no. 3), an uncalibrated date of 32,280 ± 420 BP (OxA-7857) was obtained for a charcoal sample recovered in stratigraphic association with diagnostic Middle Paleolithic stone tools within Context 24 of the Natural History Museum’s (NHM) 1995–1998 excavations (Pettitt and Bailey, 2000). In Middle Paleolithic layer IV of the Gibraltar Museum’s 1999–2005 excavations at the rear of the cave, an uncalibrated date of 32,330 ± 390 BP (OxA-10230) was obtained in the same laboratory, and a separate set of samples yielded uncalibrated dates ranging between 23,780 ± 540 BP (Beta-185345; 2σ) and 32,560 ± 780 BP (Beta-196771; 2σ) (Finlayson et al., 2006; Finlayson et al., 2008). In calendar years, these results imply persistence of the Middle Paleolithic in Gibraltar until at least 36.0–37.8 ka (the 95.4% probability interval of the calibration of Beta-196771).

The Beta samples from Gorham’s all underwent the standard ABA treatment, but the younger ones probably reflect stratigraphic intrusion because, at the rear of the cave, a several millennia-long hiatus makes for direct contact between Mousterian layer IV and Solutrean layer III (Zilhão and Pettitt, 2006). The OxA results, in turn, come from samples processed with the gentler RR treatment, which does not include a base wash (Brock et al., 2010; Wood et al., 2013).

Even though OxA-10230 was a large pine cone scale that, per Bronk Ramsey et al. (2002), made for reliable dating material, Wood et al. (2013) assume that the RR treatment was insufficient to remove all contamination from the Gorham’s OxA samples. Based on this assumption, they argue that no confidence can be placed in the notion that the site’s Middle Paleolithic significantly post-dates 40–42 ka. However, they did not test the RR results via processing of remaining material in storage, or of new samples, with ABA or ABOx-SC (they report no additional charcoal dating, only failed attempts at extracting collagen from associated animal bone). In addition, the RR-treated charcoal samples from the NHM excavations collected lower down in the Gorham’s sequence returned results as old as 51,700 ± 3300 BP (OxA-7790). If the latter were to be taken as a byproduct of incomplete decontamination producing a finite result for a sample of infinite radiocarbon age, the unremoved contaminant, if modern (i.e., F^14^C = 1), could represent no more than 0.16% of the measured carbon. For OxA-10230, modeling such a level of contamination shifts the uncalibrated radiocarbon result from 32,330 to 33,069 BP, which is, given the standard deviation, statistically the same thing.

Against this background, arguing that higher levels of contamination characterized the samples coming from the upper part of Gorham’s Mousterian sequence (but only those…) would be special pleading. The more so because the general reliability of the OxA results for the Gibraltar sites’ RR-processed charcoal samples is otherwise implied, in the case of stratigraphic units 53–55 of Vanguard Cave, by their agreement with the luminescence ages obtained for the same deposit: radiocarbon’s RR results were between 41,800 ± 1400 BP (OxA-6998) and 54,000 ± 3300 BP (OxA-6891), OSL’s was 46.32 ± 3.30 ka (OxL-1029) (Pettitt and Bailey, 2000).

In Portugal, layer 8 of the Gruta da Oliveira cave site (Fig. 1, no. 4) yielded an unquestionably Middle Paleolithic stone tool assemblage (Marks et al., 2001). Its radiocarbon dating on burnt bone treated with ABA at Groningen and with RR at Oxford yielded statistically indistinguishable results of, respectively, 31,900 ± 200 BP (GrA-10200) and 32,740 ± 420 BP (OxA-8671) (Angelucci and Zilhão, 2009). In calendar terms, these two radiocarbon results, which translate into a 95.4% probability interval comprised between 35.3 and 38.2 ka, are statistically identical to three U-series (D/A) dates on bone from the same layer (Hoffmann et al., 2013).

The time span indicated by the rich, single-occupation Mousterian open-air site of Foz do Enxarrique, near the Spanish border (Fig. 1, no. 5), is the same (Raposo, 1995). Here, the weighted average of the dates obtained by U-series on the tooth enamel of one bovid and two horse samples is 33.6 ± 0.5 ka. The accuracy of this chronology is dependent on the uncertain validity of the Early Uptake assumption underpinning the calculation of the ages, while the nature of the association between the dated faunal remains and the stone tools is an open issue. Indeed, per Brugal and Raposo (1999), the site’s faunal assemblage is primarily a natural riverside thanatocenosis, with only the cervid component bearing marks indicative of a human activity-related accumulation. The two multi-grain, K-feldspar OSL results since obtained at the site for the base of the alluvial sands within which the archeological level is contained (the T5 unit of the local terrace staircase of the Tagus) are, therefore, a better, if less precise estimate of the time of deposition of the stone tool assemblage. At 34.8 ± 1.3 and 38.5 ± 1.6 ka (after correction for anomalous fading) (Cunha et al., 2008), the OSL results support an age post-dating 40 ka for the site’s occupation — and, thus, that the Middle Paleolithic persisted in interior Iberia beyond the time of emergence of the Early Aurignacian in the Cantabrian strip and northern Catalonia.

### 3.3 The earliest Upper Paleolithic south of the Ebro

The persistence of a Neandertal-associated Middle Paleolithic from Iberia’s Mediterranean Southeast to its Atlantic seaboard implies that archeological manifestations of the modern human-associated Aurignacian I not be found across the same territory. Such is indeed the case. Neither stratigraphic units containing diagnostic assemblages nor isolated index fossils of the Early Aurignacian have been identified in the long cave sequences spanning the MP-UP transition known in those parts of the peninsula: Cova Beneito (Valencia), Cueva Bajondillo (Andalusia), Gorham’s Cave (Gibraltar), and Gruta do Caldeirão (Portugal) (Zilhão, 2006a). At these sites, and at others that are either open-air, single-occupation localities, or lack a basal Middle Paleolithic, the earliest Upper Paleolithic is the Aurignacian II (Evolved Aurignacian) or III–IV (a.k.a. Late Aurignacian).

Technologically, the Aurignacian II is defined by the débitage of carinated and thick-nosed “scrapers”/cores producing characteristically twisted blanks transformed into Dufour bladelets via inverse or alternate retouch, while the Aurignacian III–IV is characterized by the predominance of carinated and other “burin” types of bladelet cores. However, as demonstrated at La Boja, the microlithic diagnostics of the Aurignacian II persist to the end of the Aurignacian sequence. Therefore, in the absence of reliable dating, or of a technologically representative assemblage of cores and débitage products, the presence of such microliths, even though sufficient to exclude appurtenance to the Aurignacian I, does not exclude assignment to the Aurignacian III–IV. When stratigraphic sequences are not resolved to the level of detail seen at La Boja, the possibility that assemblages containing Dufour bladelets correspond to palimpsests that subsume both phases (Aurignacian II and III–IV) cannot be excluded either.

In Mediterranean Spain, the assemblages from Beneito, the rock-shelter of Ratlla del Bubo (Iturbe and Cortell, 1992), and the cave site of Cova de Mallaetes (Fortea and Jordá, 1976), all in Valencia, and from Bajondillo, are examples of clearly post-Aurignacian I collections that cannot be precisely assigned to one of the succeeding phases of the technocomplex. In the Beneito and Ratlla del Bubo assemblages, which remain undated, backed elements are found alongside the characteristic Dufour bladelets. This coexistence has led some to question the validity of the industrial diagnosis, or the integrity of the sedimentary contexts (de la Peña and Vega, 2013). However, based on the evidence from horizons OH15-OH16 of La Boja, the coexistence suggests instead that the Beneito and Ratlla del Bubo assemblages either are Late Aurignacian or include a component belonging to that phase. The Mallaetes context lacks diagnostic stone tools but yielded lozenge bone points in association with a conventional charcoal date of 29,690 ± 560 BP (KN‐I/926). The Bajondillo context contains diagnostics suggestive of the Aurignacian II and is dated to 33,690 ± 1195 BP (Ua‐17150) and 32,770 ± 1065 BP (Ua‐18050); however, given the inadequate nature of the samples (of “sediment and charcoal”) and the imprecision of the results, appurtenance to the succeeding Aurignacian III–IV cannot be excluded. A related problem exists with the two large, well-studied stone tool assemblages from the open-air Aurignacian sites of the Rio Maior basin, in Portugal: Gato Preto’s is of Aurignacian II affinities but is dated by Thermoluminescence (TL) and therefore with a large 95.4% probability interval, 30.3–45.9 ka; and Vale de Porcos’s, technologically of Aurignacian III–IV affinities, remains undated (Zilhão, 2006b).

It has been proposed that the diagnostic microlithic tool-type of the Late Aurignacian is an elongated, straight variant of the Dufour bladelet pointed by alternate retouch (Zilhão et al., 2010c). This variant is known from layer 2 of the cave of Pego do Diabo, in Portugal, and from the disturbed, surficial deposits capping the Mousterian sequence of Cueva de Zafarraya, in Andalusia. At the Portuguese site, the Pleistocene fauna associated with the small assemblage of such Dufours yielded four AMS radiocarbon dates on tooth samples treated with both the Longin and the ultrafiltration protocols. Under the stringent criterion of considering reliable only those samples for which both the standard gelatin production and the >30 kDa (thousands of Daltons) ultrafiltered production yielded statistically identical results, the Pego do Diabo deposit accumulated between 29,090 ± 270 BP (VERA-4047) and 30,260 + 330/-320 BP (VERA-4050). The earlier result overlaps those for OH15-OH16 of La Boja, but the later one extends the range for another millennium, until ca.33 ka. Because the dated fauna is non-anthropogenic, however, it cannot be ascertained whether the “Pego do Diabo points” (a) stand for a “Final” phase, dating beyond 34.0 ka, of the Aurignacian technocomplex in Western Iberia, as the younger result might suggest, or (b) are a component of the ca. 34.0–35.5 ka Late Aurignacian, as indicated by the earlier result. If the second hypothesis is retained, the implication would be that the microlithic tool-kit of the Late Aurignacian was more diverse than so far documented in Valencia and Murcia.

Be it as it may, the Mula basin sites suffice to demonstrate that, by 36.5–37.1 ka, the Aurignacian II was already present in Iberian regions to the South of the Ebro basin. This interval is the same during which, based on Bayesian modeling of available dates, Banks et al. (2013b) found that the transition from the Early to the Evolved Aurignacian had occurred to the North. This technological transition would therefore seem to have been concomitant with a process of settlement expansion: in Northern Europe, toward the British Isles and equivalent latitudes of Germany and Poland that, during the previous phase, had become devoid of human occupation; in Iberia, toward the lands beyond the Ebro basin, eventually leading to replacement of their late-persisting Mousterian and the assimilation of its Neandertal makers. The “Ebro Frontier” model provides a biogeographical and paleoecological framework for the interpretation of these developments in terms of population history.

### 3.4 The “Ebro frontier”

In Iberia, the Ebro basin nowadays lies at the interface between two biogeographic regions defined after the distribution of plant communities: Eurosiberian and Mediterranean (Rivas-Martínez, 1987). The separation runs along the southern foothills of the Cantabro-Pyrenean mountains but, during the Upper Pleistocene, its very existence and latitudinal placement must have been dependent on the period’s highly variable and frequently oscillating climates.

During MIS 4, Eurosiberian steppe-tundra environments spilled into and beyond the Ebro basin well into the Iberian core. This is shown by the distribution of wooly rhino and mammoth finds: along the Mediterranean coast, down to the Llobregat delta, near Barcelona; in central Iberia, as far West as the Manzanares valley (Madrid) and as far South as the northern flanks of the Sierra Nevada (Granada) (Daura et al., 2013). During the Last Glacial Maximum (LGM), Europe’s Upper Pleistocene cold fauna (mammoth, wooly rhino, bison, reindeer) was again present in Catalonia, the Cantabrian strip, and parts of the northern Meseta but absent from Valencia, Murcia, Andalusia, and Portugal. These differences in the composition of the large herbivore fauna imply significant environmental gradients within the peninsula during MIS 4 and the LGM, albeit ones that (a) did not follow the present Eurosiberian/Mediterranean divide, and (b) given the shared aspects of stone tool technology and the widespread homogeneity in rock art styles observed through the Gravettian and most of the Solutrean all the way from Portugal, in the West, to the Rhone valley, in the East, did not represent significant barriers to the movement of people, the circulation of objects, or the exchange of ideas.

We also know that, during periods of extreme aridity such as the episode of iceberg discharge known as Heinrich Stadial (HS) 4, which lasted for a few centuries around ca.40 ka, the kinds of semi-desert environments nowadays confined to northern Almeria and southern Murcia expanded to the Mesetan hinterland and the badlands of the middle and upper Ebro basin (d’Errico and Sánchez-Goñi, 2003; Sepulchre et al., 2007). Conversely, during periods of milder, wetter climatic conditions such as Greenland Interstadial (GI) 8 (ca.38.2–36.6 ka), mountain forests and wooded landscapes underwent a very significant expansion below the latitude of 40°N (Fletcher et al., 2010). Judging from what happened in the Holocene, during such milder periods human settlement must have retracted to the resource-richer littoral areas, leading to the breaking-up of exchange and communication networks, and favoring the emergence of cultural/biological isolates.

Based on this evidence, the “Ebro Frontier” model hypothesizes that steppe-tundra environments would have been continuously present in Northern Iberia through the entire MP-UP transition process and that, during this period, the Ebro basin would have functioned as a major physical and biogeographical divide due to: (a) the establishment of semi-desert conditions in the basin itself, the northern flanks of the Iberian Range, and the Mesetan hinterland, in HS4, and (b) the development in adjacent lands to the South and West, both before and after this extreme aridity event, of extensive mountain forests and open woodlands. At present, this hypothesis remains difficult to test, because the paleoenvironmental data available are insufficient to reconstruct, with the spatial and temporal resolution required, the impact of these climatic oscillations on the ecosystems of the territory across which the environmental gradient developed. However, the divergent cultural-historical trajectories followed either side of the “Ebro Frontier” after ca.45 ka — namely, the failure of the Châtelperronian, the Protoaurignacian and the Aurignacian I to extend southward — do imply the presence of a major, long-lasting barrier to migration, gene flow and diffusion.

The spread of the Aurignacian II into Southern and Western Iberia signals the disappearance of the conditions underpinning the preceding pattern of cultural divergence, whatever their cause. That paleoenvironmental factors must have played a role is nonetheless intimated by the temporal coincidence of the replacement of Iberia’s late-persisting Mousterian (ca.36.5–37.1 ka) with the global climatic transition from GI 8 (the longest and mildest of all MIS 3 insterstadials) to Greenland Stadial (GS) 8 (a “normal” cold phase) (Rasmussen et al., 2014). During this transitional period, the Eurosiberian steppe-tundra could and likely did begin to spread into the Iberian core, while the charcoal from sub-complex AS1 of Cueva Antón (12% cryophilous pines, 85% steppic taxa, 3% riverside taxa; Zilhão et al., 2016: Fig. 8, SI Table 2) indicates a descent of the montane pine forest belt from above 1100 m to below 400 m, in agreement with the near disappearance of Mediterranean forest taxa seen at this time in the deep-sea pollen record (Fig. 9).

The presence of a major biogeographical gradient along the Ebro basin acquires broader paleoanthropological significance because of the period when it happened to be separating modern humans and Neandertals. In and of itself, however, the existence at that time of such a gradient, with attendant implications for diffusion and exchange, in no way should be mistaken for something exceptional or unique. After the LGM, for instance, the Ebro basin would come to separate moderns (Badegoulian and Early Magdalenian) from other moderns (Upper Solutrean and Solutreo-gravettian) for a comparable duration — three to four millennia (Banks et al., 2009). Conversely, prior to 42 ka the Ebro basin had already been separating Neandertals (Châtelperronian) from other Neandertals (Mousterian) — and may well have continued to do so for another couple thousand years if Neandertals were also involved in the manufacture of the Protoaurignacian.

The Protoaurignacian is well documented along the shores of the Cantabrian Sea, from the Basque sites of Isturitz and Labeko Koba in the East to the Asturian site of La Viña in the West (Zilhão, 2006a). Even though no archeologically associated diagnostic human remains have so far been found across the Protoaurignacian’s entire geographical range (Bulgaria to northern Spain) and temporal span (39–42 ka), the genome of the Oase 1 adult male shows that he had had a “pure” Neandertal ancestor only four to six generations back (Fu et al., 2015). Combined with the age of the fossil (directly dated by radiocarbon to 37.1–41.4 ka) (Trinkaus et al., 2013), this genomic evidence implies a strong probability of overlap between Neandertals and at least the beginnings of the Protoaurignacian. The latter’s industrially “intrusive” characteristics and similarity with the Near Eastern, modern human-associated Early Ahmarian suggest an intrinsic relation to modern human immigration. The technological innovations the Protoaurignacian stands for, however, could well have diffused into Neandertal territory well in advance of the arrival of the admixture front. Since no evidence exists that an “archeological culture = human type” equation applies to the Protoaurignacian, it remains entirely plausible, therefore, that it was also made by variously mixed Neandertal-modern human, or even “pure” Neandertal populations — and especially so in the West (Trinkaus and Zilhão, 2013; Zilhão et al., 2015).

If Neandertals were also involved in the making of the Protoaurignacian, then it is only in Aurignacian I times, after 40 ka, that the Ebro basin represented a Neandertal/modern human “frontier.” If so, the emergence of such a “frontier” would have been broadly coincidental with the 39.9 ka explosion of the Phlegraean Fields caldera, whose ash fall-out blanketed vast stretches of Italy and Southeastern Europe, severely disrupting food chains for an extended period — the highest trophic levels, including human hunters, being most impacted. For the populations of Western Europe, which was not directly affected, the main consequence of the explosion would have been to bring about a release from the constraints of demographic pressure induced across the continental landmass by the previous millennia of population growth and Neandertal assimilation. In this scenario, the explosion would have constituted a historically contingent but significant factor contributing to explain why Middle Paleolithic Neandertals persisted for so long in the territories of Europe’s Far West (Zilhão 2009; Fitzsimmons et al., 2013; Marti et al., 2016; Giaccio et al., 2017).

Whether, at the time of this catastrophic event, the Neandertal/modern admixture front had already reached the Pyrenees and the Cantabrian strip for quite some time or had just arrived there remains an open issue. But, whichever the case, the explosion’s impact on the modern human populations of Central and Eastern Europe would have stalled the westward expansion of the front after ca.40 ka. If a biogeographical gradient was then extant across the Ebro basin, the demographic crisis caused by the Phlegraean Fields explosion would have enhanced that gradient’s effect. And if, with the return to normal stadial conditions, following the end of GI 8, that effect ceased to operate, it would have done so at a time when replenishment of the Central/Eastern European sink created by the explosion would also have reset demographic pressure over the peripheries. For Northern Europe, the consequence would have been resettlement. For Iberia, it would have been the eventual assimilation of the last of Europe’s Neandertals, as postulated by the “Ebro Frontier” model. Both expectations are met by the empirical record.

## 4 Conclusions

The technological and use-wear evidence rejects interpreting layer I-k of Cueva Antón and occupation horizons OH20 and OH19 of La Boja as distinct structural poses of a single, multifaceted system. Put another way, the small lithic assemblage in layer I-k of Cueva Antón cannot be interpreted as a functionally specialized, or activity-specific facies of the region’s Evolved Aurignacian. Instead, layer I-k of Cueva Antón and occupation horizons OH20 and OH19 of La Boja stand for concrete manifestations of mutually exclusive, long-lasting technologies whose succession, rather than a gradual transition, truly consisted of an abrupt replacement. As the efficiency of stone tool production in terms of cutting edge per unit of mass is identical in both technologies (Muller and Clarkson, 2016), the parsimonious reading of this replacement process is that it represents a major break, with demic underpinnings, in regional cultural trajectories.

The evidence from stone tool technology and the stratigraphic layout of sites is that the pattern derived from the high-precision Mula basin data can be extrapolated to all Iberian regions to the South of the Ebro basin. In these regions, artefact assemblages attributable to the earliest phases of Western Europe’s Upper Paleolithic are missing from stratified sites that contain deposits spanning the MP-UP transition, and have never been found as single-component, open-air contexts. In addition, no isolated occurrences of their index fossils (e.g., Châtelperronian points/knives, or Aurignacian split-based bone points) have ever been reported among surface, mixed, or post-depositionally disturbed deposits. From the basics of Prehistoric Archeology, i.e., from the culture-stratigraphic reasoning providing the framework for all its chronologies, the only inference that one can derive from this pattern is that, southward of the Ebro basin, a late-persisting Mousterian occupies the time slot in which the Aurignacian I is found elsewhere. The radiocarbon evidence is entirely consistent with this notion, which available luminescence and U-series independently support, and which no other kinds of radiometric dating results have so far countered.

A corollary of these findings is that Neandertals persisted until ca.37 ka across Southern and Western Iberia — which carries implications for the authorship of all other aspects of these regions’ archeological record. For instance, given their dating and archeological associations, there can be no question that the painted/perforated shells from Cueva Antón and Cueva de los Aviones, as well as the abstract engraving and ornamental use of raptor feathers documented at Gorham’s Cave, stand for manifestations of Neandertal symbolism (Zilhão et al., 2010a; Finlayson et al., 2012; Rodríguez-Vidal et al., 2014). Knowing that minimum ages of 40.8 ka for a red disk and 37.3 ka for a hand stencil have been obtained at El Castillo cave (Cantabria) (Pike et al., 2012), and that such motifs exist in Extremaduran and Andalusian sites, it is easy to see how the “Ebro Frontier” pattern may also bear implications for the authorship of cave paintings.

Recent advances in the field of Genetics increasingly make it clear that, in the Late Pleistocene of Eurasia, the continental extension of rather homogeneous archeological cultures is superimposed on complex ancestry patchworks (Mallick et al., 2016; Pagani et al., 2016). This can be explained by a pattern of long-distance diffusion and cultural resilience, which maintained networks over the long-term, combined with extended periods of geographical isolation, which conserved regional genetic variants. The “Ebro Frontier” effect makes this mechanism apparent even in the refugia of Southern Europe and especially so at the time of the MP-UP transition. This visibility is due to when the frontier formed and for how long it lasted, both allowing the effect to be picked-up with the current resolution of dating techniques. Likely, however, similar, broadly coeval but chronometrically less visible Late Pleistocene frontiers must have existed in other parts of Asia and Europe, as well as during the earlier phases of the process of modern human dispersal into these continents.

The results we report here highlight the need for proper integration of the biological and the archeological evidence when reconstructing Late Pleistocene population histories. All lines of evidence are now converging to support replacement-through-admixture, or Assimilation, as the best explanation for the disappearance of the Neandertal and other archaic phenotypes. The Iberian evidence suggests this was a time-transgressive evolutionary outcome stemming from dynamic, complex and geographically uneven processes — a punctuated history in which the long-term maintenance of pan-continental networks of gene flow and cultural exchange did not exclude the occurrence of extended periods of significant geographical isolation.

## 5 Materials and methods

### 5.1 Archeological excavation and analysis

Excavation proceeded through décapage along observed boundaries, whether natural (e.g., the interface with the underlying geological stratigraphy) or anthropogenic (e.g., the base of distinct occupation floors stacked up within a single natural stratigraphic unit), with subdivisions when necessary. Finds were piece-plotted with the help of a laser level, to the nearest centimeter, against grid and site datum. Use-wear analysis of stone tools was based on differential interference contrast microscopy, carried out with a BHMJ Olympus model (at × 200 or × 400 magnification), and followed standard recommendations for the cleaning and preparation of the material. Large samples of the sediment were floated for the recovery of paleobotanical data; the remainder was entirely dry-sieved using two-sieve stacks (2 and 1 mm mesh-sizes). The analysis of pollen, charcoal, mollusk shell and animal bone followed standard protocols. Stratigraphic cross-sections were geologically described, drawn and digitally recorded, as were the surfaces exposed at each step of the décapage process. At Finca Doña Martina, the DStrectch plug-in for ImageJ was used to highlight color contrasts and produce prints used in the field to help with the décapage of stratigraphic interfaces. Photo mosaics were assembled using PT GUI or Microsoft ICE and orthorectified with the University of Venice’s RDF software. Elevation maps and 3D models were produced with Surfer. Undisturbed soil and bulk sediment samples were collected for micromorphological, phytolith and biomolecular analysis.

### 5.2 Radiocarbon dating

Only securely provenanced, taxonomically classified charcoal samples were submitted for dating. All samples were treated with the ABA protocol, and the humic fractions of several samples were also measured (Wild et al., 2008). For Finca Doña Martina’s, a milder treatment was used for some, due to poor preservation; in most cases, only the humic fraction could be dated. The results for this site are therefore all minimum ages. At Cueva Antón, the ABA treatment proved insufficient to remove all contamination, but the chronology of layer I-k reported here is entirely based on results obtained for samples that were processed with the ABOx-SC protocol (Zilhão et al., 2016). To check if a similar problem existed at La Boja, some of its samples were also processed with ABOx-SC, in parallel to the standard ABA treatment (Wild et al., 2008) and using a modified version of the procedure given in (Brock et al., 2010), i.e. acid and base treatment at 60 °C. In addition, to control for the accuracy of individual measurements, some ABA-treated samples were dated twice. The ABOx-SC results and the repeats were in all cases statistically indistinguishable from the original ABA date. When more than one result for a single charcoal fragment was obtained, the corresponding average was used. Calibration was carried out with the INTCAL13 curve in Calib 7.0 (Stuiver and Reimer, 1993; Reimer et al., 2013). The Fig. 9 plot was prepared in OxCal 4.2.4 (Bronk Ramsey, 2009).

### 5.3 Luminescence dating

The ADB samples were extracted from macroscopically homogeneous silt-rich deposits (Fig. S4.9). Due to the unconsolidated nature of trench walls, it was decided not to drive metal cylinders into the sediment; instead, the samples were extracted with a knife, in complete darkness. Coarse grain quartz (100–150 μm) and potassium feldspar (100–200 μm) were extracted using conventional sample preparation techniques (Kehl et al., 2016). All measurements were carried out on an automated Risø TL/OSL DA 20 reader equipped with a calibrated ^90^Sr beta source and an EMI 9235 photomultiplier. Multiple-grain quartz samples were measured using the single-aliquot regenerative-dose protocol (SAR) (Murray and Wintle, 2000; Murray and Wintle, 2003), including signal stimulation by blue diodes (470 nm, FWHM = 20) and signal detection through a Hoya U340 filter. The initial 0.8 s of the signal minus a background of the last 5 s was used for quartz dating. Preheat plateau and dose recovery tests were carried out to check the suitability of the measurement protocol. Single-grain quartz dating was not feasible because of low signal intensities.

Multiple-grain potassium feldspar samples were measured using the post-infrared infrared stimulated luminescence signal measured at 290 °C (pIRIR_290_) (Thiel et al., 2011). Stimulation was carried out with infrared diodes (870 nm, FWHM = 40), and the signals were detected through an interference filter (410 nm). The initial 4 s of the signal minus a background of the last 20 s was used in the pIRIR dating. Prior IR stimulation temperature tests and dose recovery tests (24 h Hönle Sol2 bleaching) were carried out to check the performance of the measurement protocol. Equivalent doses were calculated using the arithmetic mean (AM), except for sample C-L3905, for which we also used the minimum age model (MAM) (Galbraith et al., 1999). Additionally, infrared stimulated luminescence measured at 50 °C (IR_50_) was applied (Wallinga et al., 2000; Preusser, 2003), and the signal was corrected for anomalous fading using the approaches of Auclair et al. (2003) and Huntley and Lamothe (2001).

Data analysis was carried out using the R luminescence package (Burow, 2017, Kreutzer, 2017, Kreutzer et al., 2017). The radionuclide concentrations of the surrounding sediments were measured using high resolution gamma-ray spectrometry. The dose rate was calculated using Dose Rate and Age Calculator (DRAC) (Durcan et al., 2015), and included conversion factors (Guérin et al., 2011) and an assumed water content of 5 ± 2%. The internal beta dose rate contribution of the feldspar samples was calculated by assuming a potassium content of 12.5 ± 0.5% (Huntley and Baril, 1997). The cosmic dose rate was calculated after Prescott and Hutton (1994). Dose distributions are displayed as abanico plots (Dietze et al., 2016) (Fig. 6, Fig. 7). Equivalent doses calculated with the arithmetic mean and the Central Age Model (CAM) are statistically indistinguishable at 1σ and finally the arithmetic mean was used.

A typical dose response curve and a decay curve are shown for quartz sample C-L3905 (Fig. 7a). Preheat plateau tests (Fig. 7b) indicated that the equivalent dose of the quartz is independent from temperature treatment in the ranges 180–240 °C (C-L3901), 220–280 °C (C-L3904), 180–280 °C (C-L3905), and 240–280 °C (C-L3906). Dose recovery tests showed that a laboratory given dose was best recovered using a temperature of 180 °C for samples C-L3901 and C-L3905 and of 260 °C for samples C-L3904 and C-L3906 (Fig. 7c). Prior IR stimulation temperature tests carried out for feldspar sample C-L3905 indicated a plateau between 80 °C and 180 °C (Fig. 7d). Laboratory doses were recovered with a ratio of the measured to the given dose of 1.07 ± 0.06 (a residual dose of 5 Gy after 24 h of bleaching in the Hönle Sol2 solar simulator was subtracted). A representative dose response curve for this feldspar sample is shown in Fig. 7e and the dose distribution in Fig. 7f.

The laboratory experiments confirmed the suitability of the measurement protocols for both quartz and feldspar minerals. Except for sample C-L3903, the quartz OSL age estimates are in stratigraphic order, scatter between 57.7 ± 3.2 ka and 32.6 ± 1.9 ka, and are consistent with the radiocarbon ages obtained for the same units. pIRIR_290_ and IR_50_ dating was carried out to investigate if the quartz OSL signal was likely to be fully bleached at the time of deposition. An internal crosscheck of the two minerals is advisable (Murray et al., 2012) because the pIRIR_290_ and IR_50_ signals bleach slower than the quartz OSL signal (Buylaert et al., 2012). Comparison of the mean age estimates of all three luminescence signals shows good agreement between the quartz OSL and feldspar IR_50_ and pIRIR_290_ ages of sample C-L3901. For sample C-L3905, the quartz (35.8 ± 2.8 ka) and IR_50_ age estimates are younger than the pIRIR_290_ age (45.4 ± 5.6 ka), which indicates incomplete bleaching of the feldspar pIRIR_290_ signal at deposition. This is supported by the good agreement of the quartz OSL and feldspar IR_50_ results with the calibrated radiocarbon age (34.9–37.1 ka; VERA-5854) obtained for the same stratigraphic unit, confirming complete bleaching of the OSL and IR_50_ signals. Applying a MAM to the feldspar pIRIR_290_ dataset results in an age of 37.4 ± 5.3 ka, which demonstrates that the MAM successfully extracts individual equivalent dose values from the distribution that are likely to be fully bleached at deposition. For samples C-L3902 and C-L3904, the pIRIR_290_ age estimates tend to overestimate the quartz and IR_50_ results. It was not possible to extract those individual equivalent doses from the distribution that are likely to have been completely bleached prior to deposition using the MAM. Quartz sample C-L3903 appears to be underestimated compared to the underlying samples and we value this result as an outlier.

## Declarations

### Author contribution statement

João Zilhão: Conceived and designed the experiments; Performed the experiments; Analyzed and interpreted the data; Wrote the paper.

Diego Angelucci, Valentin Villaverde, Josefina Zapata: Conceived and designed the experiments; Performed the experiments; Analyzed and interpreted the data.

Daniela Anesin, Thierry Aubry, Ernestina Badal, Dan Cabanes, Martin Kehl, Nicole Klasen, Armando Lucena, Ignacio Martín-Lerma, Susana Martínez, Henrique Matias, Davide Susini, Peter Steier, Eva Maria Wild: Performed the experiments; Analyzed and interpreted the data.

### Competing interest statement

The authors declare no conflict of interest.

### Funding statement

Archaeological fieldwork and research at Cueva Antón and the Rambla Perea rock-shelters was funded by the Dirección General del Medio Natural de la Región de Murcia, the Municipality of Mula, the University of Murcia, the Fundación Séneca (Murcia), the Ministerio de Ciencia e Innovación (grants HAR2011-24878, HAR2014-52671-P and CGL2012-34717), the Generalitat Valenciana (grant PROMETEOII/2013/016), the Excellence Research Projects Program of the Andalusian Government (grant P11-RNM-7033), and the Leakey Foundation. The German Research Foundation’s (DFG) project CRC 806 (“Our Way to Europe. Culture-Environment Interaction and Human Mobility in the Late Quaternary”) funded the luminescence dating.

### Additional information

No additional information is available for this paper.
