# Supplementary material for: Precise dating of the Middle-to-Upper Paleolithic transition in Murcia (Spain) supports late Neandertal persistence in Iberia
Source: Heliyon. 2017 Nov 16;3(11):e00435. doi: 10.1016/j.heliyon.2017.e00435 (PMC5696381; doi:10.1016/j.heliyon.2017.e00435)
Supplement: Supplementary information [file mmc1.pdf]

Precise dating of the Middle-to-Upper Paleolithic transition in Murcia (Spain) supports late Neandertal persistence in Iberia

SUPPLEMENTARY INFORMATION

Chapter 1.
Geographical setting and archeological context

Chapter 2.
The cave/rock-shelter of Cueva Antón

Chapter 3.
The rock-shelter of Finca Doña Martina

Chapter 4.
The rock-shelter of La Boja

Chapter 1.
Geographical setting and archeological context

1.1. THE MULA BASIN

Cueva Antón and the Rambla Perea rock-shelters are situated in the Mula basin, which occupies the geographical center of the Autonomous Region of Murcia, Spain (Fig. S1.1). The basin is a ca.700 km^2 tectonic depression — a graben filled with >1000 m-thick Miocene marls — delimited to the North, South, and West, by the Sierras of Ricote, Espuña, and Cambrón, respectively. These mountain ranges rise above 1000 m, while the elevation of the basin itself decreases gradually from ca.400 m along the western border to ca.200 m along the eastern border. The basin drains to the valley of River Segura, which communicates it with the littoral plains of Murcia and Campos de Cartagena extending to East and South (Fig. S1.1A).

The mean annual temperature is 19.7 °C. The mean annual rainfall ranges are 350-500 mm, in the Northwest, and 250-350 mm, in the lower elevation badlands extending downstream of the town of Mula. The dominant soil temperature regimes are thermic, and soil moisture regimes are aridic to xeric (García-Cortés et al., 1999). The current vegetation is a xerophytic brushwood with *Artemisia herba-alba*, *Rosmarinus officinalis* and *Stipa tenaccisima*. Aleppo pine (*Pinus halepensis*) and juniper (*Juniperus phoenicea*, *J. oxicedrus*) thrive in areas with deeper soils, while *Tamarix*, *Nerium oleander* and *Phragmites* occur along river margins.

The basin's extant surface morphology is structured by (a) ridges of diverse carbonate lithology (limestone, conglomerate, breccia, calcarenite) resulting from Pliocene and Pleistocene tectonics and (b) the incision of the streams that traverse the basin on their way to the Segura, namely River Mula and its tributaries. The Pleistocene deposits formed in association with these landforms — cave and rock-shelter fills, slope deposits, alluvial terraces — are well known for their potential to preserve rich records of human occupation. Indeed, by the last decade of the 20th century, Upper Pleistocene archeological sites had already been documented to the Southeast (e.g., Sima de las Palomas de Cabezo Gordo; Walker et al., 2012), West (e.g., the Cueva Negra del Estrecho del Río Quípar; Walker et al., 2016), and North (e.g., the cave art sites around Cieza; Salmerón et al., 1999). It was not until the 1990s, however, that the basin's Paleolithic archeology was first revealed — at Cueva Antón (Martínez-Sánchez, 1997).

1.2. THE RESEARCH PROJECT

In 1929, River Mula was dammed just upstream of the town of the same name, resulting in the formation of the La Cierva reservoir. In the 1990s, the raising of the dam's wall triggered an assessment of environmental impact. Among the mitigation measures recommended, that assessment mentioned the archeological testing of a large cave located at the tail of the artificial lake, locally known as Cueva Antón (Fig. S1.1B). Eventually carried out in 1991, this salvage operation uncovered Middle Paleolithic occupation horizons buried in a thick, well-stratified fluviatile sequence. Based on correlation with a 5-7 m terrace dated elsewhere, the excavators estimated that the site's fill had accumulated between 38 and 40 ka (Martínez-Sánchez, 1997).

Interest in Cueva Antón was revived in 2005, in the context of preliminary reconnaissance work for a project targeting the Middle-to-Upper Paleolithic transition in Iberia (Zilhão and Villaverde, 2008; Zilhão et al., 2010a). The reasons were twofold: (a) the nature of the sedimentary envelope warranted an expectation of high stratigraphic integrity for both artefacts and ecofacts, while (b) the age suggested by the 1991 study implied that the site's archeological content might well date to the time range of relevance to test the hypothesis of late Neanderthal persistence in Iberian regions located to the South of the Ebro drainage (the Ebro Frontier model; Zilhão, 1993). Cueva Antón was therefore selected for additional excavation and analysis related to the Middle Paleolithic component of the project.

The Rambla Perea gorge, a deeply incised, meandering valley located ca.1.5 km north of Cueva Antón, was surveyed at the same time. The watercourse flowing along the bottom of this gorge is the main left tributary of River Mula. Prior to diversion for flash-flood control, this stream was active year-round because the permanent spring it originates in, Fuente Caputa, is found in correspondence with a tectonically active escarpment and drains a large underground aquifer. In the initially surveyed section (Fig. S1.1C), the valley runs almost straight, following a tectonic alignment. This disposition determines an asymmetric transverse profile and, along its northern slope, the presence of outcrops of Upper Miocene biocalcarenite that form near-vertical, continuous, up to 30 m-high rock walls. Such morphology favored the formation of rock-shelters, while the year-round availability of water, controlled by local geology more than annual rainfall, was unlikely to have been significantly affected by past climatic oscillations. Combined, these factors suggested that, for humans, the Rambla Perea would have been a focal point of the landscape. Hence, it could be expected to contain archeological sites upon which to base the project's Upper Paleolithic component.

Indeed, flints of unambiguous Upper Paleolithic affinities were seen at the base of one of the gorge's rock faces during a first visit to the valley carried out on December 11, 2005; this locality was thence designated Finca Doña Martina (FDM), after the owner of the farmstead below (Fig. S1.2). Based on the observation that a large and deep but emptied rock-shelter had formed downstream in the same bedrock strata, it was inferred that one could also exist, even though completely buried by slope deposits, at the foot of a rock face situated in intermediate position (Fig. S1.1C). Subsequent testing eventually confirmed the inference, and this new locality was thence designated as Abrigo de La Boja (ADB; Fig. S1.2), after the name locally given to the abundant wormwood shrubs covering the surface of the site and adjacent slopes.

The La Cierva reservoir inundated a similar gorge, El Corcovado, akin to the Rambla Perea even though shorter in length. The original depth of the El Corcovado incision can be pictured from the ca.100 m difference in elevation between riverbed and adjacent terrain observed at the damming site. Between this point and Cueva Antón, at the tail of the reservoir, River Mula bridged, over ca.1 km, a difference in elevation of some 50 m. In the regional landscape, these two nearby gorges play a similar role — communicating the Mula basin with the mid-elevation limestone plateau extending to the North of the Sierra de Ricote ridge (Fig. S1.1A). During the Pleistocene, for game and humans moving to, or coming back from this higher terrain, El Corcovado and Rambla Perea would have provided the shortest routes to do so.

Coupled with their proximity, the near-identical geographical setting of the three sites targeted by the project meant that access to raw-material and food resources could be held constant in terms of assessing whatever variation might be observed when comparing their archeological records. Thus, any aspects of change through time in technology, spatial patterning of the occupations, or functionality not accounted for by parallel change in the sites' morphology (e.g., due to erosion, collapse, or filling-up) could be assumed to relate to change in culture, social organization and settlement-subsistence system — i.e., the realms the Middle-to-Upper Paleolithic transition primarily manifests itself in.

Given the favorable conditions, funding was sought, secured, and a decade of field work ensued, beginning in 2006 at Cueva Antón with the cleaning-up of the debris accumulated at the bottom of the 1991 trench and the re-analysis of its exposed cross-sections. The time range covered by the correlation and combination of the stratigraphic sequences exposed extends from the Last Interglacial to the very end of the Pleistocene, i.e., from ca.90 to ca.10 ka. Details on the results obtained so far can be found in Zilhão et al. (2010b, 2016), Lucena et al. (2012), Angelucci et al. (2013, 2017), Román et al. (2013), Burow et al. (2015), and references therein. The chapters that follow provide overviews of each of the three sequences and contain supporting, detailed information on the issues of site formation, dating and assemblage definition underpinning current debates on the Middle-to-Upper Paleolithic transition in Iberia and beyond (Zilhão, 2006; Zilhão and Pettitt, 2006; Finlayson et al., 2008; Zilhão et al., 2010c; Kehl et al., 2013; Wood et al., 2013).

1.3. REFERENCES

ANGELUCCI, D.; ANESIN, D.; SUSINI, D.; VILLAVERDE, V.; ZAPATA, J.; ZILHÃO, J. (2013) — *Formation processes at a high resolution Middle Paleolithic site: Cueva Antón (Murcia, Spain)*. «Quaternary International», 315, p. 24-41.

ANGELUCCI, D.; ANESIN, D.; SUSINI, D.; VILLAVERDE, V.; ZAPATA, J.; ZILHÃO, J. (2017) — *A tale of two gorges: Late Quaternary site formation and surface dynamics in the Mula basin (Murcia, Spain).* «Quaternary International» (http://dx.doi.org/10.1016/j.quaint.2017.04.006315).

BUROW, C.; KEHL, M.; HILGERS, A.; WENIGER, G.-C.; ANGELUCCI, D. E.; VILLAVERDE, V.; ZAPATA, J.; ZILHÃO, J. (2015) — *Luminescence Dating of Fluvial Deposits in the Rock Shelter of Cueva Antón, Spain*. «Geochronometria», 42, p. 107-125.

FINLAYSON, C.; FA, D. A.; JIMENEZ ESPEJO, F.; CARRIÓN, J. S.; FINLAYSON, G.; GILES PACHECO, F.; RODRÍGUEZ-VIDAL, J.; STRINGER, C. B.; MARTINEZ RUIZ, F. (2008) — *Gorham's Cave, Gibraltar — The persistence of a Neanderthal population*. «Quaternary International», 181, p. 64-71.

GARCÍA-CORTÉS, Á.; GALLEGO-VALCARCE, E.; BARETTINO-FRAILE, D. (eds.) (1999) — *Atlas del medio natural de la Región de Murcia*. Murcia, ITGE and Región de Murcia.

KEHL, M.; BUROW, C.; HILGERS, A.; NAVAZO, M.; PASTOORS, A.; WENIGER, G.-C.; WOOD, R.; JORDÁ PARDO, J. F. (2013) — *Late Neanderthals at Jarama VI (Central Iberia)?* «Quaternary Research», 80, p. 218-234.

LUCENA, A.; MARTÍNEZ, S.; ANGELUCCI, D. E.; BADAL, E.; VILLAVERDE, V.; ZAPATA. J.; ZILHÃO, J. (2012) — *La ocupación solutrense del abrigo de La Boja (Mula, Murcia, España)*. «Espacio, Tiempo y Forma. Serie I, Nueva época. Prehistoria y Arqueología», 5, p. 447-454.

MARTÍNEZ SÁNCHEZ, C. (1997) — *El yacimiento musteriense de Cueva Antón (Mula, Murcia)*. «Memorias de Arqueología de la Región de Murcia», 6, p. 18-47.

ROMÁN, D.; ZILHÃO, J.; MARTÍN-LERMA, I.; VILLAVERDE, V. (2013) — *La ocupación epimagdaleniense del abrigo de la Finca de Doña Martina (Mula, Murcia)*, in DE LA RASILLA, M. (ed.) — «F. Javier Fortea Pérez. *Universitatis Ovetensis Magister*. Estudios en homenaje», Universidad de Oviedo/Ménsula Ediciones, p. 167-178.

SALMERÓN, J.; LOMBA, J.; CANO, M. (1999) — *El arte rupestre paleolítico de Cieza: primeros hallazgos en la Región de Murcia. Resultados de la I campaña de prospecciones «Losares-Almadenes 93»*. «Memorias de Arqueología de la Región de Murcia», 8, p. 93-111.

WALKER, M. J.; LÓPEZ-MARTÍNEZ, M. V.; ORTEGA-RODRIGÁÑEZ, J.; HABER-URIARTE, M.; LÓPEZ-JIMÉNEZ, A.; AVILÉS-FERNÁNDEZ, A.; POLO-CAMACHO, J. L.; CAMPILLO-BOJ, M.; GARCÍA-TORRES, J.; CARRIÓN-GARCÍA, J. S.; SAN NICOLÁS-DEL TORO, M.; RODRÍGUEZ-ESTRELLA, T. (2012) — *The excavation of buried articulated Neanderthal skeletons at Sima de las Palomas (Murcia, SE Spain)*. «Quaternary International», 259, p. 7-21.

WALKER, M. J.; ANESIN, D.; ANGELUCCI, D. E.; AVILÉS-FERNÁNDEZ, A.; BERNA, F.; BUITRAGO-LÓPEZ, A. T.; FERNÁNDEZ-JALVO, Y.; HABER-URIARTE, M.; LÓPEZ-JIMÉNEZ, A.; LÓPEZ-MARTÍNEZ, M.; MARTÍN-LERMA, I.; ORTEGA-RODRIGÁÑEZ, J.; POLO-CAMACHO, J. L.; RHODES, S. E.; RICHTER, D.; RODRÍGUEZ-ESTRELLA, T.; SCHWENNINGER, J.-L.; SKINNER, A. R. (2016) — *Combustion at the late Early Pleistocene site of Cueva Negra del Estrecho del Río Quípar (Murcia, Spain)*. «Antiquity», 90, p. 571-589.

WOOD, R.; BARROSO-RUÍZ, C.; CAPARRÓS, M.; JORDÁ PARDO, J. F.; GALVÁN SANTOS, B.; HIGHAM, T. F. G. (2013) — *Radiocarbon dating casts doubt on the late chronology of the Middle to Upper Paleolithic transition in southern Iberia*. «Proceedings of the National Academy of Sciences USA», 110 (8), p. 2781-2786.

ZILHÃO, J. (1993) — *Le passage du Paléolithique moyen au Paléolithique supérieur dans le Portugal*, in CABRERA, V. (ed.) — «El Origen del Hombre Moderno en el Suroeste de Europa», Madrid, Universidad Nacional de Educación a Distancia, p. 127-145.

ZILHÃO, J. (2006) — *Chronostratigraphy of the Middle-to-Upper Paleolithic Transition in the Iberian Peninsula*. «Pyrenae», 37, p. 7-84.

ZILHÃO J.; PETTITT, P. (2006) — *On the New Dates for Gorham's Cave and the Late Survival of Iberian Neanderthals*. «Before Farming»: [online version], 2006/3, article 3; [print version], 2006/3, p. 95-122.

ZILHÃO, J.; VILLAVERDE, V. (2008) — *The Middle Paleolithic of Murcia*. «Treballs d'Arqueologia», 14, p. 229-248.

ZILHÃO, J.; ANGELUCCI, D.; BADAL, E.; LUCENA, A.; MARTÍN, I.; MARTÍNEZ, S.; VILLAVERDE, V.; ZAPATA, J. (2010a) — *Dos abrigos del Paleolítico superior en Rambla Perea (Mula, Murcia)*, in MANGADO, X. (ed.) — «El Paleolítico superior peninsular. Novedades del siglo XXI», Barcelona, Universidad de Barcelona, p. 97-108.

ZILHÃO, J.; ANGELUCCI, D.; BADAL-GARCÍA, E.; d'ERRICO, F.; DANIEL, F.; DAYET, L.; DOUKA, K.; HIGHAM, T. F. G.; MARTÍNEZ-SÁNCHEZ, M. J.; MONTES-BERNÁRDEZ, R.; MURCIA-MASCARÓS, S.; PÉREZ-SIRVENT, C.; ROLDÁN-GARCÍA, C.; VANHAEREN, M.; VILLAVERDE, V.; WOOD, R.; ZAPATA, J. (2010b) — *Symbolic Use of Marine Shells and Mineral Pigments by Iberian Neandertals*. «Proceedings of the National Academy of Sciences USA», 107 (3), p. 1023-1028.

ZILHÃO, J.; DAVIS, S. J. M.; DUARTE, C.; SOARES, A. M. M.; STEIER, P.; WILD, E. (2010c) — *Pego do Diabo (Loures, Portugal): Dating the Emergence of Anatomical Modernity in Westernmost Eurasia*. «PLoS ONE», 5 (1), e8880 (doi:10.1371/journal.pone.0008880).

ZILHÃO, J; AJAS, A.; BADAL, E.; BUROW, Ch.; KEHL, M.; LÓPEZ-SÁEZ, J. A.; PIMENTA, C.; PREECE, R. C.; SANCHIS, A.; SANZ, M.; WENIGER, G.-C.; WHITE, D.; WOOD, R.; ANGELUCCI, D. A.; VILLAVERDE, V.; ZAPATA, J. (2016) — *Cueva Antón: A multi-proxy MIS 3 to MIS 5a paleoenvironmental record for SE Iberia*. «Quaternary Science Reviews», 146, p. 251-273.

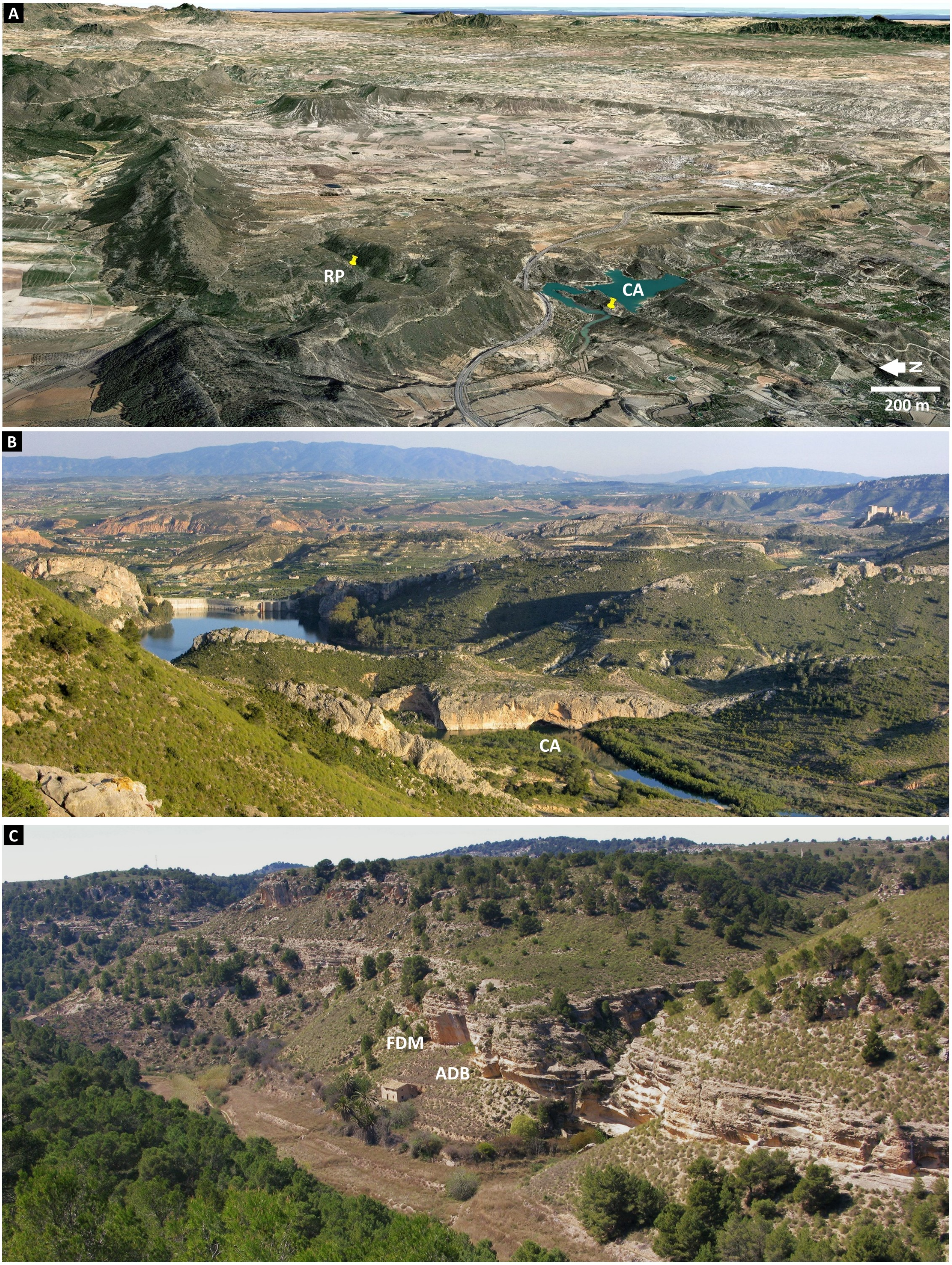

Fig. S1.1. Geographical setting of the Mula localities. A. GoogleEarth view of the Mula basin (March 29, 2015 image; elevation ×1.5) with location of Cueva Antón (CA), in the reservoir-inundated El Corcovado gorge, and of the Rambla Perea sites (RP). **B.** Panoramic photomosaic of the Mula valley upstream from the La Cierva dam (April 23, 2009). **C.** Position of the rock-shelters of Finca Doña Martina (FDM) and La Boja (ADB) in the middle section of the Rambla Perea, overviewed from downstream (March 20, 2008).

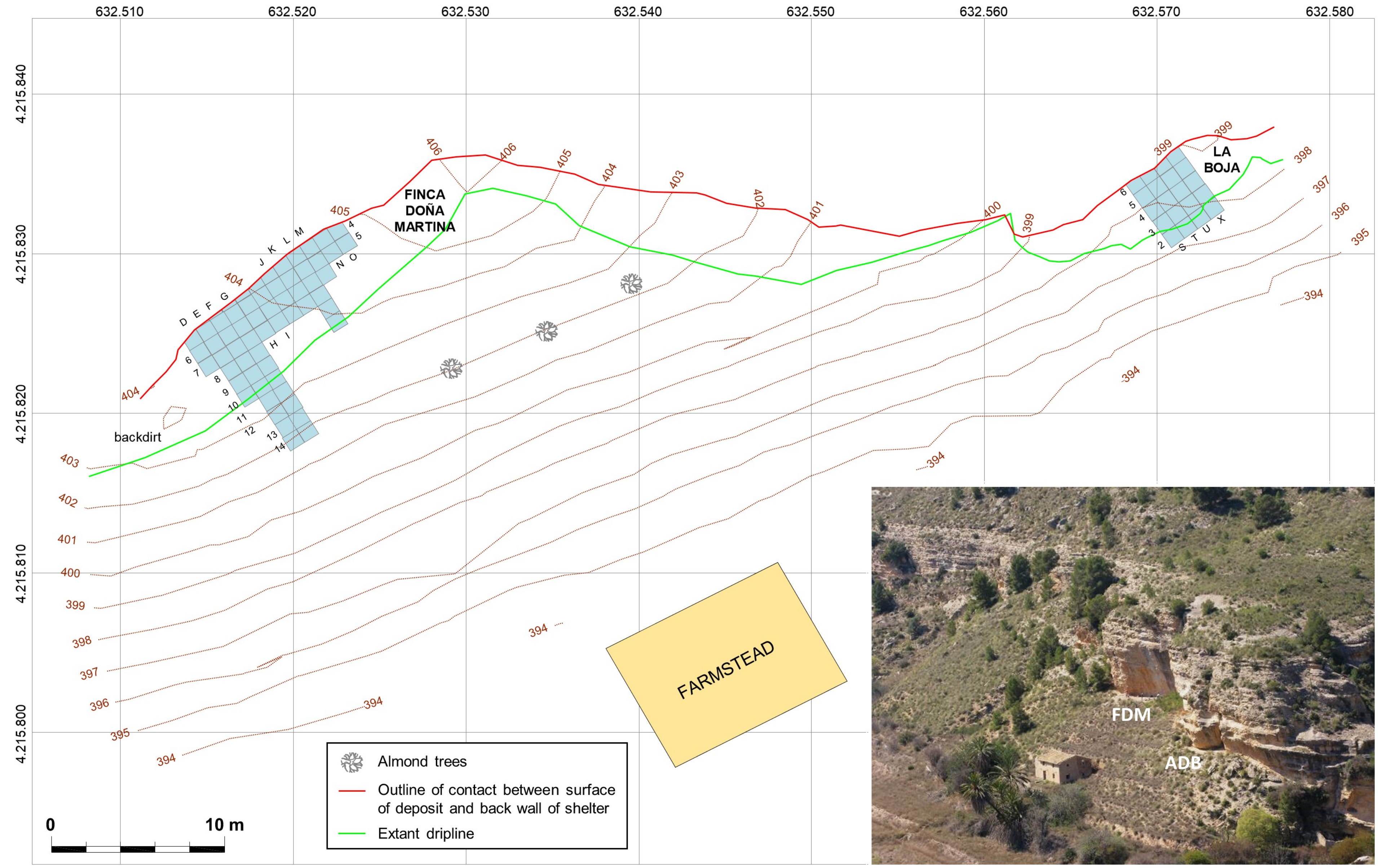

Fig. S1.2. The Rambla Perea rock-shelters: Finca Doña Martina (FDM) and Abrigo de La Boja (ADB). Surface topography with indication of the grid units excavated as of the end of the 2014 field seasons and overview of the two shelters taken from the SE, opposite side of the gorge, on March 23, 2008. Topographic survey and CAD (1:100 original) by Aerograph Studio. Coordinates are U.T.M. (ED50) and elevations are above sea level (Alicante datum).

Chapter 2.
The cave/rock-shelter of Cueva Antón

2.1. INTRODUCTION

Cueva Antón (38°03’52” N, 01°29’47” W) is located on the right bank of the River Mula, in the external side of the tight meander through which, prior to construction of the La Cierva dam, the stream entered the now submerged El Corcovado gorge (Fig. S1.1; Fig. S2.1). First investigated in 1991 in the context of a salvage operation (Martínez-Sánchez, 1997), the site was the target of extensive excavation between 2006 and 2012. This work established the stratigraphic, chronometric and paleoenvironmental background to human occupation (Zilhão et al., 2010, 2016; Angelucci et al., 2013; Burow et al., 2015) (Figs. S2.2-S2.10).

Detailed information on the methodology followed in the excavation and analysis of the site can be found in the published literature. Here, the focus lies on its archeological content, specifically the presentation and discussion of the late Middle Paleolithic context found in uppermost layer I-k. The evidence from the next archeologically fertile unit found as one moves down in the sequence, layer II-l, is also considered. Even though this unit significantly predates layer I-k, the comparison highlights the extent to which, regardless of change through time in settlement, subsistence, technology, or social organization, spatial constraints conditioned the site’s usefulness as a shelter for people, with attendant implications for the formation of an archeological record.

Following Zilhão et al. (2016), fluviatile sediments began to accumulate in Cueva Antón during the early or middle part of Marine Isotope Stage (MIS) 5. They hypothesize that the process would have been triggered by loss to erosion of the external wall of a large, preexisting karst fissure. Its interior having thus became exposed to penetration by the river, a fluviatile succession could accumulate inside and be subsequently preserved as a terrace-under-shelter deposit hanging a few meters above the present streambed. This river-accumulated deposit forms complex AS, which is sandwiched between basal, endokarst-accumulated palustrine sediments (complex FP) and the silts of variable thickness that, in recent times, during periods of long-term submersion by the La Cierva reservoir, blanketed the Pleistocene succession (complex DD). Due to its location inside a karst feature, the sediments of the AS complex also include variable amounts, increasing with proximity to the back wall, of material derived from the degradation of the surrounding bedrock.

A summary stratigraphic outline of the succession is provided in Table S2.1, and the reference stratigraphic cross-section is illustrated in Fig. S2.4. The lateral and vertical variation observed across the excavated area of the site can be appreciated from the additional cross-section and décapage information provided in Figs. S2.5-S2.9. A schematic 3D model of the accumulation process as reconstructed for the central part of the site is provided in Fig. S2.10.

Based on the erosional discontinuities identified, complex AS has been subdivided into five sub-complexes that can be succinctly described as follows:

AS5

Basal sandy beach deposit containing the remains of two stratigraphically well-separated episodes of human occupation appearing as dense, spatially extensive, lenticular scatters of stone tools, animal bone and wood charcoal structured around well-preserved hearth features — layers III-i/j and III-b/d.

AS4

Sterile remnant of an eroded sandy beach deposit accumulated after a sedimentation hiatus denoted by the paleosoil formation seen in layer II-u, which caps AS5.

AS3

Sterile sands, silts and clays for the most part accumulated in a boggy riverside context.

AS2

Gravel, sands and silts featuring, in layer II-l, a single episode of human occupation, represented by a low-density scatter of stone tools contained in river-accumulated sands whose trampling eventually erased the original cross-bedded structure.

AS1

Sterile, river-accumulated sequence of lenses of sand and silt, capped by a dense breccia — layer I-k (laterally continuing, against the back wall, as the 1991-defined units I-g and I-h) — made of small, angular, wall-degradation clasts packed in a sandy matrix and containing a low-density scatter of stone tools, animal bones and charcoal.

Coupled with the paleoenvironmental information derived from terrestrial mollusks, wood charcoal, and pollen, the OSL ages for the AS2-to-AS5 sequence suggest that layer II-l dates to the very end of MIS-5a, between 70 and 75 ka (Fig. S2.3). The chronology of sub-complex AS1 is based on the radiocarbon dating of wood charcoal samples processed with the rigorous ABOx-SC protocol. Consistency with stratigraphic order and the quality of the samples' chemistry warrant the reliability of these radiocarbon results, which place AS1 in the 35.1-37.7 ka interval (Table 1; Zilhão et al., 2016: Table 2). Combined, the OSL and radiocarbon dates imply a hiatus of some 35,000 years during which no sedimentation occurred at the site. In agreement with the geometry of the units making-up this part of the succession, the occurrence of such a hiatus is consistent with valley incision during MIS-4 and early MIS-3.

Along the E-W axis of the site, layer II-l was found across the area taken down to its depth (Fig. S2.11); sagittally, however, it wedged out along the slope of the embankment formed as the channel migrated out (to the inner side of the meander, at the end of MIS-5a) and downward (forming the Cueva Antón terrace, during MIS-4). Inside the cave, it was not until Greenland Interstadial (GI) 8 that the fluviatile dynamics resumed, as shown by the texture of the basal AS1 units (layers I-i, I-j, II-a, II-c and II-b). Unlike AS2, however, these units contain no cobble, gravel or coarse sand. They are entirely made up of fine sediments, which is consistent with valley incision and a decrease in the energy of the accumulation relative to MIS-5 times: even though occasionally inundated by overflowing waters, the cave would now be too high above the streambed for the deposition of channel-bottom material to be possible.

The basal AS1 alluvium (layers II-a and II-b) fills-up a rill that, outward, abuts the ridge of the talus created by post-AS2 valley incision (Figs. S2.7, S2.9-S2.10). This rill shows that erosional processes were active during the previous hiatus. Mostly, they would have consisted of run-off and limited overland flows, possibly originating in the large, upwardly oriented fissures and joints observed along the back wall, which may have functioned as temporary outlets draining the plateau above. All of this is especially apparent in the western part of the site, to which layers I-i, I-j and I-k did not extend. The primarily non-fluvial nature of layer I-k and its chronological proximity to underlying layer II-a mean that its delimitation can be taken as a proxy for the outline of the river margin through the time of accumulation (Fig. S2.10).

After GI 7, a new round of valley incision left the fill of Cueva Antón exposed as ground surface ever since. Coupled with the impact of repeated inundation since the construction of the La Cierva dam, this long-term exposure must have implied the loss to erosion of a significant thickness of the uppermost Pleistocene deposit, well apparent in the outline of the DD/I-k contact as seen in the J>I20-22 cross-section (Fig. S2.5). That such losses were limited can be put down principally to the coarse, densely packed, and carbonate-indurated nature of the unit.

The rim of large blocks and boulders accumulated in rows 18-19 of the grid that can be appreciated in Fig. S2.7 must also have played a role in protecting the AS1 deposit against erosion. The components of this rim all lie at the interface between layer I-k and the immediately underlying alluvium, suggesting that they stand for a single event of roof collapse, probably of seismic origin. Indeed, we know that multiple-ton boulders fell at the eastern end of the cavity before the beginning of the 2006-2012 work but after 1991 (they are not visible in photos taken during that year's excavation). Like the walls and roof above the inundation line, the old collapse is uniformly soot-stained because, in the 1930s, the cave sheltered a bread-baking oven. The recently collapsed material, however, exposes fresh, non-stained breaks.

The event responsible for this latest episode of roof fall is likely to have been the seism of magnitude 4.8 (Richter scale) that hit the region on February 2, 1999 (http://www.rinamed.net/es/casos/mula_terrem/mula_terrem.htm). This earthquake had a rather close to the surface hypocenter (1.1 km-deep), and an epicenter some 5 km north of the town of Mula, i.e., <2 km from Cueva Antón. The boulder rim skirting layer I-k could have been produced in similar manner.

No archeologically relevant burrow features were identified during the excavation of Cueva Antón. The large chamber well apparent in the east wall of the 1991 trench and whose inward continuation was clearly delimited in the 2011 excavation of the I20 "telephone booth" and adjacent squares (Fig. S2.5) is no exception. The mouth of that chamber was in the SE quadrant of grid unit H19 and cut through the upper part of AS2 down to layer II-i, without reaching layers II-k and II-l. Having affected neither layer I-k nor layer II-l, this feature is therefore of no consequence for the assessment of the stratigraphic integrity of the archeology buried in the units of concern here.

2.2. THE ARCHEOLOGY OF LAYER II-l

Excluding the ubiquitous rabbits, which, through the entire Cueva Antón sequence, are accumulated by the eagle-owl and, hence, part of the natural background, the faunal remains retrieved in layer II-l total less than 30 specimens. Of these, only a handful could be taxonomically assigned — to *Equus* sp. and *Cervus* sp. (Zilhão et al., 2016: Table 5). Surface conditions range from the slight polish and smoothing apparent in the equid tooth illustrated in Fig. S2.12 to some significant rounding, in a few cases entailing loss of surface morphology. These features suggest an alluvial origin. However, the fact that the deposit is affected by trampling confirms exposure as ground surface for some length of time and is consistent with humans having occupied the place once the accumulation of sediments had ceased. Much the same is implied by the condition of the associated stone tools (Figs. S2.13-S2.15). Even though patinated to varying degrees, they display fresh edges, indicating that they were discarded in situ, not transported — as otherwise implicated by the refitting evidence.

Together, this information is suggestive of the sporadic human occupation of a narrow stretch of frequently inundated riverside terrain. Of the 35 m² area where the excavation went down to or beyond the elevation of layer II-l, the latter only existed over some 29 m². Its total extent probably is not much more than that because (a) layer II-m can be observed outcropping directly under the DD deposit west of column Q of the grid, while (b) the inclination of the bedrock (Fig. S2.2) implies that layer II-l is unlikely to be present east of column G. We can therefore reconstruct the area available for settlement as a ca.50 m² crescent-shaped beach tucked under the cave's roof and isolated from the surrounding slopes — except when lowered river levels left a dryland path between the water and the vertical escarpment extending upriver of the cave opening (Fig. S2.1).

No archeology is reported from the 1991 excavation of layer II-l. However, the grid units excavated in 2006-2012 that are adjacent to that year's trench yielded stone tool finds of substantial size (e.g., the K19 core and the J19 sidescraper of Fig. S2.14, nos. 1-2). Therefore, the 1991 pattern is more likely to reflect expedient excavation in a salvage context than genuine absence and, when computing the density of the artefacts' distribution (Tables S2.2-S2.3), the 9 m² then excavated ought to be excluded from consideration. Even so, the value is very low: 44 items over some 20 m², i.e., 2.2/m².

Except for the immediately available limestone component (a tested cobble, refitted flake blanks, and one retouched tool; Fig. S2.15), the lithic assemblage of layer II-l is mostly imported. Only 18% are knapping debris and, bearing in mind the recurrent inundation of the place, the six items of flint chippage in II-l may well be, like the large herbivore bones and teeth, a transported, natural component of the deposit: all are very small, ranging in mass between 0.02 and 0.36 g, and two are indeed rolled. On the other hand, the K19-14 (Fig. S2.14, no. 1) centripetal/discoid core probably represents a lost object because the flint variety it is made of is not represented among the blanks. This volume therefore appears not to have been reduced at the site — unless the activity took place inside the area of the 1991 trench, and the corresponding products, byproducts and debris went unnoticed at the time.

The only secure instance of on-site flint knapping in layer II-l is that documented by item P20-7, a sidescraper re-sharpening flake (Fig. S2.13). Another small flake of identical size and made on the same raw-material (N20-5), retrieved nearby, may have been produced during the same knapping event. The re-sharpened tool, however, was not found. Likely, it was taken away by its owner upon leaving the site.

The large, distolaterally broken J19-18 sidescraper is the only formal tool of the assemblage that features a break (Fig. S2.14, no. 2). The missing part was not found, so it cannot be ascertained whether the object fractured on- or off-site. Subsequent on-site use, or re-use, is nevertheless indicated by macroscopic wear showing that the active part of the tool (represented by the sidescraper-retouched edge) extended to and along the break.

The remaining finds are either unretouched flakes and flake fragments (seven) or sidescrapers (five). We cannot exclude that these items reflect loss or discard with no on-site use, as seems to be the case with the K19-14 core. The two documented instances of on-site re-sharpening and/or re-use suggest, however, that this is unlikely. Even though the lithics have yet to be analyzed for microscopic wear, these data imply that tasks involving the use of stone tools were carried out in the context of the layer II-l occupation(s), no matter how sporadic and transient they must have been. Given the apparent absence of anthropogenic faunal remains, such tasks may well have been limited to repair and replacement — of clothing, hunting equipment and/or travel gear.

Even if no supporting dating evidence had been available, there could be no doubt that, from both the technological and the typological standpoints, this assemblage, small as it is, belongs fully in the Middle Paleolithic. In the absence of refits or characteristic byproducts, abandoned cores such as K19-14 can represent the endpoint of either discoid or Levallois recurrent-centripetal reductions. Both are diagnostically Middle Paleolithic blank production methods. Moreover, in Western Europe, no assemblages of similar size consisting entirely of sidescrapers, notches and unretouched flake blanks have ever been found within Upper Paleolithic stratigraphic sequences, or radiometrically dated to that period. In short, (a) the small size of the layer II-l assemblage must relate to the constraints on human use imposed by the site's then extant spatial configuration, and (b) such a small size is no impediment for the unambiguous manifestation of the technologically diagnostic concepts underpinning raw-material economy and blank production.

2.3. THE ARCHEOLOGY OF LAYER I-k

Excluding the bones of birds, micromammals and rabbits accumulated by the eagle-owl, layer I-k yielded 34 faunal remains. Only a cervid, probably *Cervus elaphus*, could be determined. One long bone splinter belongs to a larger herbivore, probably an equid (Zilhão et al., 2016: Table 5). The edges and the surfaces of the bones are fresh, but none is burnt and no cut-marks have been identified. Therefore, this small assemblage may well be unrelated to the human occupation, and reflect instead episodic use of the site by carnivores. That most of the remains come from the eastern part of the layer's excavated area, which yielded no artefacts, supports this inference (Fig. S2.16).

Of the ca.54 m² excavated in the Z-L columns of the grid, layer I-k existed in some 36 m². To the West, the boundaries observed in plan as much as in cross-section views suggest that the layer extended into the area of the 1991 trench only marginally. It is therefore entirely plausible that the lack of finds from I-k among that year's faunal and stone tool collections stands for a genuine absence. However, the distribution of refitted flint items links grid units J19 and K19, excavated in 2008, with I20, excavated in 2011. This connection suggests that products and/or byproducts of the reduction of the same block could have been present, even though missed, in the intermediate grid unit J20, excavated in 1991 (Figs. S2.16-S2.18).

Given these data, the density values one can derive from the stone tool counts in Table S2.4 are of the same order of magnitude as those for layer II-l: 0.9/m² for the faunal remains, 0.6/m² for the lithics. Excluding the 1991 trench and postulating that the grid units east of column D, which yielded no stone tools, lied outside of the occupied area, the total surface decreases to 25 m². Using this denominator brings the density up, but not significantly: to 1.4/m² and 0.8/m² for fauna and lithics, respectively. Such low numbers mean that the finds made in layer I-k could well stand for a few sporadic, pass-through occupations, if not a single such episode of site use — one whose remains were then syn-depositionallly scattered along the E-W dip of the stratification by run-off and gravity.

This evidence further suggests that, like the fauna, the abundant but quite dispersed wood charcoal found in layer I-k may well be largely non-anthropogenic. There can be no question, however, that its stratigraphic association with the layer's stone tools is reliable. Indeed, of the 90 pieces of charcoal that underwent anthracological examination, all those that could be classified belong to Pleistocene taxa (*Juniperus* sp. and *Pinus sylvestris/nigra*) (Zilhão et al., 2016: SI Table 2). The absence of such Holocene taxa as *Olea* sp. or *Pinus halepensis* is matched by the results obtained with the radiocarbon dating of samples selected from the examined assemblage: of the 11 that were ABOx-processed, seven failed due to low yields, and the four successful ones yielded Pleistocene results consistent with expectations derived from the characteristics of the associated stone tools. The absence of intrusive charcoal can also be explained by the truncation undergone by layer I-k — its uppermost reaches, those that, through long-term exposure as ground floor, would have been affected by trampling and reworking, must have been eroded away (most recently because of river damming and the attendant submersion of the site).

Given its scarcity in archeological remains, layer I-k was excavated as a single field unit, with no internal spit subdivision. Compared with the values recorded for the surface and base of the layer, the finds' elevation data, however, indicate that most if not all come from its lower reaches, if not the very bottom. In grid unit I20, for instance, the elevation of two of the items included in this layer's refit (I20-6 and I20-7; Fig. S2.18) could be precisely noted: 117 cm below datum. This is 1 cm above the base of the unit at the center of the NW quadrant where those items came from, and 5-10 cm below the surface of the unit as estimated from the values for surrounding points. Likely, therefore, the erosional truncation of the upper part of layer I-k entailed no significant loss of this unit's original archeological content.

One of the ABOx-dated samples from layer I-k also came from grid unit I20. It was retrieved in the NE quadrant and at an elevation of 115 cm below datum, closer to the surface of the deposit and above the flint material. In contrast, the other sample came from the very base of layer I-k in grid unit G21, 2.3 m away (Fig. S2.16). Since there is no reason to suppose that the charcoal scatter originates in a single depositional event, or that it must be of one and the same age, this spatial information carries two implications: firstly, that the result obtained for the G21 sample best reflects the time of occupation; secondly, that an upper boundary for such a time is provided by the *terminus ante quem* represented by the result for the I20 sample. Be it as it may, both results are consistent with the *terminus post quem* represented by the result obtained for a sample (E21-11) from immediately underlying layer II-a. Given these stratigraphic constraints and the samples' ages (Table 1), the artefacts contained in layer I-k must have been used and/or discarded at the site no earlier than 37.1 ka.

This late date has led some to question the homogeneity and definition of layer I-k (Wood et al., 2013). However, the refitting evidence (Fig. S2.18A-C) concurs with the absence of intrusive charcoal in supporting the layer's stratigraphic integrity, and no Upper Paleolithic stone tool types are present in the lithic assemblage, in which diagnostically Upper Paleolithic blank production methods are not represented either. No indication exists, therefore, that we might be dealing with a palimpsest containing both Middle and Upper Paleolithic material and that the dated charcoal might relate to the latter instead of the former.

The Middle Paleolithic affinities of the stone tool assemblage are, in turn, undeniable: each one of those methods that are documented would suffice, in and of itself, to support assignment to the Middle Paleolithic, and the more so since they co-occur. Such methods are:

- Centripetal (Discoid or Levallois), represented by the J19-4 core and its products and byproducts, most of which could be refitted back (Fig. S2.17, no. 1; Fig. S2.18A-C);
- Discoid, represented by the blank for the D20-2 notched piece, which is made on a recycled, discoid core-trimming byproduct (Fig. S2.17, no. 5);
- Levallois, represented by the H21-8 laminar flake (Fig. S2.18D-E, Fig. 19, no. 1);
- Kombewa, represented by the F18-1 core (Fig. S2.17, no. 4);
- the production of naturally backed, orange segment-like flakes, probably deliberately overshot discoid blanks, represented by the E19-7 denticulate (Fig. S2.17, no. 3); for flint, this method is unknown in the regional Upper Paleolithic but well documented in its Mousterian (see Chapter 3).

The other core is a "splintered piece" documenting the use of the bipolar technique (Fig. S2.17, no. 2), also represented by the use-worn, unretouched blank F19-1 (Fig. S2.19, no. 2). Bipolar reduction can also be found in the regional Upper Paleolithic (see Chapter 4) but, given its ubiquity across the culture-stratigraphic sequence of Southwestern Europe's Upper Pleistocene (Aubry et al., 1997), it remains entirely consistent with the Middle Paleolithic affinities of everything else.

In a context of such short, sporadic visits to the place (by small groups and/or isolated individuals) as those recorded in layer I-k, one would expect the stone tools used at the site (as opposed to simply therein lost or discarded) to have been mostly involved in repairing and re-tooling tasks involving the processing of wood. The macro- or microscopic use-wear evidence is consistent with this notion (Table S2.5). Contact with bone or antler is documented in addition to wood in the case of the H21-8 Levallois blank (Fig. S2.19, no. 1). However, given that the layer's animal bone is likely to be non-anthropogenic, that wear may well represent prior, off-site use of one of this imported object's cutting edges.

As with layer II-l, the small size of the layer I-k assemblage must relate to the limited accessibility of the site and its general unsuitability to serve as a shelter for a significant number of people, or a significant length of time — the more so for layer I-k, as it formed after valley incision had left the cave hanging above the streambed at the base of a 20 m-high, vertical cliff-face. We could reconstruct layer II-l as a crescent-shaped 50 m^2 beach tucked under the cave's roof that, most of the time, would have had no dryland access. From Figs. S2.10 and S2.16 we can reconstruct layer I-k as a narrow, sloping tongue of angular limestone gravel extending across a similar surface — a band of terrain no more than 5 m-wide running along the back wall of the cave, access to which would have been subject to the same constraints as during layer II-l times. No wonder, therefore, that, functionally, despite being 35,000 years apart, the two occupations are strikingly similar.

Such a similarity is also apparent in the realm of raw-material economy, as the I-k refit unit matches the II-l episode of limestone knapping in documenting the association of imported items with the exploitation of locally available stuff. The flint variety represented in the I-k refit has primary sources a few hundred meters away, in the valley's Cretaceous limestone bedrock, where it occurs as narrow, tectonized bands featuring much cleavage. Such structure is well apparent in the block that was reduced at Cueva Antón, and is one that renders this raw-material unsuitable to produce large blanks using complex reduction methods. These characteristics explain well why the knapping episode documented in layer I-k apparently produced a single usable piece (the flake extracted at the end of the sequence), which we could not find and would seem to have been exported.

The above has implications for the interpretation of the K19-3 perforated, ochre-stained half-valve of *Pecten maximus* (Fig. S2.20) found in close spatial association with the J19-4 core and some of its products and byproducts (Fig. S2.16). Ochre may have utilitarian functions, namely in the preparation of hides, but no wear related to such functions has been identified in the lithics from layer I-k, and none is ochre-stained. This evidence rejects interpretations of the shell's pigmentation as accidental, i.e., as resulting from post-depositional accumulation of iron oxides brought in for hide-processing tasks or locally produced by diagenetic processes. The additional evidence acquired since publication of the shell's age, association, dating and symbolic significance (Zilhão et al., 2010) therefore supports the original argument: the parsimonious interpretation of the Cueva Antón *Pecten* remains that it entered the site as a painted/perforated item of body decoration therein discarded after breaking.

2.4. CONCLUSION

At the site, the layer I-k assemblage is by no means unique. Its characteristics relate to the exceptionally high resolution of the Cueva Antón archeological record, which enabled the preservation as separate stratigraphic units of contexts that either result from single, sporadic occupation events leaving a limited number of remains, or stand for the accumulated result of a small number of such events occurring within a very short time span. In contrast, the site formation frameworks most commonly encountered when dealing with cave and rock-shelter sites are characterized by lower sedimentation rates, entailing the formation of stratigraphic sequences of lesser resolution. In such "normal" frameworks, substantial assemblages can build-up even if each of the individual occupation events subsumed therein is of the same kind as those recorded in layers I-k and II-l of Cueva Antón. The reason is simple: in such frameworks, the time span represented by the accumulation is much longer.

At times of cultural or technological transition, low sedimentation rates may end up in the formation of stratigraphic units containing assemblages that feature the different terms, or steps, of such a transition. In that situation, disentangling the different components and the meaning of their co-occurrence can only be made against an external frame of reference. Put another way, when sedimentation is slow, establishing whether the co-occurrence of different technological systems stands for their true integration, for their true coexistence, or for a palimpsest effect can only be done post-hoc, against previously acquired knowledge. At Cueva Antón, rapid build-up is documented by the radiocarbon dating of sub-complex AS1 and the sedimentological characteristics of last interglacial sub-complexes AS2 to AS5 (Zilhão et al., 2016). In such a context, it is entirely to be expected that, large or small, the artefact assemblages retrieved from individual stratigraphic units will reflect use and discard within a restricted time window and, hence, that, in culture-stratigraphic terms, their content will be homogeneous.

Thus, questioning the Middle Paleolithic nature of layer I-k would be no more warranted than doing it for layer II-l, which yielded an assemblage of similar size and bearing the same kinds of technological diagnostics. When the two assemblages are compared, the only difference resides in that the retouched tools made on flint are all sidescrapers in II-l and all notches, denticulates, or use-worn, unretouched blanks in I-k. Given the small size of the assemblages, the significance of this difference, which may well be of a functional nature, is difficult to assess.

In this respect, it is nevertheless worth noting that, elsewhere in Iberia, the few secure, very late Middle Paleolithic contexts known also feature very low percentages of retouched tools. Those that we do find in such contexts are, as in layer I-k of Cueva Antón, only or mostly notches and denticulates. Layer 8 of Gruta da Oliveira (Almonda karst system, Torres Novas, Portugal), placed by radiocarbon in the 35.6-38.6 ka interval, is a case in point. Here, the age of the assemblage is independently supported by U-series results for the same layer (Hoffmann et al., 2013) and, among a total of 84 blanks greater than 2.5 cm, only five — all of which are discontinuously notched/denticulated pieces — were retouched (Marks et al., 2001).

A similar pattern is apparent at the open-air site of Foz do Enxarrique (Vila Velha de Ródão, Portugal), where the archeological context is buried in rapidly accumulated inundation silts belonging to the 5-10 m alluvial terrace of the Tagus, dated by OSL to the 31-40 ka interval (Cunha et al., 2008, 2012). Nearly 10,000 artefacts were recovered at this site, where, per Brugal and Raposo (1999: 369-370), "the industry is characterized by numerous discoid and Levallois recurrent centripetal cores" and "retouched tools are dominated by notches and denticulates, sidescrapers are rare and tools of 'Upper Paleolithic' type are virtually absent."

Layer I-k therefore does not "walk alone." Dating error and/or problems of assemblage definition have been shown to underpin the attribution of some Iberian occurrences to a late-persisting Middle Paleolithic (Zilhão, 2006; Wood et al., 2013; Kehl et al., 2013). However, the high resolution of the Cueva Antón stratigraphic sequence and the quality and accuracy of the radiocarbon dating of sub-complex AS1 and, within it, layer I-k, confirm that, at least in Southeast Spain, the Middle Paleolithic did not disappear — and, hence, neither did its Neandertal makers — until after some 37,100 years ago.

2.5. REFERENCES

ANGELUCCI, D.; ANESIN, D.; SUSINI, D.; VILLAVERDE, V.; ZAPATA, J.; ZILHÃO, J. (2013) — *Formation processes at a high resolution Middle Paleolithic site: Cueva Antón (Murcia, Spain)*. «Quaternary International», 315, p. 24-41.

AUBRY, Th.; ZILHÃO, J.; ALMEIDA, F.; FONTUGNE, M. (1997) — *Production d'armatures microlithiques pendant le Paléolithique supérieur et le Mésolithique du Portugal*, in BALBÍN, R.; BUENO, P. (eds.) — «II Congreso de Arqueología Peninsular. Paleolítico y Epipaleolítico», Zamora, Fundación Rei Afonso Henriques, p. 259-272.

BRUGAL, J. P.; RAPOSO, L. (1999) — *Foz do Enxarrique (Ródão, Portugal): first results of the analysis of a bone assemblage from a Middle Paleolithic open site*, in «The Role of Early Humans in the Accumulation of European Lower and Middle Paleolithic Bone Assemblages», Mainz, Monographien des Römisch-Germanischen Zentralmuseums 42, p. 367-379.

BUROW, C.; KEHL, M.; HILGERS, A.; WENIGER, G.-C.; ANGELUCCI, D. E.; VILLAVERDE, V.; ZAPATA, J.; ZILHÃO, J. (2015) — *Luminescence Dating of Fluvial Deposits in the Rock Shelter of Cueva Antón, Spain*. «Geochronometria», 42, p. 107-125.

CUNHA, P. P.; MARTINS, A. A.; MURRAY, A. S.; HUOT, S.; MURRAY, A.; RAPOSO, L. (2008) — *Dating the Tejo river lower terraces in the Ródão area (Portugal) to assess the role of tectonics and uplift*. « Geomorphology», 102, p. 43-54.

CUNHA, P. P.; ALMEIDA, N. A. C.; AUBRY, Th; MARTINS, A. A.; MURRAY, A. S.; BUYLAERT, J.-P.; SOHBATI, R.; RAPOSO, L.; ROCHA, L. (2012) — *Records of human occupation from Pleistocene river terrace and aeolian sediments in the Arneiro depression (Lower Tejo River, central eastern Portugal)*. « Geomorphology», 165-166, p. 78-90.

HOFFMANN, D. L.; PIKE, A. W. G.; WAINER, K.; ZILHÃO, J. (2013) — *New U-series results for the speleogenesis and the Paleolithic archeology of the Almonda karstic system (Torres Novas, Portugal)*. «Quaternary International», 294, p. 168-182.

KEHL, M.; BUROW, C.; HILGERS, A.; NAVAZO, M.; PASTOORS, A.; WENIGER, G.-C.; WOOD, R.; JORDÁ PARDO, J. F. (2013) — *Late Neanderthals at Jarama VI (Central Iberia)?* «Quaternary Research», 80, p. 218-234.

MARKS, A.; MONIGAL, K.; ZILHÃO, J. (2001) — *The lithic assemblages of the Late Mousterian at Gruta da Oliveira, Almonda, Portugal*, in ZILHÃO, J.; AUBRY, Th.; CARVALHO, A. F. (eds.) — «Les premiers hommes modernes de la Péninsule Ibérique. Actes du Colloque de la Comission VIII de l'UISPP, Vila Nova de Foz Côa, Octobre 1998», Trabalhos de Arqueologia 17, Lisboa, Instituto Português de Arqueologia, p. 145-154.

MARTÍNEZ SÁNCHEZ, C. (1997) — *El yacimiento musteriense de Cueva Antón (Mula, Murcia)*. «Memorias de Arqueología de la Región de Murcia», 6, p. 18-47.

RASMUSSEN, S. O.; BIGLER, M.; BLOCKLEY, S. P.; BLUNIER, TH.; BUCHARDT, S. L.; CLAUSEN, H. B.; CVIJANOVIC, I.; DAHL-JENSEN, D.; JOHNSEN, S. J.; FISCHER, H.; GKINIS, V.; GUILLEVIC, M.; HOEK, W. Z.; LOWE, J. J.; PEDRO, J. B.; POPP, T.; SEIERSTAD, I. K.; STEFFENSEN, J. P.; SVENSSON, A. M.; VALLELONGA, P.; VINTHER, B. M.; WALKER, M. J. C.; WHEATLEY, J. J.; WINSTRUP, M. (2014) — *A stratigraphic framework for abrupt climatic changes during the Last Glacial period based on three synchronized Greenland ice-core records: refining and extending the INTIMATE event stratigraphy*. «Quaternary Science Reviews», 106, p. 14-28.

REIMER, P. J.; BARD, E.; BAYLISS, A.; BECK, J. W.; BLACKWELL, P. G.; BRONK RAMSEY, C.; BUCK, C. E.; CHENG, H.; EDWARDS, R. L.; FRIEDRICH, M.; GROOTES, P. M.; GUILDERSON, T. P.; HAFLIDASON, H.; HAJDAS, I.; HATTÉ, C.; HEATON, T.J.; HOFFMANN, D. L.; HOGG, A. G.; HUGHEN, K. A.; KAISER, K.F.; KROMER, B.; MANNING, S. W.; NIU, M.; REIMER, R. W.; RICHARDS, D. A.; SCOTT, E. M.; SOUTHON, J. R.; STAFF, R. A.; TURNEY, C. S. M.; VAN DER PLICHT, J. (2013) — *IntCal13 and MARINE13 radiocarbon age calibration curves 0-50000 years calBP*. «Radiocarbon», 55 (4), p. 1869-1887.

SÁNCHEZ-GOÑI, M. F.; BARD, E.; LANDAIS, A.; ROSSIGNOL, L.; d'ERRICO, F. (2013) — *Air-sea temperature decoupling in western Europe during the last interglacial-glacial transition*. «Nature Geoscience», 6, p. 837-841.

SÁNCHEZ-GOÑI, M. F.; LANDAIS, A.; FLETCHER, W. J.; NAUGHTON, F.; DESPRAT, S.; DUPRAT, J. (2008) — *Contrasting impacts of Dansgaard-Oeschger events over a western European latitudinal transect modulated by orbital parameters*. «Quaternary Science Reviews», 27, p. 1136-1151.

SÁNCHEZ-GOÑI, M. F.; BARD, E.; LANDAIS, A.; ROSSIGNOL, L.; d'ERRICO, F. (2013) — *Air-sea temperature decoupling in western Europe during the last interglacial-glacial transition*. «Nature Geoscience», 6, p. 837-841.

STUIVER, M.; REIMER, P. J. (1993) — *Extended ^{14}C Data Base and Revised CALIB 3.0 ^{14}C Age Calibration Program*. «Radiocarbon», 35 (1), p. 215-230.

WOOD, R.; BARROSO-RUÍZ, C.; CAPARRÓS, M.; JORDÁ PARDO, J. F.; GALVÁN SANTOS, B.; HIGHAM, T. F. G. (2013) — *Radiocarbon dating casts doubt on the late chronology of the Middle to Upper Paleolithic transition in southern Iberia*. «Proceedings of the National Academy of Sciences USA», 110 (8), p. 2781-2786.

ZILHÃO, J. (2006) — *Chronostratigraphy of the Middle-to-Upper Paleolithic Transition in the Iberian Peninsula*. «Pyrenae», 37, p. 7-84.

ZILHÃO, J.; VILLAVERDE, V. (2008) — *The Middle Paleolithic of Murcia*. «Treballs d'Arqueologia», 14, p. 229-248.

ZILHÃO, J.; ANGELUCCI, D.; BADAL-GARCÍA, E.; d'ERRICO, F.; DANIEL, F.; DAYET, L.; DOUKA, K.; HIGHAM, T. F. G.; MARTÍNEZ-SÁNCHEZ, M. J.; MONTES-BERNÁRDEZ, R.; MURCIA-MASCARÓS, S.; PÉREZ-SIRVENT, C.; ROLDÁN-GARCÍA, C.; VANHAEREN, M.; VILLAVERDE, V.; WOOD, R.; ZAPATA, J. (2010) — *Symbolic Use of Marine Shells and Mineral Pigments by Iberian Neandertals*. «Proceedings of the National Academy of Sciences USA», 107 (3), p. 1023-1028.

ZILHÃO, J; AJAS, A.; BADAL, E.; BUROW, Ch.; KEHL, M.; LÓPEZ-SÁEZ, J. A.; PIMENTA, C.; PREECE, R. C.; SANCHIS, A.; SANZ, M.; WENIGER, G.-C.; WHITE, D.; WOOD, R.; ANGELUCCI, D. A.; VILLAVERDE, V.; ZAPATA, J. (2016) — *Cueva Antón: A multi-proxy MIS 3 to MIS 5a paleoenvironmental record for SE Iberia*. «Quaternary Science Reviews», 146, p. 251-273.

Table S2.1. Cueva Antón stratigraphy. Depositional environments recorded in the succession. The solid lines indicate major erosive surfaces, the broken lines indicate minor discontinuities (after Angelucci et al., 2013 and Zilhão et al., 2016)

Complex	Unit(s)	Depositional environment
DD		twentieth century artificial reservoir
TL		exposed surface
AS1	I-g, I-h, I-k	alluvial (floodplain plus bar/levee intercalation and one lacustrine event) alternating to (and ending with) wall degradation and runoff
	I-i	
	I-j, II-a	
	II-c	
	II-b	
AS2	II-d, II-e	alluvial bar/levee alternating to wall degradation and runoff
	II-f	
	II-g	fining upward alluvial sequence (channel, bar and floodplain), with intercalated events of wall degradation
	II-h, II-i	
	II-k	
	II-l top	
	II-l, II-m	
AS3	II- ñ, II-z, II-o	fining upward alluvial sequence (bar and floodplain) capped by lacustrine event
	II-p	wall degradation followed by alluvial floodplain
	II-q	alluvial sequence (channel, bar and floodplain) followed by wall degradation
	II-s	
	II-t	
AS4	II-ø	alluvial bar/levee
AS5	II-u	alluvial bar/levee with events of wall degradation and slope outwash
	II-w	
	II-t, II-y	
	III-a	
	III-b, III-c	alluvial bar/levee
	III-d	alluvial bar
	III-e, III-f, III-g	
	III-i	wall degradation and alluvial bar
	III-j	
	III-k	
	III-l	
	III-m, III-n	alluvial bar
FP	IV	'lacustrine'

Table S2.2. Cueva Antón layer II-l. Stone tool technological categories. Two small flint nodules (possibly manuports, or else, given the fluvial nature of the accumulation, part of the natural geological background) are not included. N = number, M = mass in grams

RAW-MATERIAL	CORES		FLAKE BLANKS						LAMINARY BLANKS				DEBRIS				TOOLS		TOTAL	
			Complete		Fragment		Small		Blade		Bladelet		Chippage		Chunk					
	N	M	N	M	N	M	N	M	N	M	N	M	N	M	N	M	N	M	N	M
**Flint**	1	49.3	3	17.7	4	6.6	2*	1.5	–	–	–	–	6	1.4	–	–	6	102.3	22	178.9
**Quartzite**	–	–	–	–	–	–	–	–	–	–	–	–	–	–	–	–	–	–	–	–
**Limestone**	1	148.0	2	17.2	6	37.9	–	–	–	–	–	–	2	1.0	–	–	1	17.3	12	221.4
**Quartz**	–	–	–	–	–	–	–	–	–	–	–	–	–	–	–	–	–	–	–	–
**TOTAL**	2	197.3	5	34.9	10	44.5	2	1.5	–	–	–	–	8	2.4	–	–	7	119.6	34	400.3

*The two small flint flakes are by-products of a single sidescraper re-sharpening event

Table S2.3. Cueva Antón layer II-l. Classification of cores and retouched tools. Includes only formal, retouch-modified items

Cores	N
chopper	1
centripetal (Levallois or discoid)	1
**TOTAL**	2

Formal retouched tools	N
notched piece	1
sidescraper	
simple	4
transversal	1
convergent	1
**TOTAL**	**7**

Table S2.4. Cueva Antón layer I-k. Stone tool technological categories. Two small, unmodified limestone cobbles (possibly manuports, or else, given the fluvial nature of the accumulation, part of the natural geological background) are not included. N = number, M = mass in grams

RAW-MATERIAL	CORES (a)		FLAKE BLANKS						LAMINARY BLANKS				DEBRIS				TOOLS (b)		TOTAL	
			Complete		Fragment		Small		Blade		Bladelet		Chippage		Chunk					
	N	M	N	M	N	M	N	M	N	M	N	M	N	M	N	M	N	M	N	M
**Flint**	3	110.3	3	21.8	4	5.9	2	3.7	–	–	–	–	5	0.7	1	3.3	2	21.3	20	167.0
**Quartzite**	–	–	–	–	–	–	–	–	–	–	–	–	–	–	–	–	–	–	–	–
**Limestone**	–	–	–	–	–	–	–	–	–	–	–	–	–	–	–	–	–	–	–	–
**Quartz**	–	–	–	–	–	–	–	–	–	–	–	–	–	–	–	–	–	–	–	–
**TOTAL**	3	110.3	3	21.8	4	5.9	2	3.7	–	–	–	–	5	0.7	1	3.3	2	21.3	20	167.0

(a) 1 Kombewa, 1 discoid and 1 bipolar
(b) 1 notched piece and 1 denticulate

Table S2.5. Cueva Antón layer I-k. Use-wear evidence (flint).
Material used on, inferred function, and traces of residue present

	Illegible	None	Wood	Hide	Meat	Bone	Projectile	Ochred	Total
notches	–	–	1	–	–	–	–	–	**1**
denticulates	–	–	1	–	–	–	–	–	**1**
unmodified blank	–	–	2	–	–	1	–	–	**3**
**TOTAL**	–	–	4	–	–	1	–	–	**5**

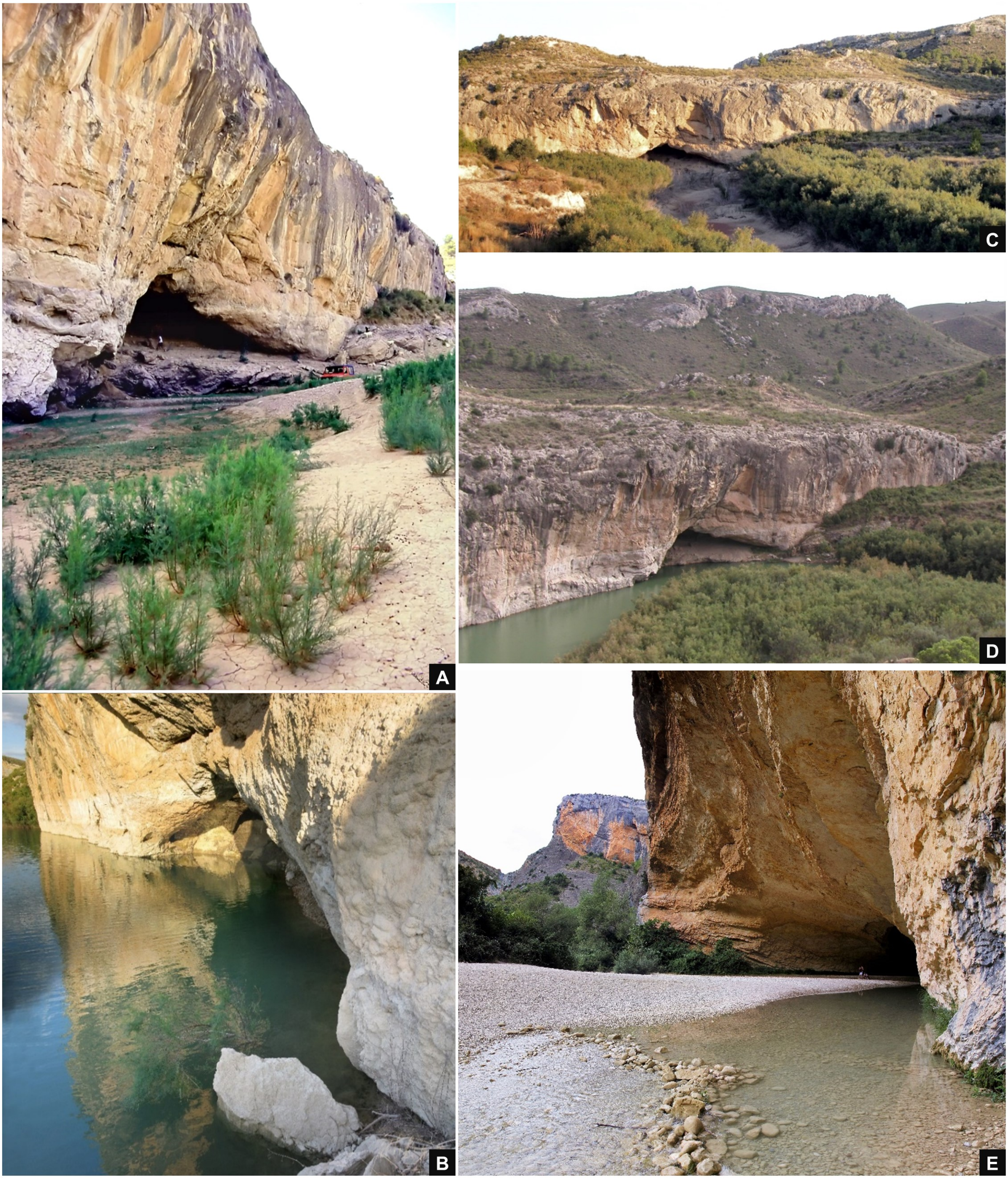

Fig. S2.1. Cueva Antón. Site setting. A. The mouth of the cave seen from the NE at the time of the 1991 salvage work (photo: C. Martínez-Sánchez). **B.** The mouth of the cave on September 19, 2009; the high water level prevented access to the site by land until the end of the following summer. **C.** The cave seen from the NW on September 26, 2011, when the reservoir had dried out in front of the site. **D.** The site, the meander made by the Mula to enter the El Corcovado gorge, and the tectonic escarpments structuring the local landscape, overviewed from the NE on September 24, 2007. **E.** Cueva de Picamartillo, in the canyon of River Vero (Alquézar, Huesca), a modern analogue for the position of Cueva Antón relative to the river at the time of its Middle Paleolithic occupation. **A.-D.:** after Zilhão et al. (2016: Fig. 1), with permission from Elsevier.

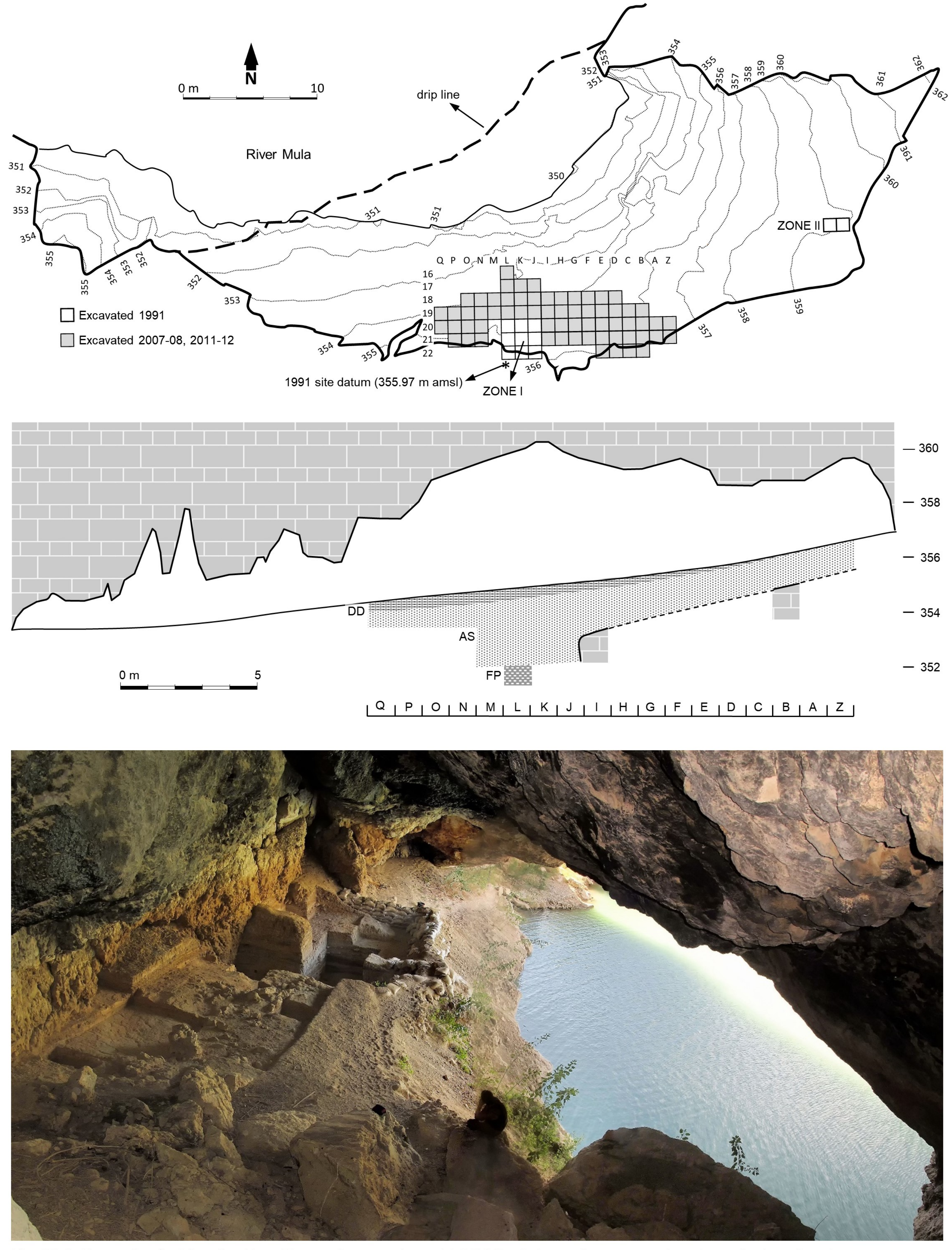

Fig. S2.2. Cueva Antón. The site. Top. Plan and excavation grid. **Middle**. Schematic cross-section along the interface between rows 19 and 20 of the excavation grid (DD = Dam Deposits; AS = Archeological Succession; FP = Fine Palustrine). **Bottom.** The cave's interior overviewed from the East on September 15, 2012, at the end of the field season. Elevations are in m asl. After Zilhão et al. (2016: Fig. 2), with permission from Elsevier.

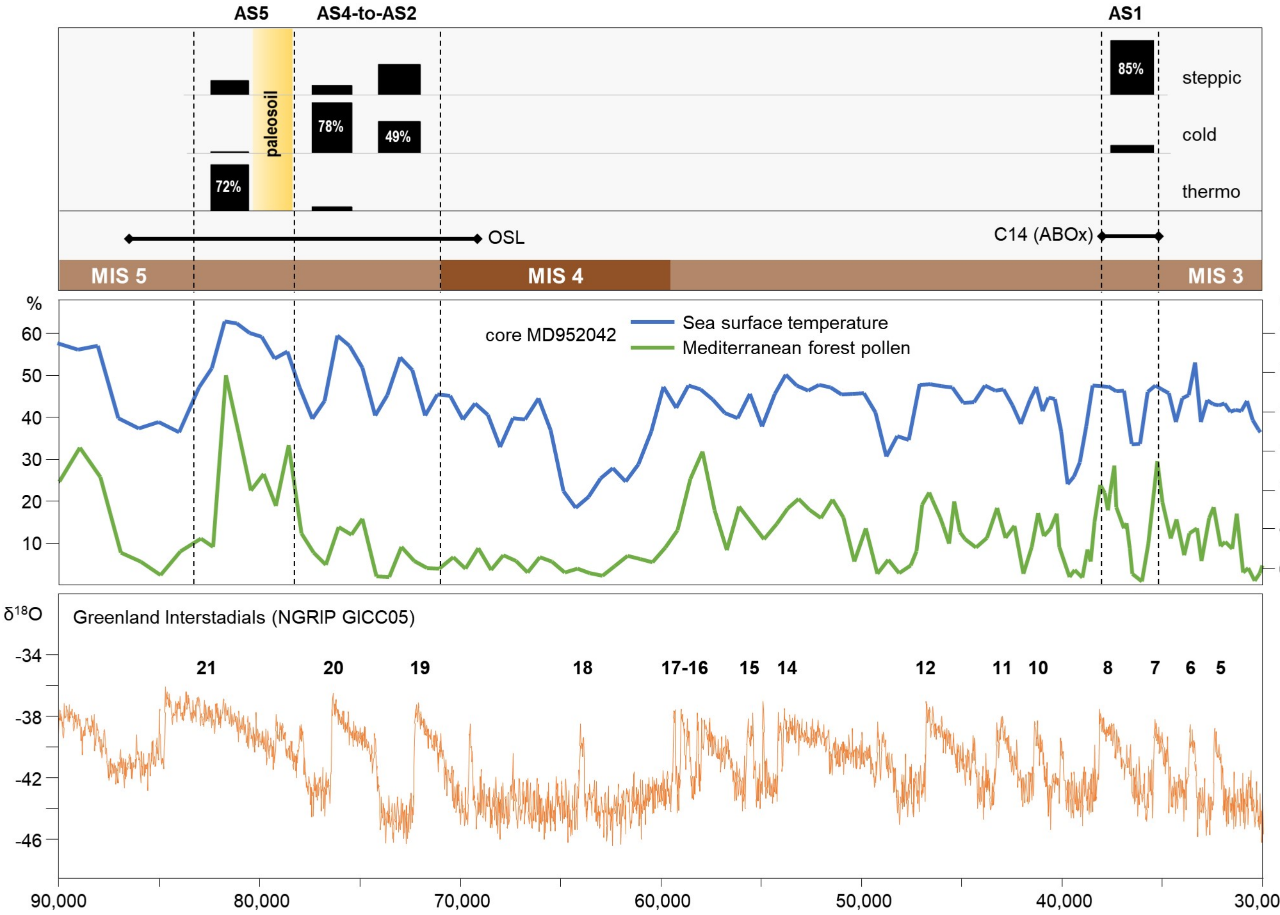

Fig. S2.3. Cueva Antón. Correlation with the global record. For each sub-complex, the vegetation category with the highest charcoal frequency is indicated; comparison is made with the Greenland ice core oxygen isotope stratigraphy, as well as with the variation in sea surface temperature and Mediterranean pollen observed across the 90-30 ka interval in a marine core off the Portuguese coast (Sánchez-Goñi et al., 2008, 2013; Rasmussen et al., 2014; Zilhão et al., 2016). The black bars correspond to the 95.4% probability intervals of the chronometric results; for the OSL dating of the earlier part of the sequence, the range is derived from the values calculated with a sample's water content of 12%, following Zilhão et al. (2016). The paleosoil at the top of sub-complex AS5 and the underlying *Pinus halepensis*-dominated units have been aligned with the 50% spike of the deep-sea Mediterranean pollen curve.

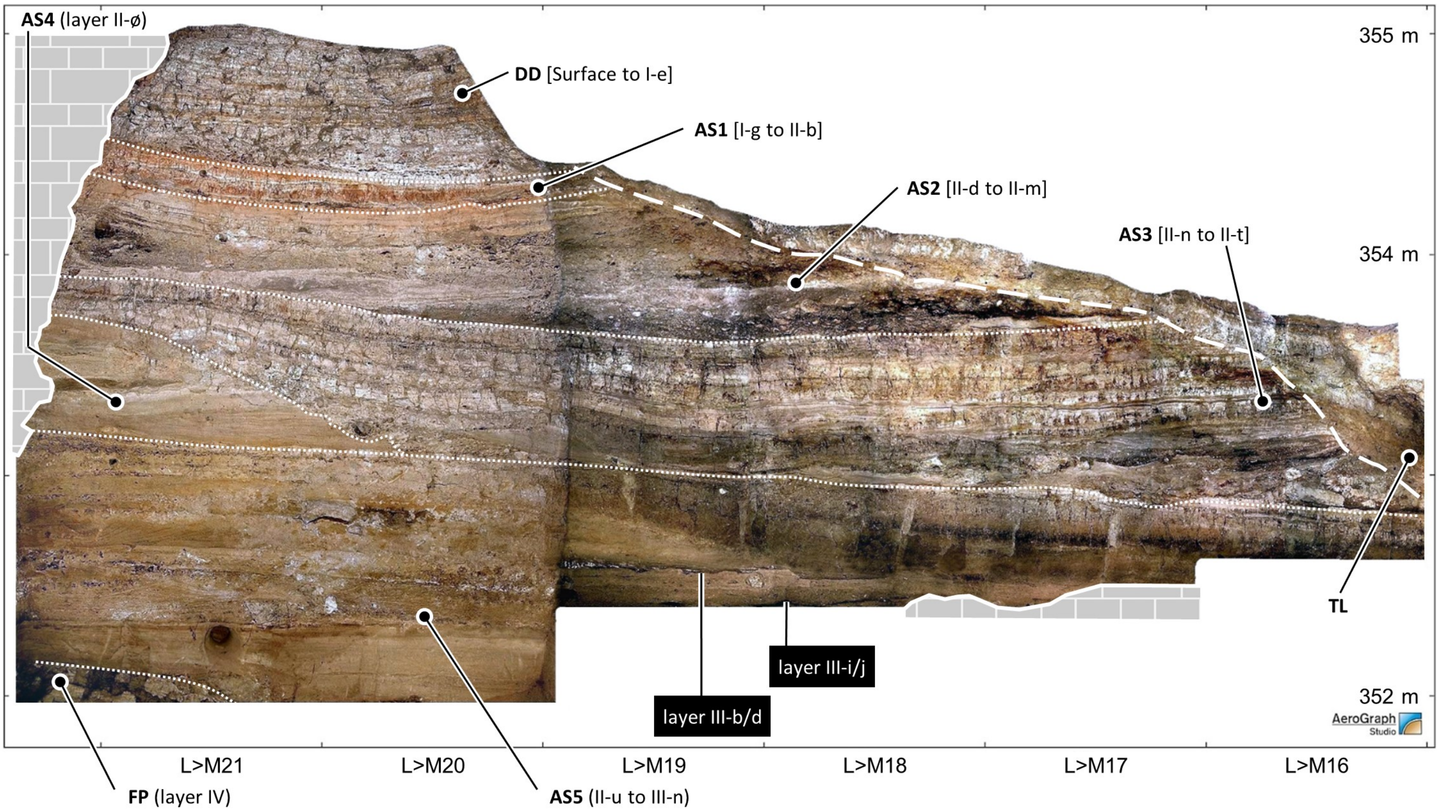

Fig. S2.4. Cueva Antón. The reference stratigraphic cross-section. Orthorectified photomosaic of the west wall of grid units L/16-21 at the end of the 2011 field season. Elevations are in m asl. After Zilhão et al. (2016: Fig. 4), with permission from Elsevier.

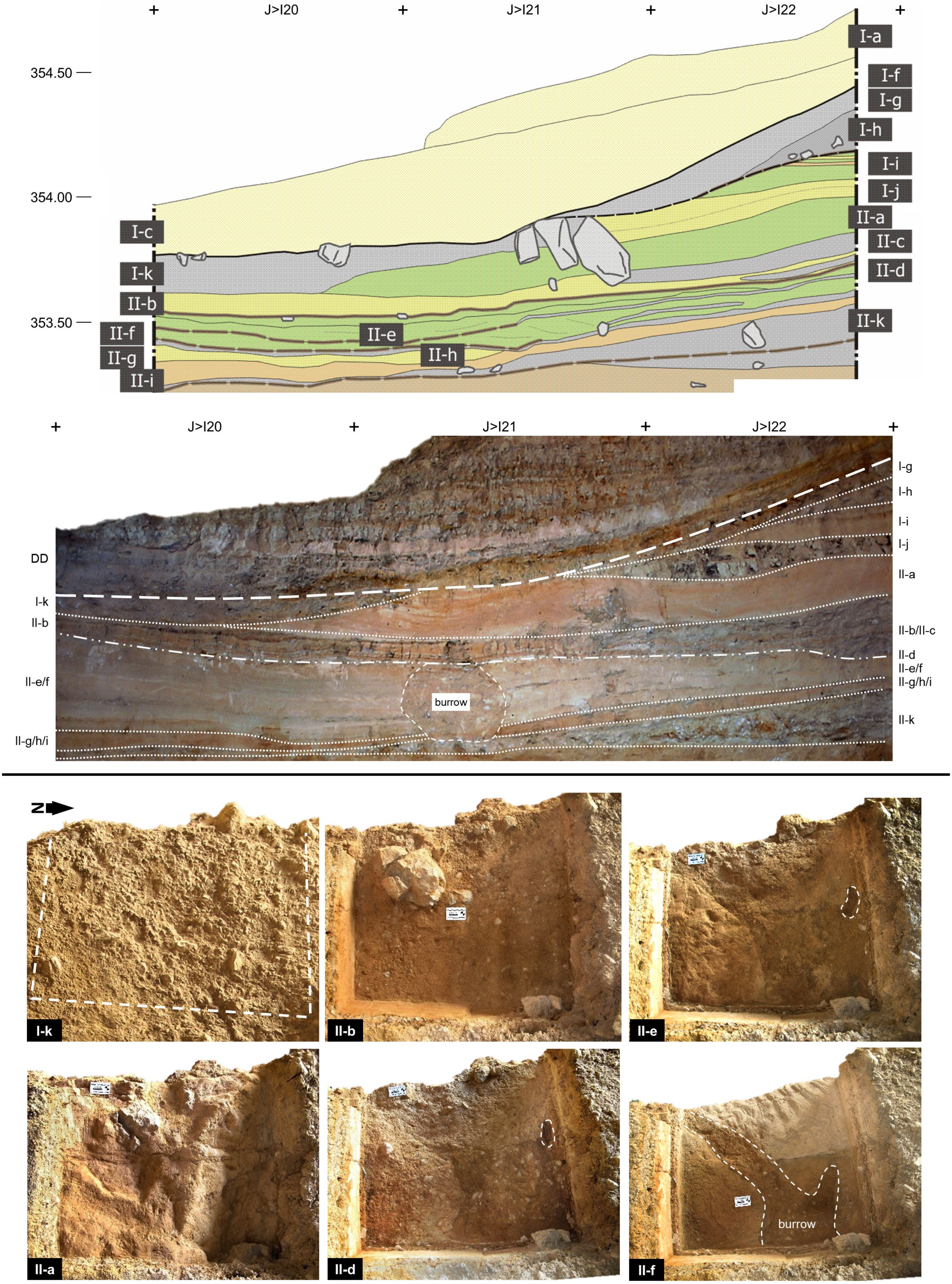

Fig. S2.5. Cueva Antón. Stratigraphic units in the upper part of the East cross-section of 1991. Top. Drawing (after Martínez-Sánchez, 1997; modified by Angelucci et al., 2013) and oblique view taken from the SW corner of the trench at the time of digging (photo: C. Martínez-Sánchez). **Bottom.** Décapage exposures in grid unit I20, excavated in 2011. Elevations are in m asl.

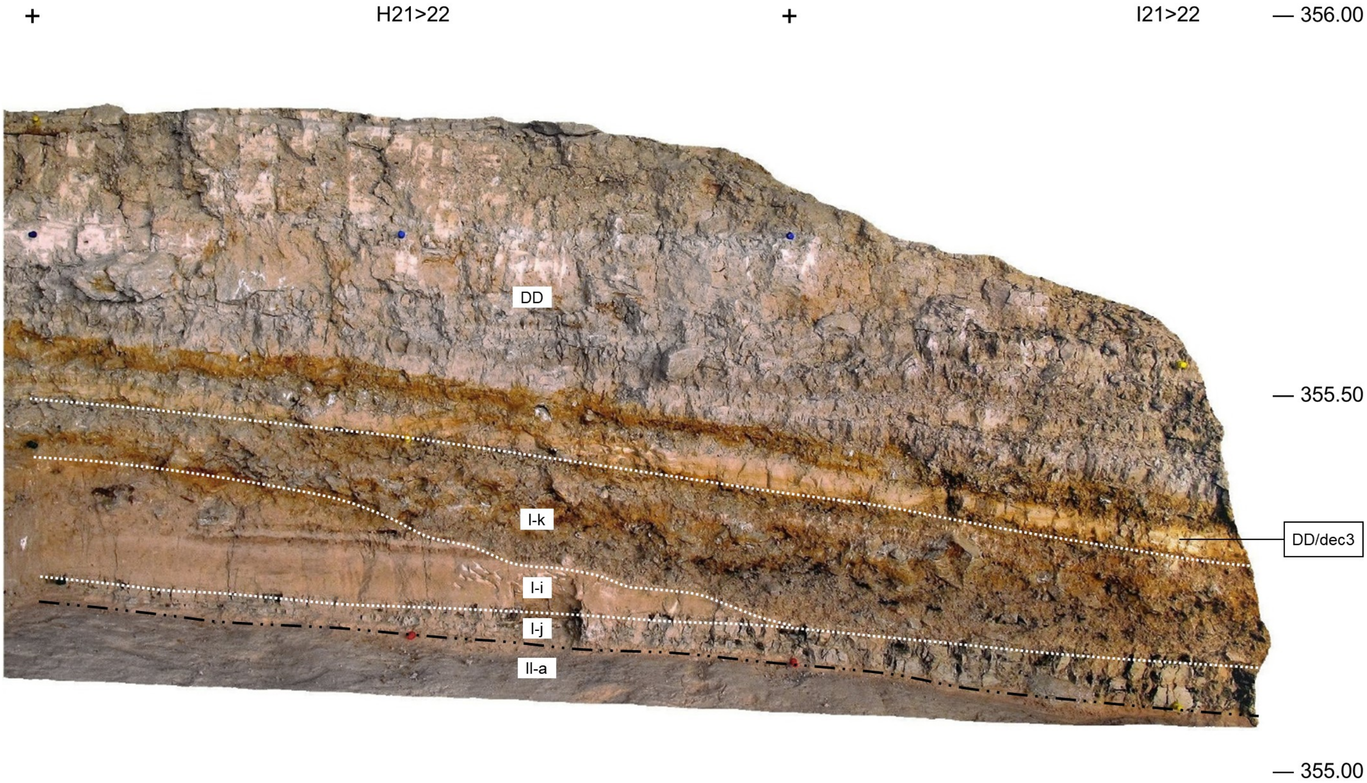

Fig. S2.6. Cueva Antón. South cross-section of grid units H-I/21. Orthorectified photomosaic taken at the end of the 2011 field season. Note the west-dipping of the stratification, the sharp boundaries between stratigraphic units, and the erosional nature of the interface between I-k and I-i. “dec 3” is the designation used in the field for the basal spit in the excavation of the post-dam deposits of the East trench; this unit is of the same texture and structure as many of the inundation levels seen in the Pleistocene sequence, but contained modern, radiocarbon-dated pine charcoal. Elevations are in m asl.

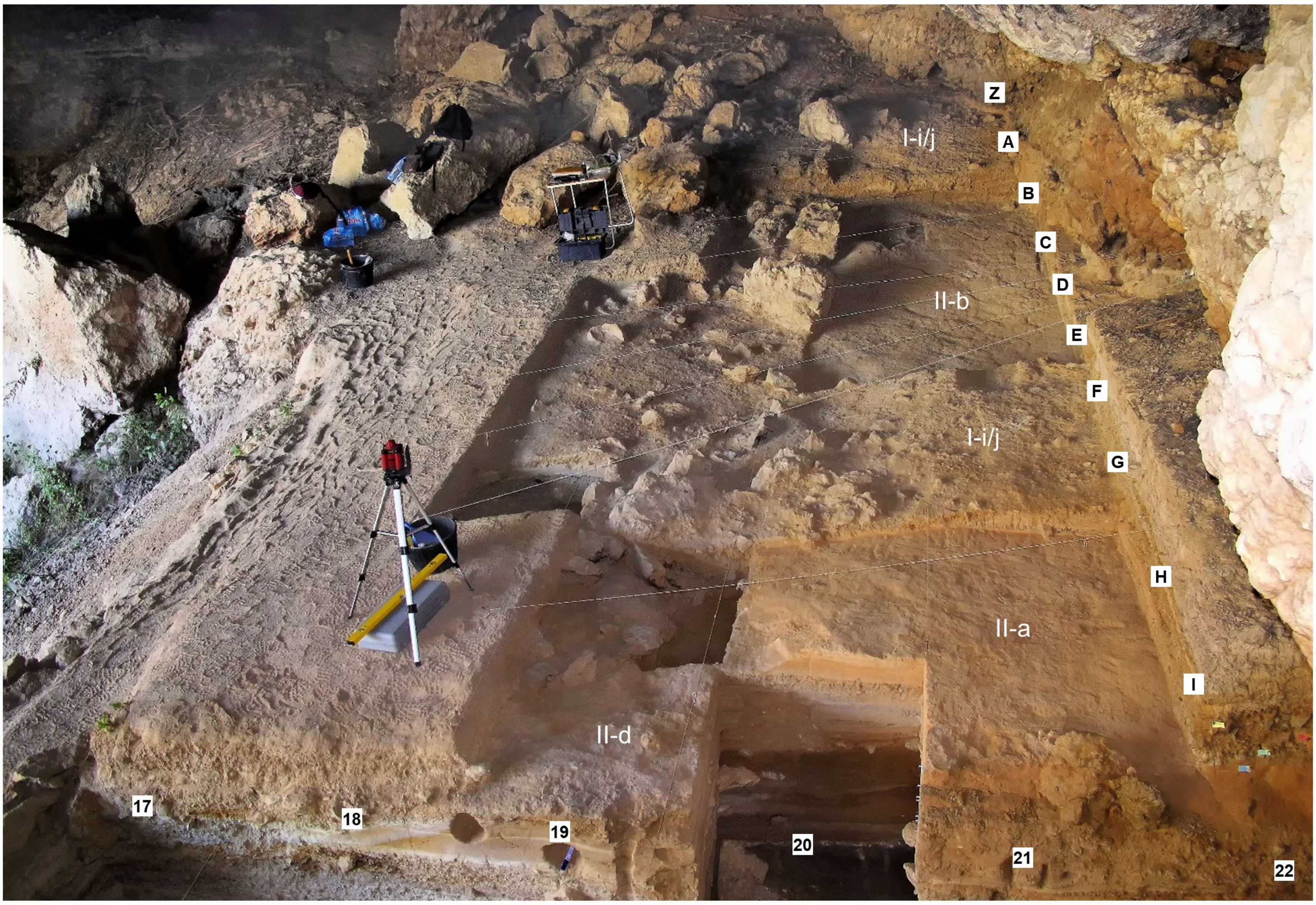

Fig. S2.7. Cueva Antón. The East trench on September 7, 2011. The units exposed at this stage are indicated. In the foreground, note the 1 m² “telephone booth” (square I20), whose sediment was floated in its entirety; the burrow feature here identified at the top of AS2 opened from the base of AS1 in the SE quadrant of grid unit H19.

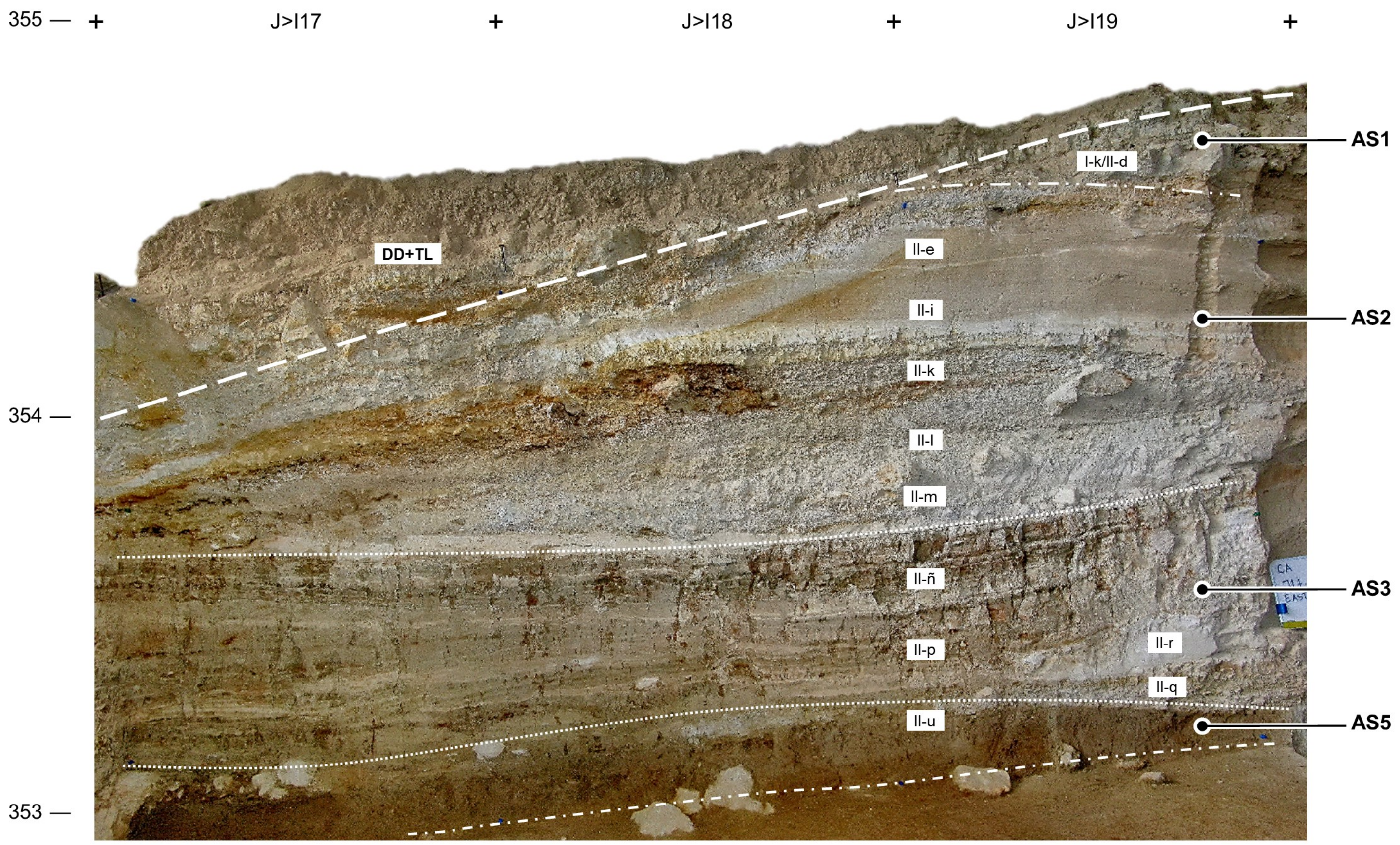

Fig. S2.8. Cueva Antón. East cross-section of grid units J/17-19. Orthorectified photomosaic taken at the end of the 2008 field season. In this area, layers II-a, II-c and II-b wedged out in row 19, and layer II-d changed into a hard calcareous crust marking the interface with the truncated, sloping surface of underlying sub-complex AS2. In this part of the trench, the different layers making up AS1 were archeologically excavated as a single unit, designated "I-k/II-d.". Elevations are in m asl. After Zilhão et al. (2016: Fig. 5), with permission from Elsevier.

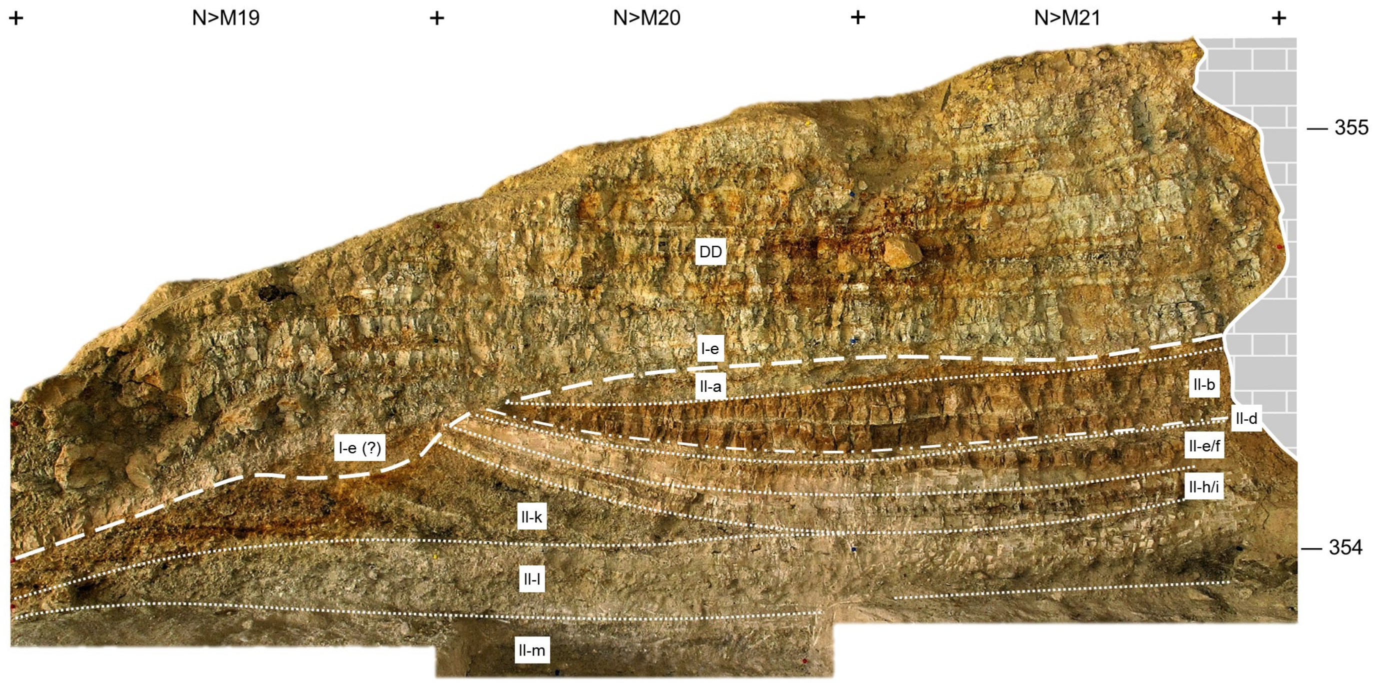

Fig. S2.9. Cueva Antón. East cross-section of grid units N/19-21. Orthorectified photomosaic taken in 2011. Layer I-e is a lens of reworked sediments between the in situ Pleistocene sequence and the dam deposits, rich in rabbit bone and featuring a few, very recent remains of domesticates. Here, AS1 filled an erosional rill. Elevations are in m asl.

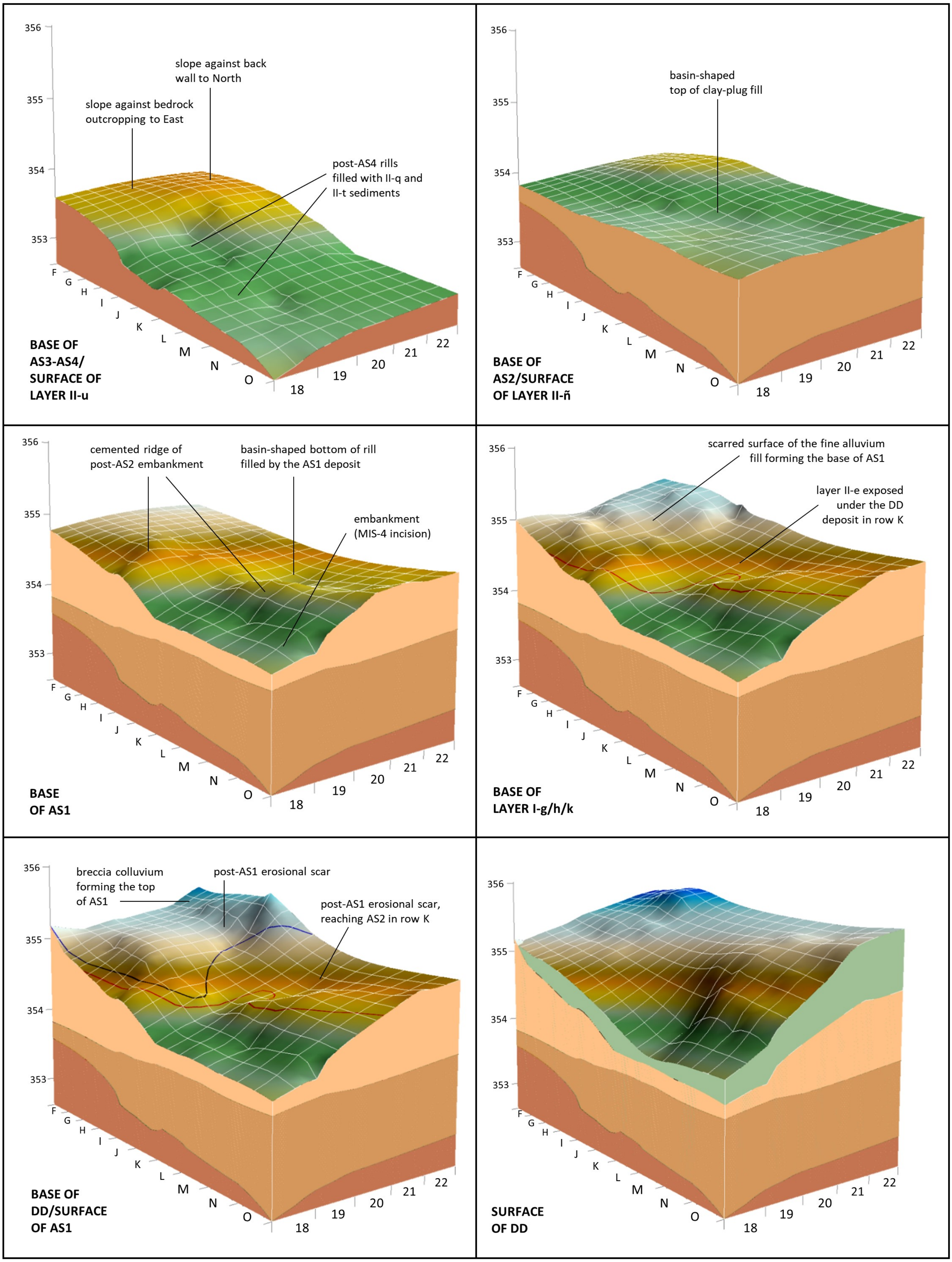

Fig. S2.10. Cueva Antón. The accumulation process in the central part of the site. Simplified 3D model based on the elevation of stratigraphic boundaries as recorded in cross-sections and décapage surfaces. The red and blue lines indicate the outward limits of the basal, alluvial (layers I-i/j to II-b) and upper, colluvial (layers I-g/h/k) parts of AS1, respectively. These boundaries are a proxy for the position of the river margin at the beginning of the corresponding accumulation processes. Elevations are 2× and in m asl.

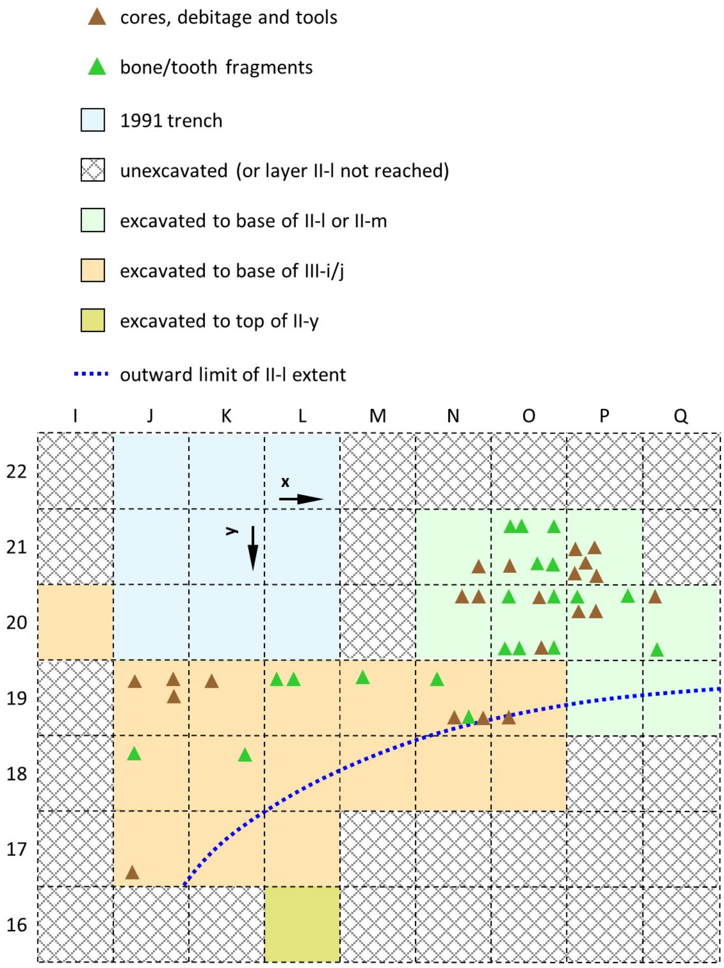

Fig. S2.11. Cueva Antón. Spatial distribution of the finds from layer II-l (piece-plotted or provenanced to quadrant). The low density of the scatter and the small number of stone tool finds relate to the limited space available for settlement once the main channel of the river, which previously ran along the back wall of the cave, migrated out, toward the inner side of the meander, leaving a narrow sand beach behind — layer II-l, which wedged out over underlying layer II-m along the indicated limit.

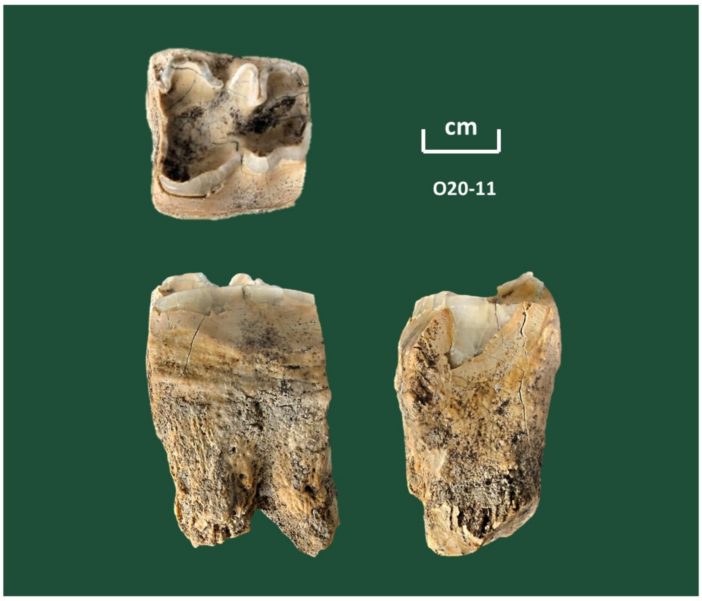

Fig. S2.12. Cueva Antón. Equid tooth from layer II-l. Note the manganese staining and the smoothing and slight polish of the surfaces. These features of the layer's larger mammal assemblage suggest an alluvial origin and, together with the patination seen among the lithics, imply that the deposit corresponds to frequently inundated riverside terrain.

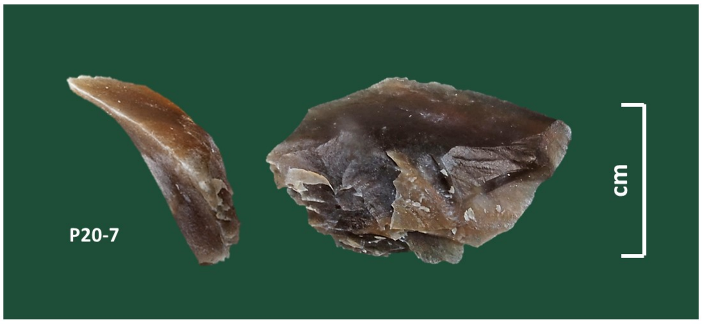

Fig. S2.13. Cueva Antón. Layer II-l retouch debris. Sidescraper re-sharpening flake.

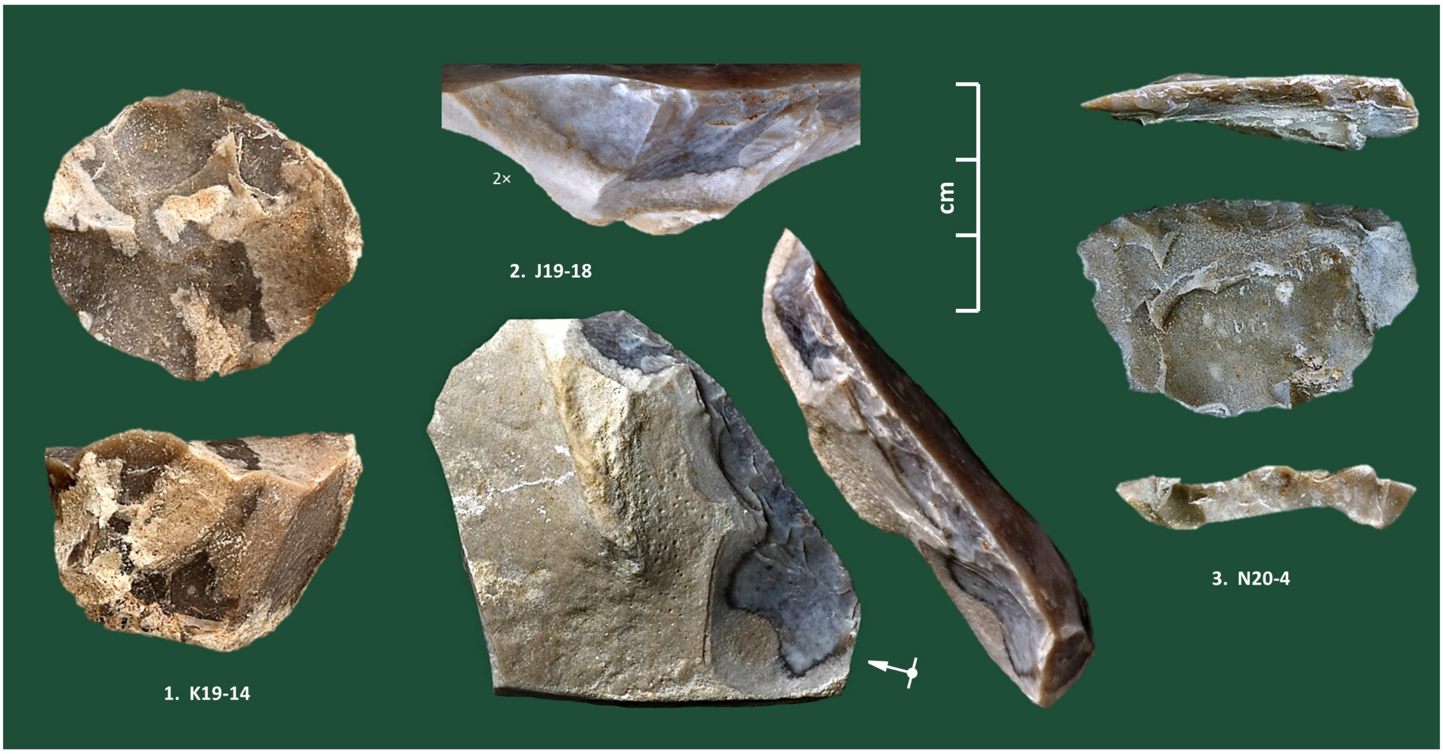

Fig. S2.14. Cueva Antón. Layer II-l flint artefacts. 1. centripetal core; **2-3.** sidescrapers.

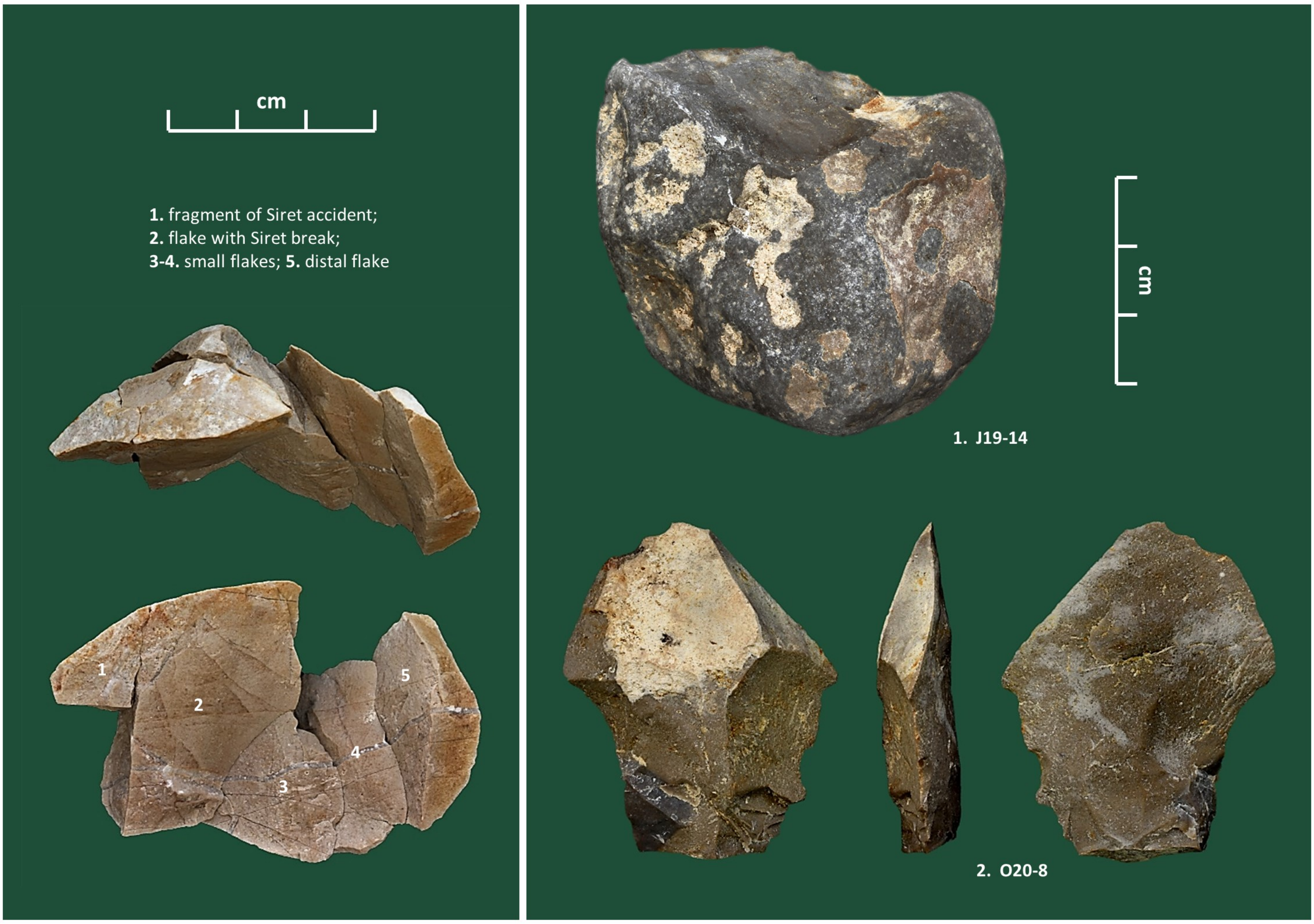

Fig. S2.15. Cueva Antón. Layer II-l limestone artefacts. Left. Refit unit. These five items, plus one chip and one flake fragment from the same block that could not be refitted, come from the NE quadrant of grid unit P21; this tight clustering suggests that both the small size of the layer's stone tool assemblage and the low density of the scatter are unlikely to be due to erosional processes related to surface dynamics. **Right.** Stone tools knapped and used on-site. **1.** tested limestone cobble; **2.** notched flake.

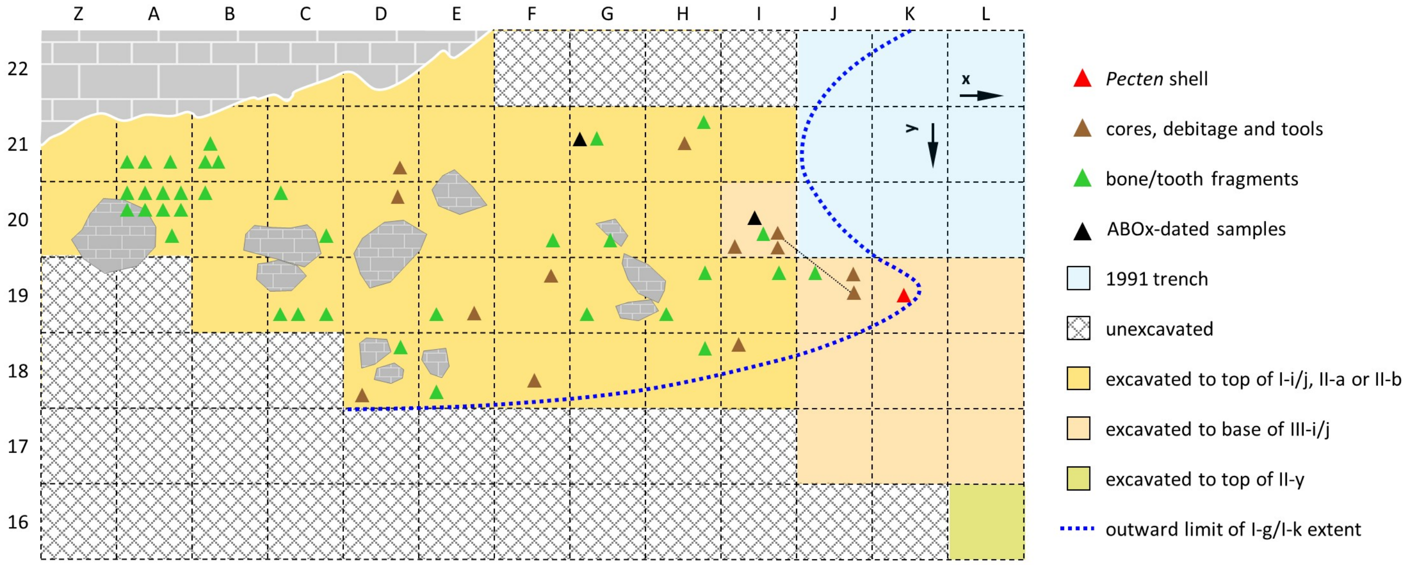

Fig. S2.16. Cueva Antón. Spatial distribution of the finds from layer I-k (piece-plotted or provenanced to quadrant). The low density of the scatter and the small number of stone tool finds relates to the erosional truncation of the deposit along the site's E-W axis and to the limited space available for settlement between the back wall and the river margin. The latter's position is given by the layer's external boundary and implies that, most of the time, accessing this narrow band of dry terrain would require the crossing of a significant extent of inundated or bogged terrain. The dotted line connects refitted items.

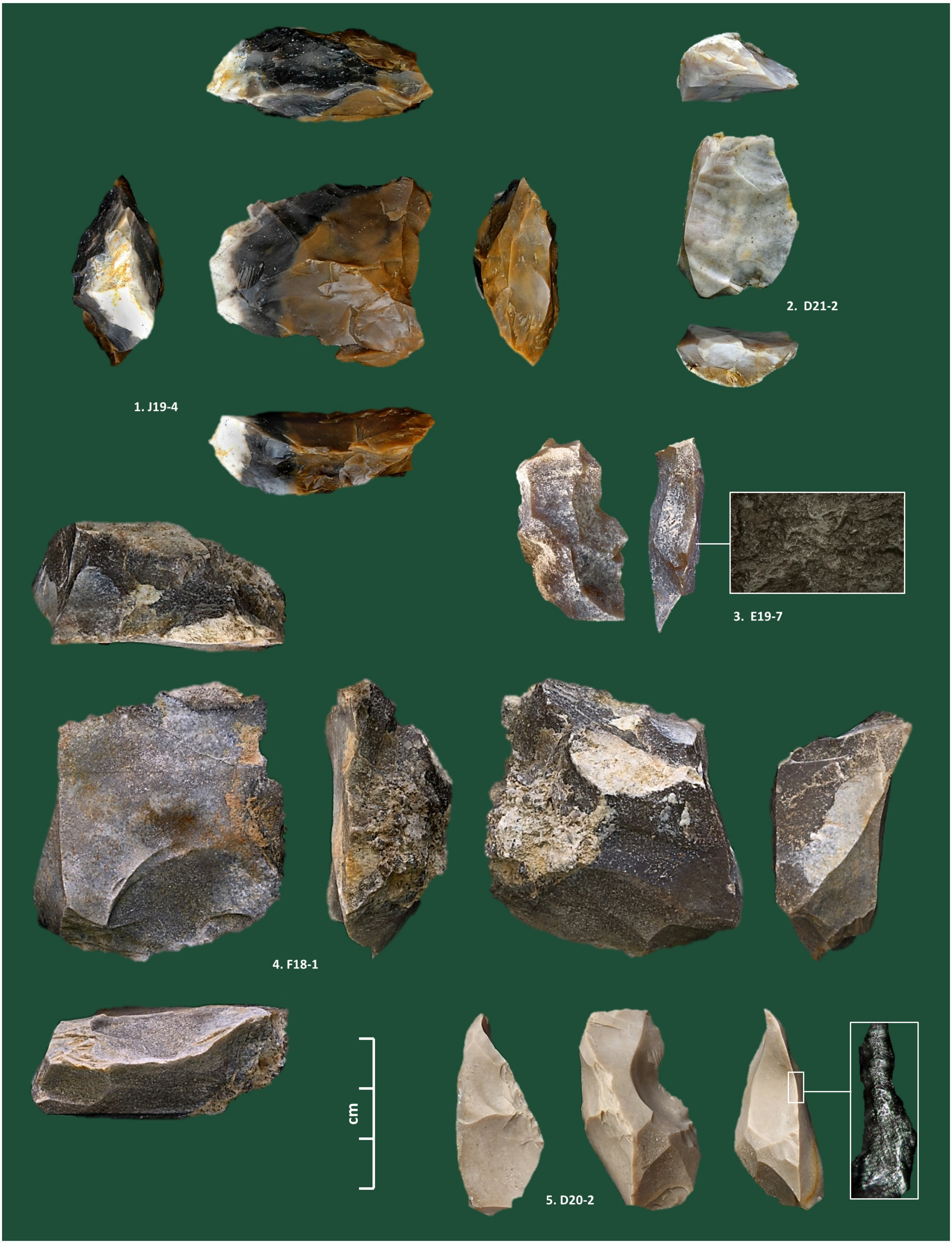

Fig. S2.17. Cueva Antón. Layer I-k cores and retouched tools (flint). 1. centripetal (discoid) core; **2.** bipolar core; **3.** denticulate made on a naturally backed, orange-segment flake blank; **4.** Kombewa core; **5.** notch made on an atypically backed knife (the blank is a core-trimming by-product — removal of the lateral crest of a discoid core). Both retouched tool items present microscopic use-wear evidence (extensive, characteristic but attenuated, greasy polish) indicative of wood-working: on the ventral side (E19-7; (×200) and in the deeper, cutting area of the notch (D20-2; ×400).

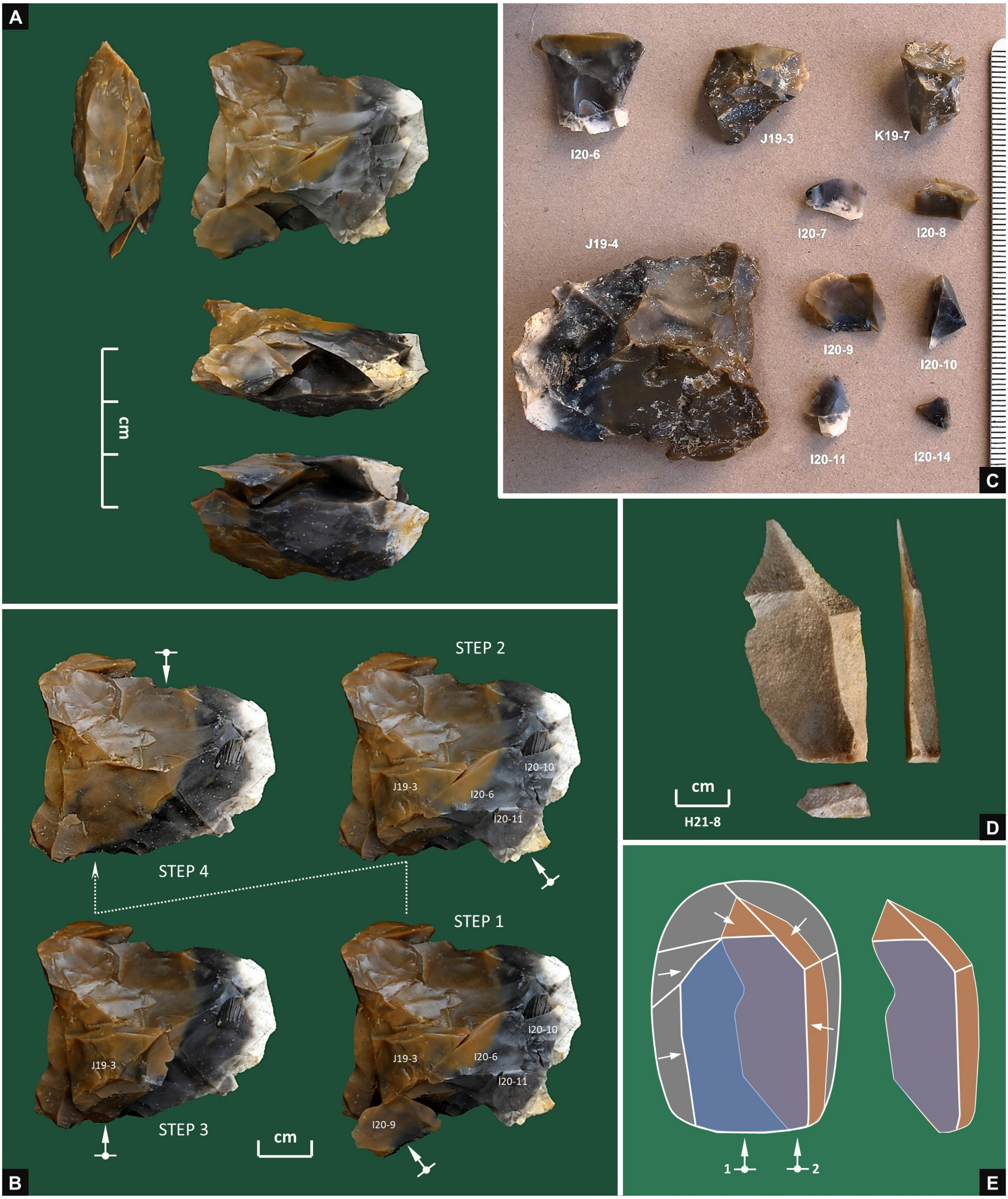

Fig. S2.18. Cueva Antón. Middle Paleolithic technology in layer I-k. A. The J19-4 core refit unit: platform-prepared, centripetal surface-reduction for the extraction of flakes and small flakes. **B.** The refit sequence. Step 1: after the removal of three flakes (one hinged) and a small flake, denoted by the corresponding scars, another small flake (I20-9) was removed from the same side. Step 2: removal of a flake (I20-6+10+11) that hinged and shattered at extraction. Step 3: removal of missing or non-refitted flakes, small flakes and debris (namely, I20-7, I20-8, I20-14 and K19-7) and, lastly, of a trimming flake intent on eliminating a pre-existent hinge and whose basal part broke away (J19-3). Step 4: removal of a flake from the opposite side (denoted by the corresponding scar), followed by fracture and discard of the core. **C.** Field photo of the finds produced by the reduction of the J19-4 core; given their proveniences (grid units J19, K19 and I20), it seems likely that grid unit J20, excavated in 1991, would also have contained items derived from this knapping event, ones that went unnoticed at the time. **D-E.** Laminar flint flake and schematic representation of its position in an unidirectional recurrent Levallois scheme (lateral removal after extraction of an oval-shaped preferential flake).

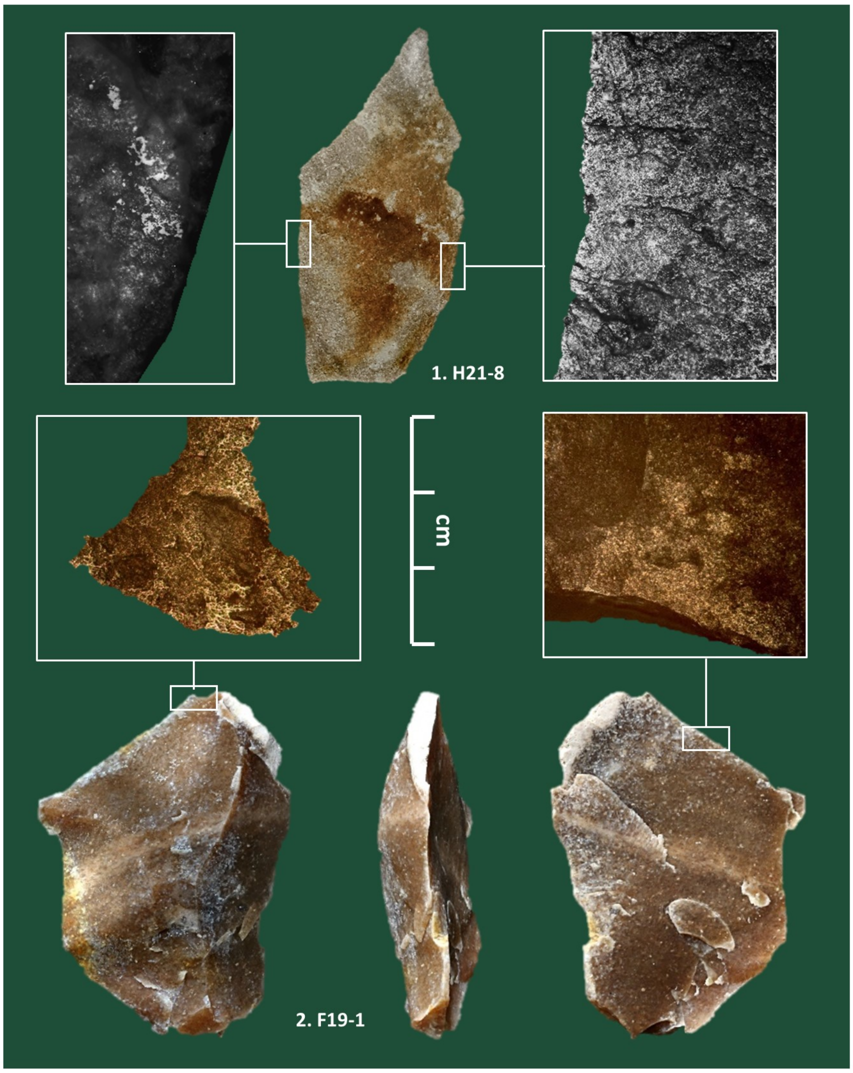

Fig. S2.19. Cueva Antón. Layer I-k non-retouched flint blanks with use-wear. 1. laminar Levallois flake bearing microscopic use-wear on the ventral side — characteristic wood polish on the left, and polish and micro-chipping indicative of work on hard animal tissue (bone or antler) on the right; **2.** blank extracted by bipolar reduction from a flake recycled as a core and bearing wood-working use-wear on both sides of the distal edge (a characteristic polish, greasy and faded, more intense in the high points of surface topography and becoming more diffuse toward the middle and low-elevation areas), more intense on the dorsal side (suggesting implication in planing or smoothing tasks).

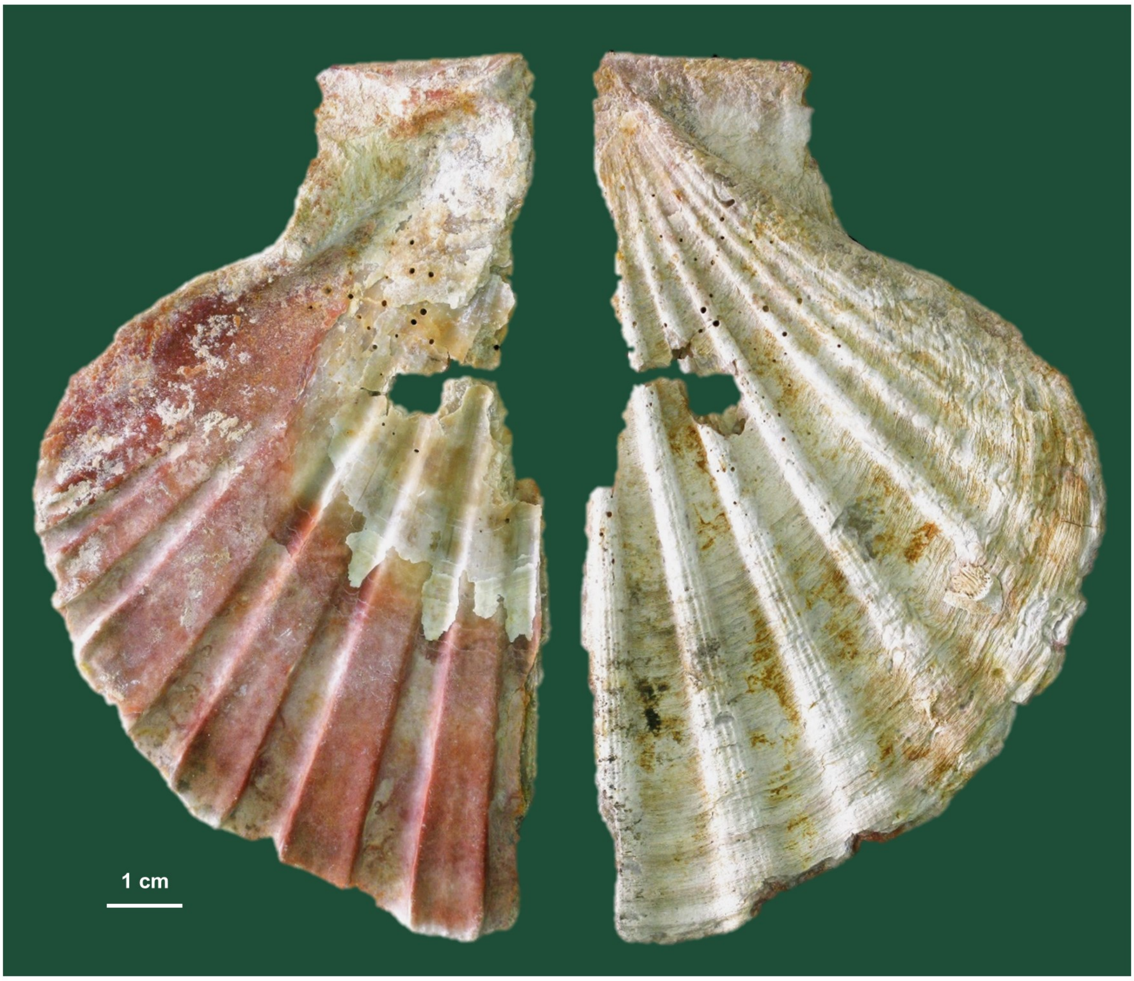

Fig. S2.20. Cueva Antón. Layer I-k symbolic artefacts. Both sides of K19-3, a perforated, ochre-painted half-valve of *Pecten maximus* (cf. Zilhão et al., 2010).

Chapter 3.
The rock-shelter of Finca Doña Martina

3.1. DESCRIPTION

Finca Doña Martina (FDM; 38°04’43” N, 01°29’25” W) is located at the base of an overhanging rock face rising above the left slope of the Rambla Perea valley, part of a large cliff generated by the incision of the watercourse. The bedrock belongs to an upper Miocene, poorly deformed sedimentary formation. At the site, it is formed of limestone: a fine to very fine calcarenite with poorly visible bedding. The back wall is the interior side of a joint exposed by the massive loss of a triangular prism of bedrock. This slabbing-off event created an extensive, dihedral, wedge-like recess in the cliff face that, today, despite subsequent events of overhang collapse, still features a sheltered band of terrain extending up to 5 m behind the drip-line (Fig. S1.2; Fig. S3.1).

The archeologically fertile sedimentary fill rests on and against bedrock. Its accumulation was made possible by the presence of two stepped, largely flat bedrock platforms exposed by archeological excavation in the western half of the recess (Figs. S3.2-S3.3). The upper platform, narrow and featuring a varied micro-relief, runs along the back wall. It covers an area roughly corresponding to rows 4-7 of the excavation grid, decreasing in width from ca.3 m, at the western end of the rock face, to ca.1 m in column O, at the eastern end of the trench. Longitudinally, its surface is nearly horizontal; in row 6, it rises from 402.75 m, in column O, to 402.93 m, in column D. Sagittally, it presents a slight outward dip; along the separation between columns J and K, it goes down from 403.13 m against the back wall to 402.64 m at the edge of the ca.1.5 m escarpment separating it from the lower platform. The latter runs parallel to the back wall, is almost 50% wider than the upper platform (ca.5 m in columns F and G), and dips slightly to the East (from 401.65 m in column E to 401.20 m in column L).

A second, significantly higher (in column G, ca.4 m-high) escarpment marks the outward boundary of the site. Along this boundary, the sedimentary facies changes from a rock-shelter accumulation formed behind a drip-line to a slope deposit devoid of archeological content.

3.2. EXCAVATION APPROACH AND METHODS

At the time of discovery, the site’s ground surface featured a significant dip to the West in the eastern half (Figs. S3.1D, S3.4A). The area where it flattened out — approximately corresponding to column L of the excavation grid — was also where the band of sheltered terrain was widest (Fig. S1.2). These factors underpinned the decision to place here the initial testing trench, open in 2007 in grid units L/5-7; a perpendicular trench was started at the same time in grid units N-O/5 (Fig. S3.4B). In subsequent seasons, these trenches were extended to grid units L/8-9, L4 and N-O/4, and taken down to bedrock. This work established the site’s basic stratigraphic outline and was followed, between 2008 and 2010, by the open-area excavation of grid units D-K/4-7. In this phase, the different strata recognized were exposed across the entire surface of the trench, layer by layer, until bedrock was reached (Fig. S3.4C-E).

Eventually, stratigraphic units that did not exist elsewhere or had gone unrecognized until then were identified at the base of the deposit in the western half of the D-K/4-7 trench. Thus, in 2012 and 2013, to gain a better understanding of the new units and their archeological content, the trench was extended southward to grid units E-G/8-12 (Fig. S3.4F-G). The outward escarpment was identified at this stage.

Given the inconclusive results of geophysical surveys carried out in the interim, a trench (grid units F-G/13-14) was opened in 2014 to test the possibility that a third bedrock platform existed at lower elevation (Fig. S3.4H). This 5 m-deep sounding cut through slope deposits until it hit an accumulation of huge boulders beyond which no further progress was possible. Whether a third platform-cum-shelter exists at FDM remains therefore uncertain, even though, when the general dip of the Miocene stratification is considered, the bottom elevation reached at FDM roughly corresponds to that at which bedrock was found 50 m downstream at La Boja (ADB). Work at FDM concluded in 2016, when grid units M7 and N-O/6-7 were excavated to verify the geometry and boundaries of the basal Middle Paleolithic deposit in the eastern part of the site (Fig. S3.2). The stratigraphic observations made are accounted for in the following, but not so the additional stone tool finds, which remain unprocessed but support the chrono-stratigraphic framework derived from the 2007-2014 material. In the end, the excavation's planar surface and volume totaled 59.9 m^2 and 90.3 m^3, respectively.

During the initial testing phase, the excavation proceeded via slicing the sedimentary fill into spits that respected observed stratigraphic boundaries whenever possible. If the thickness and heterogeneity of the deposit made the recognition of such boundaries difficult, or if these where gradual and hard to follow, the testing operations switched to horizontal spits of arbitrary thickness (5 or 10 cm). A good grip on the characteristics of the succession was thus acquired, and produced the guidelines and criteria required to undertake the open-area excavation of the different strata recognized. This work proceeded entirely along natural stratigraphic boundaries and, when necessary, via the subdivision of the different units into spits that followed the dip of the stratification (Fig. S3.5). The finds from the arbitrary spits of the testing phase were retrospectively assigned via comparison of their **x,y,z** information with the volumetry of stratigraphic units as reconstructed from cross-sections. Where ambiguity remained, no assignment was made and such finds were excluded from consideration.

Post-depositional disturbance features of two kinds were encountered. Patches of grey-colored sediment extending down from the top soil (layer 2) affected layers 3 and 4 without physically altering the general texture and structure of the deposit. These were diagnosed as accumulations of organic matter related to the vegetation growing on the surface and treated as lateral variation. Westward of the 2007 trench, a large disturbance feature, denoted by a much looser sediment fill, the near absence of finds, and the presence of large vertical blocks, affected a significant portion of layers 4 and 5 (Fig. S3.5F). Diagnosed as a rabbit warren, this feature was entirely emptied prior to excavation of the rump of those units. A similar procedure was followed in the case of a tunnel identified in grid units E-G/8 along the edge of the upper platform (at the elevation of layers 8 and 9, and partly filled with layer 7b material).

All décapage surfaces and stratigraphic cross-sections were drawn and/or photographed. The DStrectch plug-in for ImageJ was used to highlight color contrasts and produce prints used in the field to help with the décapage of stratigraphic interfaces (Fig. S3.5J). Photo mosaics were assembled using PT GUI or Microsoft ICE, and orthorectified with the University of Venice's RDF software. Elevation maps, 3D models and area/volume estimations were produced with Surfer. Elevations and finds were recorded and/or piece-plotted with the help of a laser level, to the nearest centimeter, against the grid and the site datum, which was placed on the back wall of the shelter at the elevation of 405.85 m.

Cores and core fragments, complete and proximal blades and bladelets, retouched tools and fragments thereof were systematically piece-plotted, and, in the lowermost Middle Paleolithic, the same for complete flakes. The rest were bagged together with sieve finds from the same spit and grid unit. The charcoal for anthracological study was collected in similar manner, but the fragments spotted during excavation as suitable for radiocarbon dating were all piece-plotted. In addition, multi-kg samples of carbon- or charcoal-rich sediment associated with hearths or their remnants were saved for subsequent flotation in the laboratory. In the 2007-2010 field seasons, finds were numbered sequentially, 1-to-*n*, per grid unit (e.g., L5-1 to L5-*n,* H7-1 to H7-*n*, etc.); in 2012-2016, they were numbered sequentially, 1-to-*n*, per year of excavation (e.g., 2012-1 to 2012-*n*, 2013-1 to 2013-*n*). The sandy, dry nature of the deposit dispensed wet-sieving, so the excavated sediment was dry-sieved in its entirety through two-sieve stacks with meshes of 2 and 1 mm (Fig. S3.5A). Undisturbed samples for soil micromorphological analysis of the sediments and of the few fire features identified were collected throughout.

Despite the non-acidic nature of the deposit, bones were seldom present. Even if no more than very small splinters, all were therefore piece-plotted. Mollusk shell preserved well. Land snail remains were particularly abundant in disturbed or organic matter-rich areas of the sedimentary fill, and the dominant taxon (*Iberus alonensis*) is well known for its burrowing behavior and preference for sheltering in rocky fissures (Moreno-Rueda, 2006). For these reasons, land snail shell was considered as post-depositionally intrusive if not natural background noise (the latter otherwise corroborated by the present-day ubiquity of *I. alonensis* shell in the slopes of the gorge). Given the inland location of the site, marine and fluviatile mollusk shells were, however, clearly anthropogenic. Therefore, such shells, often pierced for use in body ornamentation, were piece-plotted when seen at excavation, even when they were no more than small valve fragments, while land snail remains were discarded.

Surveys of the region were carried out to identify the provenience of the diverse flint varieties represented. Several potential sources located within a radius of 20 km have been identified but this aspect of the project remains in a preliminary stage. The study of lithics used traditional typological classification but otherwise followed the "economics of stone" approach outlined in Zilhão (1997): in the analysis of reduction sequences and site function, "splintered pieces" were treated as bipolar cores, while "burins" and thick (carinated and nosed) "scraper" forms were defined as blanks for the extraction of bladelet products.

All cores, retouched tools and unretouched blanks (complete flakes in the Middle Paleolithic assemblage; blades and bladelets, including fragments, in the Upper Paleolithic) were individually measured, weighed and recorded for a set of technological attributes. To enable comparison across the Middle/Upper Paleolithic technological divide, “small flakes” (<2.5 cm) were counted separately. Use-wear analysis of selected samples was based on differential interference contrast microscopy, carried out with a BHMJ Olympus model (at ×200 or ×400 magnification), and followed standard recommendations for the cleaning and preparation of the material (Plisson, 1985).

3.3. STRATIGRAPHIC OUTLINE

All units contain local limestone fragments, variable in shape, size and abundance, and are enriched of post-depositional calcium carbonate or gypsum. Due to the complex geometry and variable extent of the different stratigraphic units, none of the reference cross-sections and associated décapage plans (Figs. S3.6-S3.13) features the complete succession, which, from top to bottom, is made up of the units described below, designated in the field as “layers” and primarily defined after the L>M cross-section (Fig. S3.8) (all Munsell color descriptions are on moist sediment).

Unit 1

Thin surface layer disturbed by trampling**,** exposed only in the inner parts of the rock-shelter, along the back wall. The texture is silt, with few stones (often with a sub-horizontal orientation plane), a weakly developed “platy” structure, and a clear boundary.

Unit 2

Poorly developed A horizon atop the succession. It is a 10YR4/3 soft silt with very weak crumb structure, some roots, common dispersed organic matter, and a sharp linear boundary. Stones of distinct types, randomly distributed and oriented, are common. In areas of the excavation extending beyond the drip-line, layers 4, 5 or 7, bioturbated in a manner resembling unit 2, are exposed as ground surface. The horizontal transition from a “buried-under-the-overlying-strata” to an “exposed-at-ground-surface” condition being gradual, the bioturbated, surface-exposed, devoid-of-finds reaches of those layers were not differentiated during excavation, as reflected in the captions to the cross-sections illustrated here.

Unit 3

Possibly an E horizon poorly developed from slope sediment. It is a 10YR5/4 sandy silt, massive, slightly firm, with no organic matter and a sharp boundary. Stones of distinct types, with random distribution and orientation, are common. In section view, it is discontinuous, weakly visible. At décapage, it consisted of a variably mottled lens of light, fine, largely loose sands rich in angular debris and containing numerous fragments featuring in situ shattering (frost-weathering?), comprised between a dark Unit 2 and a somewhat cemented, salmon- or light grey-colored Unit 4 (Fig. S3.5B, S3.5F). As is also the case with Unit 2, the stone tools retrieved in Unit 3 are of Epimagdalenian affinities.

Unit 4

Laterally variable unit in which two facies were recognized. **Unit 4a** is a 10YR4/4, massive, slightly firm sandy silt with scarce stones (the tabular ones often being parallel to the lower interface), scarce, dispersed organic matter (increasing toward the back wall), and a clear boundary. **Unit 4b** is the same without the organic matter and of color 10YR6/6. Unit 4a was restricted to column L of the excavation grid and adjacent areas of columns K and M-O. It remains to be clarified whether this subunit's organic matter content is anthropogenic or reflects bioturbation and soil formation during a depositional hiatus. The lithics retrieved in Unit 4 are of Upper Solutrean affinities.

Unit 5

Thick layer of breccia/diamict**,** formed of cross-bedded or festooned, poorly recognizable lenses, with stone-line intercalations. It is a 10YR6/6 sandy silt with no organic matter, firm, weakly cemented by calcium carbonate, and a clear boundary. Stones of different types are common. It yielded Upper Solutrean diagnostics.

Unit 6

Organic matter-enriched lens, wedging out in all directions (Figs. S3.5C, S3.8, S3.14). It is a 10YR2/1 sandy silt with some clay, few stones, frequent, finely dispersed organic matter, massive, firm, and with a clear boundary. It remains to be clarified whether this facies stands for a remnant, poorly developed A horizon, or for a localized accumulation of micro-charcoal particles related to human occupation.

Unit 7

Layer of breccia/diamict material. It is a 10YR5/4 sandy silt, massive, firm, with some calcified roots penetrating from unit 5, weak carbonation and a sharp boundary. Stones are scarce (near wall) to abundant (outwards). The presence of large, darker mottles denotes bioturbation (few, but relatively large burrows filled with organic sediment). At excavation, layer 6 was treated as lateral variation of layer 7 and, archeologically, they have been considered as a single unit, layer 6/7, within which the boundaries of unit 6 broadly coincide with the area featuring the highest density of stone tool finds. In rows 4-6 of the western part of the D-K/4-7 trench this deposit was shallow and clearly post-depositionally disturbed. Along row 7 and the outward half of row 6, its surface was scoured by erosion, leaving a steep micro-ravine atop and against which unit 5 came to lay (Fig. S3.14). The stone tools from layer 6/7 are of Gravettian affinities.

Unit 7b

Similar to unit 7, which it underlies in the western part of the site. The excavation trench in which this unit was recognized (E-G/8-12) extends outward of the drip-line, explaining why the sequence is enriched in large horizontal stones, some of which are >1 m-long, derived from the degradation of the shelter's overhang (Figs. S3.10-S3.12). As with overlying Unit 7, this layer yielded diagnostic Gravettian lithics.

Unit 8

Clast-supported fine to medium breccia filling channels and scours cut in underlying unit 9 (erosive features oriented E to W, with platy stones sometimes imbricated and often weathered). The matrix is a fine material as in unit 7b, locally enriched in sandy silt derived from unit 9. The boundary is erosive and the mass of the deposit presents horizontal discontinuities making for situations of lateral contact of its edges with the interface between layers 7b and 9 (Figs. S3.12-S3.13). The stone tools retrieved in the body of unit 8 are of Aurignacian affinities.

Unit 9

Relatively homogeneous, 10YR6/4 silt with fine sand and scarce stones (plus occasional fragments of reworked clay at the base), massive, firm, poorly cemented by calcium carbonate, with discrete packing, no organic matter, and a gradual, poorly distinct boundary. Some darker, large mottles denote bioturbation. During fieldwork, further subunits (9b and 9c) were distinguished, based on local variation in texture and amount of stones. Inward, this unit leans against the edge of the upper bedrock platform; outward, it becomes much thicker (ca.1 m) and dips S/15° as it extends beyond the boundary of the lower platform (Figs. S3.8, S3.10). In the N-O/4-5 grid units and along the southern edge of row 7 of the D-K/4-7 trench, layer 9 outcropped as narrow bands of yellow sandy sediment bounded by the 5>6 and 7>8 cross-sections, outward, and by the upper bedrock platform, inward. At the time of excavation, these bands of sediment, which featured significant bioturbation, were thought to represent the basal aspect of layer 7. They were eventually recognized for what they were, and the corresponding excavating spits and finds retrospectively assigned, based on analysis of the cross-sections and on lateral continuity with the typical aspect of the unit — first defined in L/7-8 and subsequently found to extend to surrounding grid units with the 2010 excavation of the M column, the 2012 excavation of E-G/8-9, and the 2016 excavation of M7 and N-O/6-7. All stone tools retrieved in Unit 9 are of unambiguous Middle Paleolithic (Mousterian) affinities.

Unit 10

Also known as “pisto,” this archeologically sterile unit was first recognized in the L/7-8 deep sounding, where it corresponded to a 7.5YR4/4 massive, weakly porous silty loam with common, mm- to 2 cm-long fragments of pink/red laminated clay/marl, scarce fragments of local rock, occasional fragments of calcite/aragonite crusts, common microcrystalline gypsum nodules, scarce carbonate and Mn-Ox nodules, no organic matter, and a sharp boundary to bedrock. This unit was also found in E-G/8-10. Here, and especially so towards the back wall of the lower bedrock platform, it was rich in large, angular, non-weathered blocks, and heavily cemented. In this area, a <5 cm-wide fissure, internally padded with a continuous layer of calcite/aragonite crystals, separated the sediment from the escarpment leading to the upper bedrock platform against which it leaned.

Unit 11

Thick, archeologically sterile breccia making up the bulk of the FDM talus scree**.** Formed of poorly stratified alternations of a clast-supported breccia of local rock (locally partially open-work) within a 10YR5/6 silty sand with common to frequent stone fragments, it dips S/15° and was recognized over a thickness of ca.3 m in the F-G/13-14 sounding (Fig. S3.10).

Unit 80

Saprolite developed from bedrock. It is a fine open-work breccia made of cm-sized platy and tabular bedrock fragments oriented parallel to bedrock. Prior to recognition of the basal horizons of the succession lying beyond the edge of the upper platform, this unit separated layer 7 from bedrock and, as such, was initially designated as "layer 8" (in e.g., annual excavation reports and in Zilhão et al., 2010), subsequently changed to unit 80.

3.4. RADIOMETRIC DATING

The provenience, composition and nature of the samples submitted for radiocarbon dating are given in Table S3.1, and the results obtained are listed in Table S3.2. No charcoal was found in layers 3, 5, 8 and 9; hence, the lack of results for these units. In 2012, sediment samples were taken in the E-G/8>9 cross-section for the luminescence dating of layers 7b and 9. The tests carried out in the luminescence laboratory of the Institute of Geography, University of Cologne, indicated, however, that the material was unsuitable for dating. The cause probably lies in the sediment being freshly derived from the weathering of the local bedrock and, thus, not having undergone the number of transport-deposition cycles required to develop favorable luminescence characteristics.

Radiocarbon dating was carried out on samples submitted to the Vienna laboratory (VERA). Despite reasonable size and good preservation of tissue structure, enabling ready taxonomic identification, the standard Acid-Base-Acid (ABA) pre-treatment proved to be too aggressive for the site's charcoal. The cause probably lies in leaching and other geochemical processes related to site formation mechanisms. Regardless of this problem's exact nature, the consequence was that dating had to be carried out on the humic acids fraction or after a mild form of ABA. Incomplete decontamination, or contamination by younger humic acids present in the humic acids fraction being an issue, the results must be treated as minimum ages only.

At nearby La Boja, better charcoal preservation enabled measurement of the ABA-treated sample material (and obtained humic fraction), and, in some cases, the more aggressive ABOx-SC extraction procedure was also applied (see Chapter 4). No difference was observed between the ABA and ABOx-SC results for a single sample. Some of the humic acid results were up to two millennia younger, while others fell in the same statistical ball park. The latter was the case for results no older than about 21-23 ka (uncalibrated), but also for some samples in the 40-45 ka (uncalibrated) range. These comparative data, the lack of evidence for soil formation in the areas of FDM where the dated samples came from, and the stratigraphic consistency of the results obtained (Figs. S3.15-S3.16) suggest that, even though almost certainly underestimated, the results reported in Table S3.2 probably are only moderately so.

The layer 4 samples all come from one and the same hearth feature and ought to have returned the same date. That such was not the case corroborates that the results are affected by incomplete decontamination or the presence of younger humic acids in the humic acids fraction, and implies that the older result, placing the feature in the 22.8-23.4 ka cal BP range, best approximates its real age. This interval is likely to still represent something of an underestimation but, be it as it may, the Upper Solutrean affinities of the layer 4 stone tools constrain its accumulation to no earlier than the beginnings of the 24th millennium cal BP.

One of the layer 6/7 samples (H6-51) comes from the periphery of a hearth, while the other (H6-63) was collected a few cm underneath, near the contact with layer 80. Assuming that the oldest result (VERA-5367dHS_2) is least affected by residual contamination and, hence, provides the best age estimate, layer 6/7 would date to the first half of the 31st millennium cal BP. However, this sample was made up of scattered charcoal and, given the gradual nature of the stratigraphic boundary with layer 7b, it cannot be excluded that some relates to the latter. Indeed, if layer 7b extended toward the back wall as no more than a thin lens atop the basal saprolite, it could well have gone unrecognized beyond the boundaries recorded during the décapage of the basal spits of row 6 of the E-K/4-7 trench. Bearing this possibility in mind and knowing that the VERA-6170HS result comes from the sample most closely associated with the layer's single preserved hearth feature (H6-51; Fig. S3.15), assigning to this occupation an age within the 29th or the 30th millennium cal BP would seem more reasonable.

The result for layer 7b (VERA-5368HS) is consistent with those for overlying layer 6/7. At the adjacent site of La Boja, the corresponding stratigraphic slot (horizon OH14) is undated and was poor. However, it yielded Gravettian diagnostics, and the age of the horizons that sandwich it is reliable: 27,260±230 BP (VERA-5789) for OH13; 30,548/+363/-347 BP (VERA-6153) for OH15 (see Chapter 4). The humic acids result for VERA-5789 is in agreement with the ABA result. Indeed, statistically, the three Early Gravettian results for La Boja OH14 and FDM layer 7b are indistinguishable, while broad synchronicity in the phasing of the two sites' sedimentary accumulation is to be expected. Thus, we can conclude that FDM layer 7b likely dates to the end of the 32nd millennium cal BP.

3.5. SITE FORMATION

A 3D model of the extent, geometry and stratigraphic relationships of the different units, based on the combination of the cross-section and décapage records, is given in Fig. S3.17.

As the basal deposits (layers 10, 11 and 80) do not overlap (Fig. S3.17A-D), the sequence in which they formed remains to be clarified and can only be addressed in indirect manner. With current evidence, layer 10 would seem to be the oldest. The lack of finds and organic matter, combined with the non-weathered condition of the angular detrital fragments it contains, suggest that layer 10 could well correspond to the interior deposit that once filled the joint along which the rock-shelter formed and that the slabbing-off event triggering the site formation process eventually exposed. If so, the geometry of this unit's erosional surface (Figs. S3.8, S3.10-S3.11) would result from the initial phase of configuration of a slope linking the streambed below with the recess thus newly formed in the valley's left bank cliff face.

In Fig. S3.17C, layer 80 appears to cover bedrock in the interior grid units of the western part of the trench in discontinuous manner only; this is due to the fact that saprolite was often too thin there for differentiation at décapage to be possible. Beyond the drip-line, atop the largely horizontal lower platform, the absence is, however, genuine. Layer 80 therefore corresponds to the degradation of the local bedrock under sheltered conditions, prior to a time when the formation of slope deposits outside and lower down generated a topographic baseline enabling the accumulation of a stable rock-shelter fill. The formation of the saprolite and the accumulation of the slope deposit are probably coeval, but all that can be said with certainty is that they pre-date the accumulation of layer 9, which leans against or overlies layer 80 inward of the drip-line and, outward, overlies layers 10 and 11 (Fig. S3.17E).

Layer 9 of grid units L9 and E-G/9-14 featured tabular carbonate crusts of variable thickness and extension, and many of the stone tools therein presented thick carbonate coatings (Fig. S3.18-S3.19). No coatings of similar thickness and extent were observed among the lithics from overlying units but, at comparable elevation (>65-75 cm below ground surface), those units only existed behind the drip-line. Outward, only the portions of layers 7b and 8 extending to rows 9-11 of the grid were buried so deeply, and some of their lithics also feature carbonate coatings (even though thinner and less extensive). The distribution of these precipitates closely following the shelter's extant drip-line, they cannot be relicts of a mid-Upper Pleistocene paleosoil. Instead, they must relate to the calcic horizon of a Holocene Mediterranean soil, denoting how far down water percolated from the ground surface during its formation.

A massive erosional event removed most of layer 9 in the central and western parts of the upper platform, where bedrock was left exposed, bare or covered by a thin layer 80. The geometry of the erosive scar suggests that the implicated agent was water running along the back wall of the site and cascading to S and SW from an area of the upper platform's edge located in columns J-K of the grid. The origin of that water probably lies in the fissure that prolongs the back wall to the East, reactivated as a karst outlet draining the plateau above. As a consequence of these processes, the baseline for subsequent depositional events featured a prominence of the upper bedrock platform that effectively divided the site into two different parts that, for a while, underwent separate depositional histories: FDM-West, where, after a hiatus of unknown duration, layer 9 was overlain by layers 8 and 7b; and FDM-East, where layer 9 was eventually covered by layers 7 and 5 (Fig. S3.17F-I).

The geometry of these stratigraphic interfaces explains why the basal reaches of layers 7b and 6/7 yielded a couple of items that, from technological and typological standpoints, are of Middle Paleolithic affinities (Table S3.3; Fig. S3.20). One (2012-159) is a flint denticulate made on a cortically backed, orange segment-like flake, probably a deliberately overshot discoid blank. It was found in grid unit E9, in an area where layer 7b was in lateral contact with layers 8 and 9 (Figs. S3.12-S3.13). There can be little doubt that this is a reworked object. The other (J6-63) is a slightly patinated sidescraper fragment found at the base of layer 6/7 in a place that, at the time of formation, lied at the foot of a steeply sloping layer 9 long exposed as FDM-East's ground surface (Fig. S3.17E-H). It is clearly an inherited item incorporated by progradation.

Blanks such as that used for the 2012-159 denticulate are well represented among the complete, modified and unmodified flakes from layer 9 — by four items (three sidescrapers and one naturally backed knife) out of 26, i.e., 15% — but absent from the equivalent ensemble of flint blanks from layers 8, 7b and 6/7 (N=122). The J6 sidescraper is made on quartzite, a raw-material rarely found in the site's Upper Paleolithic levels, in which it was exploited in expedient manner, to produce blanks that remained unretouched. That only two such "contaminants" were found among the 210 formal retouched tools found in layers 8, 7b and 6/7 (see below) suggests that the presence of reworked, undiagnostic Middle Paleolithic material in these units is anecdotal and of no consequence for the study of spatial distributions and the lithic economy and lithic technology of the site's Upper Paleolithic occupations.

The subsequent accumulation of layer 6/7 testifies to a change in the source of the sediment, now coming mostly from the opposite side of the recess, and to the onset of the constitution of the site's still extant West-dipping ground surface (Fig. S3.17H). From this time onwards, FDM-West behaved as the brim of a depositional cone in which most sedimentary accumulation occurred eastward of column H. The thickness of layer 5 in the central part of FDM-East (Fig. S3.17I), especially apparent in the L>M cross-section (Fig. S3.8), is a byproduct of this change in the dynamics of the accumulation, combined with the presence of a depression caused by the scouring of the previously formed deposit (Fig. S3.17H) — probably due to a new episode of reactivation of the fissure prolonging the site's back wall to the East. Once ground surface again reached a stable level, the dynamics of the accumulation was reduced, leading to the formation of layer 4 (Fig. S3.17J), and eventually came to a halt. After the hiatus — manifested in the incipient soil formation probably present in unit 4a and during which rabbits extensively burrowed the site — came the last stage of the accumulation, represented by layers 3 and 2 (Fig. S3.17K-L). This phase corresponds to the eventual filling-up of the slightly inward-sloping, basin-shaped space comprised between the back wall and the drip-line extant since layer 4 times.

A number of expectations for the conservation of an archeological record can be derived from this formation process. Firstly, that sedimentation will have been rapid and the remains abandoned on-site quickly buried in packages of significant thickness. Secondly, bearing in mind the average duration of Middle and Upper Paleolithic technocomplexes, that individual layers will correspond to separate phases of the regional chrono-stratigraphic sequence, with minimal palimpsest effects. Thirdly, notwithstanding the localized impact of bioturbation and the potential presence of inherited material, that the level of assemblage integrity will be, in general, high. Fourthly, that the nature of the sedimentary accumulation combined with the site's significant exposure to the elements (despite the presence of an overhang, FDM is more a *pied-de-falaise* than a true rock-shelter) will cause significant syn-depositional scattering of the remains of individual occupation episodes by gravity- and water-driven surface dynamics, generating relatively low-density, spatially homogeneous find distributions. Fifthly, that the preservation of features (e.g., hearths) will be exceptional and limited to those areas most protected from such surface dynamics — close to the back wall and where the slope extant at the time of occupation flattened out.

These expectations, which we developed at the end of the initial testing phase, guided the excavation approach and the methodology subsequently used. Realizing that the potential of the site principally lied in providing a framework for the region's Upper Pleistocene cultural stratigraphy, emphasis was put in the acquisition of lithic assemblages fit for purpose, i.e., sufficiently large (in composition) and complete (in the representation of the different stages of the reduction sequence). For that potential to be realized, however, a large area and a significant volume of sediment would have to be excavated. Hence the adoption of an optimized strategy for the recording of finds, features, surfaces and sections, and the decision to prioritize stratigraphic control over resolution in the spatial distributions (implying, for instance, that the sediment was excavated and sieved by grid units, not by ¼ m² quadrants).

3.6. SPATIAL DISTRIBUTIONS

The spatial distribution of the lithics from layers 9, 8, 7b and 6/7 retrieved between the 2007 and 2014 field seasons is illustrated in Figs. S3.21-S3.24. To avoid distortions caused by different excavators potentially using different cut-off criteria for the recovery of chippage in the sieving process (e.g. when sorting the smaller mesh size), the distributions are assessed by mass instead of number of items. Consequently, for layer 6/7, only flint was considered, as the presence of a few large cores and manuports made on locally available raw-materials (quartzite and limestone) creates distributional peaks that are more apparent than real.

In agreement with site formation expectations, the distributions are fairly homogeneous and, when apparent exceptions are considered in their detail, unimodal. The sorting and classification of sieve finds from layers 5, 4 and 3 has yet to be carried out in full. Based on field observations and the distribution of piece-plotted items, there is no reason, however, to suspect that they will pattern differently.

In layer 9, the higher values tend to be found outward. Grid unit F8 is exceptional because of a large (32.2 g) limestone flake, a raw-material otherwise represented in this layer by a small flake fragment and nothing else. The absence of finds in E-L/4-7 is explained: (a) close to the back wall, by the surface of the site corresponding, at the time, to the upper platform's rock floor; (b) away from the back wall, by erosional loss of the deposit, leading to exposure of the sagittally oriented bedrock prominence found in columns I-K of the grid (Fig. S3.9). Loss to erosion also explains well the marked difference between the total for the grid units where layer 9 remained thick (e.g., L/8-9) and those where erosion truncated its upper reaches.

The layer 8 stone tool distribution is biased by two factors: the low number of finds, which means that peaks will be present in those squares that yielded larger, heavier pieces, namely cores (e.g., F11, where two of them come from); and the highly irregular geometry of the deposit, which implies that mass/area ratios are in this instance a poor indicator of the density of finds per volume unit of excavated sediment. The impact of these factors is clearly apparent when the layer 8 artefact distribution is contrasted with those of layers 7b and 6/7, which yielded larger assemblages and featured a more regular geometry.

Indeed, despite post-depositional, surface dynamics-induced homogenization, the original spatial structure of the Gravettian occupations has remained relatively well preserved. The layer 7b distribution is clearly unimodal and features higher values coincident with the location of the hearth features excavated therein (Figs. S3.25-S3.26). The layer 6/7 distribution is only apparently bimodal, as the trough in H-J/6-7 correlates with (a) the loss to erosion, along row 7, of a significant part of the deposit (Fig. S3.14), and (b) the decreased thickness of the layer in row 6, due to the inward rising of the bedrock (Fig. S3.15). If we filter out the impact of these factors we see a clear concentration in K-M/4-6, coincident with the extent of the black facies ("layer 6"). The concentration is surrounded by a scatter of gradually decreasing density, the apparent (and minor) anomaly represented by O5 being due to the bias introduced by the presence of a large core.

3.7. FEATURES

Figs. S3.25-S3.28 illustrate the few occupation features that could be identified during excavation. All correspond to hearths that underwent erosion to a varying degree. Hearth 1, at the base of layer 4, is the single instance in which a remnant of the original succession of ash, charcoal and burnt sand remained in situ (Fig. S3.28B-E). The reason lies in the cementation of parts of the ash component, within which there were large chunks and even short fragments of carbonized juniper twigs. Hearth 2, in the same stratigraphic position, had been almost completely eroded, and only the presence of a few burnt stones associated with a dark stain, denoting a concentration of micro-charcoal particles, betrayed the presence of a fire feature (Fig. S3.28A); even so, the evidence is weak, and the identification of this feature is tentative. Hearth 3, at the top of layer 6/7, corresponded to a hard patch of cemented ash associated with a scatter of charcoal and reddened stones (Figs. S3.14, S3.27). This cementation explains why Hearth 3 could survive at the edge of the ravine created in the G-K/7 grid units by the washing away of the upper part of layer 6/7. Conversely, the burrowing of the rabbit warren occupying grid units I-J/5-6 and their periphery explains why the preservation of Hearth 1 and Hearth 2 was partial or poor.

While Hearths 1, 2 and 3 correspond to fires lit on a bare ground, Hearths 4 and 5, at the top of layer 7b (Figs. S3.25-S3.26), are of a different type, one that is represented by several intact examples at the nearby La Boja site (see Chapter 4). From these examples we can infer that Hearths 4 and 5 of FDM are the subsurface, stone-filled component of complex features whose above-surface parts — the ash and charcoal produced by the burnt fuel — were lost to erosion. The dark staining of the sediment downslope from, and inwards of Hearth 4 testifies to the process, the retention of micro-charcoal particles being due to the barriers represented by (a) the outcropping of stones that already belong in underlying layer 8, (b) the bedrock prominence that at this stage separated FDM-West from FDM-East, and (c) the escarpment leading to the site's upper platform. The fact that all the charcoal from layer 7b was collected in row 7 (F7, G7 and H7), and most in G7 (i.e., in the grid unit placed in the exact direction leading downslope to NE from the middle of the E-G/8-9 trench), is consistent with this interpretation.

3.8. THE MIDDLE PALEOLITHIC (LAYER 9)

Tables S3.4-S3.5 summarize the composition of the layer 9 stone tool assemblage, and a representative sample is illustrated in Fig. S3.29. Flint accounts for 96% of the finds (87% by mass), and 100% of the cores. The latter's small number (nine) and average mass at discard (18.6 g; 15.0 g considering only the five unbroken ones) imply a type of site occupancy characterized by intensive consumption of mostly imported blanks, as otherwise suggested by the high tool÷blank ratio (0.6 by mass). Supporting this inference, the raw-material available was reduced for the extraction of even minimal amounts of cutting edge, as indicated by (a) the size of the cores' longest measurable removal scars (between 9.7 and 16.4 mm), and (b) small flakes representing 67% of all the unretouched, complete flint blanks.

This economic pattern explains the preponderance of the Kombewa technique (Fig. S3.29, no. 1), well suited for the recycling of flake blanks as cores for the production of smaller flakes. The Levallois and Discoid methods are represented by a few diagnostic blanks (Fig. S3.29, no. 2) and preparation products, as well as by one core. The latter is a recurrent centripetal Levallois core for which, given the known difficulty in distinguishing the two techniques when dealing with exhausted specimens, a discoid classification would also have been reasonable.

Out of the 36 tools, five are typologically unclassifiable, broken pieces. Excluding these, sidescrapers represent 68% of the total. The use-wear evidence (Table S3.6, Fig. S3.30) indicates that they were used indistinctively to work on wood, hide, and hard animal tissue, as well as for cutting meat. This evidence is consistent with the notion that their retouch responds to the need to extend the use-life of a blank's worn-out edges rather than to normative considerations related to intended design. That such edge-rejuvenation tasks were carried out on-site is documented by re-sharpening debris and by extensions of well-defined use-wear polish cut by retouch removals (Fig. S3.30, no. 2).

The other tools are one denticulate, one notched piece, three backed knives (e.g., L9-6; Fig. S3.19), one atypically retouched piece and two Mousterian points. The latter were indeed used for the stone-tipping of projectiles, as shown by their diagnostic wear. Distally, the larger (Fig. S3.29, no. 4; Fig. S3.30, no. 6) features an impact fracture and, proximally, bears characteristic microscopic impact striations. In addition, the blank's butt had been retouched to facilitate hafting through alternating removals that thinned the dorsal edge of the platform and, ventrally, eliminated the bulb. The very tip of the smaller point (Fig. S3.29, no. 3; Fig. S3.30, no. 7) — made on an older, patinated, recycled blank — is missing (possibly due to impact), but its classification as a point is supported by (a) the regularly retouched edges distally converging towards a 45° angle, (b) the overall symmetry of its triangular outline, and (c) the microscopic impact striations present proximally on the ventral side.

The small size of the assemblage suggests infrequent and short-lived stays, while the fact that tools related to on-site, domestic tasks make up most of the assemblage means that such stays were not purely logistical in nature. A residential signal can also be seen in the fact that most phases of the reduction sequence — namely, core-preparation and core-trimming, blank production, tool consumption, re-sharpening — are represented.

Neither unworked nodules nor cores discarded during the initial configuration phase have been recovered. The raw-material acquisition stage of the reduction sequence is therefore missing. This absence concurs with the intensive reduction of flint volumes to suggest that (a) raw-material sources were for the most part located at a significant distance and (b) raw-material procurement was embedded in daily subsistence tasks and/or routine travelling (e.g., when relocating settlement).

3.9. THE EARLY UPPER PALEOLITHIC

3.9.1. Aurignacian (Layer 8)

The inventory of the lithics recovered in layer 8 is given in Table S3.7, and details on the kinds of cores, bladelet blanks and retouched tools are given in Table S3.8. A representative sample is illustrated in Figs. S3.31-S3.32. All cores, blanks and tools are on flint, but quartz and quartzite are represented in the debris category.

As identical recovery techniques were used in the excavation of both layers 8 and 9, the much higher percentage of the assemblage represented by debris in layer 8— 77% of the total, up from 54% in layer 9, the percentages by mass being 30% and 15%, respectively — might indicate that on-site knapping was more frequent than before. Note, however, that the retrospective assignment to layer 9 of the finds made at the base of the M-O/4-6 trench could only be made for the diagnostic material, not for the potentially associated debris. Given the low numbers involved, the bias introduced by this factor is, however, marginal. Therefore, the lower lithics per square meter ratio of layer 9 probably reflects a higher level of syn-depositional scattering, including the downslope washing-away of a larger proportion of the lighter-weight debris component. In this context, the lower overall number of blanks, cores and tools in layer 8 (75, down from 176 in layer 9) could result from the smaller size of the area in which it was found.

The average mass of (unbroken) discarded cores is the same as in the Mousterian, even though all were now used for the extraction of bladelets, mostly using the carinated/nosed “scraper” reduction method (Fig. S3.32, nos. 1-2). This technological change is reflected in the average mass of complete, retouched and unretouched flint blanks (flakes, small flakes, blades and bladelets), which decreases more than twofold, from 5.0 g in the Mousterian to 1.9 g in the Aurignacian. There are also three proximal, unretouched blade blanks and one endscraper made on the proximal end of an overshot laminar blank (Fig. S3.32, no. 3). These data suggest a flint economy no different from that obtaining during the Mousterian, i.e., characterized by intensive consumption of mostly imported blanks with on-site recycling of exhausted volumes (broken or worn-out blades and flakes) to extract small amounts of cutting edge used to retool or replenish the tool-kit. The fact that the tool÷blank ratio (0.8 by mass) is in layer 8 even higher than in layer 9 further supports this conclusion, but there is a significant difference: in layer 8, no cores, core-preparation or core-trimming by-products document the on-site production of non-microlithic blanks.

Functionally, both occupations yielded evidence for the carrying out of the same kinds of tasks, as the activities represented by the layer 8 retouched tools are the same as in layer 9: use-wear documents the mounting of bladelets, including unretouched ones, as elements of composite projectiles (Fig. S3.32, no. 6), while wood- and hide-processing tasks were carried out with scrapers and atypically retouched flakes (Fig. S3.32, no. 3; note how this item's polished surface is abruptly cut by the retouch, documenting on-site re-sharpening of a previously used tool). The differences are technological, relate to the methods used in the reduction of available volumes — now geared towards the production of bladelets — and result in a more efficient exploitation of the raw-material. Typologically, these differences are expressed in the replacement of sidescrapers by endscrapers, and in weapon tips being now armed with series of barbs weighing in the range of 0.2 g each instead of a single, axially fitted, triangular point weighing in the range of 5 to 30 g. However, the spatial segmentation of the reduction sequence, as best seen in the lack of evidence for the on-site production of blades, suggests occupations whose logistic, hunting component overshadowed the residential one.

Despite the small size of the assemblage, there can be no doubt as to its affinities with the Aurignacian; its stratigraphic position — sandwiched between Mousterian and Early Gravettian layers — matches its combination of carinated or nosed "scrapers" with very small bladelets bearing a marginal, mostly inverse or alternate retouch (i.e., of the Dufour type). Specifically, the presence of one nosed "scraper," and the fact that 40% of the 15 bladelet blanks complete or sufficiently complete to assess torsion are twisted (e.g., the retouched specimen of Roc-de-Combe subtype illustrated in Fig. S3.31, or the unretouched bladelet illustrated in Fig. S3.32, no. 4) support assignment to the Evolved (Aurignacian II) rather than the Early (Aurignacian I) phase of the technocomplex.

3.9.2. Early Gravettian (Layer 7b)

The Early Gravettian stone tool inventory (Tables S3.9-S3.10) nearly quadruples that of the Aurignacian in both number and mass. This increase is due neither to a larger amount of debris (73% of the total, about the same as in layer 8) nor to the marginally larger size of the area of the site in which layer 7b was present (see Figs. S3.22-S3.23). All other things having remained equal, these numbers suggest that either layer 7b spans a longer time interval or FDM was now being more frequently occupied.

As before, flint is overwhelmingly dominant (97% by mass), with quartzite being represented by a handful of flakes and some chippage. By mass, however, the average size of unbroken cores (6.1 g) is halved by comparison with both the Mousterian and the Aurignacian, even though the average mass of complete, retouched and unretouched flint blanks remains broadly similar (2.4 g in layer 7b, 1.9 g in layer 8). This pattern correlates with a change from carinated/nosed "scrapers" to "burins" as the preferred type of bladelet core. It may well be the case, therefore, that the emergence of this preference reflects a continuing trend towards increased efficiency in the exploitation of immediately available volumes, in the context of a flint economy that remained characterized by intensive consumption of imported blanks coupled with on-site recycling of exhausted material.

The tool÷blank ratio of layer 7b (0.05 by mass) is more than one order of magnitude below the ratio seen in both the Mousterian and the Aurignacian. This is due to the fact that, three notches and three atypically retouched pieces excepted, this layer's retouched tools are all small, light-weight projectile points: Gravette (see Fig. S3.33) or microgravette points, and backed bladelets (all broken, probably fragments of microgravettes, given the *sur enclume* retouch and impact fractures seen on most). Sampling bias cannot explain the lack of endscrapers because they are present in the layer 8 assemblage, which is significantly smaller. In addition, the functionally equivalent sidescrapers dominate in layer 9. Therefore, even though the economy of flint remained the same, a significant change in functionality would seem to have occurred at the onset of the Gravettian: the shelter would have become a place used in more specialized manner, in relation to hunting activities only and with few to no domestic tasks (e.g., hide-working) being carried out on-site. This inference, which remains to be tested via use-wear analysis of the unretouched material, is consistent with two other observations: the absence of blade cores, core-trimming or core-preparation elements, and the fact that all bladelet cores derive from the recycling of débitage blanks.

The fact that >25% of all bladelet blanks (and 33% of the retouched ones, all transformed in backed micro-points) bear diagnostic marks of having been removed from "burins" corroborates the techno-economic classification of these items as cores. Typologically, the significant feature of layer 7b is the disappearance of the Dufour bladelet, replaced by characteristically Gravettian backed elements. There are two marginally backed bladelets, but one comes from around a small burrow at the base of layer 7b of grid unit E9 that disturbed the underlying Aurignacian in layer 8. This item could well be, therefore, in derived position. However, this microlith type was found through the site's Upper Paleolithic sequence, so no a priori reason exists to question its presence in the layer 7b bladelet tool assemblage.

3.9.3. Middle Gravettian (Layer 6/7)

Tables S3.11-S3.12 provide stone tool counts for layer 6/7. As the percentage of debris is roughly the same as in layers 8 and 7b, including or excluding them does not change the fact that the total number of finds more than triples relative to layer 7b. This increase is broadly proportional to the difference in the size of excavated areas (see Figs. S3.22-S3.23). When only flint is considered, the mass data replicate the pattern, even though including two large quartzite cores biases the mass per square meter ratio. As is also the case with limestone, the use of this locally available raw-material was, however, anecdotal. The logic of flint economics therefore suggests that not much changed in the frequency of site visits.

The average mass (12.4 g) of the unbroken, discarded flint cores increases in layer 6/7 to about the same as in the Mousterian and the Aurignacian. However, the average mass (1.8 g) of complete, retouched and unretouched flint blanks is in line with the values for the earlier Upper Paleolithic occupations of the site, while the tool÷blank ratio (0.1 by mass as well as by number), even though doubling the Early Gravettian value, remains more in line with the latter than with the much higher values seen earlier on.

This aspect of continuity with the Early Gravettian is insufficiently explained by the importance of the microlithic component of the tool-kit, which represents only 33% of the total in layer 6/7, down from 57% in layer 7b. In addition, endscrapers, absent in the latter, are now well represented: among the tools that could be typologically classified they are 14. Adding seven broken ones that were counted as part of the "retouched piece fragment" category, endscrapers amount to nearly a quarter of the retouched tools found in layer 6/7.

Knowing that the average mass of cores is higher than in the Early Gravettian while that of the blanks remained broadly the same, the mass and typology of layer 6/7's retouched tools would seem to point out to one of the following: (a) a type of site occupancy that resulted in more waste and was therefore less stressed by raw-material procurement concerns; or (b) an underestimation of the amount of cutting edge actually used resulting from significant functional use of unretouched blanks. Bearing in mind the amount of debris, byproducts, and lesser-quality blanks discarded in the course of the reduction of a single large volume (for an archeological example of such a complete sequence, see the refitted mottled flint core reduced in Terminal Gravettian layer 2 of Lapa do Anecrial, in Portugal; Zilhão, 1997; Almeida et al., 2007), the former would seem to be the parsimonious hypothesis.

The notion that flint was being less intensively reduced and that more on-site knapping of flint volumes introduced as raw or tested nodules was taking place in layer 6/7 times is consistent with two other observations: (a) the presence of a significant number (27) of core-preparation and core-trimming elements, including platform rejuvenation *tablettes* and crested flakes, blades and bladelets; and (b) the fact that, excluding fragments, prismatic types bearing blade and bladelet removal scars, none of which were found in layer 7b, represent a quarter of all cores retrieved in layer 6/7 and amount to a total mass of 231.0 g (almost as much as the Aurignacian assemblage as a whole).

Taken together, these lines of evidence suggest continuity with the Early Gravettian and preceding periods in the low frequency of site visits, coupled with a shift back to the Mousterian pattern of occupations being of a more residential nature — hence the increase in the number and mass of tool-kit components related to domestic and on-site production tasks, and the representation of all stages of the stone tool reduction sequence. Residency, even if still for short durations, is additionally hinted at by the anthropogenic imprint possibly represented by the black, micro-charcoal staining of the sediment in the area featuring the higher concentration of artefact remains (grid units K-M/4-6, across which the "layer 6" aspect of layer 6/7 could be observed; see Figs. S3.5C, S3.8, S3.14, S3.24, S3.27).

It was adjacent to this area of layer 6/7, in grid units I-J/5-6, that the only substantial faunal remains recovered at the site were found — a cluster of *Equus* sp. teeth, a couple complete, the rest fragmentary. Excluding the few Holocene intrusions present in layers 2 and 3, these teeth amount to 87%, by mass, of the site's 128.7 g-strong assemblage of faunal remains. Their preservation must be related to highly localized chemical conditions: micro-sheltering from run-off coupled with incipient, syn-depositional cementation, the latter due to increased carbonate precipitation in the presence of ash and in proximity to both back wall and bedrock.

The shift in emphasis from the essentially logistic occupations of layer 7b times back to ones of a more residential nature probably explains as well the number of body ornaments found in layer 6/7 (Table S3.13; Fig. S3.34). Indeed, layer 7b yielded no more than two small, sieve-retrieved marine or fluviatile shell artefacts, while a third comes from a large burrow cutting through layers 7, 7b and 8. In contrast, layer 6/7 yielded 16 marine or fluviatile shell finds. The perforations borne by some (mostly gastropods and scaphopods, but also an ochred half-valve of *Pecten jacobaeus*; Fig. S3.34, no. 1) suggest they were used, combined or in isolation, as beads, pendants, necklaces or other items of personal ornamentation requiring suspension. A number of valve fragments from different taxa may correspond to fragments of the same, to cutting tools (as in e.g. the case of *Mytilus* sherds), or to containers.

In agreement with the fact that diagnostic “burin spalls” are one third of all bladelet blanks, either retouched or unretouched (and 57% of the total among backed microliths), “burins” remain the most common type of bladelet core; as in layer 7b (Fig. S3.33), on-truncation types dominate, and Gravette points (represented in layer 6/7 by a single apical fragment with diagnostic impact striations; Fig. S3.35) are outnumbered by microgravettes (Fig. S3.36, no. 3). The domestic tool-kit is mostly made-up of endscrapers, two of which (L6-38 and K4-24) have been examined for use-wear and present typical hide-working polishes (Fig. S3.35). Notched pieces come next, and three continuously retouched blades complete the domestic tool component of the lithic assemblage.

3.10. THE LATER UPPER PALEOLITHIC

3.10.1. Site function

The sieve-collected fraction of the assemblages from layers 2 to 5 remaining to be sorted and analyzed, patterns of site occupancy in post-Gravettian times can only be derived, for the time being, from field observations and typological considerations (Table S3.14). This evidence suggests a pattern of site use similar to that inferred for layer 6/7. For layer 3, however, the completeness of reduction sequences, the number of blade and bladelet cores and of core-preparation and core-trimming elements implies more frequent and lengthier visits.

These inferences are supported by the presence of hearth features in layer 4. No such features were found in the other units of the site’s later Upper Paleolithic sequence, in which, however, the absence probably relates to preservation issues: in layer 5, it can be explained by the higher energy of the syn-depositional environment; in layer 3, by the homogenization of the deposit necessarily deriving from a more intensive use of a more restricted space.

Such preservation factors, however, fail to explain why, above layer 6/7, marine or fluviatile mollusk shell, and hence items of personal ornamentation, are represented by only a single specimen (a scaphopod retrieved in the upper reaches of layer 5 during the 2016 field season). One possibility is that the phenomenon relates to issues of territoriality, e.g., the geographical extent of these periods’ subsistence and exchange catchments. This is most certainly the case with Epimagdalenian layer 3, as a similar absence seems to characterize the intensive Upper Magdalenian occupation of the nearby La Boja rock-shelter.

3.10.2. Upper Solutrean (Layers 5 and 4)

The index fossils leave no doubt as to the Solutrean affinities of layer 5. Laurel-leaf foliates are represented by a broken piece recycled into an endscraper (Fig. S3.36, no. 2), manufacture errors (e.g., the overshot flake that eliminated the opposite edge of a large preform; Fig. S3.36, no. 1), several fragments of unfinished pieces, and a few, characteristic bifacial thinning flakes. The barbed-and-tanged, so-called Parpallò point is also present (Fig. S3.36, no. 4). This combination unambiguously places the assemblage in the Upper Solutrean.

In the eastern part of the excavated area, layer 5 accumulated atop and against the markedly West-dipping slope of layer 6/7. Therefore, in this area, the artefact content of layer 5 could have been impacted by progradation effects. In the central and western parts of the site, the interface with underlying layer 6/7 corresponded to a major erosional hiatus that, in grid units H-K/7 and L/7-9, entailed deep scouring (Figs. S3.14, S3.27). This process left the walls of the thusly formed rill exposed to erosion and, hence, available to contribute laterally derived material to the deposit that eventually filled it up, i.e., to layer 5. The homogeneity of the layer 5 stone tool assemblage therefore needs to be critically examined.

Given the primarily gravity- and/or water-driven nature of the formation processes involved, their impact, if any, on the layer 5 lithics ought to be most apparent among the smaller-sized components: the bladelet tool class, which includes six backed items. All come from columns L, M and N of the grid, i.e., from those areas of the excavation in which the West-dipping of the layer's lower boundary was more pronounced. The "burins" on truncation, which form the overwhelming majority of this core category in Gravettian layers 7b and 6/7 (Tables S3.10, S3.12) and represent >50% of the corresponding typological tool class in layer 5 (Table S3.14), represent a different, potentially technologically problematic component. Of the ten found in layer 5, four come from grid unit L8, 15-55 cm below the elevation of the uneroded edge of layer 6/7 as exposed in grid units K-L/5, and eight come from columns L and M of the grid, east of where the interface between layers 5 and 6/7 flattened out.

If, in order to filter out such potential sources of contamination, we restrict the analysis of the layer 5 assemblage to finds that (a) come from a 2 m-wide band of columns D-L running along the back wall and (b) were made at elevations above the edge of the rill, we end up with no more than 20 items, all from grid units H-I/5-6, J5 and K-L/4-5. This stratigraphically secure assemblage includes five of the seven Solutrean diagnostics in Table S3.14, including the Parpallò point, but only one burin on truncation and one backed bladelet fragment (with *sur enclume* retouch). These two items, however, come from the last spit (A7) into which, in the D-K/4-7 trench, layer 5 was subdivided (the base of this spit therefore corresponding to the décapage of the surface of layer 6/7). Outside the black-stained "layer 6" areas, such décapage was fraught with the difficulties arising out of the gradual nature of the stratigraphic interface. Indeed, the field notes contain warnings to the effect that, in some grid units (namely K5), the exposed surface had undercut the interface by some 2-3 cm. Given this evidence, whether a component of Gravettian tradition was indeed present in the Upper Solutrean must remain an open issue — at least based on the information provided by the excavation of FDM.

Layer 4 yielded no bifacial thinning byproducts, but contained a small number of impact-fractured shouldered point fragments. Three more were recovered in layers 2 and 3: they reflect the significant bioturbation undergone by the upper reaches of the stratigraphic sequence, not a persistence of the type into later periods. The impact-fractured tang of a Parpallò point (J7-18; Fig. S3.36, no. 5) was also recovered in this layer. Interpreting the latter's significance is difficult for the same site formation-related reasons that complicate the interpretation of the layer 5 assemblage: the possibility, in fact the necessity, that progradation processes will have introduced in the sediments making up layer 4 material derived from areas of layer 5 located upslope to the East. This is especially so for the small-sized items, the implication being that we cannot exclude that J7-18 derives from layer 5. Whether Parpallò and shouldered points were used in association during the interval represented by layer 4 is therefore an issue that, at FDM, cannot be resolved with present evidence.

3.10.3. Epimagdalenian (Layers 3 and 2)

The Epimagdalenian affinities of the assemblages from layers 3 and 2 were established by Román et al. (2013), who already provided a summary of the data available until 2010, the last field season during which these units were extensively excavated. The counts in Table S3.14 differ slightly from those given in Román et al.'s Table 1, basically because stricter criteria were followed in separating simply edge-worn from atypically retouched items. These differences have no impact on the fundamentals of Román et al.'s conclusions, which remain unchanged: even though the assemblages could not be dated, their typological structure follows the Spanish Mediterranean-wide pattern and allow assignment of layer 3 to an early phase of the Epimagdalenian, characterized by the significant number of Malaurie points, and of layer 2 to a more advanced phase of the technocomplex, characterized by the introduction of lunates.

3.11. CONCLUSIONS

The excavation of the Finca Doña Martina rock-shelter advanced our knowledge of the Paleolithic archeology of Eastern Spain, with implications beyond regional boundaries. The following is a non-exhaustive selection focusing on key points of ongoing scientific debate:

- The excavated deposit contains a record of human occupation ranging from the Mousterian to the Epimagdalenian, showing that the Mula basin was settled during glacial maxima (the Solutrean) as much as during periods of milder, close-to-present, transitional climatic conditions (the early Epimagdalenian, which radiocarbon dates elsewhere place in the Alleröd, and the Mousterian, which, by stratigraphic position, probably dates to the second half of Marine Isotope Stage 3).
- Given the chronological and paleoclimatic range of the site's culture-stratigraphic sequence, the parsimonious interpretation of its hiatuses is that they relate to geological processes, i.e., that such hiatuses are depositional or erosional in nature, not a reflection of human demography (specifically, that they cannot be interpreted as humans having been driven away from the region for extended periods because of environmental changes determined by the rapidly oscillating climates of the Late Pleistocene).

- When analyzed from the perspective of flint economics, and considering as well the spatial segmentation (or lack thereof) revealed by the representation of the different phases of reduction sequences, the stone tool assemblages suggest that the site was mostly used in residential manner; a focus on hunting-related as opposed to domestic tasks is, however, apparent in the Early Upper Paleolithic (and especially so in Early Gravettian layer 7b), while more frequent visits, and a more intensive use of the space available, can be inferred for the Epimagdalenian.
- Contra recent claims that the Gravettian represents the earliest phase of Eastern and Southern Spain's Upper Paleolithic sequence (de la Peña, 2013), the stratigraphic position, level of assemblage integrity, and techno-typological features of the stone tools recovered in layer 8 corroborate the evidence for the regional presence of the Aurignacian derived from a number of sites in the País Valenciano (e.g., Cova Beneito, Mallaetes) and Andalusia (e.g., Cueva Bajondillo) (Zilhão, 2006).
- The finding in basal layer 5, during the 2016 field season, of a Mediterranean-type backed-and-shouldered blade (i.e., an unfinished point), documents the association of this type with the barbed-and-tanged, Parpallò point. The hypothesis that the emergence of the latter predates the first appearance of shouldered points and defines an initial phase of the Upper Solutrean in the region, as suggested by Tiffagom (2006) and accords with the sequence seen in Portugal, e.g. at Gruta do Caldeirão (Zilhão, 1997), is therefore not supported; but neither can it be excluded, given the significant hiatus that separates layer 5 from the underlying Gravettian deposit.
- If the area-excavation of FDM had been limited to 10-15 m² against the back wall and around the initial test trench we would have failed to detect the Early Gravettian, Aurignacian, and Mousterian occupations, and we would have been unable to carry out a proper analysis of formation process; our results show how, at sites exposed to syn-depositional surface dynamics, bioturbation, and post-depositional disturbance — as is the rule with rock-shelters — the interpretation of stone tool assemblages requires prior assessment of their homogeneity, even though the assemblages are recovered in and/or labelled according to geologically defined units of provenience; proper evaluation of their homogeneity will in turn contribute to a fuller understanding of lateral variation and accumulation dynamics, both of which depend on the excavation of sufficiently extensive areas.

3.12. REFERENCES

ALMEIDA, F.; BRUGAL, J.-Ph.; ZILHÃO, J.; PLISSON, H. (2007) — *An Upper Paleolithic Pompeii: Technology, Subsistence and Paleoethnography at Lapa do Anecrial*, in BICHO, N. (ed.) — «From the Mediterranean basin to the Portuguese Atlantic Shore: Papers in Honor of Anthony Marks. Actas do IV Congresso de Arqueologia Peninsular», Faro, Promontoria Monográfica 07, Universidade do Algarve, p. 119-139.

BRONK RAMSEY, C. (2009) — *Bayesian analysis of radiocarbon dates*. «Radiocarbon», 51(1), p. 337-360.

MORENO-RUEDA, G. (2006) — *Selección de hábitat por dos subespecies de* Iberus gualtieranus *(Gastropoda, Helicidae) en Sierra Elvira (SE de España)*. «Zoologica Baetica», 17, p. 47-58.

PEÑA, P. de la (2013) — *The beginning of the Upper Paleolithic in the Baetic Mountain area (Spain)*. «Quaternary International», 318, p. 69-89.

PLISSON, H. (1985) — *Étude fonctionnelle d'outillages lithiques préhistoriques par l'analyse des micro-usures: recherche méthodologique et archéologique*. Ph.D. dissertation, Université de Paris I (Panthéon-Sorbonne).

REIMER, P. J.; BARD, E.; BAYLISS, A.; BECK, J. W.; BLACKWELL, P. G.; BRONK RAMSEY, C.; BUCK, C. E.; CHENG, H.; EDWARDS, R. L.; FRIEDRICH, M.; GROOTES, P. M.; GUILDERSON, T. P.; HAFLIDASON, H.; HAJDAS, I.; HATTÉ, C.; HEATON, T.J.; HOFFMANN, D. L.; HOGG, A. G.; HUGHEN, K. A.; KAISER, K.F.; KROMER, B.; MANNING, S. W.; NIU, M.; REIMER, R. W.; RICHARDS, D. A.; SCOTT, E. M.; SOUTHON, J. R.; STAFF, R. A.; TURNEY, C. S. M.; VAN DER PLICHT, J. (2013) — *IntCal13 and MARINE13 radiocarbon age calibration curves 0-50000 years calBP*. «Radiocarbon», 55 (4), p. 1869-1887.

ROMÁN, D.; ZILHÃO, J.; MARTÍN-LERMA, I.; VILLAVERDE, V. (2013) — *La ocupación epimagdaleniense del abrigo de la Finca de Doña Martina (Mula, Murcia)*, in DE LA RASILLA, M. (ed.) — «F. Javier Fortea Pérez. *Universitatis Ovetensis Magister*. Estudios en homenaje», Universidad de Oviedo/Ménsula Ediciones, p. 167-178.

SONNEVILLE-BORDES, D.de ; PERROT, J. (1954-1956) — *Lexique typologique du Paléolithique supérieur*. «Bulletin de la Société Préhistorique Française», 51, p. 327-335; 52, p. 76-79; 53, p. 408-412, 547-559.

STUIVER, M.; REIMER, P. J. (1993) — *Extended ^{14}C Data Base and Revised CALIB 3.0 ^{14}C Age Calibration Program*. «Radiocarbon», 35 (1), p. 215-230.

TIFFAGOM, M. (2006) — *De la Pierre à L'Homme. Essai sur une paléoanthropologie solutréenne*, Liège, Études et Recherches Archéologiques de l'Université de Liège 113.

ZILHÃO, J. (1997) — *O Paleolítico Superior da Estremadura portuguesa*, 2 vols., Lisboa, Colibri.

ZILHÃO, J. (2006) — *Chronostratigraphy of the Middle-to-Upper Paleolithic Transition in the Iberian Peninsula*. «Pyrenae», 37, p. 7-84.

ZILHÃO, J.; ANGELUCCI, D.; BADAL, E.; LUCENA, A.; MARTÍN, I.; MARTÍNEZ, S.; VILLAVERDE, V.; ZAPATA, J. (2010) — *Dos abrigos del Paleolítico superior en Rambla Perea (Mula, Murcia)*, in MANGADO, X. (ed.) — «El Paleolítico superior peninsular. Novedades del siglo XXI», Barcelona, Universidad de Barcelona, p. 97-108.

Table S3.1. FDM. Radiocarbon dated samples. All samples are trench-collected fragments of *Juniperus* sp. charcoal

Sample	Spit	x	y	z	Subsample	Lab #	Observations
**Layer 4**							
H6-37	A5base	Hearth 1			Anthraco #51	VERA-6171HS	in the H6NW cemented ash remnant
H6-38	A5base	Hearth 1			Vial 1	VERA-5101a	in the H6NW cemented ash remnant; single 20 mg fragment, normal size branch
					Vial 4	VERA-5101bHS	in the H6NW cemented ash remnant; single 10 mg fragment, normal size branch
**Layer 6/7**							
H6-51	A9	42	57	262	Anthraco #1	VERA-6170HS	parts of a single charcoal piece broken for analysis
H6-63	A10	75	50	265	Vial 3	VERA-5367cHS	single, excavation-broken fragment, normal size branch
					Vial 2	VERA-5367bHS	single fragment, twig size branch
					Vial 4, A	VERA-5367dHS	in 290 mg batch of small fragments, normal size branches
					Vial 4, B	VERA-5367dHS_2	
**Layer 7b**							
G7-43	A13base	36	54	304	Vial 1, A	VERA-5368HS	in 80 mg batch of fragments possibly from a single branch

Table S3.2. FDM. Radiocarbon dating results. The ages have been calibrated against IntCal13 (Reimer et al., 2013) in Calib 7.0.4 (Stuiver and Reimer, 1993); the calibrated ages are given as 95.4% probability intervals

Sample	Lab #	Age BP	Age cal BP	$\delta^{13}C$	Observations
**Layer 4**					
H6-37	VERA-6171HS	18320±100	21906-22417	-19.4±1.8	humic acids
H6-38	VERA-5101a	18690±80	22375-22780	-20.2±2.2	mild ABA (a)
	VERA-5101bHS	19180±90	22842-23435	-23.5±2.0	humic acids
**Layer 6/7**					
H6-51	VERA-6170HS	24450±170	28058-28837	-23.0±1.3	humic acids
H6-63	VERA-5367cHS	23480±150	27408-27864	-23.4±0.6	humic acids
	VERA-5367bHS	23890±170	27654-28354		humic acids
	VERA-5367dHS	24730±170	28378-29188	-19.8±0.6	humic acids
	VERA-5367dHS_2	26610±210	30472-31137		humic acids
**Layer 7b**					
G7-43	VERA-5368HS	26990±220	30765-31312	-23.6±1.4	humic acids

(a) alkaline step (0.01M NaOH) ceased earlier due to risk of complete dissolution; the residue was used for the dating

Table S3.3. FDM Mousterian. Lithics found (in derived or inherited position) within overlying stratigraphic units

Inventory #	Raw-material	Condition	Blank technology	Classification
**In layer 6/7**				
J6-63	quartzite	ridge-worn, slightly patinated	discoid flake (distal)	sidescraper fragment
**In layer 7b**				
2012-159 [E9]	flint	retouch-exhausted	naturally backed flake	denticulate

Table S3.4. FDM Mousterian (layer 9; 2007-2014 field seasons). Stone tool technological categories. The diagnostic Mousterian material found in derived/inherited position within overlying stratigraphic units and a quartzite manuport are not included. N = number, M = mass in grams

RAW-MATERIAL	CORES		FLAKE BLANKS						LAMINARY BLANKS				DEBRIS				TOOLS		TOTAL	
			Complete		Fragment		Small		Blade		Bladelet		Chippage		Chunk					
	N	M	N	M	N	M	N	M	N	M	N	M	N	M	N	M	N	M	N	M
**Flint**	9	167.6	15	148	76	154.0	31	39.0	1	2.4	–	–	174	50.8	31	93.0	34	206.2	371	861.0
**Quartzite**	–	–	3	27.5	3	8.8	–	–	–	–	–	–	6	6.2	–	–	2	53.5	14	96.0
**Limestone**	–	–	1	32.2	1	1.7	–	–	–	–	–	–	–	–	–	–	–	–	2	33.8
**Quartz**	–	–	–	–	–	–	–	–	–	–	–	–	–	–	–	–	–	–	–	–
**TOTAL**	9	167.6	19	207.7	80	164.5	31	39.0	1	2.4	–	–	180	57.0	31	93.0	36	259.7	387	990.8

Table S3.5. FDM Mousterian (layer 9; 2007-2014 field seasons). Classification of cores and retouched tools. The diagnostic Mousterian material found in derived/inherited position within overlying stratigraphic units is not included

Cores	N
polyhedral	1
Kombewa	2
Levallois	2
discoid (broken)	1
fragment	3
**TOTAL**	**9**

Formal retouched tools	N
Mousterian point	2
notched piece	2
denticulate	1
sidescraper	
unilateral	10
transversal	1
convergent	1
denticulated	3
double	2
fragment	4
atypically retouched piece	2
naturally backed knife	3
retouched piece fragment	5
**TOTAL**	**36**

Table S3.6. FDM. Mousterian and Aurignacian use-wear evidence (2007-2014 field seasons; flint). Material used on, inferred function, and traces of residue present

	Illegible	None	Wood	Hide	Meat	Bone	Projectile	Ochred	Total
**MOUSTERIAN**									
sidescrapers	4	3	2	2	3	1	–	1	**16**
denticulates (a)	2	–	–	–	–	–	–	–	**2**
notches	–	1	–	–	–	–	–	–	**1**
points	–	–	–	–	–	–	2	–	**2**
**TOTAL**	**6**	**4**	**2**	**2**	**3**	**1**	**2**	**1**	**21**
**AURIGNACIAN**									
endscrapers	2	–	–	2	–	–	–	–	**4**
notches	1	1	–	–	–	–	–	–	**2**
atypically retouched or broken	2	1	1	–	–	–	–	–	**4**
bladelet tools	–	2	–	–	–	–	2	–	**4**
**TOTAL**	**5**	**4**	**1**	**2**	**–**	**–**	**2**	**–**	**14**

(a) includes one found in derived position at the base of layer 7b

Table S3.7. FDM Aurignacian (layer 8; 2007-2014 field seasons). Stone tool technological categories.
N = number, M = mass in grams

RAW-MATERIAL	CORES		FLAKE BLANKS						LAMINARY BLANKS				DEBRIS				TOOLS		TOTAL	
			Complete		Fragment		Small		Blade		Bladelet		Chippage		Chunk					
	N	M	N	M	N	M	N	M	N	M	N	M	N	M	N	M	N	M	N	M
**Flint**	7	69.0	4	18.1	16	23.8	15	12.1	3	2.8	15	3.6	246	53.4	4	22.5	15	49.1	325	254.3
**Quartzite**	–	–	–	–	–	–	–	–	–	–	–	–	2	0.4	–	–	–	–	2	0.4
**Limestone**	–	–	–	–	–	–	–	–	–	–	–	–	–	–	–	–	–	–	–	–
**Quartz**	–	–	–	–	–	–	–	–	–	–	–	–	1	0.3	–	–	–	–	1	0.3
**TOTAL**	7	69.0	4	18.1	16	23.8	15	12.1	3	2.8	15	3.6	249	54.0	4	22.5	15	49.1	328	255.0

Table S3.8. FDM Aurignacian (layer 8; 2007-2014 field seasons). Classification of cores, retouched tools and bladelets. Bladelet counts include both retouched and unretouched blanks

Cores	N
carinated "scraper"	2
nosed "scraper"	1
prismatic for blades	1
prismatic for bladelets	3
**TOTAL**	**7**

Bladelets extracted from	N
carinated/nosed "scraper"	9
"burin"	4
splintered piece/bipolar core	1
other	7
**TOTAL**	**21**

Retouched tools	N
endscraper on retouched piece	1
endscraper on flake	1
broken endscraper	2
notched piece	1
Dufour bladelet	4
marginally backed bladelet	1
irregularly retouched bladelet	1
atypically retouched piece	2
retouched piece fragment	2
**TOTAL**	**15**

Table S3.9. FDM Early Gravettian (layer 7b; 2007-2014 field seasons). Stone tool technological categories.
N = number, M = mass in grams). Counts include the diagnostic items found in the E-G/8 bioturbation feature and exclude a Solutrean piece from a smaller burrow and an inherited Mousterian piece from the base of the layer

RAW-MATERIAL	CORES		FLAKE BLANKS						LAMINARY BLANKS				DEBRIS				TOOLS		TOTAL	
			Complete		Fragment		Small		Blade		Bladelet		Chippage		Chunk					
	N	M	N	M	N	M	N	M	N	M	N	M	N	M	N	M	N	M	N	M
**Flint**	9	46.1	18	95.1	146	174.3	100	109.1	5	6.1	39	14.3	877	200.6	63	157.0	21	21.9	1278	824.4
**Quartzite**	–	–	3	13.2	4	9.1	2	2.4	–	–	–	–	4	2.3	–	–	–	–	13	26.8
**Limestone**	–	–	–	–	–	–	–	–	–	–	–	–	–	–	–	–	–	–	–	–
**Quartz**	–	–	–	–	–	–	–	–	–	–	–	–	2	0.1	–	–	–	–	2	0.1
**TOTAL**	9	46.1	21	108.2	150	183.4	102	111.4	5	6.1	39	14.3	883	203.1	63	157.0	21	21.9	1293	851.4

Table S3.10. FDM Early Gravettian (layer 7b; 2007-2014 field seasons). Classification of cores, retouched tools and bladelets. Bladelet counts include both retouched and unretouched blanks

Cores	N
"burin"	7
splintered piece/bipolar core	1
fragment	1
**TOTAL**	**9**

Bladelets extracted from	N
"burin"	13
other	38
**TOTAL**	**51**

Burin types	N
on truncation	
oblique	2
concave	1
convex	1
multiple	1
transversal on retouch	1
Noailles	1
**TOTAL**	**7**

Retouched tools	N
Gravette point	2
microgravette	3
notched piece	3
backed bladelet	7
marginally backed bladelet	2
atypically retouched piece	2
retouched piece fragment	1
pointed blade	1
**TOTAL**	**21**

Table S3.11. FDM Middle Gravettian (layer 6/7; 2007-2014 field seasons). Stone tool technological categories. N = number, M = mass in grams. The content of the heavily bioturbated grid units E-F/5-6 is excluded; a limestone cobble used as a hammerstone and four quartzite thermoclasts are not counted

RAW-MATERIAL	CORES		FLAKE BLANKS Complete		FLAKE BLANKS Fragment		FLAKE BLANKS Small		LAMINARY BLANKS Blade		LAMINARY BLANKS Bladelet		DEBRIS Chippage		DEBRIS Chunk		TOOLS		TOTAL	
	**N**	**M**	**N**	**M**	**N**	**M**	**N**	**M**	**N**	**M**	**N**	**M**	**N**	**M**	**N**	**M**	**N**	**M**	**N**	**M**
**Flint**	75	824.3	62	345.1	337	6660.0	241	284.8	40	119.3	223	170.0	2495	686.6	170	397.7	91	214.5	3734	3702.2
**Quartzite**	2	661.0	7	111.2	22	101.2	4	7.8	–	–	–	–	12	5.8	2	28.1	–	–	49	915.1
**Limestone**	–	–	1	4.6	3	12.1	–	–	–	–	–	–	–	–	–	–	–	–	4	16.6
**Quartz**	–	–	–	–	–	–	–	–	–	–	–	–	–	–	–	–	–	–	–	–
**TOTAL**	77	1485.3	70	460.8	362	773.2	245	292.6	40	119.3	223	170.0	2507	692.4	172	425.8	91	214.5	3787	4633.9

Table S3.12. FDM Middle Gravettian (layer 6/7; 2007-2014 field seasons). Classification of cores, retouched tools and bladelets. Bladelet counts include both retouched and unretouched blanks; one diagnostic item found in derived position in layer 3 is included

**Cores**	**N**
"burin"	31
splintered piece/bipolar core	7
nodule	1
polyhedral	1
centripetal	1
Kombewa	2
prismatic for blades	2
prismatic for bladelets	13
prismatic for flakes	6
core fragments	13
**TOTAL**	**77**

**Bladelets extracted from**	**N**
carinated/nosed "scraper" (?)	2
"burin"	86
splintered piece/bipolar core	1
other	170
**TOTAL**	**269**

**Burin types**	**N**
endscraper-burin	1
dihedral	
déjeté	1
on angle	2
on break	2
on truncation	
straight	3
oblique	7
concave	2
multiple	4
multiple mixed	1
Noailles	6
plan	2
**TOTAL**	**31**

**Retouched tools**	**N**
simple endscraper	6
atypical endscraper	3
ogival endscraper	2
endscraper on retouched blank	2
endscraper on flake	1
Gravette/Vachons points	2
microgravette	7
truncated piece	2
continuously retouched blade	3
notched piece	13
trapeze	1
truncated bladelet	3
backed bladelet	7
notched bladelet	7
marginally backed bladelet	3
atypically retouched piece	11
retouched piece fragment	15
pointed blade	2
pointed bladelet	2
**TOTAL**	**92**

Table S3.13. FDM Gravettian. Marine and fluviatile shell finds (2007-2014 field seasons). In all of the perforated gastropods, the perforation is located on the body whorl of the dorsum (a)

Ornaments (perforated)

**#**	**Taxon**	**Perforation origin**
G6-27	*Dentalium vulgare*	natural (tube segment)
G7-23	*Nassarius incrassatus*	percussion
I5-30	*Littorina obtusata*	percussion
J5-62	*Littorina obtusata*	percussion
J7-27	*Pecten jacobaeus*	undetermined
M6-21	*Cyclope neritea*	percussion
O4-6	*Trivia* sp.	percussion
F7-13	*Dentalium vulgare*	natural (tube segment)

(a) F7-13 and 2012-111 are from layer 7b, and 2012-148 comes from a large burrow cutting through layers 7, 7b and 8; all the others are from layer 6/7

Tools, containers, manuports? (unperforated and/or fragments)

**#**	**Taxon**	**Description**
I4-7	*Pecten maximus*	small rib fragment of large valve
I7-34	*Cerastoderma* sp.	small, ochred valve fragment
J5-48	undetermined	small fragment of nacre
J5-49/K5-71	*Mytilus* sp.	two small, conjoining valve fragments
K6-42	*Acteon tornatilis*	unperforated
L5-40	*Pecten maximus*	ventral edge fragment of right valve
L5-72	Pecten maximus	large ventral edge fragment of right valve
L6-53	*Theodoxus fluviatilis*	unperforated
O4-8	*Pecten* sp.	small fragment of rib
2012-111	*Glycymeris insubrica*	ventral margin fragment of <25 mm-long valve
2012-148	*Pecten maximus*	ventral edge fragment of right valve

Table S3.14. FDM Later Upper Paleolithic. Standard typological classification of stone tools (2007-2014 field seasons). Following the type-list of Sonneville-Bordes and Perrot (1954-56) with the modifications introduced by Zilhão (1997)

		Layers			
**#**	**Type**	**2**	**3**	**4**	**5**
	**ENDSCRAPERS**				
1a	simple on blade	4	8	2	3
1b	simple on flake	1	1	–	2
2a	atypical, simple on blade	–	1	1	–
2b	atypical, simple on flake	1	2	3	–
3	double	–	4	–	–
4	ogival	3	–	–	3
5a	on retouched blade	2	4	4	2
5b	on retouched flake	–	2	1	–
6b	on laurel-leaf fragment	–	–	–	1
8	on flake	1	1	–	–
10	thumbnail	5	6	–	–
13	nosed	–	–	–	1
14a	flat-nosed	–	1	–	–
	**COMPOSITE TOOLS**				
17	endscraper-burin	1	2	–	–
18	endscraper-truncation	–	1	–	–
	**PERFORATORS**				
23	perforator	–	**1**	–	–
	**BURINS**				
27	dihedral straight	–	–	–	1
28	dihedral déjeté	2	1	1	3
29	dihedral on angle	–	2	1	4
30a	angle on break	1	1	1	–
31	multiple dihedral	–	2	1	–
34	on straight truncation	–	–	–	2
35	on oblique truncation	1	1	–	–
36	on concave truncation	–	–	–	4
37	on convex truncation	–	1	–	1
38	transverse	–	1	–	1
40	multiple on truncation	–	1	2	1
41	multiple mixed	–	–	1	1
44a	plan	–	1	–	–
	**BACKED TOOLS**				
51a	microgravette	–	–	–	1
51b	unilateral micro-point	–	3	1	–
51c	unilateral micro-point fragment	2	1	–	–
51d	fragment of retouched-base micro-point	1	1	–	1
51e	bilateral micro-point	1	–	–	–
58	backed blade fragment	1	–	–	–

		Layers			
**#**	**Type**	**2**	**3**	**4**	**5**
	**RETOUCHED BLADES**				
65	unilaterally retouched blade	–	–	1	–
66	bilaterally retouched blade	1	–	1	–
	**SOLUTREAN TOOLS**				
70n	fragment of bifacial foliate	–	–	–	6
72a	fragment of shouldered point	–	–	7	–
72b	Parpallò point	–	–	1	1
	**SUBSTRATE**				
74	notched piece	1	4	4	8
75	denticulate	–	1	–	–
76	splintered piece	–	2	1	3
	**BLADELET TOOLS**				
83	segment	2	–	–	–
84	truncated	–	–	2	2
88	denticulated	–	–	1	–
89	notched	–	2	–	1
85a	backed	3	10	2	1
85c	partially backed	–	–	1	–
85f	backed, fragment	29	60	19	5
86a	truncated-backed	–	–	1	–
87a	denticulated-backed	–	–	1	–
90a	Dufour	–	1	4	–
90b	Areeiro	1	1	–	–
90c	marginally backed	–	–	9	6
91b	Malaurie point	2	9	–	–
91d	fusiform point	–	–	1	–
	**VARIA**				
92a	atypically retouched piece	–	10	–	1
92b	retouched piece fragment	5	7	13	8
92c	pointed blade	–	–	1	1
92d	pointed bladelet	–	–	–	–
	**TOTAL**	71	157	90	75

- the layer 2 counts subsume the surface and layer 1 material
- type 70n includes one layer 5 item found in a burrow in layer 7b
- type 72a includes three layer 4 items found in derived position in overlying layers 2 and 3
- type 91b includes one layer 3 item found in the large rabbit warren of grid units I-J/5-6 and their periphery

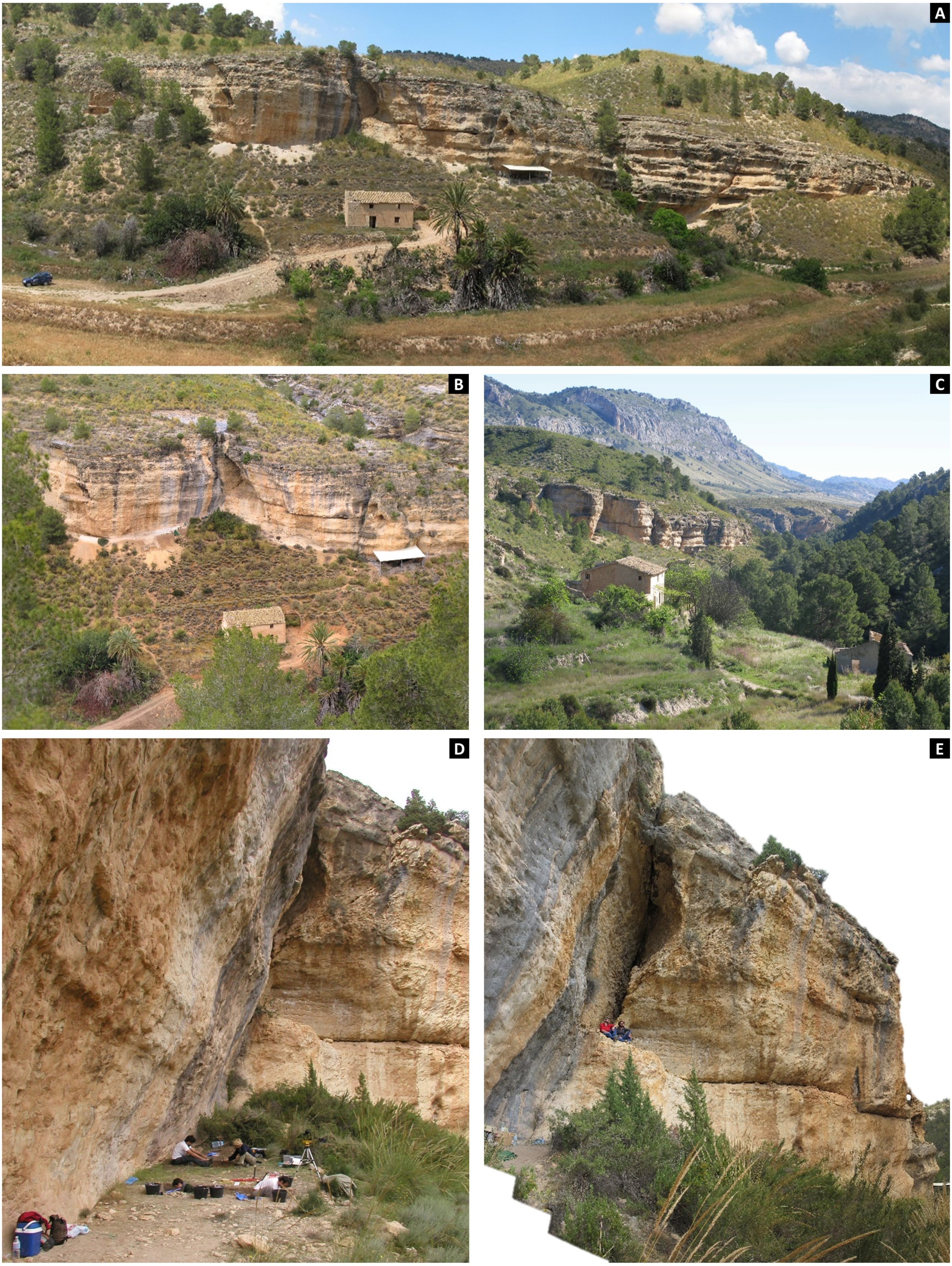

Fig. S3.1. Finca Doña Martina. Site setting. A-B. Panoramic view (April 23, 2013) and overview (April 26, 2013) of the Rambla Perea rock-shelters from the opposite side of the gorge. **C**. The middle section of the Rambla Perea from upstream (April 17, 2009). **D.** The surface of the FDM rock-shelter at the time of initial testing (April 27, 2007). **E.** The fissure prolonging eastward the exposed joint surface forming the back wall of the FDM rock-shelter (April 23, 2013).

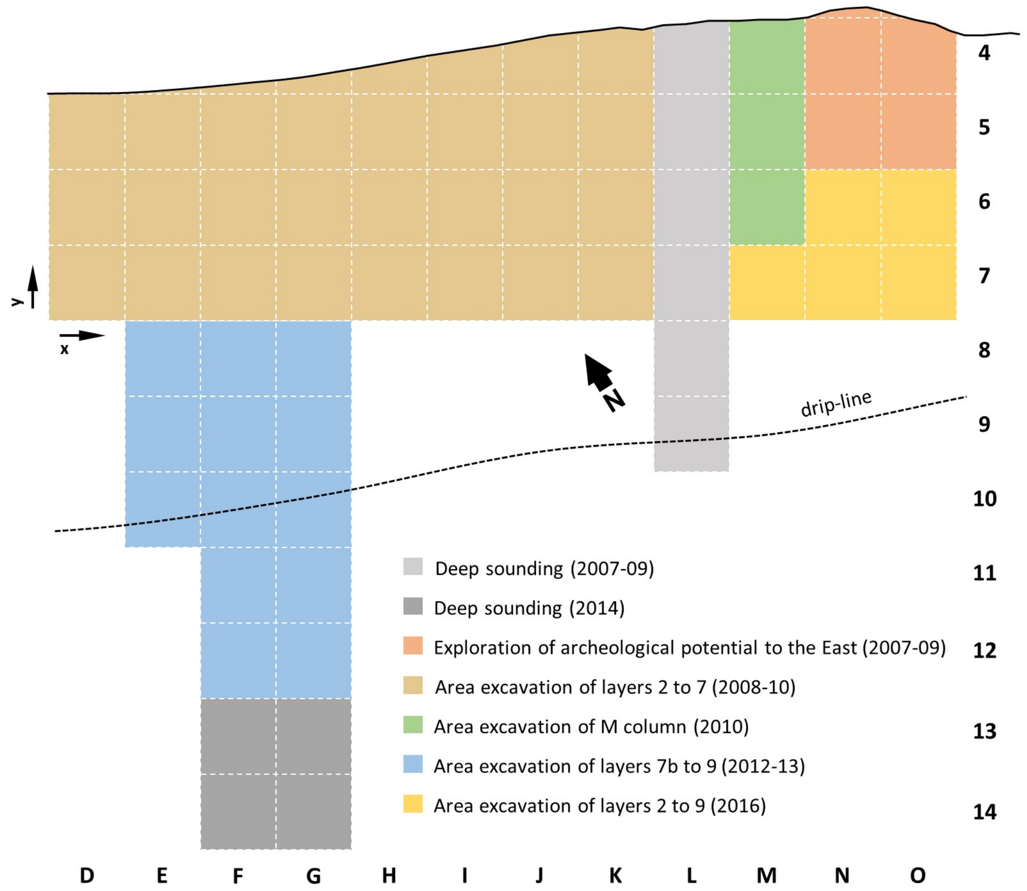

Fig. S3.2. Finca Doña Martina. Excavation grid and outline of the excavation's progress between the 2007 and 2016 field seasons; no field work was carried out in 2011 and 2015.

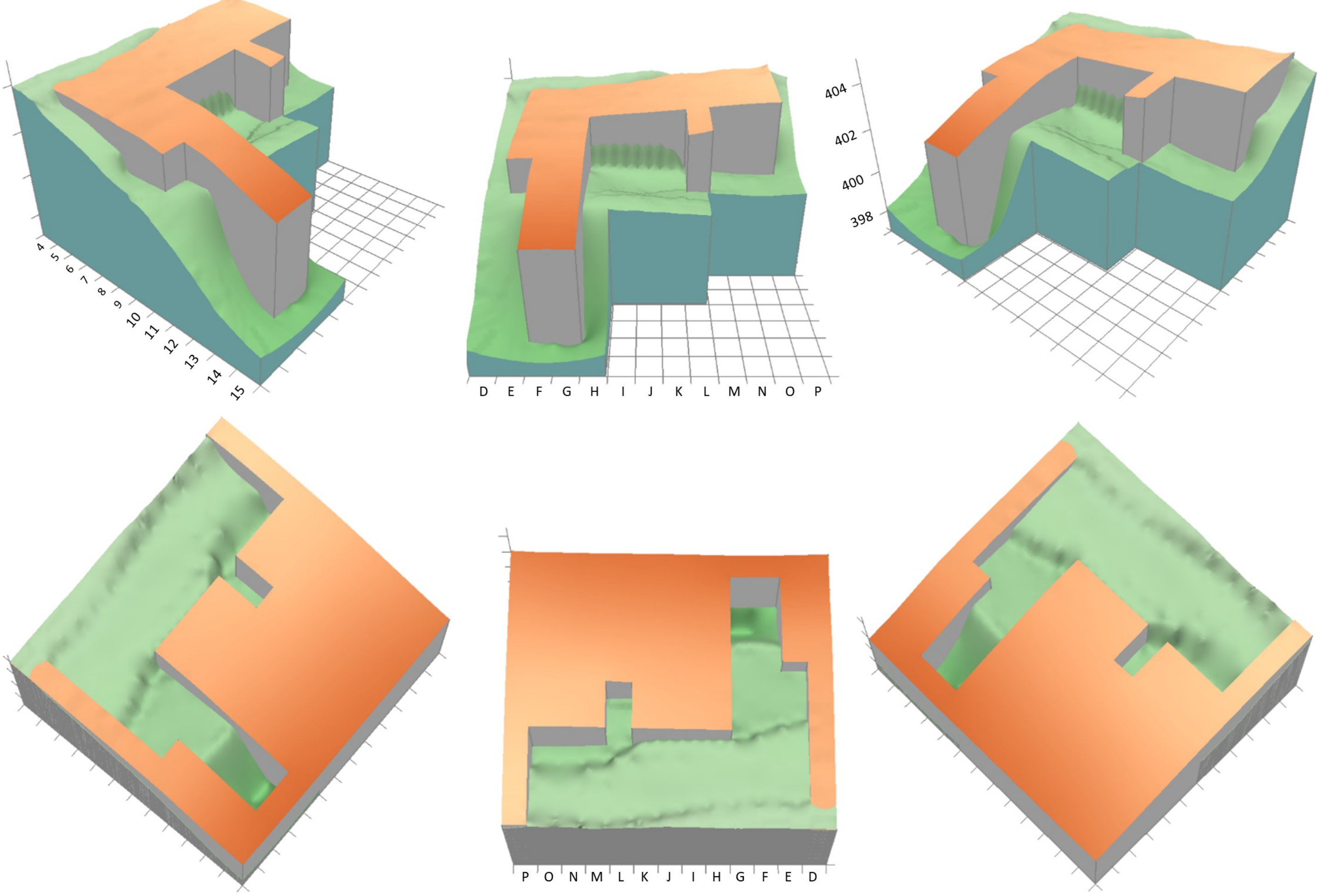

Fig. S3.3. Finca Doña Martina 3D excavation model. Top. The excavation trench rendered as a positive of the excavated volume. **Bottom.** Bedrock as exposed at the base of the trench. Elevations are in m asl. In grid units D/4-6, the shallow and disturbed deposit was excavated down to a stepping platform at the elevation of layer 6/7, thenceforth used for easy access to the trench; therefore, here, said units have not been considered part of the excavation. In grid units M-O/7, excavation stopped at the elevation of layer 10; here, the elevation of the underlying bedrock has been modelled after data from adjacent units.

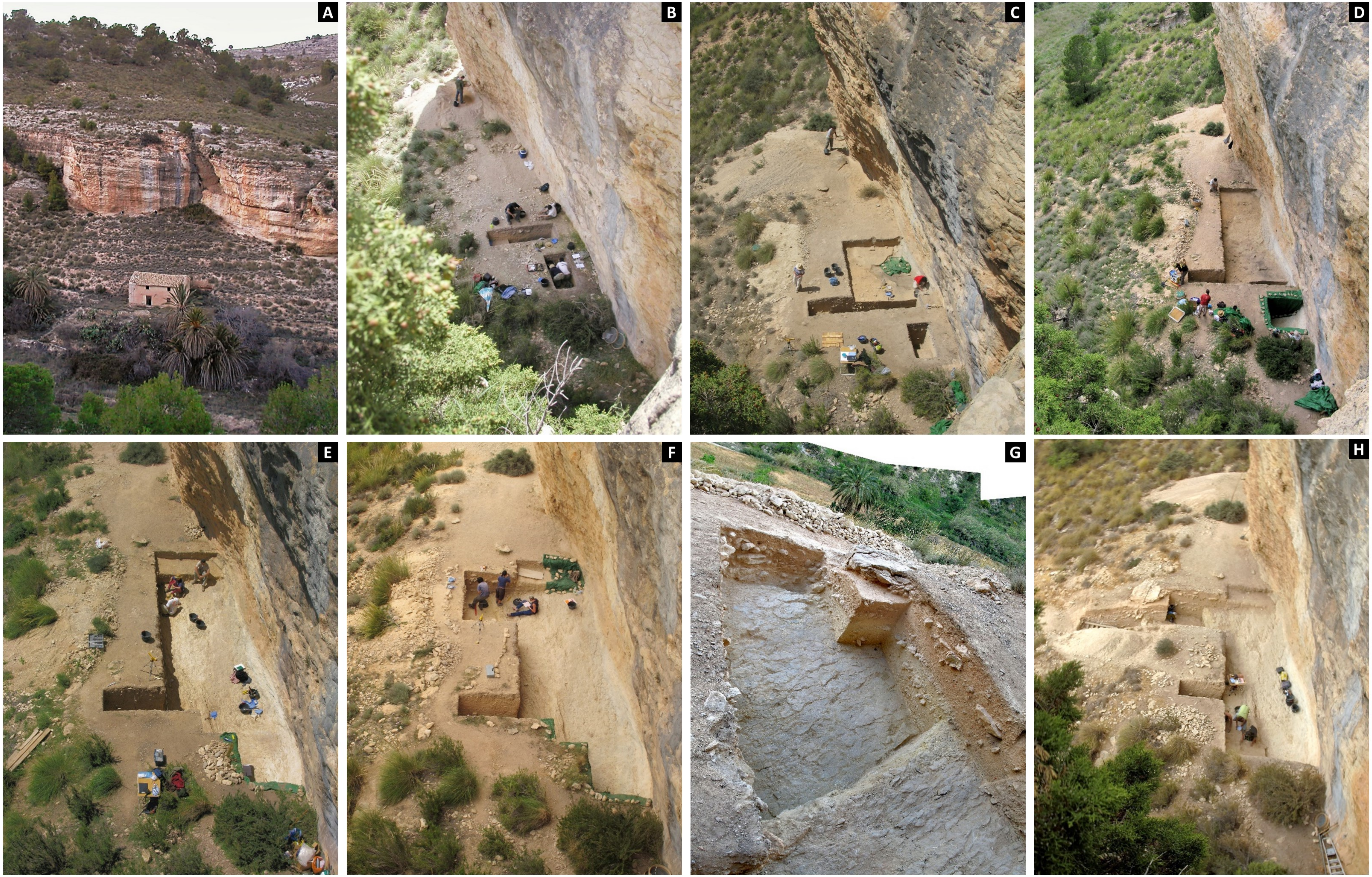

Fig. S3.4. Finca Doña Martina. The excavation's progress. A. The site on the day of discovery, prior to excavation (December 11, 2005). **B.** At the end of the 2007 field season (May 6). **C.** At the end of the 2008 field season (April 12). **D.** At the end of the 2009 field season (April 17). **E.** At the end of the 2010 field season (April 2). F. At the end of the 2012 field season (April 27). **G.** The western part of the trench at the end of the 2013 field season (April 24). **H.** Towards the end of the 2016 field season (September 23).

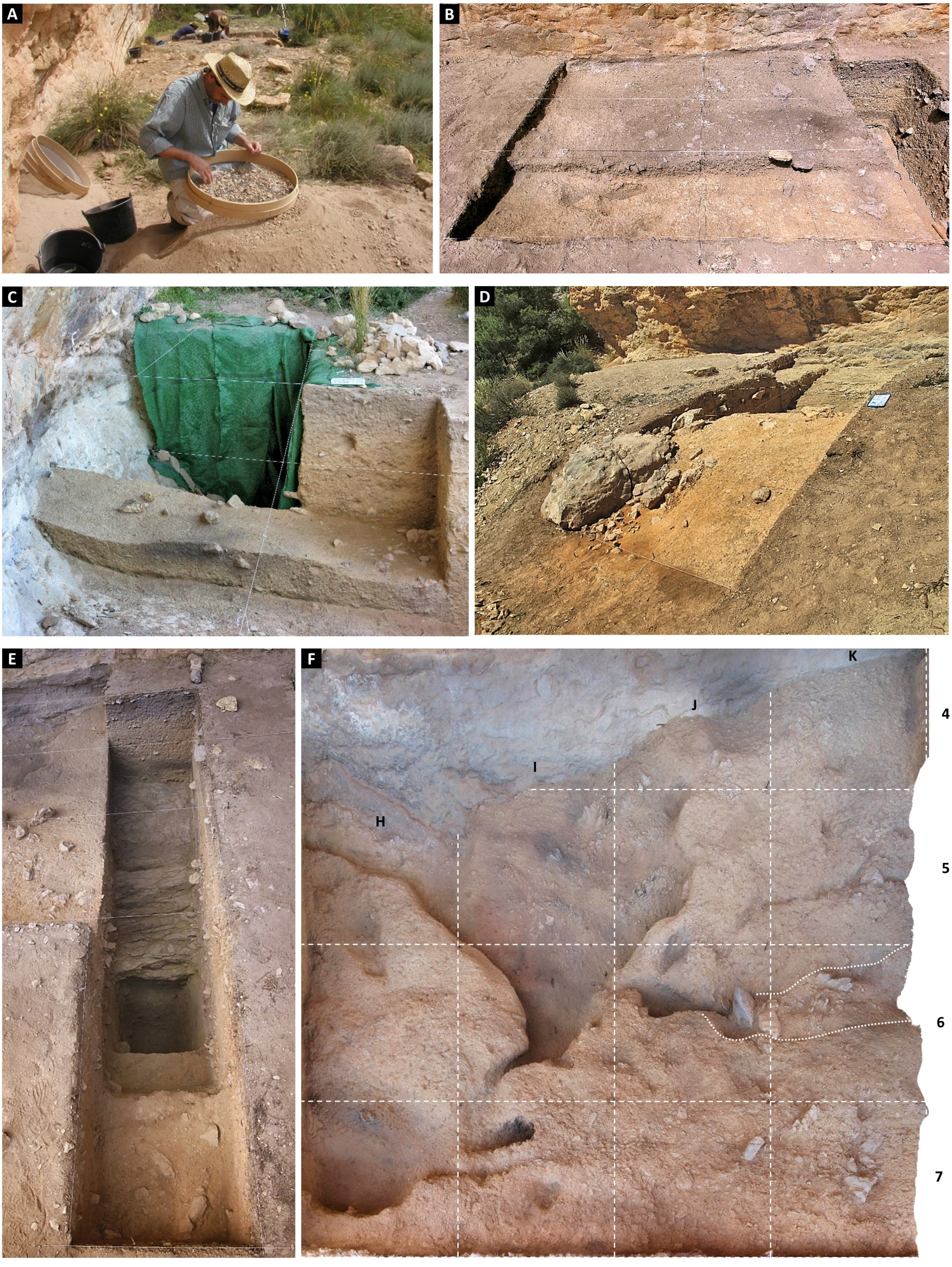

Fig. S3.5. Finca Doña Martina. Excavation approach. A. Sieving. **B-D.** Décapage along natural stratigraphic boundaries of: layers 3 in H-K/4-6 and 4 in H-K/7; layer 6/7 in M/4-6; and layer 7 in E-G/10-12. **E.** Deep sounding to bedrock (L row). **F.** The rabbit warren between the exposed surfaces of layers 4 (the high ground) and 6/7 (the low ground in the center) after the emptying of its fill; the dotted lines indicate a burrow tunnel that, due to the large vertical blocks present, was excavated at a later time.

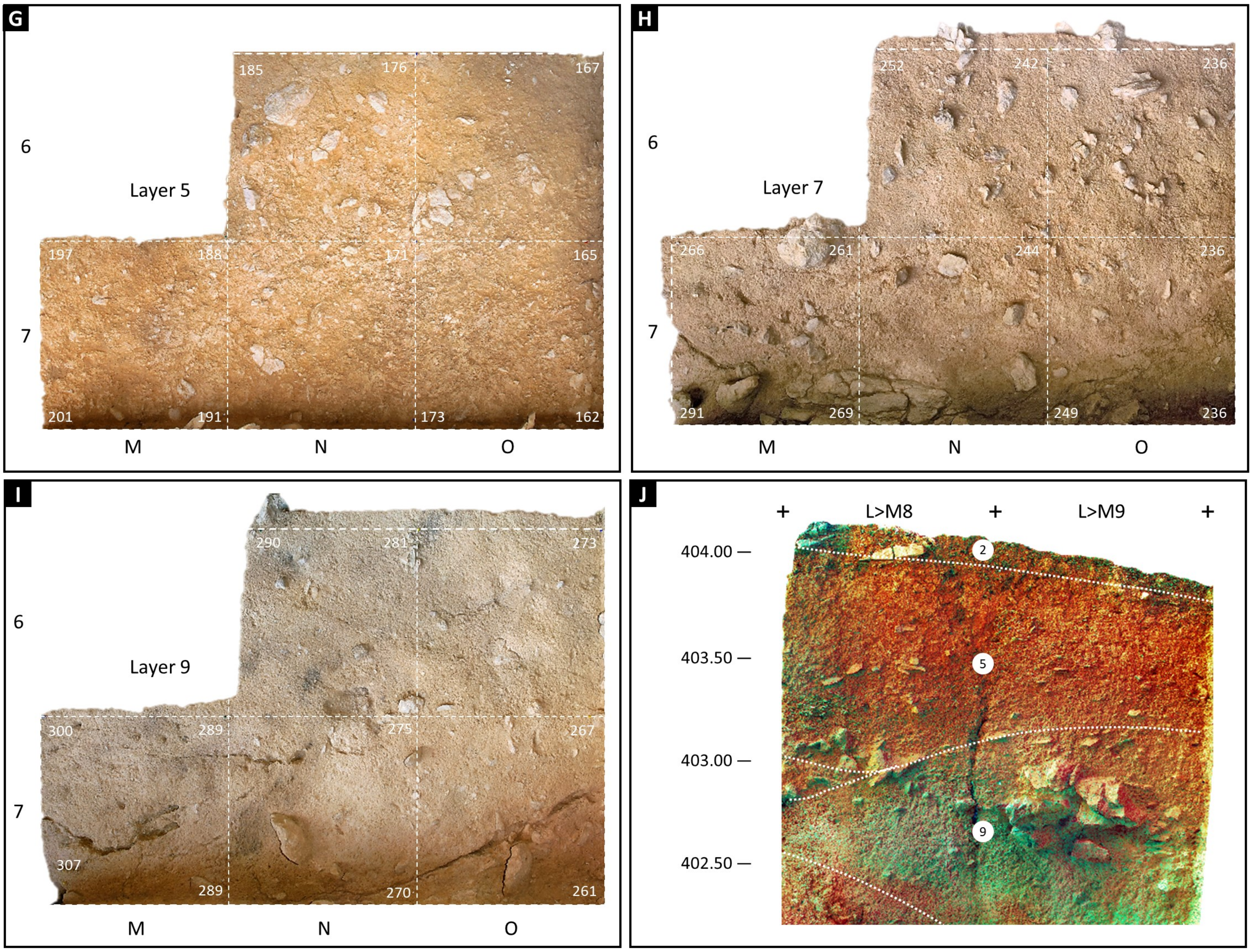

Fig. S3.5 (cont.). Finca Doña Martina. Excavation approach. G-I. Area excavation and décapage along natural stratigraphic boundaries of layers 5, 7 and 9. **J.** Orthophoto of the L>M8-9 cross-section (see Fig. S3.8), DStretched (lrd treatment) to highlight the color contrast between layers 5 and 9. Note the (micro-tectonic?) cracking of the deposit, especially apparent in layer 9.

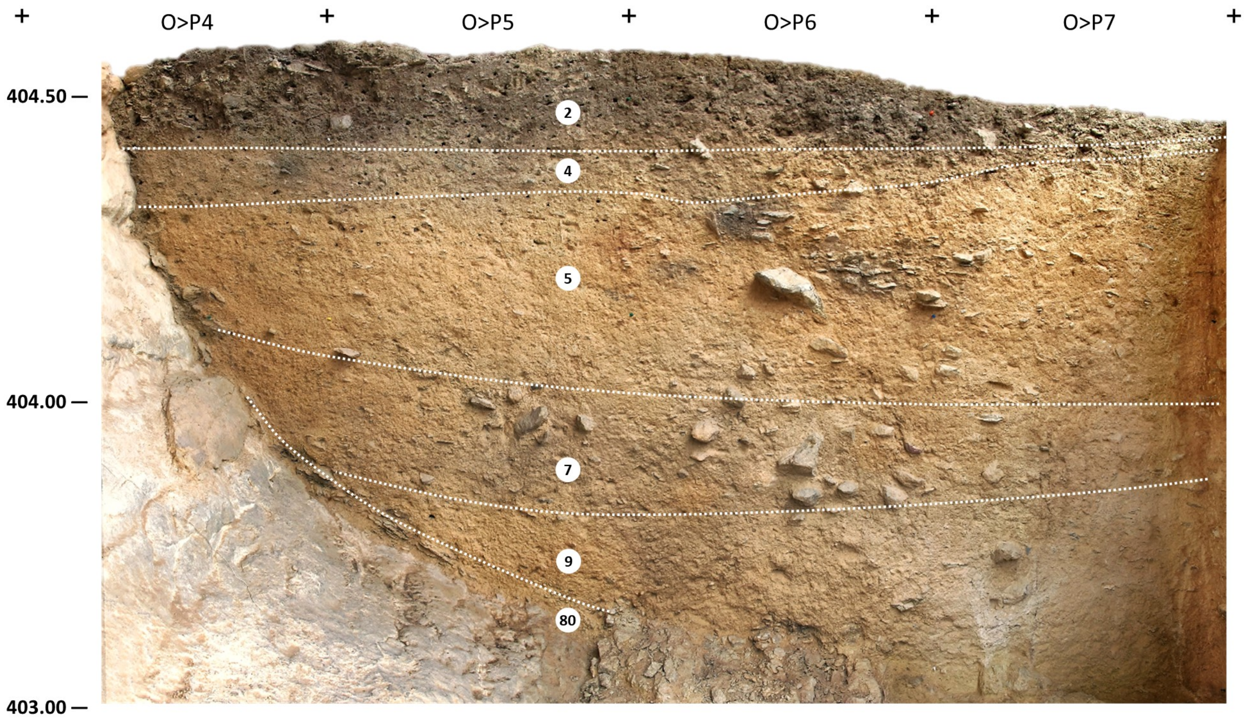

Fig. S3.6. Finca Doña Martina. The O>P stratigraphic cross-section. Orthorectified photomosaic assembled from images taken at the end of the 2016 field season. Elevations are in m asl. The upper part of the section, exposed since 2008, was since burrowed into by wasps. Layer 3 did not extend into the eastern half of the O column. Note, in O>P6, the large disturbance pocket in layer 9.

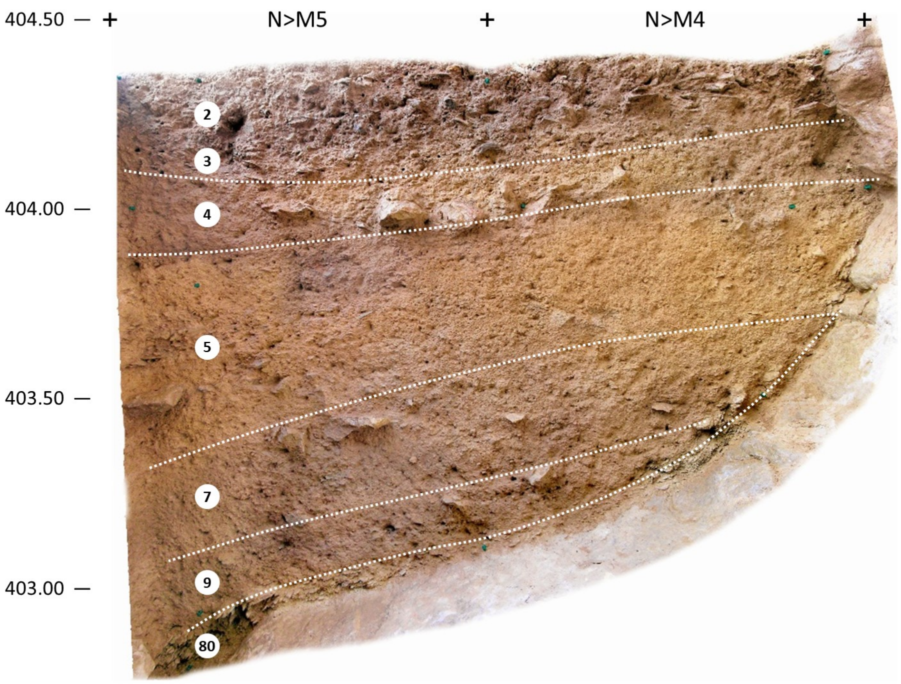

Fig. S3.7. Finca Doña Martina. The N>M stratigraphic cross-section. Orthorectified photomosaic assembled from images taken at the end of the 2009 field season. Elevations are in m asl. Note, in N>M5, the disturbance pockets found at the interfaces between layers 4 and 5 and between layers 7 and 9.

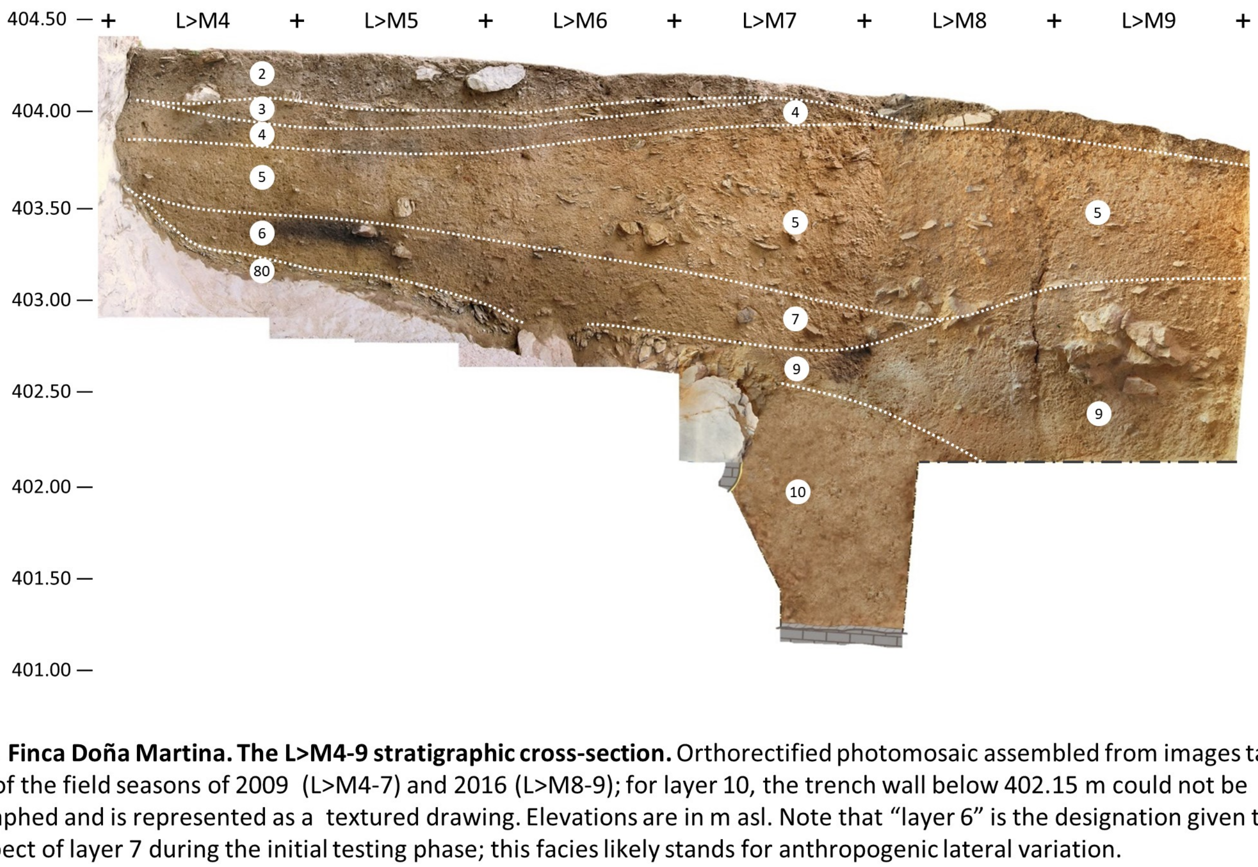

Fig. S3.8. Finca Doña Martina. The L>M4-9 stratigraphic cross-section. Orthorectified photomosaic assembled from images taken at the end of the field seasons of 2009 (L>M4-7) and 2016 (L>M8-9); for layer 10, the trench wall below 402.15 m could not be photographed and is represented as a textured drawing. Elevations are in m asl. Note that "layer 6" is the designation given to the black aspect of layer 7 during the initial testing phase; this facies likely stands for anthropogenic lateral variation.

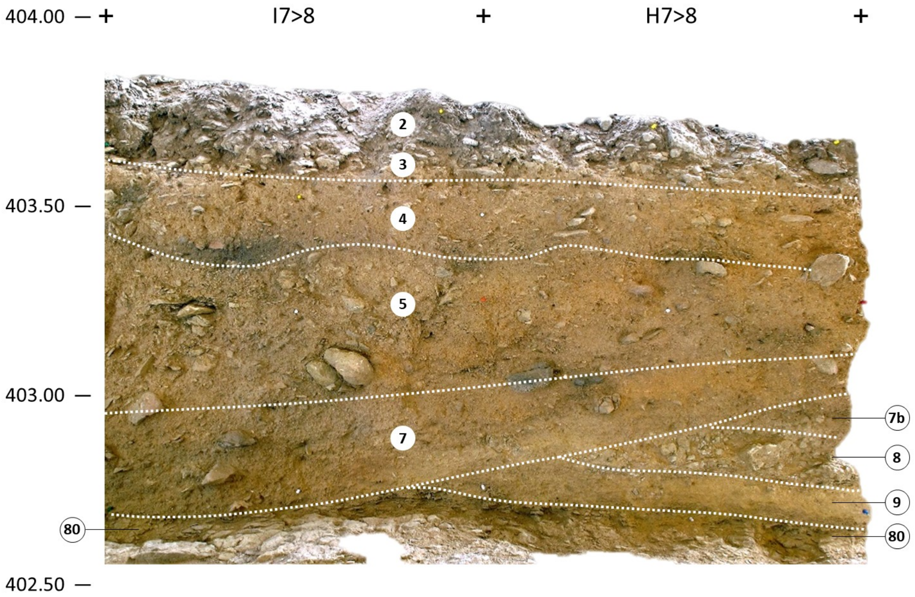

Fig. S3.9. Finca Doña Martina. The 7>8 stratigraphic cross-section. Orthorectified photomosaic assembled from images taken at the beginning of the 2012 field season. Elevations are in m asl. The dark patches observed in the body of layers 4 and 5 are post-depositional accumulations of organic matter related to animal burrowing.

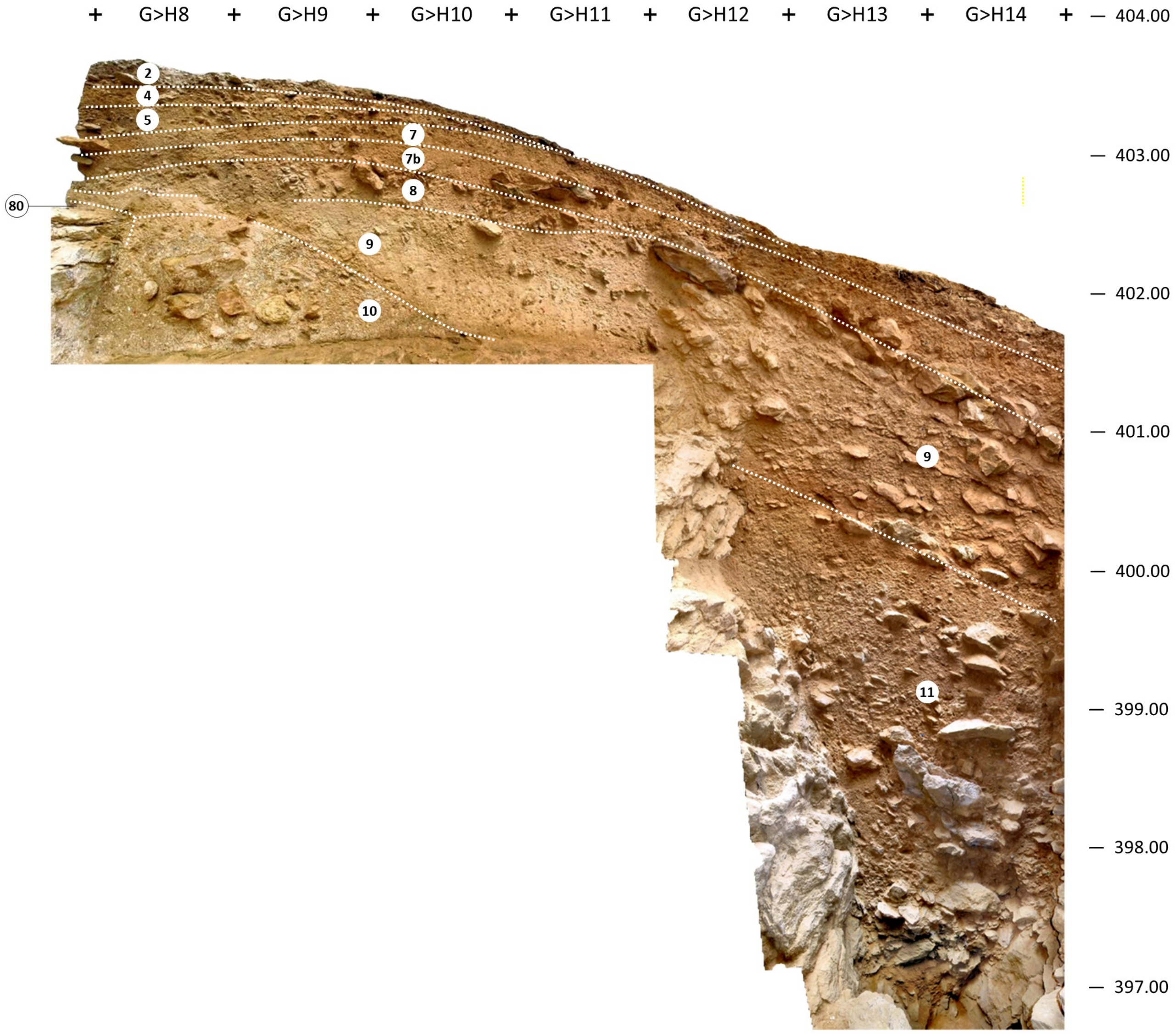

Fig. S3.10. Finca Doña Martina. The G>H stratigraphic cross-section. Orthorectified photomosaic assembled from images taken at the end of the 2013 (G>H8-12) and 2014 (G>H13-14) field seasons. Elevations are in m asl. Note, along the interface between rows 8 and 9 of the grid, the large disturbance feature affecting the sequence from the base of layer 7b to the top of layer 10; at excavation, this feature was observed to run across the entire width of the trench, from column E to column G.

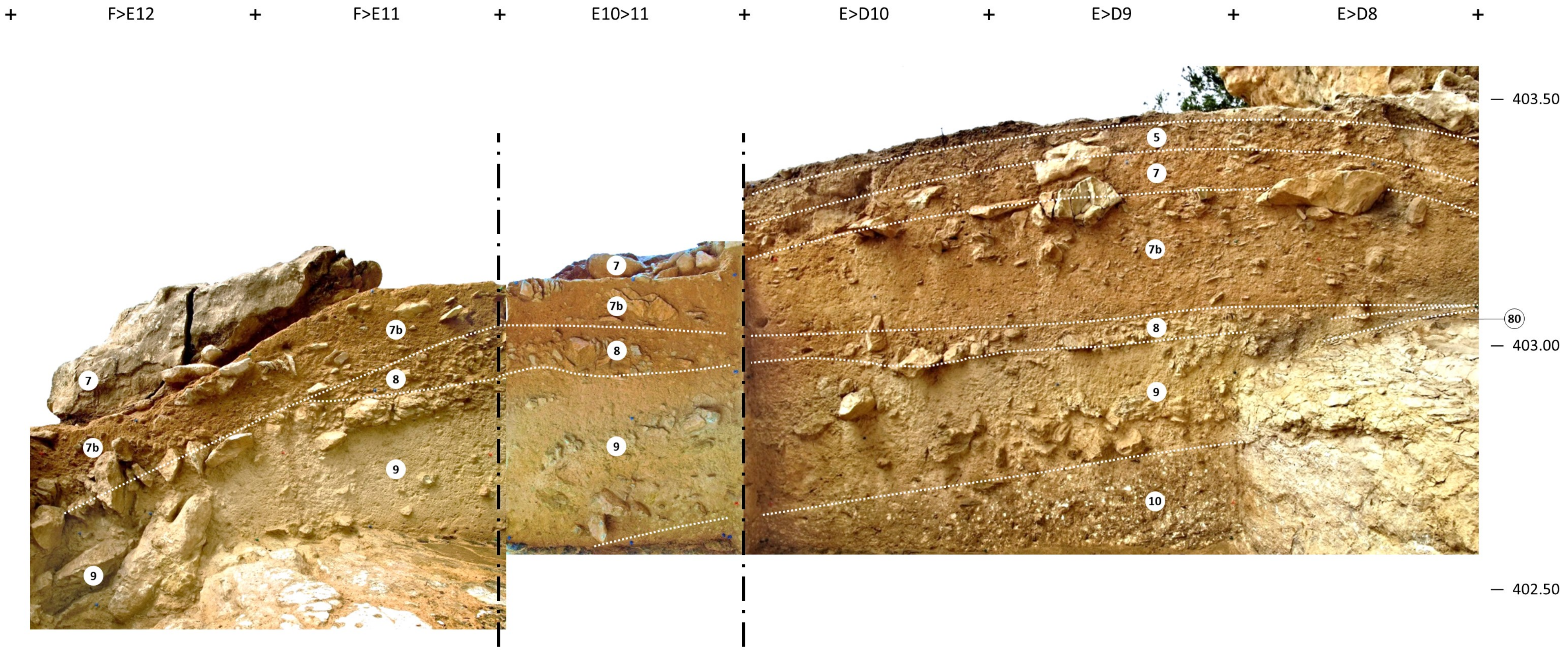

Fig. S3.11. Finca Doña Martina. The F>E, 10>11 and E>D stratigraphic cross-sections. Orthorectified photomosaic assembled from images taken at the end of the 2013 field season. Elevations are in m asl.

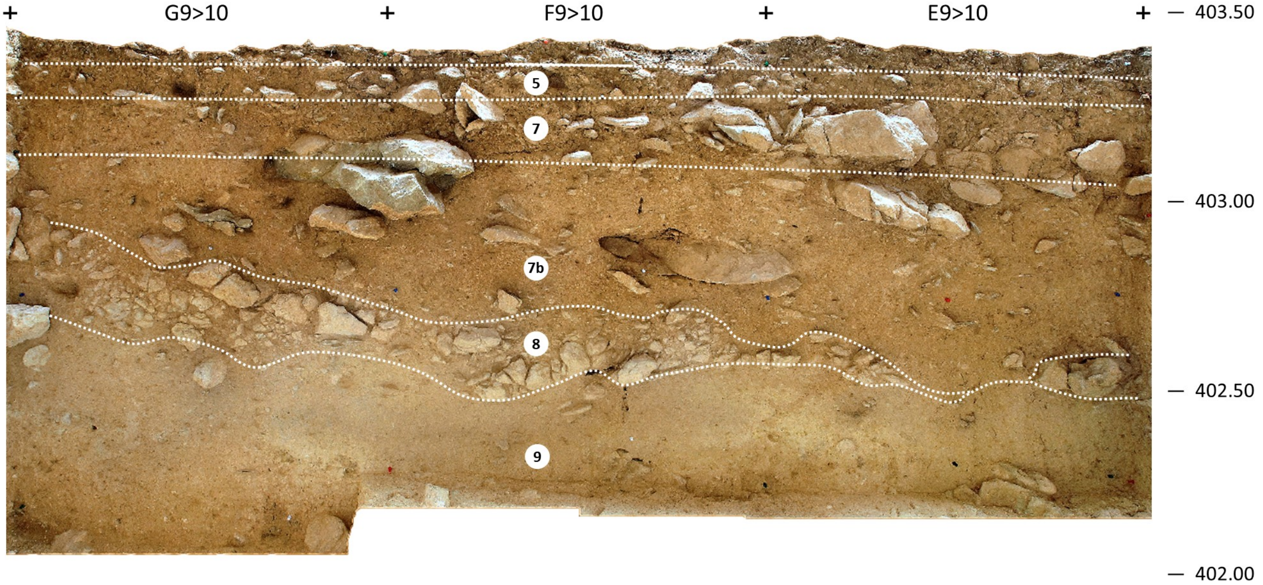

Fig. S3.12. Finca Doña Martina. The G>H stratigraphic cross-section. Orthorectified photomosaic assembled from images taken at the end of the 2012 field season. Elevations are in m asl.

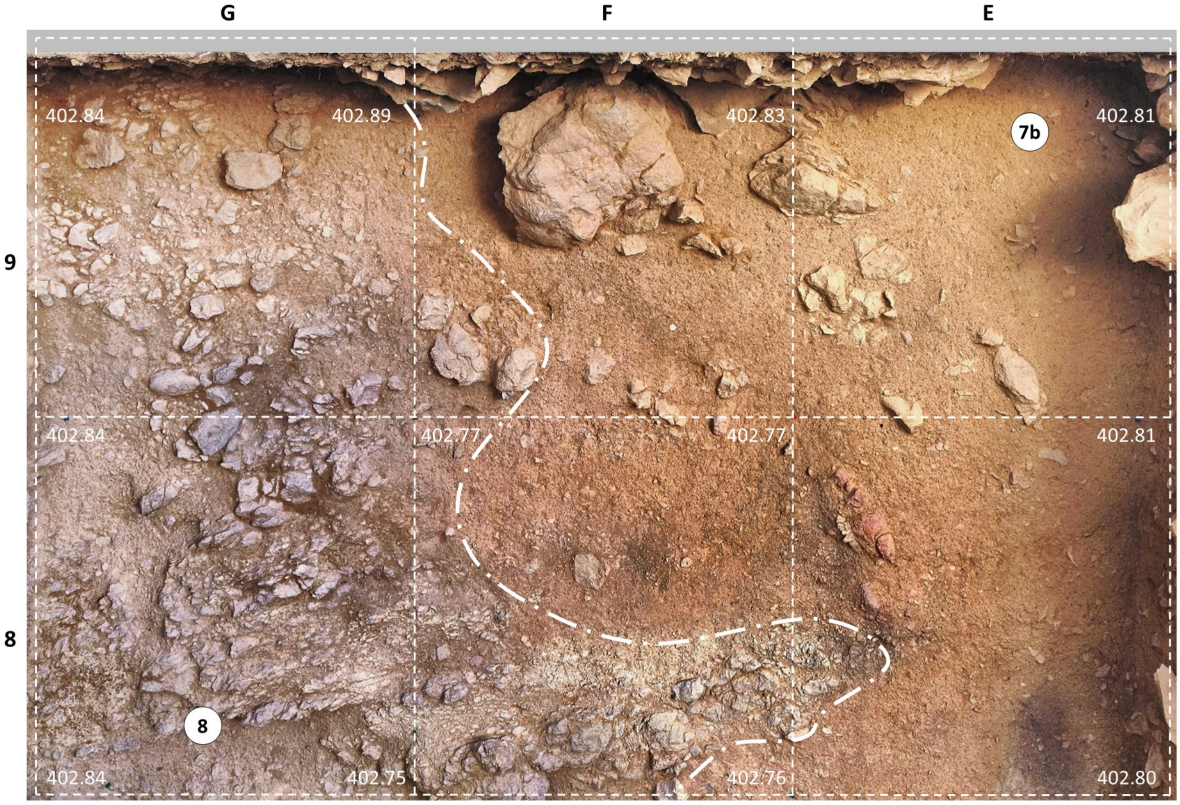

Fig. S3.13. Finca Doña Martina. Horizontal stratigraphy at the base of spit A9 of the excavation of the E/G-8/9 trench. Orthorectified photomosaic assembled from images taken in the 2012 field season. Elevations are in m asl. Note that, along the G8/G7 limit, layer 8 was defined by an accumulation of large stones that were removed prior to the mapping and recording of the surface; in this corner of the trench, the interface between layers 7b and 8 lied at the elevation of 402.90 m.

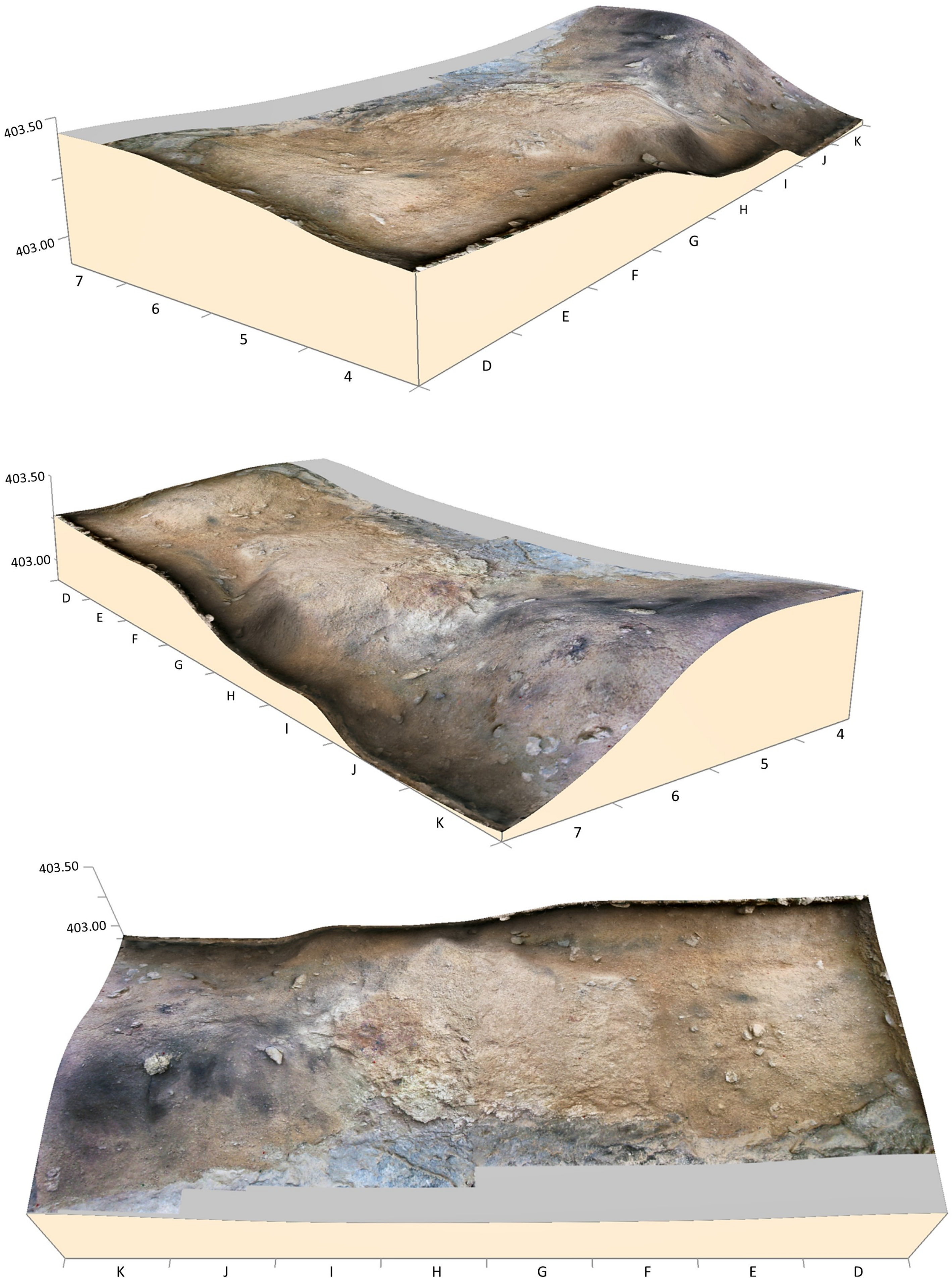

Fig. S3.14. Finca Doña Martina. The surface of layer 6/7 and the major erosional feature that truncated it as exposed in the D-K/4-7 trench. Orthorectified photomosaic assembled from images taken in the 2009 and 2010 field seasons. Elevations are ×2 and in m asl. A hearth, denoted by a semicircular, charcoal-spotted patch of reddened sands embedded in cemented ash can be seen outcropping at this stage in grid units H-I/6.

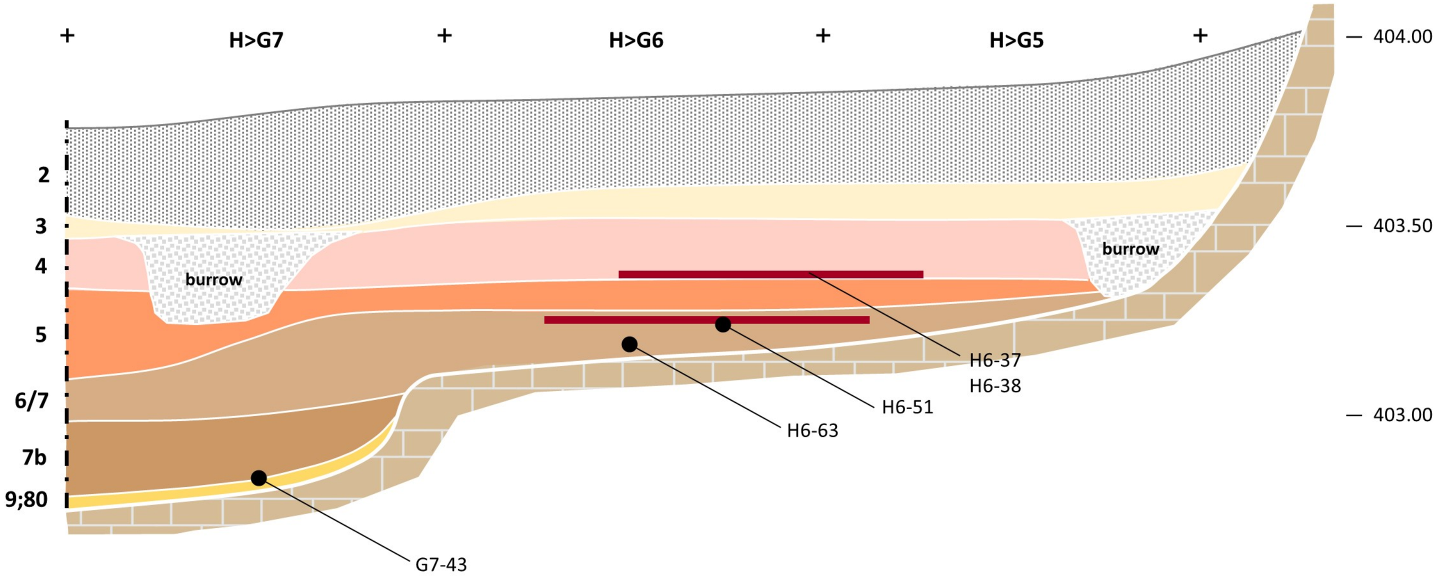

Fig. S3.15. Finca Doña Martina. Provenience of the dated samples. Samples are plotted against a schematic cross-section where layer boundaries are derived from décapage data. The thick red lines denote the rubefaction lenses marking the position of partly eroded Hearths 1 (in layer 4) and 3 (in layer 6/7). Elevations are in m asl.

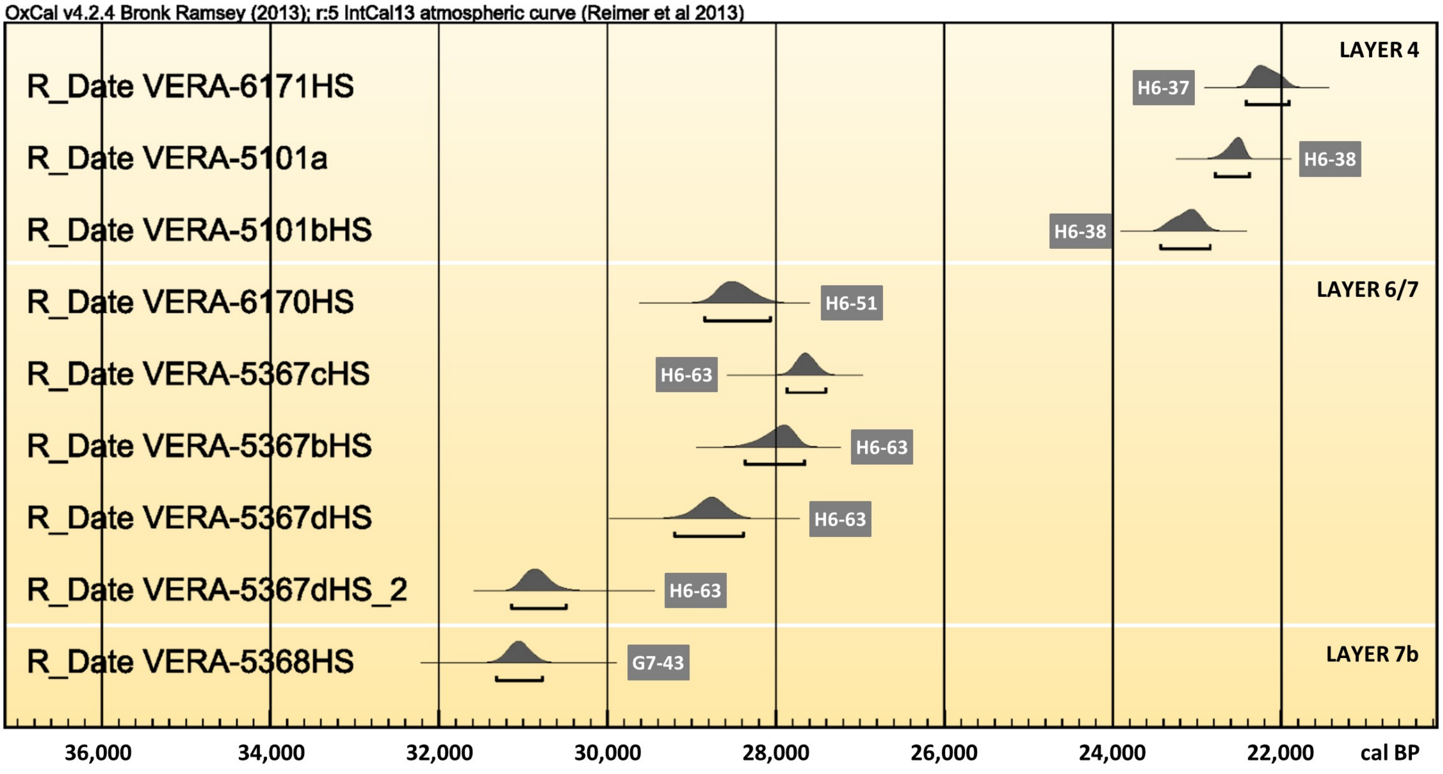

Fig. S3.16. Finca Doña Martina. Radiocarbon results. OxCal plot (Bronk Ramsey, 2009) of calibrated ages (IntCal13; Reimer et al., 2013) against stratigraphic depth. The 95.4% probability intervals are indicated below each result's probability density function. The HS results were measured on the humic acids fraction, while the VERA-5101a result was obtained on a sample whose ABA pre-treatment was mild and incomplete; these results therefore are to be considered as minimum ages only. The field numbers encode the provenience (grid unit and spit) of the charcoal samples whence come the individual pieces making up each dated subsample.

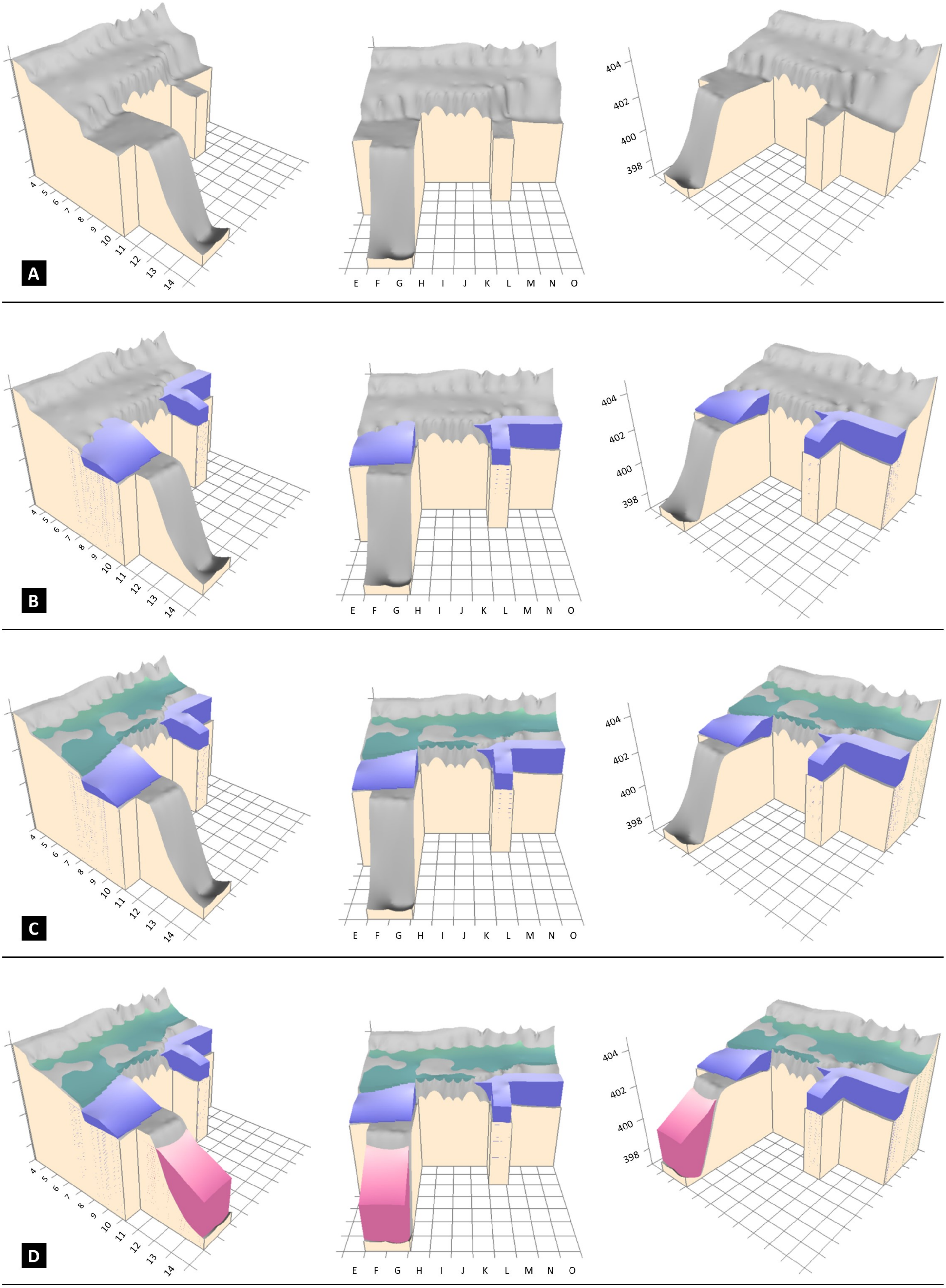

Fig. S3.17. Finca Doña Martina 3D accumulation model. Excavated extent, exposed surface, geometry, position in the sequence, and boundaries of recognized stratigraphic units. **A.** Bedrock. **B.** Layer 10. **C.** Layer 80. **D.** Layer 11. Elevations are in m asl.

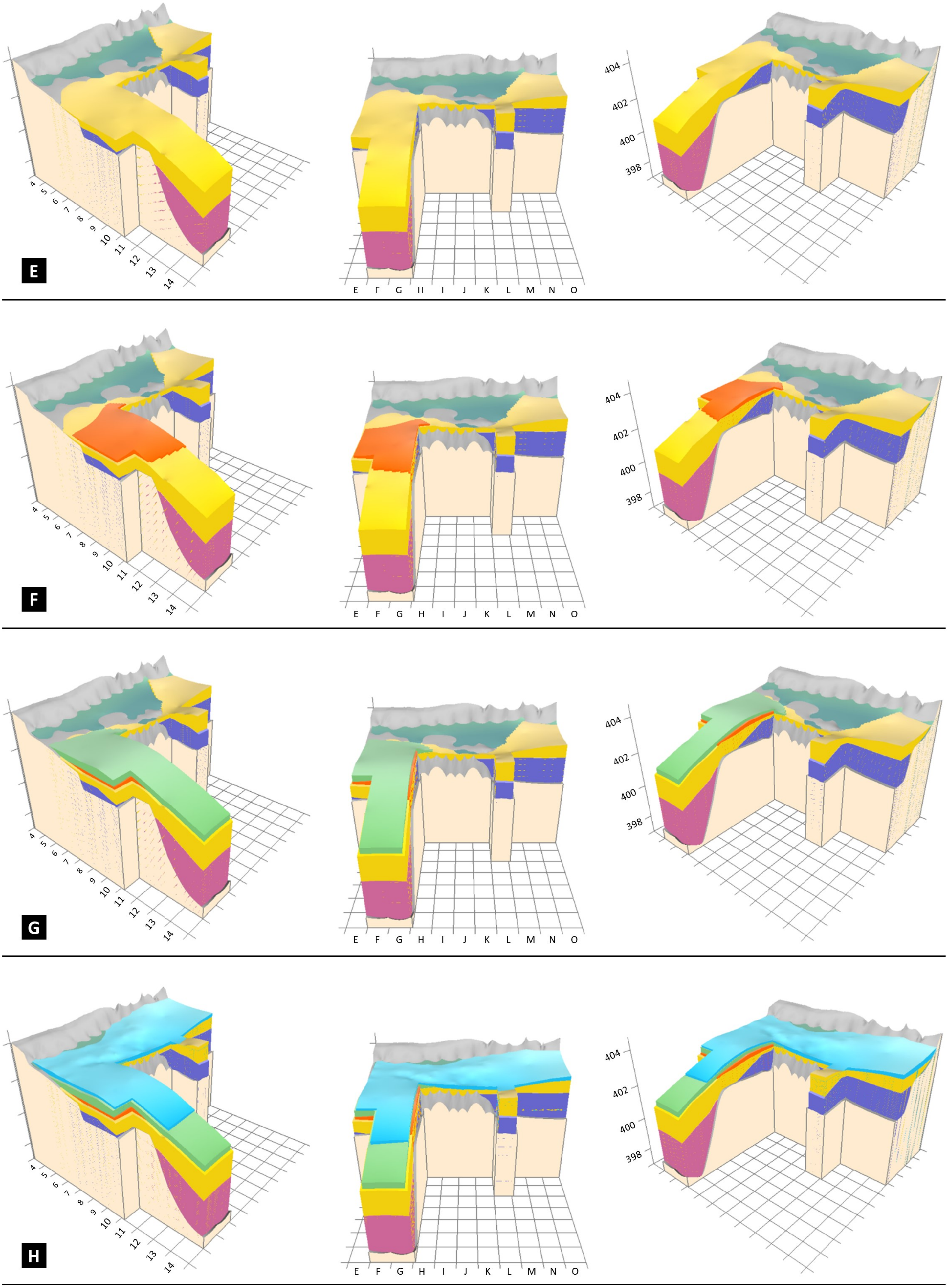

Fig. S3.17 (cont.). Finca Doña Martina 3D accumulation model. Excavated extent, exposed surface, geometry, position in the sequence, and boundaries of recognized stratigraphic units. **E.** Layer 9. **F.** Layer 8. **G.** Layer 7b. **H.** Layer 6/7. Elevations are in m asl.

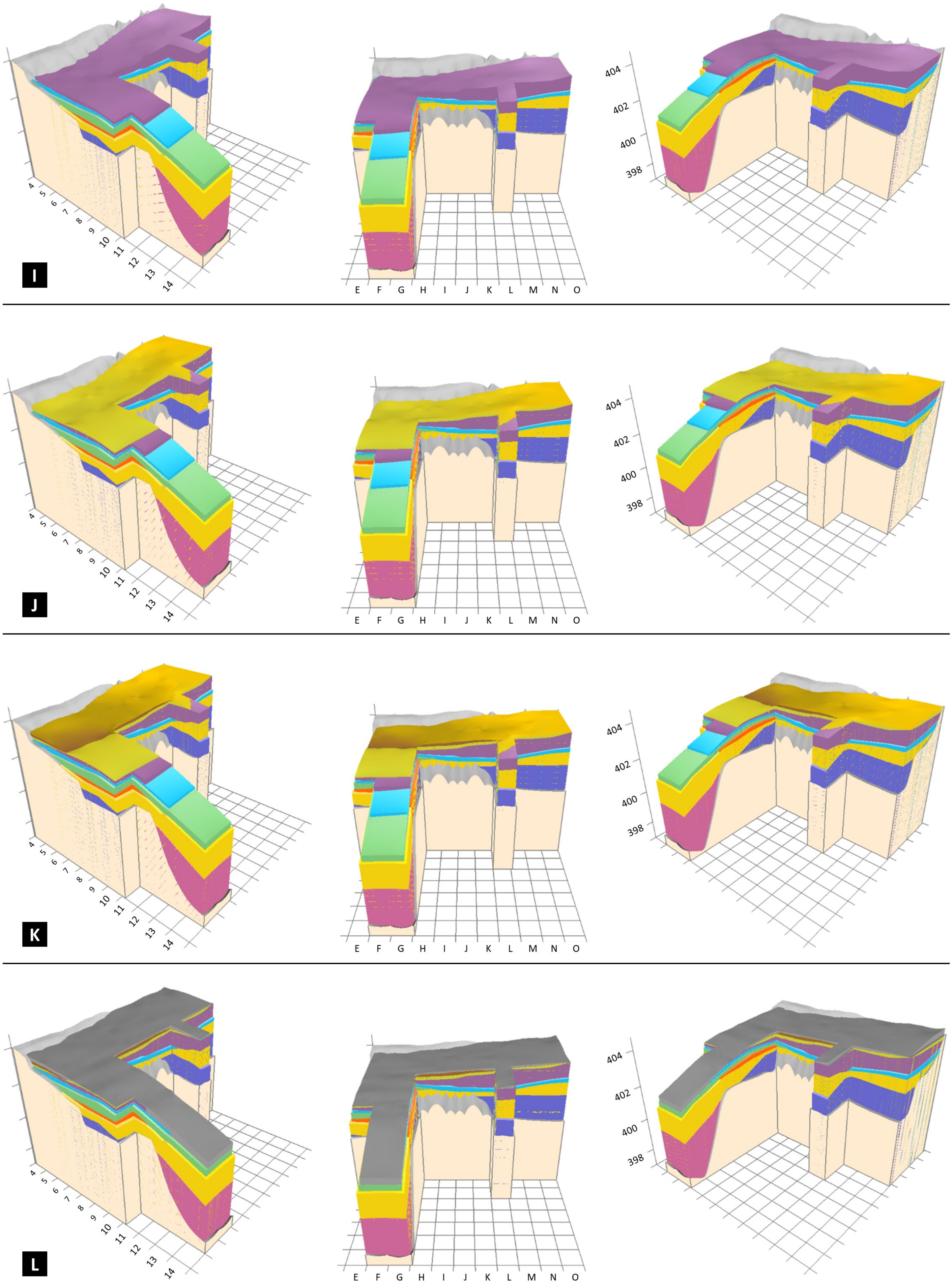

Fig. S3.17 (cont.). Finca Doña Martina 3D accumulation model. Excavated extent, exposed surface, geometry, position in the sequence, and boundaries of recognized stratigraphic units. **I.** Layer 5. **J.** Layer 4. **K.** Layer 3. **L.** Layer 1/2. Elevations are in m asl.

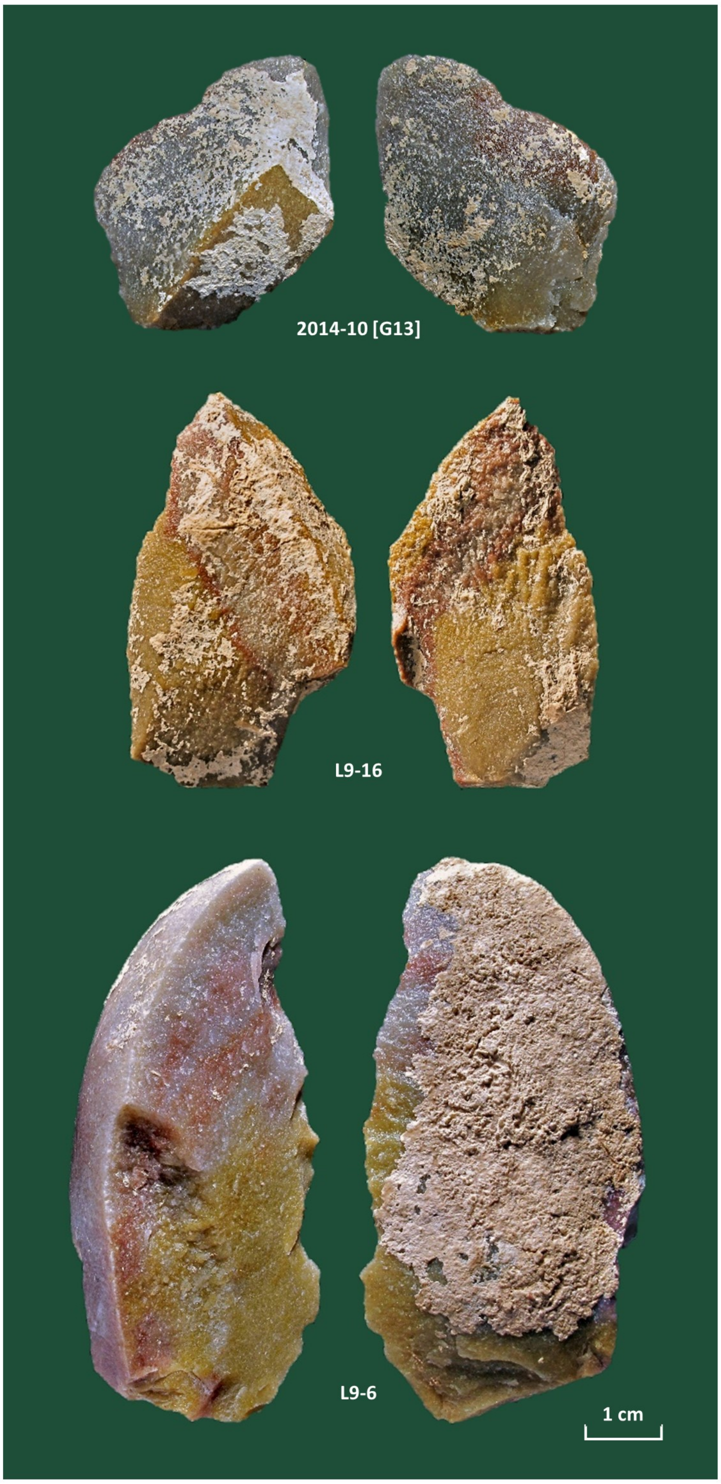

Fig. S3.18. Finca Doña Martina. Mousterian crust-coated lithics (prior to cleaning). Quartzite: blanks (L9-16, 2014-10), and naturally backed knife (L9-6).

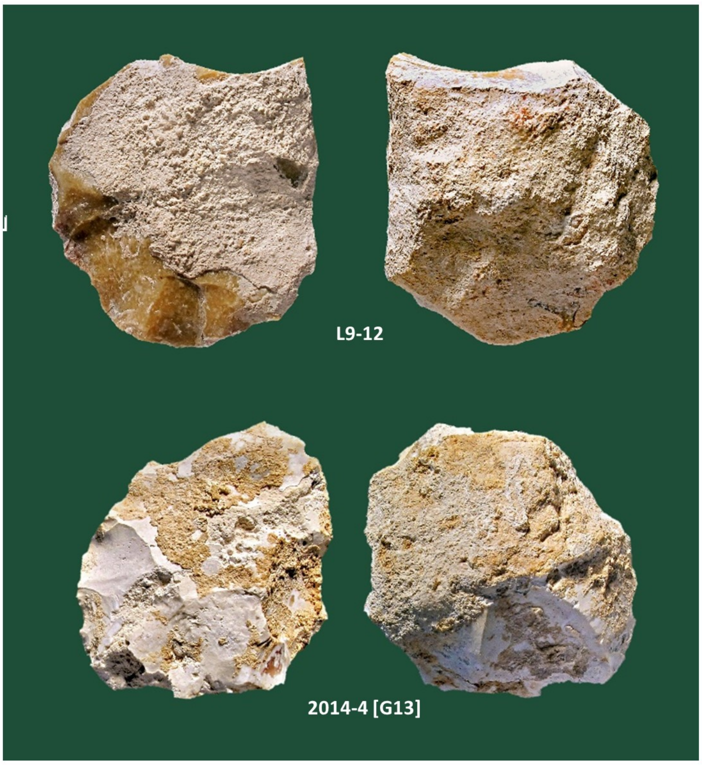

Fig. S3.19. Finca Doña Martina. Mousterian crust-coated lithics (prior to cleaning). Flint: cores.

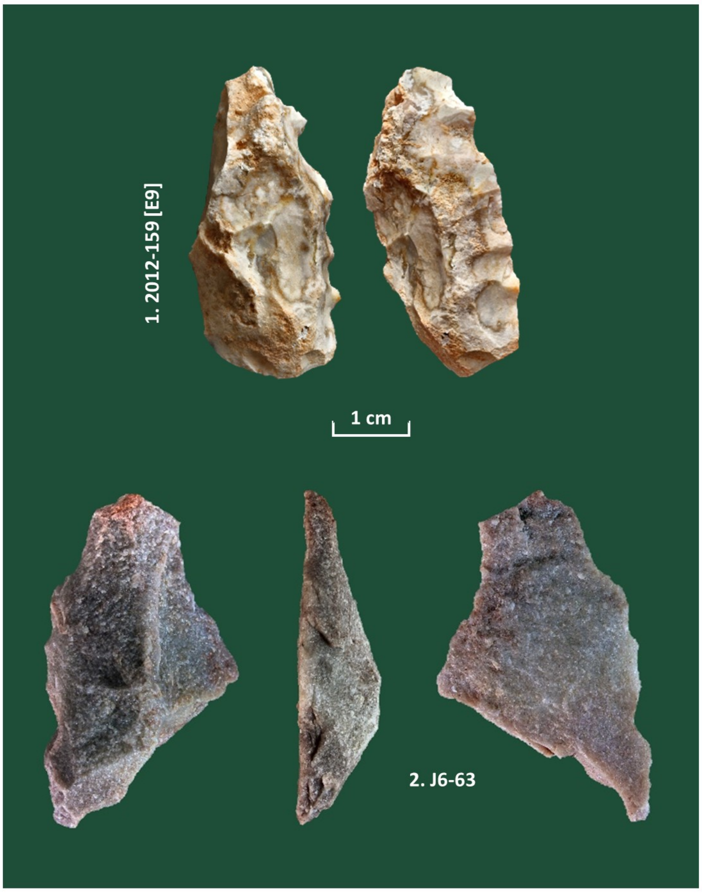

Fig. S3.20. Finca Doña Martina. Middle Paleolithic stone tools reworked into the overlying Upper Paleolithic deposit.
1. Denticulated sidescraper made on a cortically-backed, orange-segment blank retrieved in grid unit E9 at the base of layer 7b, in a situation of lateral contact with layers 8 and 9. **2.** Fragment of a slightly patinated denticulated sidescraper made of quartzite, retrieved at the base of layer 6/7 in grid unit J6.

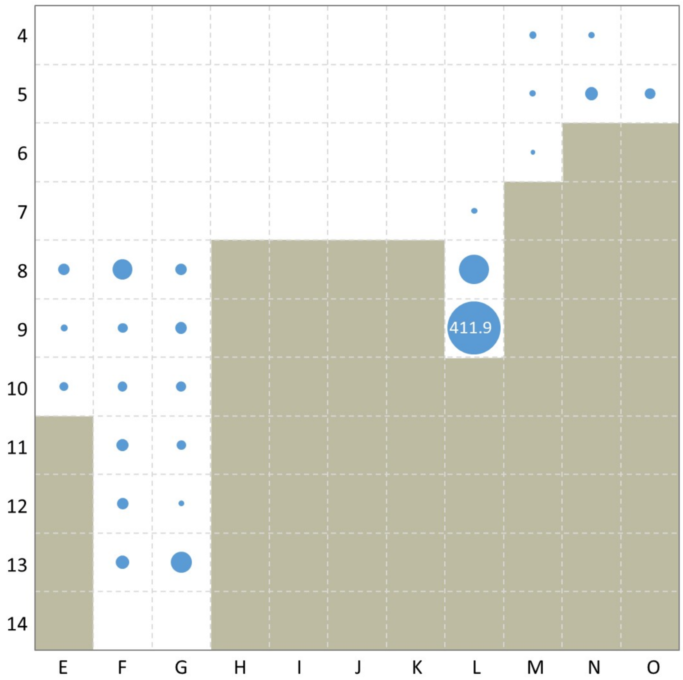

Fig. S3.21. Finca Doña Martina. Spatial distribution of the layer 9 lithics (2007-2014). Each bubble is proportional to the corresponding grid unit's percentage of the total mass (990.8 g) of the assemblage. The highest value is indicated.

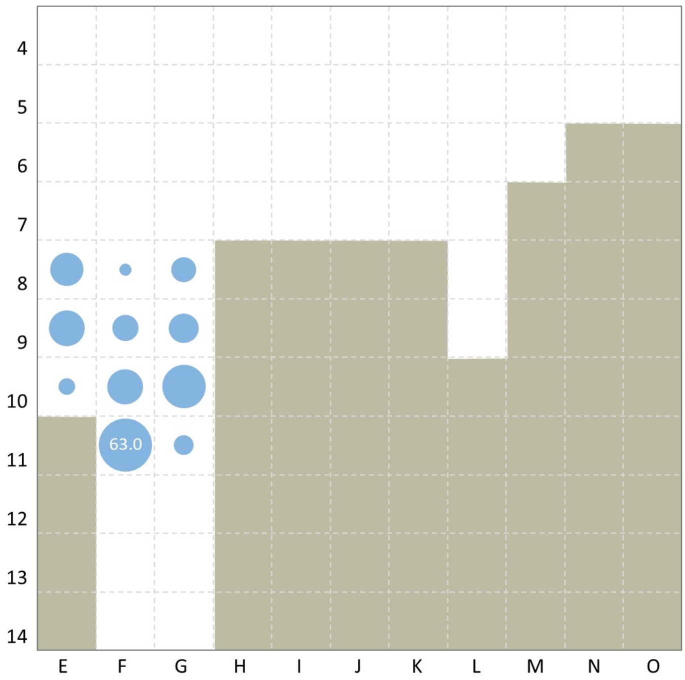

Fig. S3.22. Finca Doña Martina. Spatial distribution of layer 8 lithics (2007-2014). Each bubble is proportional to the corresponding grid unit's percentage of the total mass (255.0 g) of the assemblage. The highest value is indicated.

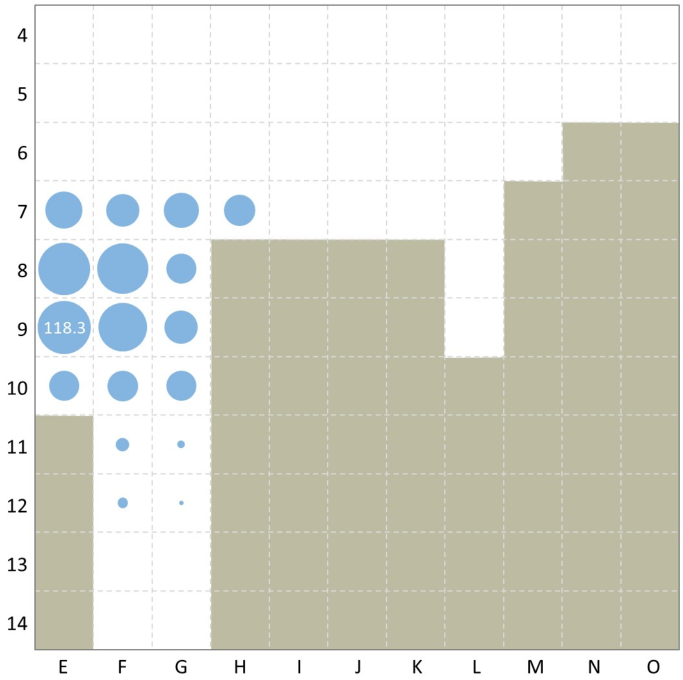

Fig. S3.23. Finca Doña Martina. Spatial distribution of layer 7b lithics (2007-2014). Each bubble is proportional to the corresponding grid unit's percentage of the total mass (824.4 g) of the assemblage. The highest value is indicated.

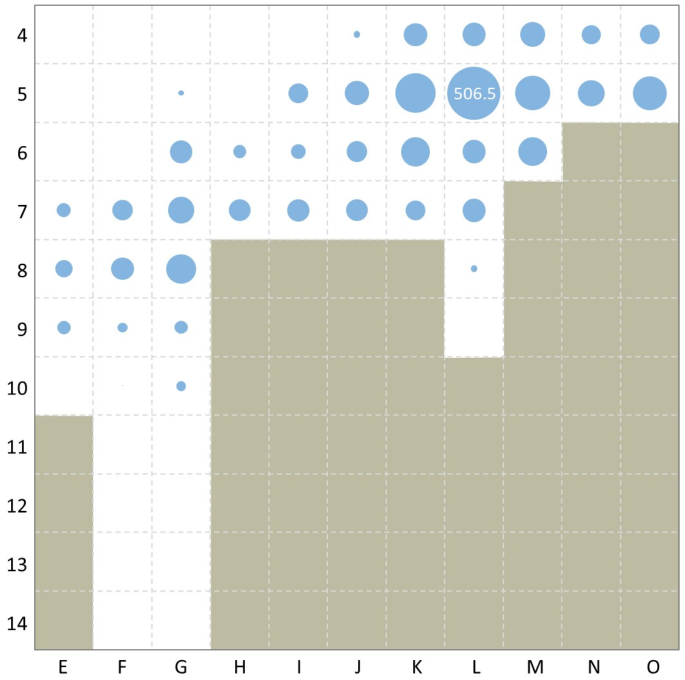

Fig. S3.24. Finca Doña Martina. Spatial distribution of layer 6/7 flint artefacts (2007-2014). Each bubble is proportional to the corresponding grid unit's percentage of the total mass (3704.9 g) of the assemblage. The highest value is indicated. Due to the significant bioturbation observed in E-F/5-6, the few artefacts recovered therein were not considered.

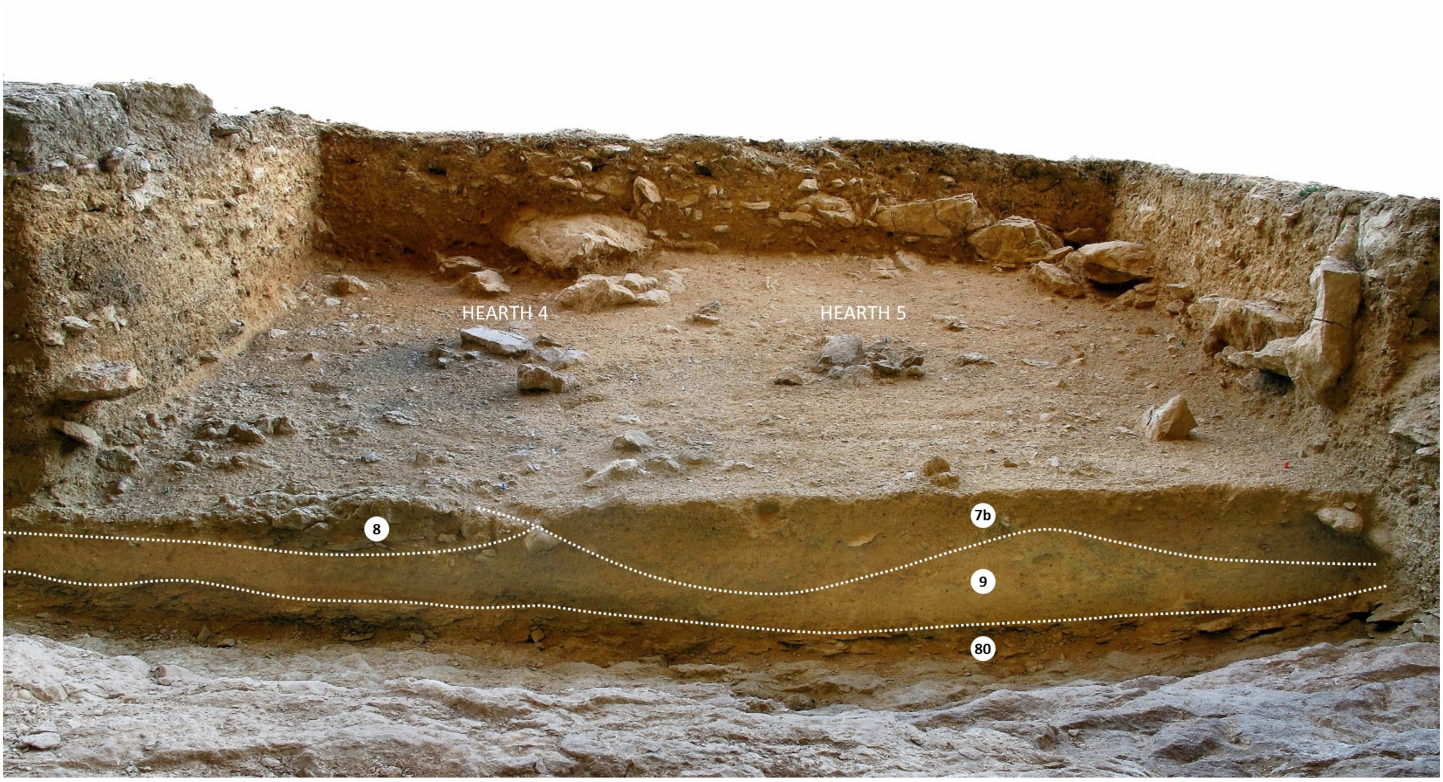

Fig. S3.25. Finca Doña Martina. The hearths in layer 7b. Oblique view of the E-G/8-9 trench, from NE, over the A6 décapage, which exposed the surface of layer 7b in these grid units. The well apparent clusters of burnt rocks surrounded by black-stained sediment denote the outcropping of Hearths 4 and 5. The approximate outlines of the contacts between layers 7b, 8 and 9 along the then still extant base of the E-G7>8 cross-section are indicated; note how layers 8 and 7b fill the scoured surface of layer 9.

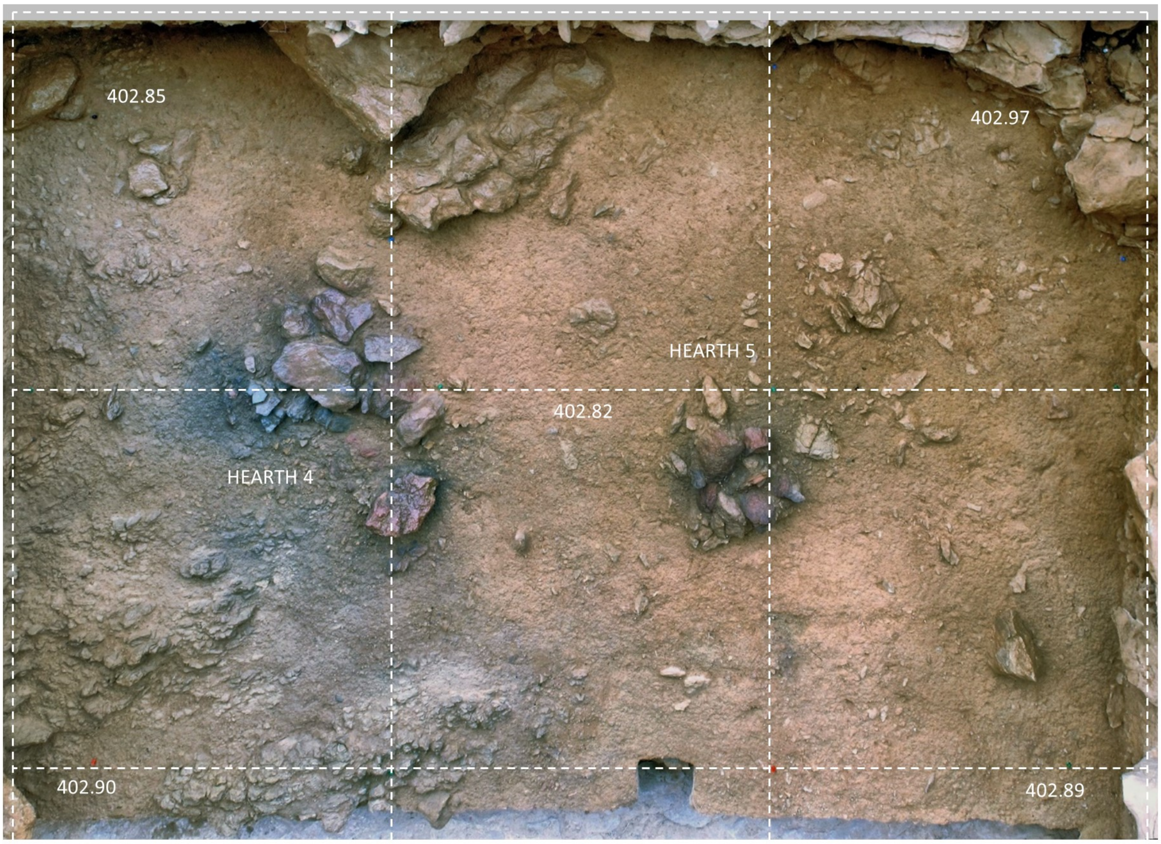

Fig. S3.26. Finca Doña Martina. The hearths in layer 7b. Orthorectified mosaic of the A7 décapage of the E-G/8-9 trench — base of the first, ca.2-3 cm-thick spit excavated into layer 7b of these units to better define the features identified when the surface of the stratigraphic unit was exposed. Prior to photography, the sediment was water-sprinkled to enhance the contrast between the two clusters of burnt stones and the layer's unburnt clast component. Note how, to the East, layer 7b and its features abut an outcropping layer 8. Elevations are in m asl.

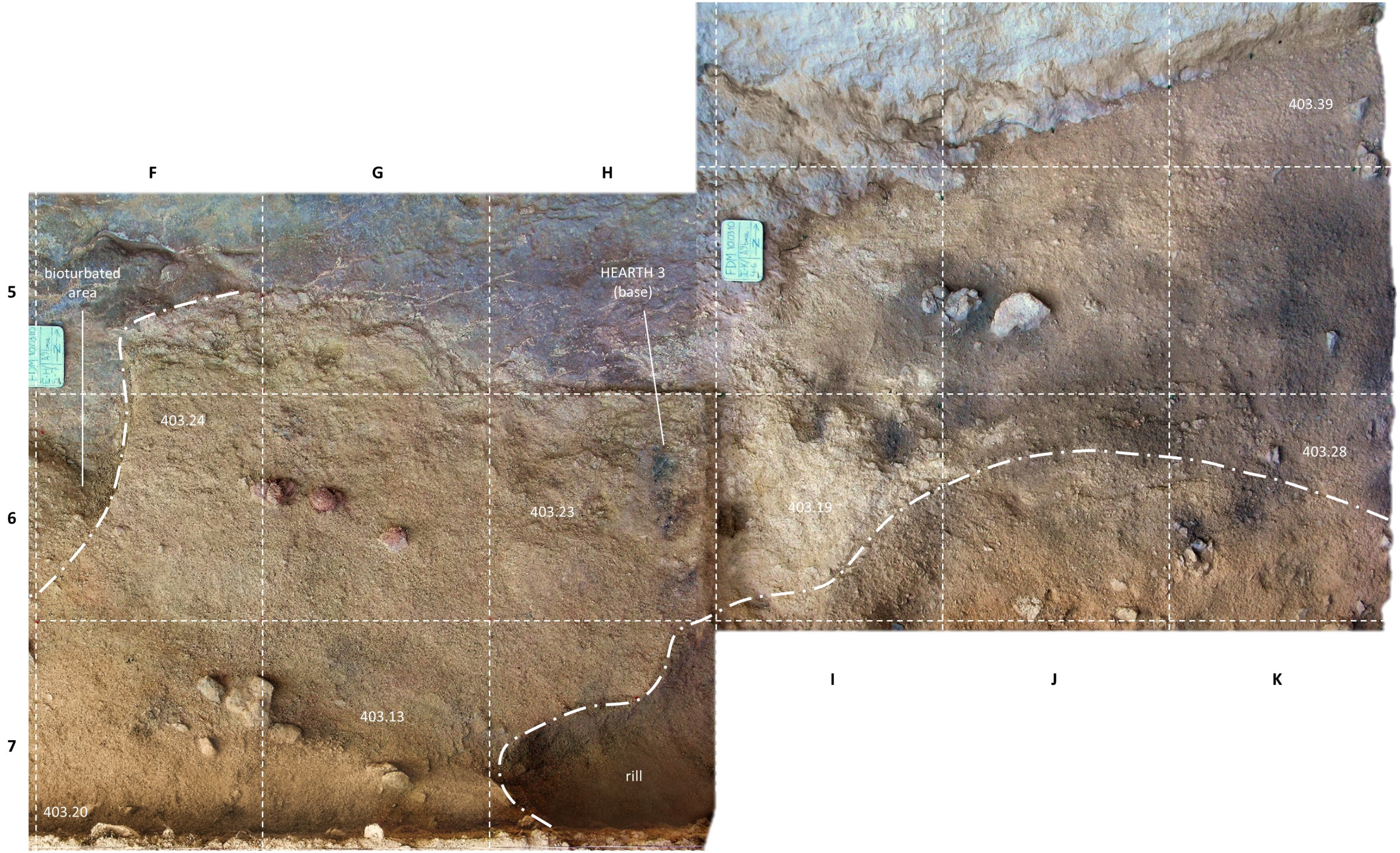

Fig. S3.27. Finca Doña Martina. The hearth in layer 6/7. Orthorectified mosaic of the A9 décapage of the E-K/4-7 trench, base of the first spit excavated into layer 6/7 of these grid units. The reddened sediments associated with a concentration of charcoal seen in grid unit H6 denote the base of Hearth 3. At this stage, grid unit I6 had already been taken down to bedrock in order to obtain a cross-section enabling sampling of Hearth 3 for soil micromorphological analysis. Note the fire-reddedned stones in G6. Elevations are in m asl.

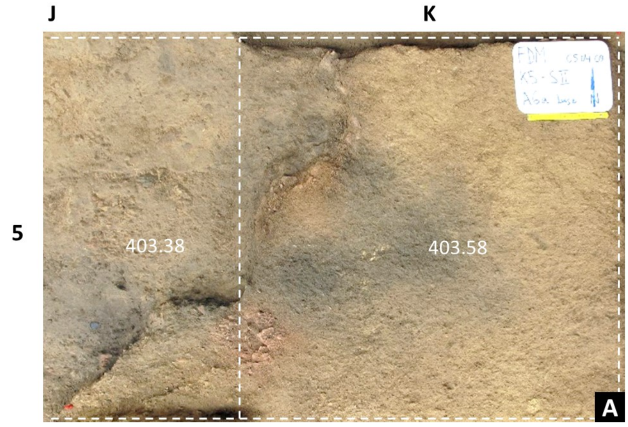

Fig. S3.28. Finca Doña Martina. The layer 4 hearths. A. Hearth 2, denoted by a cluster of burnt stones cut by the rabbit warren and associated with a concentration of micro-charcoal particles. **B.** Detail of the section (5 cm-thick) cut into Hearth 1 for micromorphological sampling. **C.** Hearth 1's remnant of cemented ash and charcoal after sectioning for micro-stratigraphic observation and sampling. **D.** The base of Hearth 1 once horizontally sectioned to verify the extension of the reddened stain and the original size of the feature **E.** The base of the A5 décapage in grid units H-K/4-7 (approximate interface between layers 4 and 5), featuring the position of the hearths relative to the rabbit warren in the middle. Elevations are in m asl. **A**, **C** and **D** have been orthorectified.

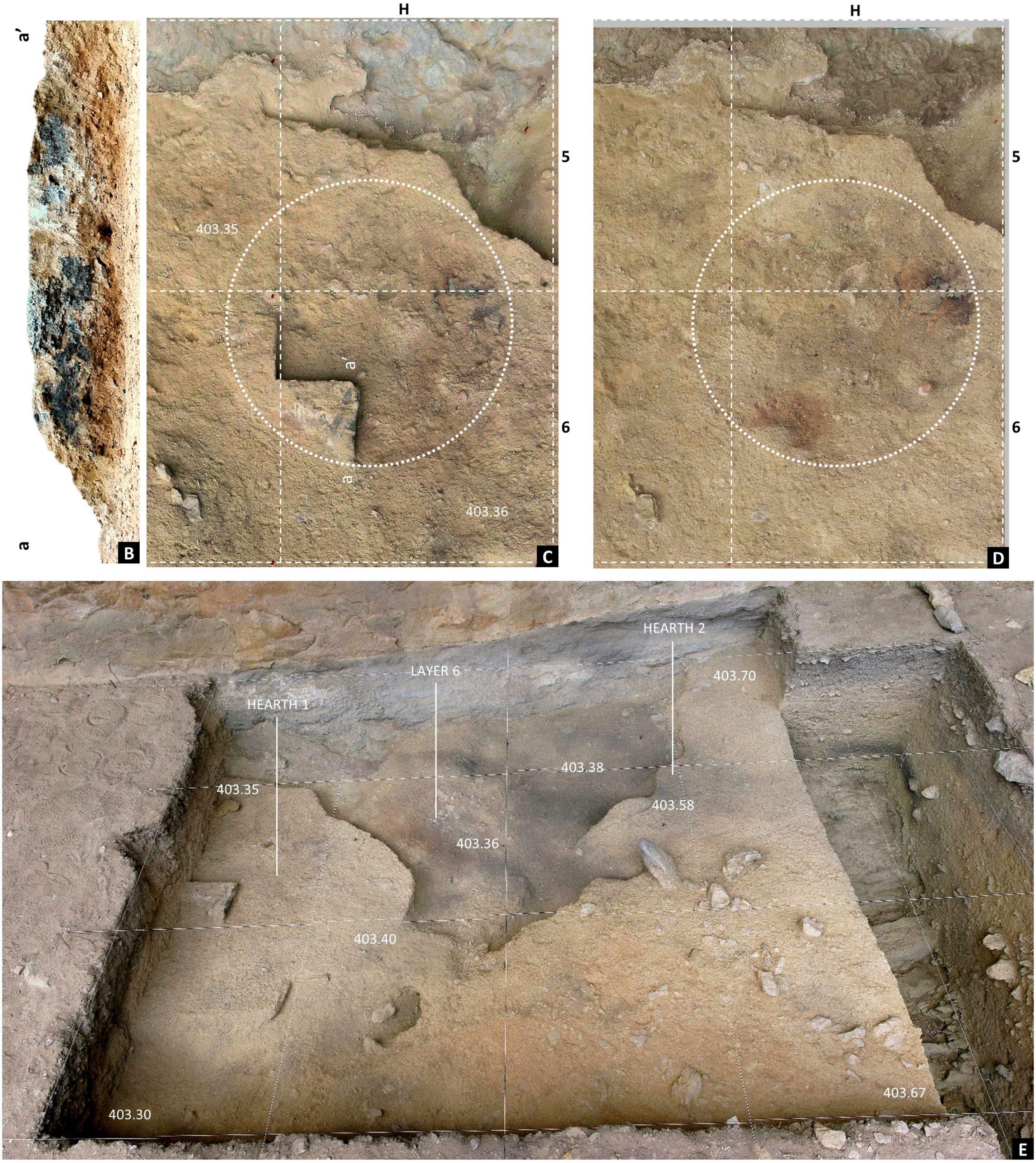

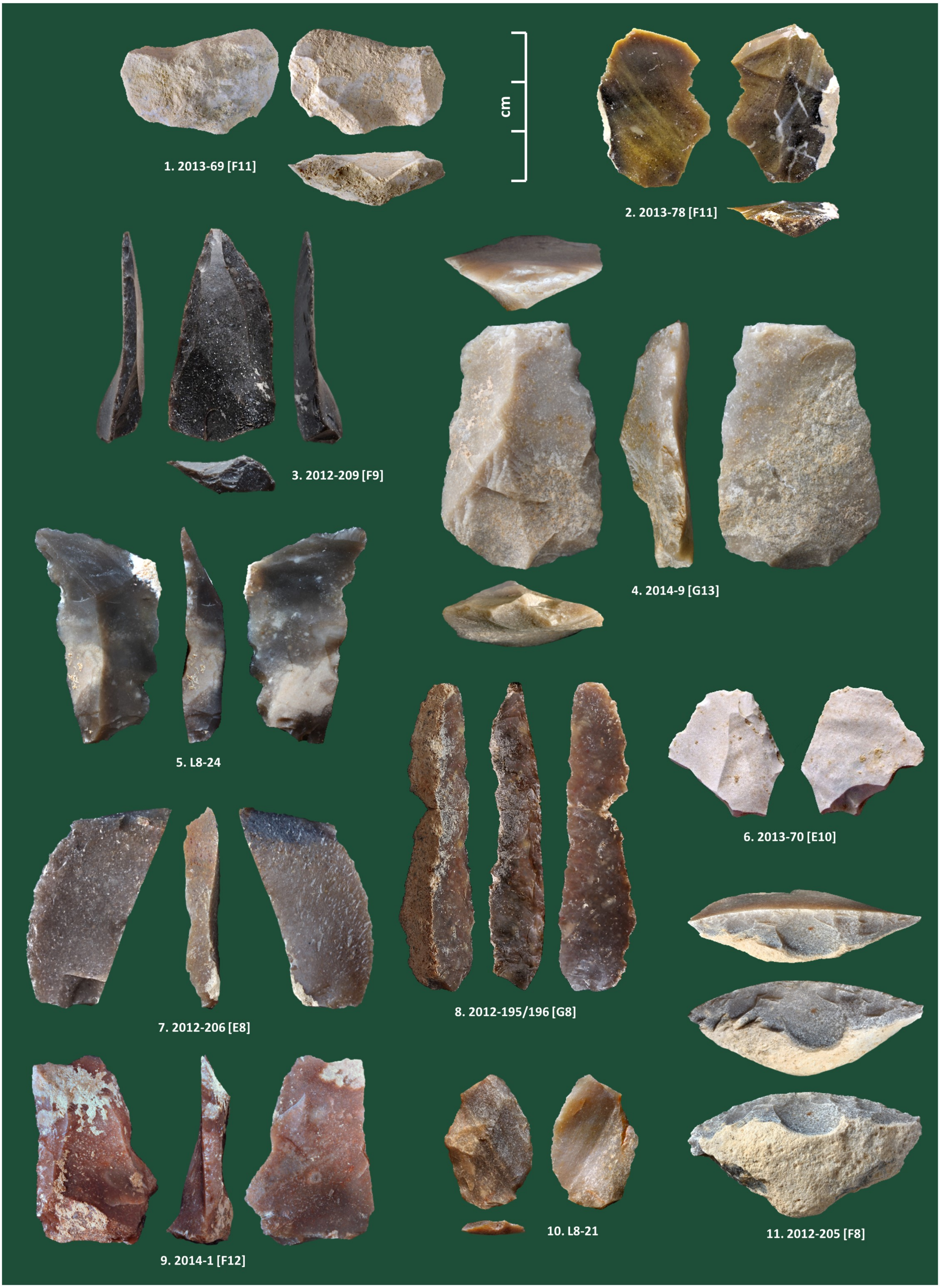

Fig. S3.29. Finca Doña Martina. Middle Paleolithic stone tools from layer 9. 1. Kombewa core on cortical flake blank; **2.** Levallois flake; **3-4.** Mousterian points; **5.** denticulate; **6-11.** sidescrapers.

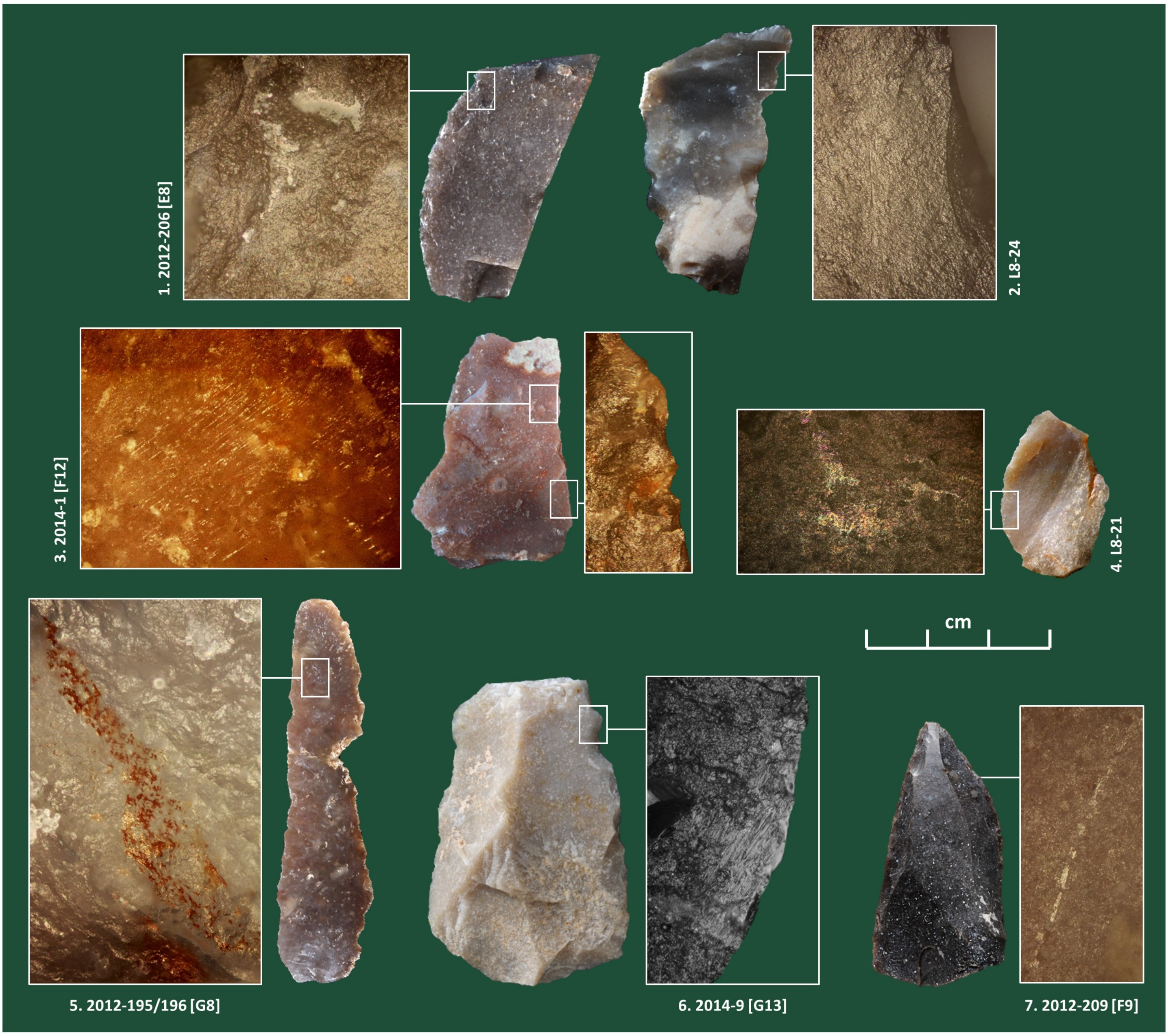

Fig. S3.30. Finca Doña Martina. Middle Paleolithic use-wear evidence. 1. The dense, curved, shiny, well-delimited polish denotes use in bone- or antler-working (×200); **2.** The detail illustrates an edge-sharpening removal that eliminated most of a use-worn area, preventing identification of motion and of the material the piece was used on (×100); **3.** The striations perpendicular to the distal edge (×400) denote a meat-cutting action while the intensity of the proximal polish denotes a hide-scraping action (×200); **4.** The attenuated, low-density, moderately shiny polish denotes wood-working (×200); **5.** The successive re-sharpening, till exhaustion, eliminated the use-wear evidence but ochre is present on the ventral side, suggesting use in hide-working tasks (×200); **6-7.** The impact striations (for 2012-209, on the ventral side) denote use as projectile points (×200).

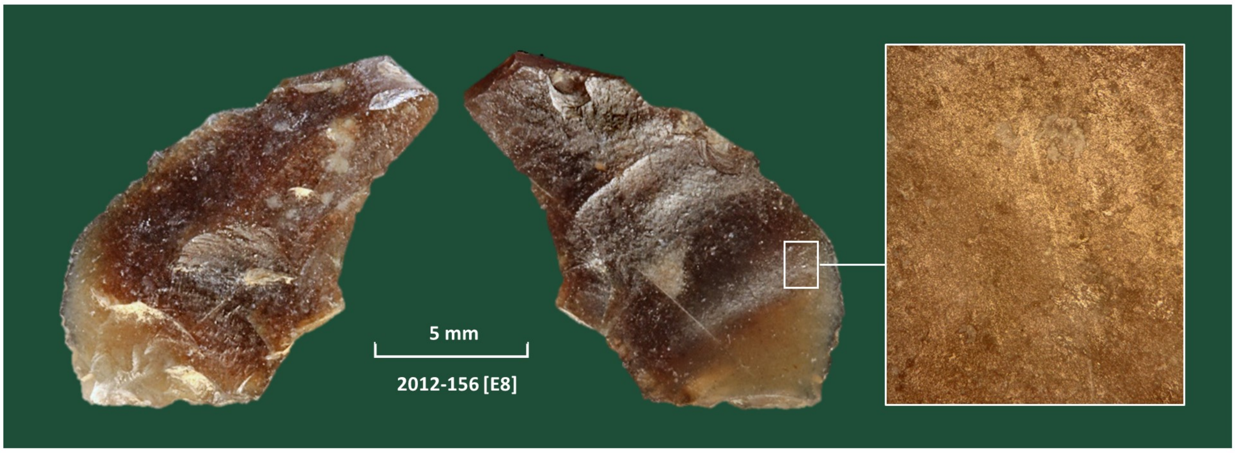

Fig. S3.31. Finca Doña Martina. Aurignacian Dufour bladelet. Short, twisted blank from carinated/nosed core with inverse retouch on the left; the proximal striations on the ventral side (×200) denote use as a projectile element.

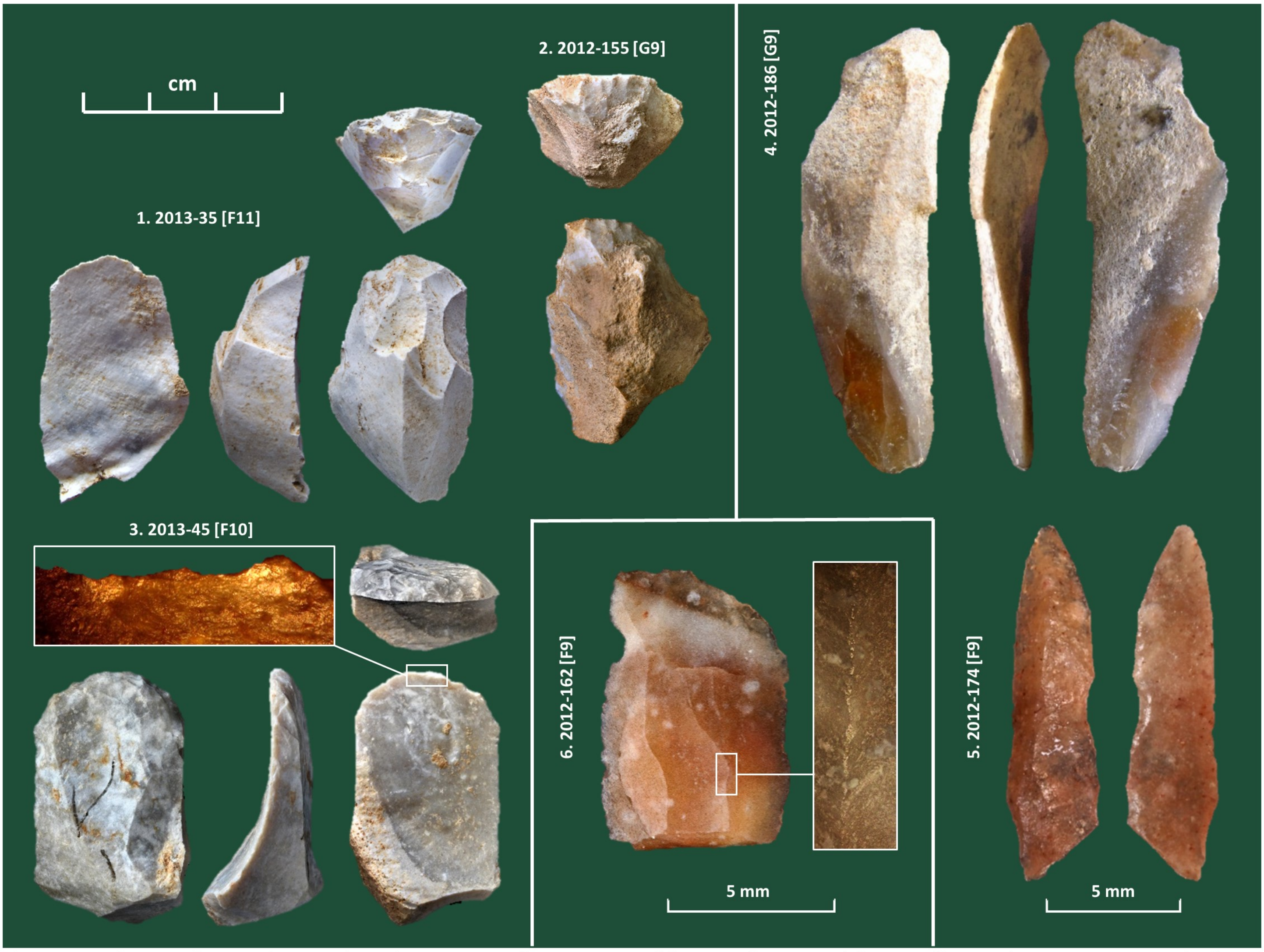

Fig. S3.32. Finca Doña Martina. Aurignacian stone tools. 1. carinated "scraper"; **2.** nosed "scraper"; **3.** endscraper on retouched piece with intense hide-working polish (×200); **4.** unretouched blank from carinated/nosed "scraper;" **5.** Dufour bladelet; **6.** bladelet with a spot of hafting-related bone polish on a dorsal ridge (×400) and edge-wear "retouch" caused by use in a composite projectile.

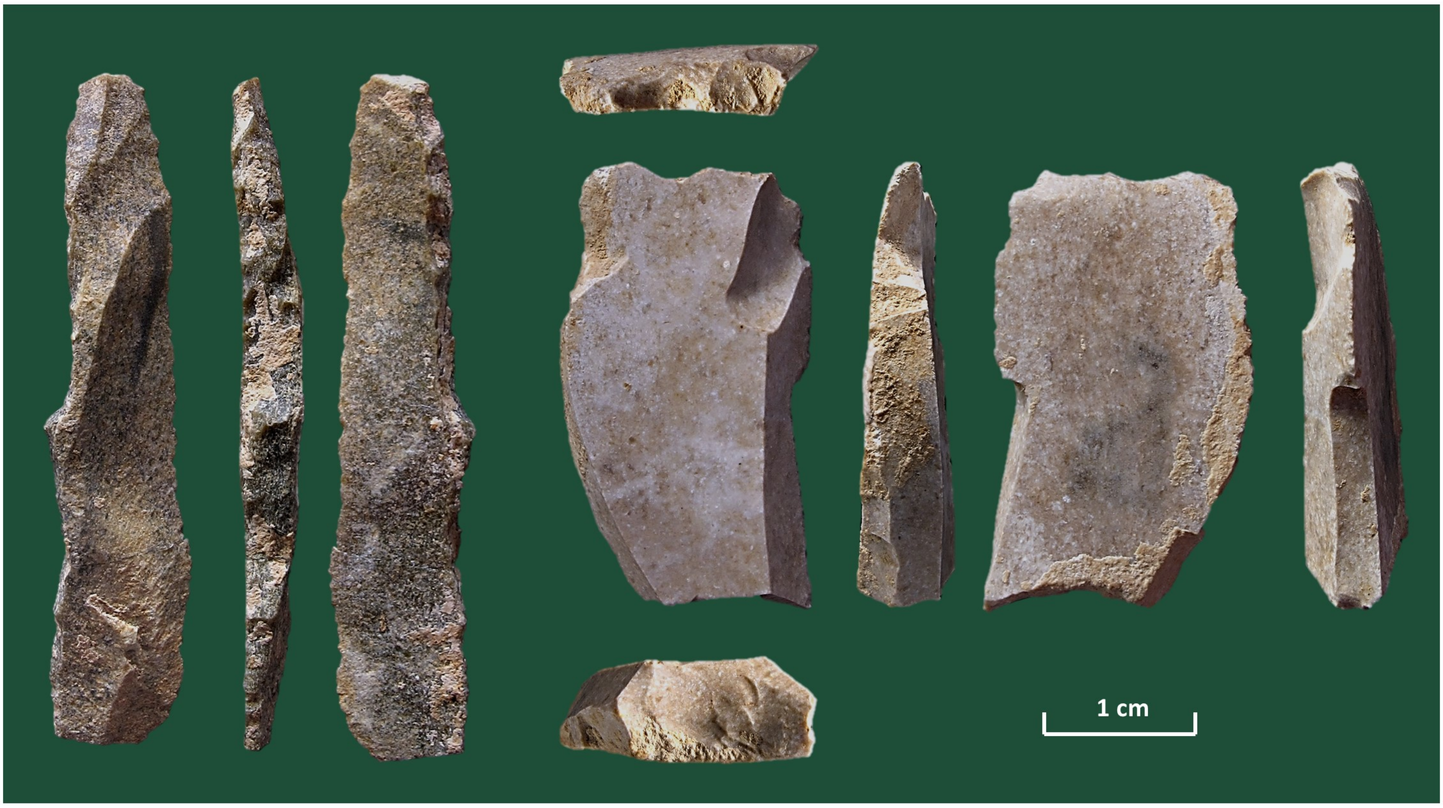

Fig. S3.33. Finca Doña Martina. Early Gravettian stone tools. Left. Gravette point (2013-57, F11) with minor distal and apical breaks. **Right.** multiple "burin" on truncation (2013-61, F11).

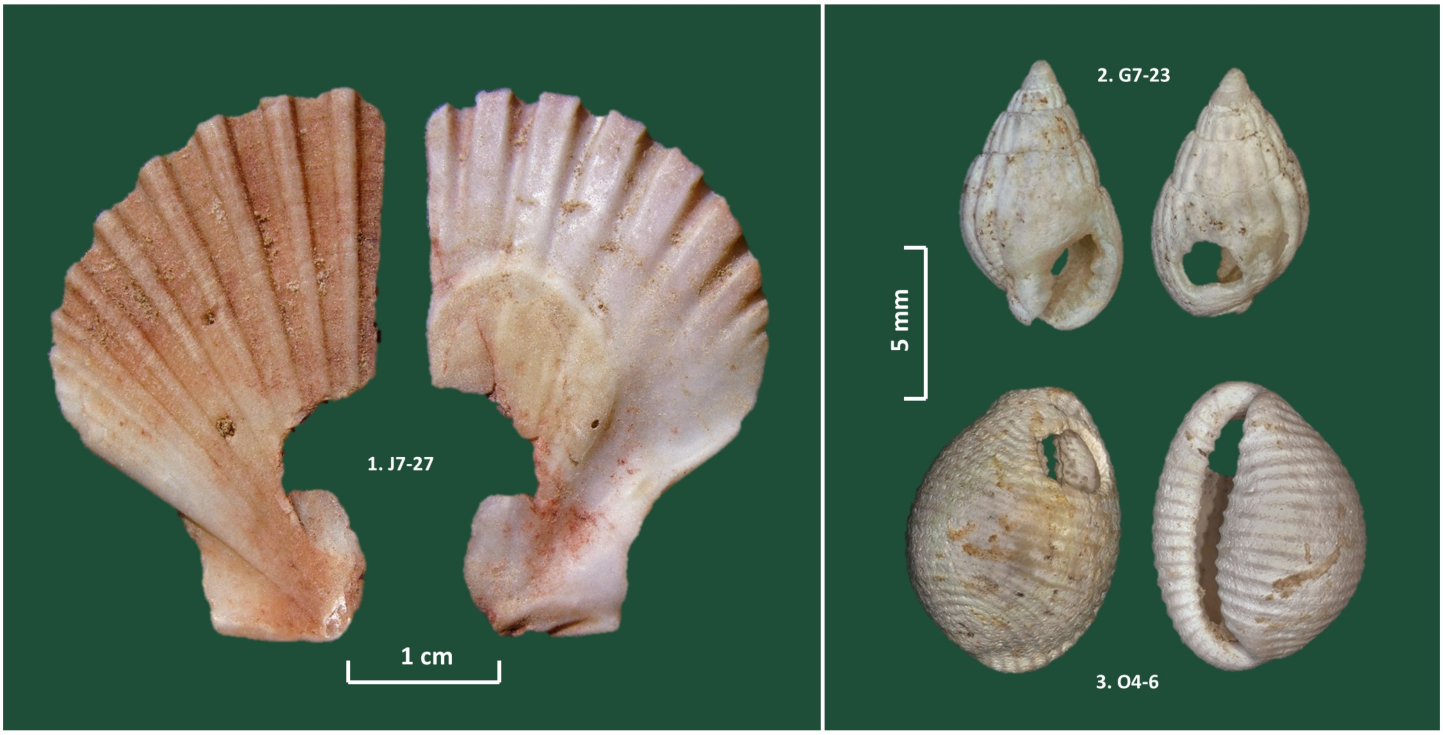

Fig. S3.34. Finca Doña Martina. Middle Gravettian (layer 6/7) personal ornaments. 1. ochred, perforated, half-broken left valve of *Pecten jacobaeus*; **2.** perforated *Nassarius incrassatus*; **3.** perforated *Trivia* sp.

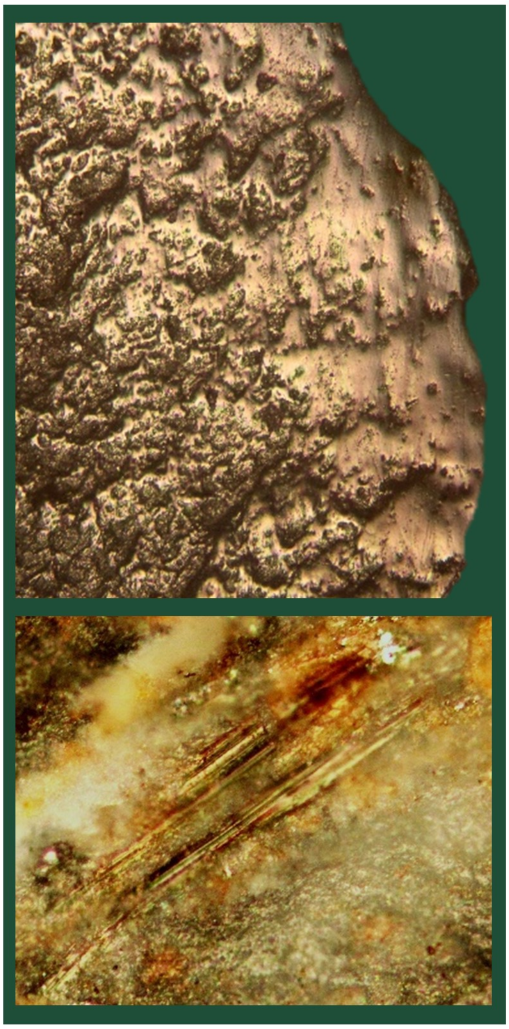

Fig. S3.35. Finca Doña Martina. Middle Gravettian use-wear evidence. Top. K4-24, an endscraper with hide-working polish on the front (×200). **Bottom.** Impact striations on the H6-60 apical fragment of a Gravette point (×400).

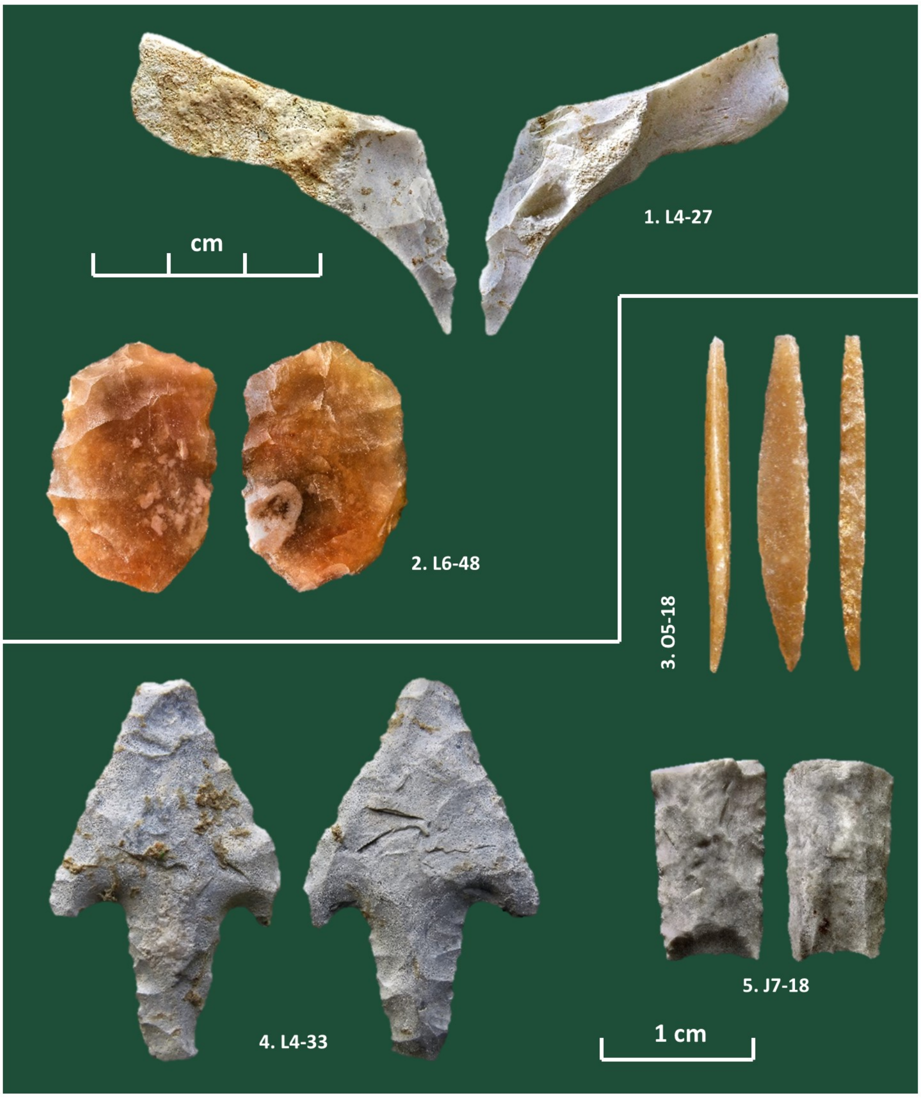

Fig. S3.36. Finca Doña Martina. Middle Gravettian and Upper Solutrean stone tools. 1. overshot bifacial thinning flake; **2.** double endscraper on broken laurel-leaf; **3.** microgravette with apical break; **4.** Parpallò point with apical impact break; **5.** tang of Parpallò point with basal impact scar (1-2,4. layer 5; 3. layer 6/7; 5. layer 4).

Chapter 4.
The rock-shelter of La Boja

4.1. DESCRIPTION

The Abrigo de La Boja (ADB; 38°04’43” N, 01°29’23” W) is located 50 m downstream from Finca Doña Martina (FDM), on the same calcarenite rock face that structures the left bank of the middle section of the Rambla Perea gorge (Figs. S1.1-S1.2). When seen from the SE, the cliff displays here a wedge-like recess akin to FDM’s, even if smaller and less pronounced. This commonality of pattern suggested the potential existence, buried under slope deposits, of an archeological site formed in the context of a “back-wall-rising-above-horizontal-platform” morphology created by the same processes that operated at FDM: tectonic (joint formation), and erosional (slabbing-off of multi-ton prisms). These expectations were confirmed. The excavation exposed a sub-vertical, nearly plane, inwardly slant, several meters-high back wall, and, opposite, an outer wall formed by the NE-SW-oriented, flat face of a large rock mass (Figs. S4.1-S4.5). Bedrock could be reached over no more than 2 m² adjacent to the back wall, but the existence of an extensive basal platform supporting the stratification is suggested by the fact that the deeper occupation horizons tend to be more substantial and richer as one moves outward (Figs. S4.6-S4.7).

The fill of ADB spans the same time interval as FDM’s but the band of terrain behind the former’s current drip line is only ca.4 m at its widest and no more than ca.10 m-long in total — i.e., it is both smaller and narrower. Compared to FDM’s, however, the post-depositional alteration of the succession is negligible, implying little or no exposure to the elements through the sedimentary accumulation process. From this apparent contradiction, we can infer that significant change occurred in the morphology of ADB since it was first used by humans. Indeed, the multi-ton slabs interstratified in the deposit, which document two major episodes of roof fall (Figs. S4.6-S4.7), bear witness to a process of change in the geometry of this locality. In addition, the many large boulders strewn downslope of the site corroborate that the cliff face receded significantly over time.

Determining how the site originally looked like requires additional excavation. With current evidence, two hypotheses can be entertained. One is that the site once was a true cave and became a rock-shelter only after the breaking-off of the rock mass currently bounding the sedimentary fill to the South. In this scenario, that mass would represent the upper portion, now loose and inward-tilted, of the cave’s original external wall. The parsimonious hypothesis, however, is that the site always was a rock-shelter and that the rock face presently making for an external wall corresponds to the flat bottom of a massive chunk of the overhang — which, formerly, would therefore have protected a much more extensive area. In this scenario, the space between the back wall and the inward face of the tilted collapse would nonetheless have also functioned as a kind of cave — closed to N and E, with an entry facing S, and naturally roofed by the substantial overlap of the two drip-lines (external, at higher elevation, the shelter’s; internal, at lower elevation, the collapsed overhang’s).

The pristine preservation of fireplaces with thick ash deposits across the Aurignacian-to-Solutrean sequence suggests the persistence of cave-like conditions throughout. Conversely, the lesser integrity characterizing the fire features of the Upper Magdalenian could relate at least in part to the site having by then already acquired its extant form.

4.2. EXCAVATION APPROACH AND METHODS

To verify whether an archeological fill did exist subsurface, an initial 2 m² test trench, placed where the band of terrain behind the extant drip line is widest (grid units S/4-5), was opened in the Spring of 2008 (Fig. S4.1A). It was then enlarged over a couple of annual, one month-long field seasons. As the excavation went deeper into the deposit, the trench was stepped to keep the height of its walls within safety parameters (Fig. S4.1B-D). Between 2008 and 2010, an area of ca.6 m² was thus taken down to ca.2.5 m below surface, from where a deep sounding (grid units T/5-6), launched in 2012, eventually reached bedrock (Fig. S4.1E-F). Between August 2013 and November 2014, the gridded area (squares S-X/2-6) was open-area excavated over a total of seven months of field work (Figs. S4.2-S4.3). Eventually, this wider trench also had to be stepped. The size of excavated surfaces thus decreased with stratigraphic depth: from 20 m² in the Magdalenian to 14 m² in the Solutrean and Gravettian, 8-12 m² in the Aurignacian, and only 2 m² in the Mousterian (Fig. S4.4). In 2016, the excavation was extended outward; at the end of the field season, it had reached the Middle Solutrean (Fig. S4.5).

The yellow sandy deposit encountered by the excavation at the base of the dark, Holocene soil extended all the way down to bedrock; only the signature left by the site's successive human occupations allowed stratigraphic subdivision (Figs. S4.6-S4.7). At décapage, this archeological stratigraphy defined by hearth features, soil burning, and/or artefact scatters could be followed with sub-centimeter precision over extensive surfaces. Away from the area in which the anthropogenic signature was strongest, the décapage would be extended laterally along the same plane, using stone lines as an auxiliary instrument. Each of these true paleo-surfaces, plus the thickness of enveloping sediment above, was defined as an "Occupation Horizon" (OH). Intervening deposits found to be sterile or containing scarce, intrusive remains were designated as "Intermediate Levels" (IL).

When OHs were thick or featured multiple, stacked-up occupation lenses, they were subdivided to the extent possible. In the field, each excavated slice ("spit") was numbered sequentially (in the format "A1" to "An"). Even though, in some cases, a one-to-one, spit-to-OH correspondence exists, often each OH is an analytical unit made-up of the addition of two or more spits. Even though the same general approach was followed in the initial phase, the limited size of the trench implied that some OHs could not be recognized, while the delimitation of some that were only marginally represented was imprecise. The integration of the two phases of excavation into a single archeo-stratigraphic scheme used a 3D model in which the paleo-surfaces of 2013-2014 were projected onto the test-excavated grid units using boundary information derived from cross-section records. In the few cases where ambiguity remained as to the assignment of a given test excavation unit to one of the 2013-2014 OHs, the corresponding finds were removed from consideration.

Otherwise, given broadly similar levels of bone and shell preservation, work followed, with a few differences, the methodology described in Chapter 3 for the excavation of FDM. Finds were piece-plotted with the same cut-off criteria, against the site grid and a datum placed on the back wall at the elevation of ca.399 m; from the beginning, however, finds were numbered sequentially, 1-to-n, per year of excavation (e.g., 2008-1 to 2008-n, etc.), and the sediment for sieving was separated per ¼ m² units. In the open-area excavation phase, the <2 mm fraction of two such units was entirely saved for subsequent flotation.

Sediment samples associated with the fire features were taken for phytolith analysis, and charcoal collected in and around them was sampled for biomolecular analysis. Control samples from non-anthropized areas of the same paleo-surfaces were taken alongside.

4.3. STRATIGRAPHIC OUTLINE

4.3.1. Archeo-stratigraphic units

Figs. S4.6-S4.7 illustrate the main cross-sections recorded at the end of the 2012 and 2014 field seasons, respectively. Combined, they represent the horizontal and vertical variation observed across the excavated area of the site.

The radiometric dating results available for the archeo-stratigraphic sequence and the corresponding provenience information and analytical data are given in Tables 2-3 and S4.1, and in Figs. S4.8-S4.9.

From top to bottom, the sequence can be subdivided into the ten main blocks described in the following.

Holocene soil
Grey, pulverulent sediment with abundant vegetal detritus and rare artefacts (wheeled pottery, metal, a few prehistoric sherds). It corresponds to the field unit "layer A."

OH0
Band of brown-grey sediments (field unit "layer B") overlying brown-yellow sands (field unit "layer C") containing small, slab-like clasts. Layers B and C contain scarce stone tools of Epimagdalenian affinities, akin to those retrieved in layer 3 of FDM. Due to animal burrowing and the presence of anthropogenic, pit- and channel-like disturbance features penetrating from the ground surface deeply into the deposit, this horizon's stratigraphically intact areas are of limited extent.

OH1
Band of yellow sands, heavily anthropized, with abundant charcoal and ash, which give it a grey color over extensive surfaces (field unit "layer D"). It contains diagnostic Upper Magdalenian stone tools. Negative features filled with a dark, organic matter-rich sediment containing abundant land snail shell and charcoal traverse the horizon in its entirety. These features correspond to the base of the network of large animal burrows penetrating into the Pleistocene deposit from the overlying Holocene levels (Fig. S4.10).

OH2-to-IL1b

Uppermost part of the yellow sands with clasts of different sizes that, variably anthropized, extend all the way down to bedrock (field unit "layer E"). OH2 is a lithic artefact scatter found at the interface between layers D and E in rows 2-3 of the 2013-2014 open-area excavation. Sample 2008-774, collected in the initial test trench, has the same interface provenience and may well relate to this OH2 occupation. Immediately below OH2, artefacts are still found, downwardly displaced, in bioturbation features. In the excavated area, however, the deposit then becomes virtually sterile until a paleo-surface defined by an eroded hearth feature is reached. Denoted by its imprint (subsurface sediment reddening; Fig. S4.11), this feature and the few associated, undiagnostic artefacts form the OH3 context. The deposit above, between OH2 and OH3, is IL1a (ca.20 cm-thick), the deposit below, between OH3 and OH4 (ca.10 cm-thick), is IL1b. The dating evidence shows that the OH2-IL1b package, even though 35 cm-thick, accumulated very rapidly, in less than one millennium, and, as with OH1 above, entirely within the time frame of the regional Upper Magdalenian.

OH4-to-OH12

Extensively anthropized yellow sands featuring a complex stack of hearth-defined paleo-surfaces (Figs. S4.12-S4.20) that associated artefacts and radiocarbon dating place in the Last Glacial Maximum (LGM). From top to bottom, the assemblages are Early Magdalenian, Solutreo-gravettian, Solutrean and Gravettian. At this time, the site seems to have been visited regularly, with stratigraphic segregation of the different horizons rendered possible by a steady rate of sedimentary accumulation. An erosional hiatus between OH7 and OH8 is suggested by a change in the dip of the paleo-surfaces and by their being in direct contact, with no intermediate sterile deposit, despite more than a thousand years apart. Another erosional episode exists at the interface between OH11 and OH12, which, in the excavated area, are separated by two millennia. Adjacent to the back wall, the existence of this latter hiatus could be suspected based on the amount of syn-depositional, small-mammal burrowing observed at the corresponding elevation (Figs. S4.17-S4.18). Outward, the hiatus manifests itself in the rill that cuts the OH12 deposit and is filled with a dark, organic matter-rich sediment whose nature and origin remain to be established (Fig. S4.19).

IL2-to-OH14

Large collapsed slab, on average ca.50 cm-thick, and sediment fill of the empty space extending to the back wall behind. Based on a microgravette found just below the slab's underside and the radiocarbon date for the small hearth lit against the base of its inward sloping upside (Fig. S4.21), the time of the collapse can be constrained to between the Late Aurignacian deposit it rests upon and the Early Gravettian. That hearth defines an exceedingly poor OH13 occupation. The thickness of deposit between OH12 and OH13 is IL2. It was sterile, except at the very top, where the finds represent downward migration from OH12. The thin deposit comprised between OH13 and the topographic base of the collapse is IL3. OH14 is the wedge of deposit filling the latter's raised butt.

OH15-to-OH20

This package corresponds to ca.75 cm of “layer E” sandwiched between two episodes of major roof collapse. The uppermost horizon, OH15, underlies the artefact-poor and undated OH14, which yielded a microgravette and, therefore, given its position in the stratigraphic sandwich, belongs in the Early Gravettian. Lithic assemblage composition and radiocarbon dating place in the Aurignacian the six horizons discriminated within the OH15-OH20 package, which features well-preserved, spatially extensive fire features (Figs. S4.22-S4.28). Numerous shell beads and, in OH17, even a bone needle, were also found. The dating suggests a hiatus between OH18 and OH17, which accords well with the fact that the OH18 hearths are eroded (Fig. S4.25). OH20 represents the earliest manifestation of the Aurignacian at the site but, in the excavated area, yielded no fire features (Fig. S4.29). This occupation took place once sedimentary accumulation had filled the space behind the collapsed slab atop which its remains are found — levelling the ground surface, that accumulation eventually provided a sub-horizontal platform amenable to settlement.

IL4

Large slab, with an average thickness of ca.90 cm, plus the sedimentary fill of the space extending behind. The slab represents a roof-collapse episode post-dating the last Mousterian occupation recorded in grid units T-U/6-7. Through the accumulation of IL4, the narrow space (Figs. S4.29-S4.30) that remained between the back wall of the shelter and the inward-facing upside of the roof-collapsed slab was not amenable to human use. Thus, as with the few items retrieved in the other ILs, the IL4 lithics must reflect downward migration from the overlying occupation — as indeed otherwise proven by the refits linking them to the OH20 stone tool assemblage.

OH21-to-OH23

Basal occupation levels, spanning a thickness of ca.75 cm, comprised between bedrock and the base of the IL4 collapse (Fig. S4.31). The fire-reddened lenses observed during excavation allow subdivision of this deposit into three horizons. The subdivision remains tentative and is bound to be revised once the excavation is able to proceed outward and explore the sediments under the collapse.

Saprolite

Thin level of clast-supported, angular breccia resulting from the degradation of bedrock under sheltered conditions (Fig. S4.31; see Chapter 3 for a similar unit at the base of FDM).

4.3.2. Radiometric dating

The provenience and composition of the samples submitted for radiocarbon dating, all of charcoal, is given in Table S4.1. The results obtained are listed in Table 2. The *Olea* sample comes from one of the burrows penetrating layer D. It was submitted to test whether the taxon was regionally present in Tardiglacial times or, as suspected, represented post-depositional intrusion. This sample was dated at the Oxford laboratory (OxA). The other results are all AMS dates run at the VERA facility, University of Vienna.

All but three of the submitted samples were successful. The exceptions are 2012-1518, 2014-3129, and 2014-3421, for which the ABA treatment proved too aggressive. They concern charcoal taken from the core of fire features, which may well explain the failure, as samples of similar age collected in the periphery of the same or identical features turned out to be wholly unproblematic. Nevertheless, the humic acids result for sample 2014-3421 seems to represent a reliable age estimate, as it is statistically indistinguishable from the results obtained for the other, successful OH19 sample (2014-3348). The other two unsuccessful samples' humic acids results are clear underestimations, as shown by their comparison with the date obtained on the successful sample for the same OH17 unit (2014-3184). Similar underestimations are apparent when comparing pairs of carbon and humic acids results obtained on other samples (e.g., from OH12, OH20, IL4). Therefore, all humic acids results have been excluded from consideration and are not included in the Fig. S4.8 plot. The fact that the magnitude of the underestimation falls within the uncertainty of the results obtained for the Mousterian occupation horizons probably explains why the effect is not apparent in those cases.

The reliability of the successful ABA dates is supported by their agreement with the ABOx results for subsamples of the same charcoal (Table 2). Optically Stimulated Luminescence (OSL) dating of the associated sediment corroborates the robustness of the site's radiocarbon chronology (Table 3; Figs. 6-8, S4.9). For the Mousterian, the OSL dates from immediately under the base of the overlying collapse, in OH21, and for OH23, 30 cm above bedrock, constrain the accumulation to the ca.51.5-57.7 ka (thousands of years) interval — in agreement with the *terminus ante quem* provided by the radiocarbon dating to 40.8-42.4 ka of a charcoal sample taken in the overlying IL4 deposit. Likewise, radiocarbon places the OH17 and OH18 horizons in the 34.2-35.6 and 34.9-37.0 ka intervals, which are encompassed by those obtained via OSL for the same units (28.8-36.4 and 30.2-41.4 ka, respectively).

4.3.3. Culture-stratigraphic sequence

Data on the stratigraphic distribution of personal ornaments and stone tool types found across the ADB stratigraphic sequence, grouped by major occupation phases, are provided in Tables S4.2-S4.3. The vertical variation in the presence of the mollusk taxa used in the manufacture of shell beads is plotted in Fig. S4.32. Representative samples of the stone tools from the mid- and later Upper Paleolithic occupation horizons and of the ochred and perforated shell finds made at the site are illustrated in Fig. S4.33 and Fig. S4.34, respectively. Preparation and analysis of the large artefact collections being a work in progress, the counts in Tables S4.2-S4.3 are bound to be modified, for instance via the inclusion of the sieve fraction. So far, this fraction has been processed for the basal levels (Early Gravettian, Aurignacian, and Mousterian) only.

For Upper Magdalenian horizon OH1 (Fig. S4.33, nos. 1-4), counts based on the 122 formal tools collected 2008-2009 have already been published (Zilhão et al., 2010). The finds made since multiply that total but do not change the structure of the assemblage. Marine shell beads are present in OH2 but, so far, absent from OH1, where the meaning of an ensemble of spatially clustered, unperforated *Melanopsis* river shells from grid units X/5-6 remains elusive.

The Early Magdalenian is represented in OH4, dated to ca.18.6 ka on a hearth-associated sample (Fig. S4.12). The assemblage is small and almost exclusively made-up of backed and very small, marginally backed bladelets (Fig. S4.33, nos. 5-6).

Lucena et al. (2012) provide preliminary data on the Solutrean sequence. The Solutreo-gravettian (Figs. S4.13-S4.14; Fig. S4.33, nos. 7-8) is represented in horizons OH5 and OH6, dated to the 19.8-21.3 ka interval. The dominant tool-types are backed and/or pointed microliths; excluding retouched piece fragments, such items represent 46% of the total. An unifacial foliate was recovered in two fragments, distal and proximal, that join minimally at the break. So far, this is the only recovered item evoking the Solutrean ancestry of the technocomplex.

The Upper Solutrean is represented in horizon OH7 (Fig. S4.15; Fig. S4.33, nos. 9-13), dated on a hearth-associated sample to ca.23.2 ka. It yielded a small number of shouldered points associated with backed and marginally backed bladelets in an industrial context dominated by endscrapers. No evidence of bifacial thinning was found. A small (basal?) fragment of an unifacial foliate point dorsally featuring the covering, flat retouch typical of the Solutrean is tentatively classified here as a willow-leaf. The Middle Solutrean is represented in horizons OH8 and OH9, dated on hearth-associated samples to 24.3-25.2 ka (Fig. S4.16). In agreement with their position in the sequence and regional chrono-stratigraphic patterns, these horizons yielded a few bifacial thinning flakes and a bifacial foliate fragment.

Lower Solutrean assemblages featuring a total of 24 unifacial points— 21% of these occupations' retouched pieces (fragments excluded) — were recovered in OH10 and OH11, dated on hearth-associated samples to 25.0-25.5 ka (Figs. S4.17-S4.19; Fig. S4.33, nos. 14-17). Aside from the Upper Magdalenian, these occupations were the site's densest and yielded the most retouched tools (despite the material from the rill in row 3, where obviously reworked Gravettian items were mixed in, not having been counted). The few backed bladelets and burins on truncation may also derive from the underlying Gravettian, as they all come from peripheral areas at the interface between OH11 and OH12 that had been disturbed by small mammal activity. Otherwise, such activity also resulted in Middle Solutrean charcoal — sample 2012-178 — being present in the fill of an OH12 burrow (see above and Tables 2 and S4.1).

Combined, the Solutrean and Solutreo-gravettian yielded most of the sequence's shell beads, including all the *Tritia neritea* and *Acteon tornatilis* specimens, a few of which were unperforated (Figs. S4.32, S4.34). A piece of red coral, *Dentalium* tubes, and other small, marine and fluviatile gastropods round-up this assemblage, which contrasts with the underlying Gravettian in the absence of perforated bivalves (a small fragment tentatively assigned to *Glycymeris* being the single possible exception).

The Middle Gravettian (Fig. S4.33, nos. 18-19) is contained in OH12, dated on a hearth-associated sample to 27.4-27.9 ka (Fig. S4.20). Even though endscrapers made on rather large blanks dominate, the stone tool assemblage features the characteristic association of backed or marginally backed bladelets with (numerous) burins on truncation.

The Early Gravettian, Aurignacian and Mousterian occupations of ADB date beyond 31 ka. They are spread across eleven Occupation Horizons (OH13 to OH23) situated beyond the base of the uppermost of the two large, collapsed roof-slabs inter-stratified in the “layer E” sands. These horizons substantiate the transition from the Middle to the Upper Paleolithic in the region. Their artefact assemblages are presented and discussed below in greater detail.

4.3.4. Site formation

The preservation of undisturbed fire features bespeaks of the exceptional integrity of most occupation horizons within “layer E.” Small scale bioturbation affected limited areas of the excavated surfaces, and can be expected to have caused some degree of post-depositional displacement of finds — mostly of small items brought up by burrowing that ended-up mixed on extant ground surfaces with the remains of later, coeval with the time of burrowing, occupations. Otherwise, when assessing the potential presence of intrusive or inherited material among the remains excavated from any given horizon, the only processes one needs to bear in mind are “wall effect,” décapage error, and palimpsest formation (the latter, when dealing with material either side of the few hiatuses of erosion and/or sedimentation identified in the sequence).

The distribution of phytoliths across the different lenses that make-up a given OH provides independent corroboration that, where the artefact content of the deposit is concerned, an exceptionally high degree of assemblage integrity ought to be expected. The results obtained for samples taken in exposed cross-sections and spanning the micro-stratigraphy of each OH over thicknesses of 2 to 5 cm reveal a very clear pattern: (a) high concentrations in the white lenses; (b) low concentrations in the underlying black lenses; and (c) zero or near-zero values in the reddened sediment below as much as in the yellow matrix sandwiching the fire-altered deposit (Fig. S4.35). The FTIR (Fourier Transform Infrared) spectroscopic analysis of the white lenses shows that they are formed of ash entirely generated by the burning of wood (not bone or grass). Conversely, the absence of such a white lens (and, hence, of phytoliths), as in Column F (Fig. S4.35), coincides, in plan view (Fig. S4.24), with unstructured surfaces whose above-ground fire features were not intact.

These phytolith data fully support the geoarcheological interpretation of the micro-stratigraphic organization of the different OH lenses developed in the course of excavation. The black lenses represent the ground fire was lit on. Their geometry is therefore a proxy for the topography of the surface at the time of occupation — that upon which the associated remains were discarded. The red lenses represent the immediate subsurface sediment — the color change results from chemical alteration of the iron component, brought about by the high temperatures reached in the fireplaces. These lenses may contain some trampled-in material related to the occupation above. The white lenses are an anthropogenic deposit consisting of the ash formed by the burning of the fuel used in the fireplaces. Finally, the yellow sediment covering each of the white-black-red ensembles of lenses corresponds to the material, principally derived from the degradation of the shelter’s walls, that eventually buried the occupation.

When the preservation of the fire lenses is pristine or near-pristine, it can be safely inferred that burial occurred very rapidly. Thus, in such cases, the artefact component of the yellow deposit capping a given package of fire-altered lenses can be considered of the same general archeological age, if not necessarily pertaining to the same occupation event. That component could represent, for instance, a scatter of finds produced during subsequent visits, ones during which the fire-related activity would have taken place in parts of the site located outside of the excavation trench. Even so, from a technological and cultural-stratigraphic perspective, no issues of assemblage heterogeneity arise from the conflation of such material with that found on the exact paleo-surface defined by a hearth or an extensive black lens.

In plan view, the fire-altered areas of the surface of a given OH are sometimes reduced to no more than a homogeneous red stain with a spatter of associated charcoal, or to a mottled mix of red, black and white. This lesser degree of preservation bespeaks of significant exposure to erosional agents, resulting in small-scale redistribution of both sediments and artefacts due to wind, surface dynamics, and/or animal activity. Except for the rill at the interface between OH11 and OH12, no evidence was found that run-off, or other types of water-related modifications, acted on the deposit in any significant manner. It is therefore likely that the eroded fire lenses reflect longer periods of exposure rather than exposure to stronger agency.

Radiocarbon dating further shows that OHs featuring some post-depositional disturbance of the original micro-stratigraphic structure tend to be separated from overlying, better preserved ones by measurable amounts of time. This is consistent with a reduction in the rate of sediment accumulation, representing (or including) actual hiatuses during which little or no deposition occurred. Two good examples of this pattern are OH18 (Fig. S4.25) and OH6 (Fig. S4.14). OH18 corresponds to a thin lens of eroded, fire-related sediment separated from the base of overlying OH17 by <5 cm of sediment but one millennium of time. OH6 is separated from overlying, statistically younger OH5, by an equally thin slice of deposit but itself yielded two statistically distinct dates. This dating evidence suggests that OH6 is a palimpsest corresponding to the horizontal redistribution and vertical compression of at least two different occupation events separated by as much as five centuries. In contrast, note how the dating of the ca.20 cm-thick package of well-preserved, stacked-up fire lenses making-up OH15 and OH16 yielded statistically indistinguishable results (Table 2).

4.4. THE HORIZONS OF THE TRANSITION

4.4.1. Lithic taphonomy

Systematic intra- and inter-level refitting was carried out for the finds made in the OH18-IL4 sequence, in order to (a) validate the expectation that the stone tool assemblages recovered within OH19 and OH20 ought to be characterized by an exceptionally high degree of stratigraphic integrity, and (b) test the interpretation of the artefact component of IL4 as representing downward migration from OH20. The analysis focused on a bioclastic flint variety that, even though present through the sequence, is especially abundant in these horizons and could be traced to a primary source located 12.7 km to the East.

Sixteen “nodule” units were discriminated, of which M and N almost certainly correspond to a single initial volume while L and Z almost certainly do not (Table S4.4). Nodule M did not refit, but six of the ten items assigned to it came from OH20 and four from IL4. The successful refits further substantiate the connection between these two archeo-stratigraphic units: of the 20 items that went into the five refitting units obtained, 16 came from OH20 and three from IL4. One, however, came from OH18. It belongs to Refit 5 in nodule N, which joins 2014-3596a, a Siret accident from OH20, with 2012-1303, a flake from OH18 (Fig. S4.36). A non-refitted small flake fragment is the other nodule N item, and it also came from OH20.

The OH18-OH20 connection documented by Refit 5 is at odds with the integrity of the intervening OH19 horizon, documented by the pristine preservation of its fire features (Figs. S4.25-S4.28). Note, however, that all the items in Refit 5 are sieve finds, which are not processed and logged until after the end of the corresponding season’s field work (and whose coordinates are approximate — to the center of the quadrant of provenience and the mid-point of the spit’s thickness). The 2012-1303 item was retrieved in the field season of August-September 2012, during which the T/5-6 test trench reached IL4 to a depth of ca.525 cm below datum. Therefore, it cannot be excluded that its assignment to OH18 derives from labelling error, for instance, a misreading of the spit information given in the accompanying label as “a45” (OH18) instead of “a55” (IL4). Alternatively, if the provenience data associated with the inventory number are correct, the object may have been brought up by the kinds of “wall effects” that one can expect to have been in operation in the NW quadrant of grid unit T6 (cf. Fig. S4.5 for the vertical reach of such effects along the site’s external wall).

Excluding the OH18 item in Refit 5, only nine other pieces (4% of the total, but only 0.1% by mass) were found above OH20 (Tables S4.5-S4.6). These counts exclude the items indicated as from “OH19/20” because they correspond to sieve material from grid unit T6, excavated to this depth as part of the 2012 deep sounding. Likely, this material derives from OH20 but its precise correlation with the OH stratigraphy of 2013-2014 could not be carried out; hence, the uncertainty expressed by their provenience designation.

Of the nine other bioclastic flint items analyzed whose stratigraphic position we can be confident about, seven are from those “nodules” (L and Z; see above) that are almost certainly made up of material from different initial volumes. As to issues of potential post-depositional displacement, their OH provenience is therefore uninformative. The other two items are from nodule J, which yielded 50% of the refits, all concerning material from OH20 and IL4. These two items are therefore likely to represent indeed upward displacement into: OH18, for a 0.5 g sieve bladelet from the southern half of grid unit U3; and OH19, for a 0.5 g chip from the NE quadrant of T6. Given their position in the trench, the causes underpinning the displacement must lie in (a) the same kinds of wall effects possibly responsible for the Refit 5 anomaly, in the case of the piece from T6, and (b) the small-scale burrowing identified in grid units T-U/3 between the base of OH17 and the base of OH20 (Figs. S4.24-S4.25, S4.28-S4.29), in the case of the piece from U3.

Among the flint artefacts retrieved in OH20 and IL4, the bioclastic variety used in the lithic taphonomy study represents 43% (192 out of 451), by number, and 80% (858 out of 1075 g), by mass. Given the representativeness of the sample and the insignificance of the anomalies, we can therefore derive from the data concerning the basal Aurignacian levels analyzed a robust inference for the whole of the sequence: that an assumption of assemblage integrity is, more than warranted, mandatory. Bearing in mind the phytolith data, this is even more so when fire features are well preserved. The fact that all other 15 refit units obtained so far — in OH17, OH16 and OH15 — link items from the same horizon, often from the same excavation spit, further strengthens the inference.

4.4.2. Mousterian

The basal Mousterian in OH21, OH22 and OH23 dates beyond 44 ka. Judging from the Aurignacian pattern, the main area of habitation must have been located 2-3 m outward of the grid units located against the back wall in which bedrock was reached and the Mousterian could be excavated. The limited size (2 m²) and marginal position of this excavation explains the small size of the assemblages (Tables S4.7-S4.8) and precludes a firm conclusion on the nature of the occupations' stone tool economics. A few preliminary observations can nevertheless be made.

Cortex was present in 30% of the flints (25 out of 83, debris and unretouched flake fragments excluded), suggesting the introduction of unmodified nodules. That some knapping did occur at the site is otherwise attested by two core-trimming elements and a Kombewa core. The latter is a cortical blank whose ventral surface was centripetally exploited to produce small flakes; the largest scar is no more than 18.5 mm long. Three sidescraper re-sharpening elements document a local consumption history for these kinds of items.

The core-trimming elements concern the correction of hinges and/or platform angles in the course of core reduction using the Discoid method. Of the 38 items of débitage and formal retouched tools for which a technological reading was possible, most (ten) also reflected use of that method. The Kombewa and Levallois methods are represented by five specimens each. Among the latter there is a double denticulate on quartzite discarded after breakage that, given this raw-material's poor representation in the assemblage, is mostly certainly an imported item (Fig. S4.37, no. 1).

The range of formal retouched tools is limited to notches, denticulates (Fig. S4.37, nos. 1, 6) and sidescrapers (Fig. S4.37, nos. 2-5). A cortical, orange-segment flake might be added to this ensemble as a possible naturally backed knife but it bears no evidence, macro- or microscopic, of having been used. Ditto for three unmodified Levallois products — two small points and a typical preferential flake. The use-wear analysis, however, showed that some unretouched or atypically retouched pieces had in fact been functional tools; most were used on wood, but defleshing is also documented (Table S4.9; Fig. S4.38).

4.4.3. Aurignacian

Like those of Solutrean and Gravettian age, ADB's Aurignacian occupation horizons yielded many shell beads, but the range of taxa is broader and includes species so far unknown in the later occupations of the site (Table S4.2). *Theodoxus fluviatilis* (Fig. S4.34) is the most abundant taxon. A small *Gibbula*, possibly *G. rarilineata*, is represented in OH20/IL4 by unperforated specimens that are heavily ochre-stained, as are the *Theodoxus* (whether perforated or not). All the *Striarca lactea* are also from OH20 and some are ochre-stained, so they may have been collected for ornamental purposes. Ditto for the ochre-stained *Mimachlamys* fragments found in OH17; despite the lack of a perforation in the shell, or part thereof, that we have, these valves may have been used as pendants. The function of the few fragments of *Pecten* and other bivalves remains elusive; as with the numerous such remains found in the Gravettian and Solutrean, it is inferred that they were introduced to the site as complete shells, possibly used as containers.

The stone tool assemblages (Tables S4.10-S4.21; Figs. S4.39-S4.43) are diagnostic, even when their size is small, as in the case of OH19's, which consists of only 146 pieces, 76% of which are debris (60%) or flake fragments (16%). Among the flints (debris excluded), blades and bladelets, both retouched and unretouched, account for as much as 33% of the assemblage in OH20, 15-22% in OH19-OH16, and 48% in OH15; they are virtually non-existent in the underlying Mousterian, which yielded a single bladelet in OH22, possibly post-depositionally displaced from IL4 via "wall effect" processes (Table S4.7). Conversely, among cores, no Levallois, discoid or Kombewa types — represented in the Mousterian deposit by discarded volumes, preparation byproducts, and diagnostic debitage — were found in the Aurignacian horizons. In the latter, all cores are either of the regular, prismatic kind or correspond to specialized types (splintered pieces, "burins," and carinated or nosed "scrapers") that were set-up, or expediently used for the extraction of bladelets only.

The technological information encoded in the refits, cores and débitage from OH20/IL4 allows us to reconstruct two reduction strategies (Figs. 11, S4.39-S4.40). One targeted the production of blade-size blanks from elongated nodules set-up as single platform cores; the platform is prepared by abrasion, the opposite end of the prism is configured to maintain the convexity of the débitage surface, and the desired end-product is a 5-10 cm-long, 1-3 cm-wide blade. The second strategy is designed for the extraction of bladelets from carinated/nosed "scrapers," and is carried out in two steps. The first step is the extraction of the blanks — thick, elongated, "naturally carinated" blades. The second step is the production of bladelets from the "scraper fronts" configured on the blanks obtained in the first step.

The evidence at hand indicates that the two strategies were used separately, not sequentially. In the examples that we have, the production of "scraper" blanks used dedicated nodules/volumes that were routinely discarded once found unfit for purpose. It cannot be excluded, however, that the production of such blanks could also have been carried out on byproducts of the initial stages of configuration and preparation of regular blade cores.

From a functional standpoint, the basal occupation in OH20/IL4 is significantly distinct from those that follow. This is well apparent in the total mass of the flint assemblage retrieved: 1076 g, of which 510 g (47%) correspond to cores. The total flint mass in OH16 (1310 g) is higher, and that in OH17 (830 g) approaches the OH20/IL4 values. However, the cores retrieved in OH16 and OH17 represent no more than 13% and 19%, respectively, of the total flint mass therein. Core size and core type also differ markedly: prismatic cores for blades are 4 out 7 in OH20/IL4, but 2 out 24 in OH16 and non-existent in the other Aurignacian horizons; and the average mass of cores is 73 g in OH20/IL4 but ranges between 3.6 and 7.6 g only in the other horizons (excluding OH15, in which the average of 14.8 g is not significant because it corresponds to only two specimens). These differences are the more significant because a succession of dense occupation lenses featuring multiple, stacked-up fire features are subsumed in OH17 and OH16.

In the OH19-OH15 sequence, therefore, available flint volumes would seem to have been much more intensively reduced. This inference is corroborated by the values concerning the proportion of each flint assemblage represented by chippage and chunks: by mass, 6% in OH20/IL4, contra between 17% (in OH19) and 35% (in OH17) for the overlying horizons. Conversely, these horizons yielded significant volumes of locally available raw-materials (cf. their large flakes and cores made on limestone), while only three limestone items, in total 1.1 g-worth, were retrieved in OH20/IL4.

These stone economics data suggest that the OH20/IL4 occupation(s) were of short duration. Likely, we are dealing with transient stays during which flint volumes collected en route were processed for the extraction of quality blanks taken away by the individual(s) doing the knapping, and whereby volumes found to be unsuitable or whose set-up failed were discarded with no further labor investment. This interpretation accords well with the lack of fire features in OH20, which is in stark contrast with the number and preservation of those found in overlying horizons.

These comparisons suggest that OH19-OH15 were characterized by lengthier stays during which flint volumes brought in as ready-made blanks or finished tools were systematically recycled prior to discard. The decrease in size seen in similar core types as one moves up in the sequence further supports this interpretation. For instance, while the two carinated “scrapers” in OH20 weighed between 13 and 48 g at discard, the corresponding values for the carinated and nosed “scrapers” in OH17, OH18 and OH19 range between 2 and 7 g (e.g., item 2014-3424, Fig. S4.42, no. 2, the mass of which is 3.2 g). The fact that the core assemblages in OH17 and OH16 are dominated by splintered pieces/bipolar cores with masses comprised between 0.4 g (item 2014-2481-1, Fig. S4.43, no. 1) and 7 g is also consistent with a flint economy whereby extracting as much cutting edge as possible from any given volume of raw-material was routinely attempted prior to discard. Indeed, out of 108 bladelet-like blanks in these two horizons, no less than 28 bore diagnostic technological attributes of extraction from splintered pieces/bipolar cores (Aubry et al., 1997) and one such blank, badly burnt, had been retouched into a Dufour bladelet (Fig. S4.43, no. 8).

Reduction-to-exhaustion of imported blanks and tools must underpin two other aspects of La Boja's Aurignacian assemblages. The first aspect is the dearth of endscrapers. Across all horizons, and excluding the type-list items that, in fact, are cores, this category is represented by only 6 out of 69 retouched tools: two small, thumbnail-like; one simple-atypical; and three fragments (Tables S4.10-S4.21). The second aspect is the low success rate in the identification of use-wear. Traces were identified in 14% (10 out of 72) of the retouched and unretouched blanks analyzed, in contrast with the 28% (11 out of 40) success rate encountered in the Mousterian (Table S4.9). Both aspects probably relate to intensified reduction in that (a) endscrapers that broke during use or could no longer be re-sharpened may have supplied a significant proportion of the blanks reduced as splintered pieces/bipolar cores, and (b) the recycling of tools may have entailed the loss of the active parts bearing evidence of the uses they had been put to.

In this context, it is probably to be expected that three out of the six pieces on which diagnostic use-wear could be identified are projectile elements (Fig. S4.41, nos. 1-3). The other tasks that left identifiable traces are the processing of wood, hide, and hard animal tissue (Fig. S4.41, nos. 4-5).

Economic and functional considerations — degree of spatial segmentation of the reduction sequences, and intensity of raw-material recycling — therefore suffice to explain the differences in composition, structure and metric attributes observed when comparing the assemblages retrieved in OH20/IL4 with those from the overlying Aurignacian horizons. From a typological standpoint, however, OH16 seems to represent a significant divide. This horizon marks the first appearance in the sequence of backed bladelets featuring a particular mode of abrupt retouch (Fig. S4.43, nos. 4-5) — short and limited to the very edge, not, as in the typical backed bladelets found in the overlying Gravettian, Upper Solutrean and Magdalenian, invasive and eliminating a significant portion of the blank. Documenting typological continuity with the preceding occupations, these "short-backed" bladelets from OH16 and OH15 are associated with the same kinds of retouched bladelets that make up the totality of the microliths in OH17, OH18, OH19 and OH20/IL4: Dufour, and marginally backed (Fig. S4.43, nos. 6-9). Based on this typological evidence, La Boja's Aurignacian sequence can therefore be subdivided into: (a) an Evolved Aurignacian, dated to between 35 and 37 ka and represented in OH20/IL4, OH19, OH18 and OH17; and (b) a Late Aurignacian, dated to around 35 ka and represented in OH16 and OH15.

The number of bladelet blanks bearing diagnostic features of extraction from a "burin" type of core that we see in OH15 may stand for another element of technological innovation. We cannot exclude that assemblage size and sampling bias are involved in the pattern to some extent. However, such "burin spall" blanks, non-existent in OH20/IL4 and in OH19, account for as much as 15% of all bladelets in OH15. Conversely, OH15 yielded no bladelets bearing diagnostic features of extraction from a carinated/nosed "scraper," whereas such blanks amount to as much as 24% of all bladelets in OH20/IL4 and to as much as 38% in OH19.

These Late Aurignacian assemblages may well represent, therefore, the beginnings of a trend that will eventually give rise to the Gravettian, one whereby (a) "burins" replace carinated/nosed "scrapers" as the primary type of specialized bladelet core, and (b) backed bladelets replace Dufours as the preferred type of lithic component arming composite projectile points made of wood or antler. The last steps of such an Aurignacian-to-Gravettian transition, however, would seem to be unrepresented at the site. This is probably due to the existence of a hiatus of deposition or occupation, if not erosion, at the interface between OH15 and OH14 — as indicated by the three millennia that, despite a stratigraphic distance of no more than ca.15 cm, minimally separate OH15 from OH13 (Fig. S4.7).

4.4.4. Early Gravettian

The Early Gravettian in OH13/IL3/OH14 (Tables S4.22-S4.25) dates to 30.9-31.5 ka and is very poor. The scarcity of finds clearly relates to the site (or at least this part of the site) having become unsuitable for habitation. Indeed, at that time, much of the area sampled by our trench was occupied by the major roof-collapsed slab that fell on top of the OH15 ground surface, with attendant implications for human use of the place. Yet, it remains significant that even such a small assemblage as that retrieved in OH13/IL3 (35 items in total, 14 only excluding debris) yielded sufficient evidence — a burin, and three bladelets, two of which refit — to allow cultural-stratigraphic assignment in general technological terms (to the Upper Paleolithic). Specific technocomplex attribution (to the Gravettian) is made possible by the substantial basal fragment of a microgravette point present in the similarly small assemblage from OH14 (82 items in total, 22 only if debris are excluded).

4.5. CONCLUSIONS

The excavation of the La Boja rock-shelter provided replication, with much-enhanced precision and resolution, of the main conclusions derived from the study of the adjacent site of Finca Doña Martina. The following is a summary of the key points.

- Continued occupation of the region through the Upper Pleistocene, irrespective of climatic oscillations, is implied by a radiocarbon dating record whose gaps would seem to correspond, with present evidence, to (a) sedimentary hiatuses (as indicated by direct stratigraphic contact between occupation horizons separated by up to three millennia), or (b) lateral variation (of occupation emplacements relative to the excavation trench).
- The classical tripartite subdivision of the Solutrean, derived from the pattern of index fossil succession observed in such thick, stratified sequences as Laugerie-Haute, in France, or Parpallò, in Spain, questioned by some since Straus's (1983) proposition of a functional interpretation of that variability, is fully supported by the La Boja evidence. In particular, the real entity of a Lower Solutrean phase defined by the presence of unifacial foliate points and the absence of laurel-leaves is confirmed.
- There can be no question that the assemblages retrieved in the OH15-OH20 horizons belong in the Aurignacian technocomplex, with associated dates placing its first presence at the site in the middle of the 37th millennium BP, at the latest.

- The Aurignacian in OH20 is in total technological and typological discontinuity with the underlying Mousterian in OH21-OH23. As a minimum of six millennia separate OH20 from OH21, this evidence does not suffice to exclude the possibility that truly “transitional” systems (in the sense of Kuhn, 2003) may have existed in the region at some point during that missing interval. However, a similar discontinuity is observed at Finca Doña Martina, and elsewhere in Western Europe the Middle and the Upper Paleolithic are discrete technical packages with no overlap. This pattern makes it possible to securely diagnose even very small assemblages on the basis of tool typology or the presence of technological traits that have true index fossil value. For instance, the evidence shows that the presence of sidescrapers, of discoid/Kombewa/Levallois cores and débitage products or byproducts, or of denticulates made on such blanks, suffices to attribute an assemblage to the Middle Paleolithic. Conversely, the presence of blades, bladelets or prismatic/”burin”/carinated “scraper” types of cores suffices to attribute an assemblage to the Upper Paleolithic. Indeed, based on such criteria, under blind testing conditions (i.e., devoid of any contextual information on dating or position in a stratigraphic sequence), an expert lithic analyst would have correctly assigned to either the Middle or the Upper Paleolithic even the smallest of the assemblages retrieved in La Boja’s occupation horizons.
- The use-wear evidence shows that the same types of tasks were being carried out at the two Rambla Perea sites across the period of the transition. Therefore, putative differences in site function cannot explain the sharp technological differences underpinning the assignment of assemblages to either the Middle or the Upper Paleolithic. Instead, it is in the changes that occurred in the technical system that may reside the explanation for why a given activity, e.g., wood-working, was executed with sidescrapers and denticulates in the Mousterian but with only perforators, notches and atypically edge-retouched blanks in the Aurignacian. Only such technological changes can explain why the sets of tools one could choose from when assessing suitability for a given task were so conspicuously different.

4.6 REFERENCES

AUBRY, Th.; ZILHÃO, J.; ALMEIDA, F.; FONTUGNE, M. (1997) — *Production d’armatures microlithiques pendant le Paléolithique supérieur et le Mésolithique du Portugal*, in BALBÍN, R.; BUENO, P. (eds.) — «II Congreso de Arqueología Peninsular. Paleolítico y Epipaleolítico», Zamora, Fundación Rei Afonso Henriques, p. 259-272.

KUHN, S. L. (2003) — *In what sense is the Levantine Initial Upper Paleolithic a “transitional” industry?* in ZILHÃO, J.; D’ERRICO F. (eds.) — «The Chronology of the Aurignacian and of the Transitional Technocomplexes. Dating, Stratigraphies, Cultural Implications», Lisboa, Trabalhos de Arqueologia 33, Instituto Português de Arqueologia, p. 61-69.

LUCENA, A.; MARTÍNEZ, S.; ANGELUCCI, D. E.; BADAL, E.; VILLAVERDE, V.; ZAPATA. J.; ZILHÃO, J. (2012) — *La ocupación solutrense del abrigo de La Boja (Mula, Murcia, España).* «Espacio, Tiempo y Forma. Serie I, Nueva época. Prehistoria y Arqueología», 5, p. 447-454.

SONNEVILLE-BORDES, D. de; PERROT, J. (1954-1956) — *Lexique typologique du Paléolithique supérieur*. «Bulletin de la Société Préhistorique Française», 51, p. 327-335; 52, p. 76-79; 53, p. 408-412, 547-559.

STRAUS, L. G. (1983) — *El Solutrense vasco-cantabrico: una nueva perspectiva*, Madrid, Centro de Investigación y Museo de Altamira.

WoRMS Editorial Board (2016) — *World Register of Marine Species*. Available from http://www.marinespecies.org at VLIZ. Accessed 2016-07-25. doi:10.14284/170

ZILHÃO, J. (1997) — *O Paleolítico Superior da Estremadura portuguesa*, 2 vols., Lisboa, Colibri.

ZILHÃO, J.; ANGELUCCI, D.; BADAL, E.; LUCENA, A.; MARTÍN, I.; MARTÍNEZ, S.; VILLAVERDE, V.; ZAPATA, J. (2010) — *Dos abrigos del Paleolítico superior en Rambla Perea (Mula, Murcia)*, in MANGADO, X. (ed.) — «El Paleolítico superior peninsular. Novedades del siglo XXI», Barcelona, Universidad de Barcelona, p. 97-108.

Table S4.1. ADB. Provenience of the radiocarbon dated samples. All samples are wood charcoal fragments (trench-collected, unless otherwise stated). Coordinates are in cm (elevations are below datum). For the intrusive charcoal collected in burrow features, the chronological horizon given reflects the age obtained for the sample, not the stratigraphic position of the surrounding, intact deposit

Horizon	Sample	Grid unit	x	y	z	Taxon	Sample observations	Lab #
**Holocene intrusion in OH1**								
burrow	2008-775	T5-SE	75	25	205	*Olea europaea*	0.09 g; highly fused and crystalized; xyz approximate	OxA-20116
**MAGDALENIAN**								
OH1	2010-27	U5-SE	75	25	208	*Juniperus* sp.	single, large charcoal fragment; xyz approximate	VERA-5363
OH1/OH2	2008-774	T5-SE	75	25	211	*Pinus nigra*	0.15 g subsample dated; xyz approximate	VERA-5212a
OH3	2013-868	S4-NE	73	55	227	*Pinus nigra/sylvestris*	1.47 g; single, very large charcoal fragment	VERA-5937
OH4	2014-846	T4-NW hearth	16	69	254	*Juniperus* sp.	single, large charcoal fragment	VERA-6080
**SOLUTREO-GRAVETTIAN**								
OH5	2012-385	U5-SW hearth	13	10	273	*Juniperus* sp.	single, large charcoal fragment	VERA-5788
OH6	2010-183	T5-SE	75	10	274	*Juniperus* sp.	vial 1; 0.07g; xyz approximate	VERA-5364a
						Juniperus sp.	vial 2; xyz approximate	VERA-5364b
**SOLUTREAN**								
OH7	2010-225	T5-SW	3	46	295	*Juniperus* sp.	large fragments, can be combined	VERA-5365
OH9	2014-1270	S4-SE hearth	85	36	306	*Juniperus* sp.	13 growth rings	VERA-6081
	2012-1522	S5-W hearth	2	7	300	*Juniperus* sp.	31 straight, wide growth rings	VERA-5850
OH10	2010-316	T5-SE hearth	84	17	316	*Juniperus* sp.	large fragments, can be combined	VERA-5366
OH11	2008-760	S5-SE	75	25	323	*Juniperus* sp.	another subsample failed ABOx at OxA; xyz approximate	VERA-5213
	2014-2578	U4-NE hearth	53	94	328	*Juniperus* sp.	single, large charcoal fragment	VERA-6152
**Solutrean intrusion in OH12**								
burrow	2012-178	T5-SW	25	25	331	*Juniperus* sp.	20 straight, wide growth rings; xyz approximate	VERA-5851
**GRAVETTIAN**								
OH12	2012-175	T5-SE hearth	56	18	335	*Juniperus* sp.	18 straight, wide growth rings	VERA-5852
OH13	2012-622	T5-NE hearth	99	54	384	*Juniperus* sp.	single, large charcoal fragment	VERA-5789 **(a)**
**AURIGNACIAN**								
OH15	2014-2903	U5-SE hearth	92	27	420	*Juniperus* sp.	single, large charcoal fragment	VERA-6153
OH16	2014-3046	U4-NW hearth	13	90	440	*Juniperus* sp.	single, large charcoal fragment	VERA-6154
OH17	2012-1518	T5-S	36	40	436	*Juniperus* sp.	wood knot; small, clean, well preserved	VERA-5853
	2014-3129	U6-SW hearth	10	10	426	*Juniperus* sp.	single, large charcoal fragment	VERA-6155
	2014-3184	U5-SE hearth	71	3	450	*Juniperus* sp.	single, large charcoal fragment	VERA-6156
OH18	2012-1352	T6-SW	45	25	453	*Juniperus* sp.	large branch or trunk; 20 straight, wide growth rings	VERA-5854
OH19	2014-3348	U4-SW hearth	20	45	470	*Juniperus* sp.	single, large charcoal fragment	VERA-6157
	2014-3421	T3-NW hearth	45	55	484	*Juniperus* sp.	single, large charcoal fragment	VERA-6158
OH20	2012-1382	T5-NE	80	88	470	*Juniperus* sp.	15 straight, wide growth rings	VERA-5855
**Hiatus**								
IL4	2012-1481	T6-SW	35	15	503	*Juniperus* sp.	14 straight, wide growth rings	VERA-5856
**MOUSTERIAN**								
OH22	2013-384	T6-E	100	18	593	*Pinus nigra/sylvestris*	large, clean	VERA-5899
	2013-330	T6-SE	70	41	602	*Pinus nigra/sylvestris*	6 growth rings	VERA-5900
OH23	2013-258	T7-SE	85	3	606	*Juniperus* sp.	19 growth rings	VERA-5901
	2013-361	T6-SE	75	25	618	*Pinus nigra/sylvestris*	from sieve; xyz approximate	VERA-5902

(a) The previously published result for this sample (Lucena et al., 2012: Table 1) is affected by a typo that went uncorrected at the time of proof revision. The sample's correct age is 27260±230 BP, as given in Table 2, not 27620±230 BP, as printed in Lucena et al. (2012)

Table S4.2. ADB. Stratigraphic distribution of marine and fluviatile shell finds (a). Taxonomy follows WoRMS Editorial Board (2016). MT = Mousterian; EA = Evolved Aurignacian (Aurignacian II); LA = Late Aurignacian (Aurignacian III-IV); EG = Early Gravettian; MG = Middle Gravettian; P-S = Proto-Solutrean (?); LS = Lower Solutrean; MS = Middle Solutrean; US = Upper Solutrean; S-G = Solutreo-gravettian; EM = Early Magdalenian; UM = Upper Magdalenian; EpM = Epimagdalenian

**PERFORATED**	**MT**	**EA**	**LA**	**EG**	**MG**	**P-S**	**LS**	**MS**	**US**	**S-G**	**EM**	**UM**	**EpM**	**TOTAL**
**Gastropoda**														
Acteon tornatilis	–	–	–	–	–	–	–	–	4	1	–	–	–	5
Conus ventricosus	–	1	–	–	–	–	–	–	–	–	–	–	–	1
Littorina obtusata	–	–	–	–	1	–	3	2	2	–	–	1	–	9
Smaragdia viridis	–	–	–	–	1	–	2	–	–	–	–	–	–	3
Theodoxus fluviatilis	–	3	–	–	–	–	6	2	2	2	–	–	–	15
Tritia incrassata	–	1	–	–	–	–	–	–	–	–	–	–	–	1
Tritia mutabilis	–	–	–	–	–	–	–	1	–	–	–	–	–	1
Tritia neritea	–	–	–	–	–	–	16	–	6	1	–	–	–	23
Trivia sp.	–	1	–	–	–	–	–	–	–	–	–	–	–	1
**Bivalvia**														
Glycymeris insubrica (with umbo)	–	–	–	–	4	–	–	–	–	–	–	–	–	4
Striarca lactea	–	1	–	–	–	–	–	–	–	–	–	–	–	1
**Scaphopoda**														
Dentalium (Antalis) vulgare	–	–	–	–	2	–	5	3	4	–	–	–	–	14
**UNPERFORATED**	**MT**	**EA**	**LA**	**EG**	**MG**	**P-S**	**LS**	**MS**	**US**	**S-G**	**EM**	**UM**	**EpM**	**TOTAL**
**Gastropoda**														
Acteon tornatilis	–	–	–	–	–	–	–	–	2	–	–	–	–	2
Gibbula sp.	–	3	–	–	–	–	–	–	–	–	–	–	–	3
Littorina obtusata	–	–	1	–	–	–	1	2	–	–	–	–	–	4
Melanopsis sp.	–	–	–	–	–	–	–	–	–	–	–	14	–	14
Nucella lapillus	–	–	–	–	–	–	–	3	–	–	–	–	–	3
Theodoxus fluviatilis	–	8	–	–	1	1	11	1	8	3	1	–	–	34
Tritia neritea	–	–	–	–	–	–	2	–	4	–	–	–	–	6
Trivia sp.	–	–	1	–	–	–	–	–	1	–	–	1	–	3
Turritela sp.	–	–	–	–	–	–	–	–	1	–	–	–	–	1
unidentified fragment	–	1	–	–	1	–	1	1	–	1	–	–	–	5
**Bivalvia**														
Acanthocardia sp. fragment	–	–	–	–	4	–	7	1	10	–	1	–	–	23
Cerastoderma sp. fragment	–	–	–	–	6	–	1	1	4	–	–	–	–	12
Glycymeris insubrica (fragment without umbo)	–	4	–	–	2	–	–	–	1	–	–	–	–	7
Mimachlamys sp. fragment	–	6	–	–	–	–	–	–	–	–	–	–	–	6
Mytilus sp. fragment	–	2	–	–	4	–	–	–	–	–	–	–	–	6
Pecten sp. fragment	–	3	–	1	4	1	6	9	4	–	2	–	–	30
Striarca lactea	–	4	–	–	–	–	–	–	–	–	–	–	–	4
unidentified fragment	–	4	2	1	9	1	24	9	12	10	2	6	1	81
**ALL**	**MT**	**EA**	**LA**	**EG**	**MG**	**P-S**	**LS**	**MS**	**US**	**S-G**	**EM**	**UM**	**EpM**	**TOTAL**
TOTAL	–	42	4	2	39	3	85	35	65	18	6	22	1	322

(a) The rock-shelter's fill contains fossil shell derived from the calcarenite bedrock. *Pecten* sp. is the most common. When sieve-collected, such finds were either subsequently discarded or, if the diagnosis was uncertain, kept but not counted. For very small fragments, however, it cannot be excluded that the "bivalve fragment" and "*Pecten* sp. fragment" categories are somewhat inflated by the presence of such inherited material

Table S4.3. ADB. Standard typological classification of stone tools. Following the type-list of Sonneville-Bordes and Perrot (1954-56), with the modifications introduced by Zilhão (1997). MT = Mousterian; EA = Aurignacian II (Evolved Aurignacian); LA = Aurignacian III/IV (Late Aurignacian); EG = Early Gravettian; MG = Middle Gravettian; P-S = Proto-Solutrean (?); LS = Lower Solutrean; MS = Middle Solutrean; US = Upper Solutrean; S-G = Solutreo-gravettian; EM = Early Magdalenian

#	Type	MT	EA	LA	EG	MG	P-S	LS	MS	US	S-G	EM
**ENDSCRAPERS**												
**1a**	simple endscraper on blade	–	–	–	–	6	–	15	5	6	2	–
**1b**	simple endscraper on flake	–	–	–	–	–	–	1	1	1	–	–
**2a**	atypical endscraper on blade	–	–	–	–	2	1	1	1	–	–	–
**2b**	atypical endscraper on flake	–	–	1	–	–	–	–	–	2	–	–
**3**	double endscraper	–	–	–	–	5	–	3	1	1	–	–
**4**	ogival endscraper	–	–	–	–	–	–	1	–	–	2	1
**5a**	endscraper on retouched blade	–	–	–	–	4	–	–	3	2	2	–
**5b**	endscraper on retouched flake	–	–	–	–	–	–	18	1	5	1	–
**7**	fan endscraper	–	–	–	–	1	–	4	–	–	–	–
**8**	endscraper on flake	–	–	–	–	1	–	1	–	1	–	–
**10**	thumbnail endscraper	–	2	–	–	–	–	–	–	1	–	–
**11**	carinated endscraper	–	3	–	–	–	–	–	–	–	–	–
**12**	atypical carinated scraper	–	2	–	–	–	–	–	–	–	–	–
**13**	nosed endscraper	–	2	–	–	–	–	–	–	–	–	–
**14a**	flat-nosed endscraper	–	–	–	–	1	–	–	–	–	–	–
**14b**	flat-shouldered endscraper	–	–	–	–	–	–	1	–	–	–	–
**COMPOSITE TOOLS**												
**17**	endscraper-burin	–	–	–	–	1	–	2	–	1	1	–
**18**	endscraper-truncation	–	–	–	–	–	–	1	–	–	–	–
**PERFORATORS**												
**23**	perforator	–	–	2	–	–	–	–	–	–	–	–
**24**	atypical perforator	–	–	–	–	–	–	1	–	1	–	–
**BURINS**												
**27**	straight dihedral burin	–	–	–	–	–	–	–	–	–	–	1
**28**	dihedral déjeté burin	–	–	–	1	3	–	1	–	1	–	–
**29**	dihedral burin on angle	–	–	–	–	–	–	–	–	1	–	–
**30a**	angle burin on natural surface or break	–	2	–	–	1	–	2	2	–	–	–
**34**	burin on straight truncation	–	–	–	–	1	–	–	–	–	–	–
**35**	burin on oblique truncation	–	1	–	–	2	–	2	–	–	–	–
**36**	burin on concave truncation	–	–	1	–	5	–	1	–	–	–	–
**38**	transversal burin on truncation	–	–	–	–	–	–	–	–	–	1	–
**40**	multiple burin on truncation	–	–	–	–	3	–	1	–	–	–	–
**41**	multiple-mixed burin	–	–	–	–	2	–	1	2	–	–	–
**42a**	Noailles burin	–	–	–	–	1	–	–	–	1	–	–
**42b**	Vale Comprido burin	–	–	–	–	–	–	–	–	1	–	–
**BACKED TOOLS**												
**51d**	basal microgravette	–	–	–	1	–	–	–	–	–	1	–
**TRUNCATIONS**												
**61**	oblique truncation on blade	–	–	–	–	–	–	1	–	–	–	–
**62**	concave truncation	–	–	–	–	–	–	1	–	–	–	–
**RETOUCHED BLADES**												
**65**	continuous-retouch blade, unilateral	–	–	–	–	1	–	1	–	1	–	–
**66**	continuous-retouch blade, bilateral	–	1	–	–	1	1	–	1	1	–	–
**SOLUTREAN TOOLS**												
**69a**	unifacial point	–	–	–	–	–	–	24	2	–	1	–
**70n**	laurel-leaf fragment	–	–	–	–	–	–	–	1	–	–	–
**71**	willow-leaf fragment	–	–	–	–	–	–	–	–	1	–	–
**72a**	shouldered point	–	–	–	–	–	–	–	–	5	–	–

Table S4.3. ADB. Standard typological classification of stone tools (cont.)

#	Type	MT	EA	LA	EG	MG	P-S	LS	MS	US	S-G	EM
**SUBSTRATE**												
**74**	notched piece	1	7	7	–	2	–	1	–	3	1	–
**75a**	denticulate	4	–	–	–	–	–	–	–	–	–	–
**75b**	double denticulate	1	–	–	–	–	–	–	–	–	–	–
**76**	splintered piece	–	19	11	–	4	–	17	5	4	2	1
**77a**	sidescraper	2	–	–	–	–	–	–	–	–	–	–
**77b**	transversal sidescraper	1	–	–	–	–	–	–	–	–	–	–
**77c**	convergent sidescraper	1	–	–	–	–	–	–	–	–	–	–
**77d**	denticulated sidescraper	1	–	–	–	–	–	–	–	–	–	–
**77f**	sidescraper fragment	1	–	–	–	–	–	–	–	–	–	–
**78b**	Vascas scraper	–	–	–	–	–	–	–	–	2	–	–
**BLADELET TOOLS**												
**81**	trapeze	–	–	–	–	–	–	–	–	2	–	–
**83**	segment	–	–	–	–	–	–	–	–	1	–	–
**84**	truncated bladelet	–	–	–	–	1	–	–	–	–	–	–
**85a**	backed bladelet	–	–	2	–	–	–	–	–	1	–	3
**85c**	partially backed bladelet	–	–	–	–	–	–	1	–	–	–	1
**85d**	double-back bladelet	–	–	–	–	1	–	–	–	–	–	–
**85f**	backed bladelet fragment	–	–	5	–	2	–	2	–	3	7	2
**86a**	backed-truncated bladelet	–	–	–	–	2	–	–	–	1	–	–
**86b**	backed, double-truncated bladelet	–	–	1	–	–	–	–	–	–	–	–
**87a**	denticulated backed bladelet	–	–	–	–	–	–	–	–	–	1	2
**89**	notched bladelet	–	3	1	–	–	–	1	1	5	–	–
**90a**	Dufour bladelet	–	3	5	–	1	1	1	–	–	–	–
**90b**	Areeiro bladelet	–	1	2	–	–	–	–	–	–	–	–
**90c**	marginally backed bladelet	–	–	5	–	1	–	1	1	5	5	3
**VARIA**												
**92a**	atypically retouched piece	5	4	5	–	3	1	3	1	9	2	–
**92b**	retouched piece fragment	3	8	4	1	6	1	26	12	25	6	–
**92d**	pointed bladelet	–	–	–	–	–	–	1	–	1	2	1
**92e**	hammerstone	–	1	–	–	–	–	–	–	–	–	–
	**TOTAL**	**20**	**59**	**52**	**3**	**64**	**6**	**138**	**40**	**95**	**37**	**15**

Table S4.4. ADB bioclastic flint analysis. Nodule types defined in horizons OH18-to-IL4

TYPE	DESCRIPTION	OBSERVATIONS
**A**	yellowish brown, opaque, very thin cortex	–
**B**	light brown, semi-opaque, very thin iron-impregnated cortex, possibly rolled	–
**C**	light brown, semi-opaque, very thin iron-impregnated cortex, possibly rolled; subcortical white weathering	–
**D**	brown, dark-spotted, semi-opaque, patinated	–
**E**	light brown, semi-opaque, patinated	–
**F**	fine-grained, brown, opaque, with rolled, whitish cortex	–
**G**	greyish brown, opaque, thin, rough-textured, iron-impregnated cortex	refits
**H**	brown, semi-opaque, heavily iron-spotted, with very thin, coarse-textured beige cortex	–
**I**	greyish brown, semi-opaque, thin, rough-textured cortex without iron impregnation	refits
**J**	reddish brown, semi-opaque, heavily iron-spotted with thick limestone-like cortex	refits
**K**	reddish brown, semi-translucent (cortex absent)	–
**L**	beige, semi-translucent, with limestone-like cortex	refits; more than one volume
**M**	yellowish-brown, semi-translucent (cortex absent)	likely a single volume
**N**	light reddish-brown, semi-opaque, iron-spotted with thin limestone-like, weathered cortex	refits; likely a single volume
**O**	yellowish brown, opaque, white thin iron-impregnated cortex with subcortical whitish weathering	–
**Z**	undetermined-to-nodule bioclastic flint of the same type and source	more than one volume

Table S4.5. ADB bioclastic flint analysis. Vertical distribution of each nodule type in units OH18-to-IL4 (number of items per archeo-stratigraphic unit)

Nodule	OH18	OH18/19	OH19	OH19/20	OH20	OH20/IL4	IL4	TOTAL
A	–	–	–	–	1	–	1	2
B	–	–	–	–	1	–	–	1
C	–	–	–	1	3	–	1	5
D	–	–	–	–	1	–	–	1
E	–	–	–	–	1	–	–	1
F	–	–	–	–	1	–	–	1
G	–	–	–	–	2	1	1	4
H	–	–	–	1	28	1	10	40
I	–	–	–	–	3	–	–	3
J	1	–	1	3	47	2	17	71
K	–	–	–	–	–	–	1	1
L	–	1	1	1	18	–	5	26
M	–	–	–	–	6	–	4	10
N	1	–	–	–	2	–	–	3
O	–	–	–	2	3	–	2	7
Z	2	2	1	–	17	–	12	34
TOTAL	4	3	3	8	134	4	54	210
Frequency	1.9%	1.4%	1.4%	3.8%	63.8%	1.9%	25.7%	100.0%

Table S4.6. ADB bioclastic flint analysis. Vertical distribution of each nodule type in units OH18-to-IL4 (total mass of items, in grams, per archeo-stratigraphic unit)

Nodule	OH18	OH18/19	OH19	OH19/20	OH20	OH20/IL4	IL4	TOTAL
A	–	–	–	–	136.6	–	1.9	138.5
B	–	–	–	–	14.2	–	–	14.2
C	–	–	–	0.1	24.2	–	0.1	24.4
D	–	–	–	–	0.9	–	–	0.9
E	–	–	–	–	4.5	–	–	4.5
F	–	–	–	–	7.5	–	–	7.5
G	–	–	–	–	28.5	6.7	3.1	38.2
H	–	–	–	0.2	49.1	0.2	15.2	64.7
I	–	–	–	–	145.3	–	–	145.3
J	0.5	–	0.5	9.0	235.4	0.6	77.8	323.8
K	–	–	–	–	–	–	0.5	0.5
L	–	4.4	1.5	0.5	37.1	–	7.7	51.2
M	–	–	–	–	2.7	–	2.4	5.1
N	14.2	–	–	–	16.5	–	–	30.7
O	–	–	–	1.2	15.2	–	8.6	25.0
Z	1.0	0.7	0.2	–	7.2	–	8.8	17.8
TOTAL	15.7	5.0	2.2	11.1	724.9	7.4	126.0	892.4
Frequency	1.8%	0.6%	0.2%	1.2%	81.2%	0.8%	14.1%	100.0%

Table S4.7. ADB Mousterian. Stone tool technological categories. N = number, M = mass in grams. OH21, OH22 and OH23 combined

RAW-MATERIAL	CORES		FLAKE BLANKS						LAMINARY BLANKS				DEBRIS				TOOLS		TOTAL	
			Complete		Fragment		Small		Blade		Bladelet		Chippage		Chunk					
	N	M	N	M	N	M	N	M	N	M	N	M	N	M	N	M	N	M	N	M
**Flint**	2	37.8	27	144,71	32	62.8	35	32.4	–	–	1	0.3	153	19,76	1	10.3	19	113,89	270	422.0
**Quartzite**	–	–	1	21,27	–	–	1	0.8	–	–	–	–	3	0,73	–	–	1	13,62	6	36.5
**Limestone**	–	–	–	–	2	6.7	1	0.4	–	–	–	–	4	1,19	–	–	–	–	7	8.3
**Quartz**	–	–	–	–	–	–	–	–	–	–	–	–	–	–	–	–	–	–	–	–
**TOTAL**	2	37.8	28	166.0	34	69.53	37	33.6	–	–	1	0.3	160	21.7	1	10.3	20	127.5	283	466.7

Table S4.8. ADB Mousterian. Classification of cores and retouched tools. OH21, OH22 and OH23 combined

Cores	N
Kombewa	1
discoid? (fragment)	1
**TOTAL**	**2**

Retouched tools	N
notched piece	1
denticulate	5
sidescraper	
unilateral	2
transversal	1
convergent	1
denticulated	1
fragment	1
atypically retouched flake	5
retouched piece fragment	3
**TOTAL**	**24**

Table S4.9. ADB Mousterian and Aurignacian stone tools (flint). Use-wear evidence

	Illegible	None	Wood	Hide	Meat	Bone	Projectile	Ochred	Total
**MOUSTERIAN**									
sidescrapers	2	3	1	–	–	–	–	–	**6**
denticulates	3	–	1	–	–	–	–	–	**4**
notches	–	–	1	–	–	–	–	–	**1**
edge-retouched pieces and fragments	1	1	1	1	1 (a)	–	–	–	**5**
unretouched flake	–	19	5	–	–	–	–	–	**24**
**TOTAL**	**6**	**23**	**9**	**1**	**1**	**–**	**–**	**–**	**40**
**AURIGNACIAN**									
cores (b)	1	5	–	–	–	–	–	–	**6**
endscrapers	1	5	–	–	–	–	–	–	**6**
perforators	1	–	1	–	–	–	–	–	**2**
notches	2	10	1	1	–	–	–	–	**14**
bladelet tools	4	22	–	–	–	–	3	–	**29**
retouched pieces and/or fragments	6	8	2	1	–	1	–	–	**18**
unretouched blanks	1	2	–	–	–	–	–	–	**3**
**TOTAL**	**16**	**52**	**4**	**2**	**–**	**1**	**3**	**–**	**78**

(a) flesh-removal or skinning, but uncertain; (b) includes carinated/nosed scrapers," "burins," and splintered pieces

Table S4.10. ADB Aurignacian (OH20). Stone tool technological categories. The items retrieved in IL4 are included. N = number, M = mass in grams

RAW-MATERIAL	CORES		FLAKE BLANKS						LAMINARY BLANKS				DEBRIS				TOOLS		TOTAL	
			Complete		Fragment		Small		Blade		Bladelet		Chippage		Chunk					
	N	M	N	M	N	M	N	M	N	M	N	M	N	M	N	M	N	M	N	M
**Flint**	7	510.5	25	190.4	56	161.0	27	23.5	18	91.2	37	11.4	262	43.5	10	19.0	8	24.1	450	1074.6
**Quartzite**	–	–	–	–	–	–	–	–	–	–	–	–	–	–	–	–	–	–	–	–
**Limestone**	–	–	–	–	1	1.0	–	–	–	–	–	–	2	0.1	–	–	–	–	3	1.1
**Quartz**	–	–	–	–	–	–	–	–	–	–	–	–	–	–	–	–	–	–	–	–
**TOTAL**	7	510.5	25	190.4	57	162.0	27	23.5	18	91.2	37	11.4	264	43.6	10	19.0	8	24.1	453	1075.8

Table S4.11. ADB Aurignacian (OH20). Classification of cores, retouched tools and bladelets. Bladelet counts include both retouched and unretouched blanks. The items retrieved in IL4 are included

Cores	N
prismatic for blades	4
carinated "scraper"	2
Kostenki truncation	1
**TOTAL**	7

Bladelets extracted from	N
carinated/nosed "scraper"	10
other	31
**TOTAL**	41

Retouched tools	N
thick, atypical thumbnail endscraper	1
notched bladelet	1
Dufour bladelet	1
Areeiro bladelet	1
atypically retouched piece	2
retouched piece fragment	2
**TOTAL**	**8**

Table S4.12. ADB Aurignacian (OH19). Stone tool technological categories. N = number, M = mass in grams

			FLAKE BLANKS						LAMINARY BLANKS				DEBRIS							
	CORES		Complete		Fragment		Small		Blade		Bladelet		Chippage		Chunk		TOOLS		TOTAL	
RAW-MATERIAL	N	M	N	M	N	M	N	M	N	M	N	M	N	M	N	M	N	M	N	M
**Flint**	6	36.0	4	8.0	23	23.5	12	11.0	–	–	6	1.9	83	14.2	4	5.2	7	17.0	145	116.8
**Quartzite**	–	–	–	–	1	0.9	–	–	–	–	–	–	–	–	–	–	–	–	1	0.9
**Limestone**	–	–	–	–	–	–	–	–	–	–	–	–	–	–	–	–	–	–	–	–
**Quartz**	–	–	–	–	–	–	–	–	–	–	–	–	–	–	–	–	–	–	–	–
**TOTAL**	6	36.0	4	8.0	24	24.4	12	11.0	–	–	6	1.9	83	14.2	4	5.2	7	17.0	146	117.7

Table S4.13. ADB Aurignacian (OH19). Classification of cores, retouched tools and bladelets. Bladelet counts include both retouched and unretouched blanks

Cores	N
prismatic for bladelets (fragment)	2
carinated "scraper"	1
nosed "scraper"	1
splintered piece/bipolar core	2
**TOTAL**	6

Bladelets extracted from	N
carinated/nosed "scraper"	3
other	5
**TOTAL**	8

Retouched tools	N
bilaterally retouched blade	1
notched piece	2
notched bladelet	2
retouched piece fragment	2
**TOTAL**	**7**

Table S4.14. ADB Aurignacian (OH18). Stone tool technological categories. N = number, M = mass in grams

			FLAKE BLANKS						LAMINARY BLANKS				DEBRIS							
	CORES		Complete		Fragment		Small		Blade		Bladelet		Chippage		Chunk		TOOLS		TOTAL	
RAW-MATERIAL	N	M	N	M	N	M	N	M	N	M	N	M	N	M	N	M	N	M	N	M
**Flint**	6	21.8	7	43.8	23	29.7	18	20.3	–	–	9	1.7	127	28.8	5	15.0	4	9.6	199	170.7
**Quartzite**	–	–	–	–	2	3.3	–	–	–	–	–	–	–	–	–	–	–	–	2	3.3
**Limestone**	–	–	–	–	–	–	–	–	–	–	–	–	1	0.3	–	–	–	–	1	0.3
**Quartz**	–	–	–	–	–	–	–	–	–	–	–	–	–	–	–	–	–	–	–	–
**TOTAL**	6	21.8	7	43.8	25	33.0	18	20.3	–	–	9	1.7	128	29.2	5	15.0	4	9.6	202	174.3

Table S4.15. ADB Aurignacian (OH18). Classification of cores, retouched tools and bladelets. Bladelet counts include both retouched and unretouched blanks

Cores	N
prismatic for bladelets (fragment)	3
carinated "scraper"	1
"burin"	1
splintered piece/bipolar core	1
**TOTAL**	6

Bladelets extracted from	N
carinated/nosed "scraper"	2
"burin"	1
splintered piece/bipolar core	1
other	6
**TOTAL**	10

Burin types	N
angle on natural surface	1
**TOTAL**	**1**

Retouched tools	N
thumbnail scraper	1
Dufour bladelet	1
atypically retouched piece	1
retouched piece fragment	1
**TOTAL**	**4**

Table S4.16. ADB Aurignacian (OH17). Stone tool technological categories. N = number, M = mass in grams; an ochre-stained quartzite cobble is not counted

RAW-MATERIAL	CORES		FLAKE BLANKS						LAMINARY BLANKS				DEBRIS				TOOLS		TOTAL	
			Complete		Fragment		Small		Blade		Bladelet		Chippage		Chunk					
	N	M	N	M	N	M	N	M	N	M	N	M	N	M	N	M	N	M	N	M
**Flint**	32	159.9	21	91.0	72	130.0	83	100.8	2	14.5	42	23.5	587	132.2	40	157.6	9	20.76	888	830.4
**Quartzite**	–	–	–	–	2	5.8	–	–	–	–	–	–	1	0.3	–	–	–	–	3	6.1
**Limestone**	–	–	8	184.9	9	35.1	2	2.3	–	–	–	–	2	0.8	5	14.0	1	22.45	27	259.5
**Quartz**	–	–	–	–	–	–	2	2.3	–	–	–	–	3	0.6	–	–	–	–	5	3.0
**TOTAL**	32	159.9	29	275.9	83	170.9	87	105.6	2	14.5	42	23.5	593	133.9	45	171.6	10	43.2	923	1099.0

Table S4.17. ADB Aurignacian (OH17). Classification of cores, retouched tools and bladelets. Bladelet counts include both retouched and unretouched blanks

**Cores**	**N**
prismatic for bladelets	4
prismatic, other	2
polyhedral	1
fragments and other	5
carinated “scraper”	1
nosed “scraper”	1
“burin”	2
splintered piece/bipolar core	16
**TOTAL**	32

**Bladelets extracted from**	**N**
carinated/nosed “scraper”	3
“burin”	6
splintered piece/bipolar core	19
other	15
**TOTAL**	43

**Burin types**	**N**
angle, on natural surface	1
on oblique truncation	1
**TOTAL**	**2**

**Retouched tools**	**N**
notched piece	5
Dufour bladelet	1
atypically retouched piece	1
retouched piece fragment	3
**TOTAL**	**10**

Table S4.18. ADB Aurignacian (OH16). Stone tool technological categories. N = number, M = mass in grams

RAW-MATERIAL	CORES		FLAKE BLANKS						LAMINARY BLANKS				DEBRIS				TOOLS		TOTAL	
			Complete		Fragment		Small		Blade		Bladelet		Chippage		Chunk					
	N	M	N	M	N	M	N	M	N	M	N	M	N	M	N	M	N	M	N	M
**Flint**	22	166.6	32	227.3	117	292.4	82	114.5	7	10.6	51	17.0	1069	224.2	44	176.6	28	80.3	1452	1309.7
**Quartzite**	–	–	–	–	1	17.3	–	–	–	–	–	–	–	–	–	–	–	–	1	17.3
**Limestone**	2	327.3	4	80.8	20	141.8	4	3.6	–	–	–	–	50	20.2	9	33.3	1	8.1	90	615.1
**Quartz**	–	–	–	–	–	–	–	–	–	–	–	–	3	0.6	–	–	–	–	–	–
**TOTAL**	24	493.8	36	308.1	138	451.6	86	118.1	7	10.6	51	17.0	1119	244.4	53	210.0	29	88.4	1543	1942.0

Table S4.19. ADB Aurignacian (OH16). Classification of cores, retouched tools and bladelets. Bladelet counts include both retouched and unretouched blanks

**Cores**	**N**
prismatic for blades	2
prismatic for bladelets	7
prismatic for flakes	2
fragments and other	2
splintered piece/bipolar core	11
**TOTAL**	24

**Bladelets extracted from**	**N**
carinated/nosed “scraper”	8
splintered piece/bipolar core	9
other	48
**TOTAL**	65

**Retouched tools**	**N**
simple endscraper, atypical	1
perforator	2
notched piece	6
short-backed bladelet	5
notched bladelet	1
Dufour bladelet	2
Areeiro bladelet	2
marginally retouched bladelet	4
atypically retouched piece	2
retouched piece fragment	4
**TOTAL**	**29**

Table S4.20. ADB Aurignacian (OH15). Stone tool technological categories. N = number, M = mass in grams

RAW-MATERIAL	CORES		FLAKE BLANKS						LAMINARY BLANKS				DEBRIS				TOOLS (a)		TOTAL	
			Complete		Fragment		Small		Blade		Bladelet		Chippage		Chunk					
	N	M	N	M	N	M	N	M	N	M	N	M	N	M	N	M	N	M	N	M
**Flint**	2	29.7	9	95.1	17	31.2	11	8.4	8	9.1	18	9.6	151	39.4	3	10.4	12	13.8	231	246.7
**Quartzite**	–	–	–	–	–	–	–	–	–	–	–	–	–	–	–	–	–	–	–	–
**Limestone**	–	–	–	–	–	–	–	–	–	–	–	–	–	–	–	–	–	–	–	–
**Quartz**	–	–	–	–	–	–	–	–	–	–	–	–	–	–	–	–	–	–	–	–
**TOTAL**	2	29.7	9	95.1	17	31.2	11	8.4	8	9.1	18	9.6	151	39.4	3	10.4	12	13.8	231	246.7

(a) two refitted fragments of a large, marginally backed bladelet, typologically listed as a single item, are here counted separately

Table S4.21. ADB Aurignacian (OH15). Classification of cores, retouched tools and bladelets. Bladelet counts include both retouched and unretouched blanks

Cores	N
prismatic for flakes	1
"burin"	1
**TOTAL**	2

Bladelets extracted from	N
"burin"	4
other	22
**TOTAL**	26

Burin types	N
on concave truncation	1
**TOTAL**	**1**

Retouched tools	N
notched piece	1
backed bladelet fragment	1
short-backed bladelet	1
backed-truncated bladelet	1
Dufour bladelet	3
marginally retouched bladelet	1
atypically retouched piece	3
**TOTAL**	**11**

Table S4.22. ADB Gravettian (OH14). Stone tool technological categories. N = number, M = mass in grams

RAW-MATERIAL	CORES		FLAKE BLANKS						LAMINARY BLANKS				DEBRIS				TOOLS (a)		TOTAL	
			Complete		Fragment		Small		Blade		Bladelet		Chippage		Chunk					
	N	M	N	M	N	M	N	M	N	M	N	M	N	M	N	M	N	M	N	M
**Flint**	2	13.6	4	46.7	6	7.6	3	2.0	–	–	6	2.5	58	16.4	2	6.4	1	0.6	82	95.8
**Quartzite**	–	–	–	–	–	–	–	–	–	–	–	–	–	–	–	–	–	–	–	–
**Limestone**	–	–	–	–	–	–	–	–	–	–	–	–	–	–	–	–	–	–	–	–
**Quartz**	–	–	–	–	–	–	–	–	–	–	–	–	–	–	–	–	–	–	–	–
**TOTAL**	2	13.6	4	46.7	6	7.6	3	2.0	–	–	6	2.5	58	16.4	2	6.4	1	0.6	82	95.8

Table S4.23. ADB Gravettian (OH14). Classification of cores, retouched tools and bladelets. Bladelet counts include both retouched and unretouched blanks

Cores	N
prismatic for bladelets	2
**TOTAL**	2

Bladelets extracted from	N
"burin"	4
other	3
**TOTAL**	7

Retouched tools	N
basal microgravette	1
**TOTAL**	**1**

Table S4.24. ADB Gravettian (OH13). Stone tool technological categories. IL3 items included. N = number, M = mass in grams

RAW-MATERIAL	CORES		FLAKE BLANKS						LAMINARY BLANKS				DEBRIS				TOOLS (a)		TOTAL	
			Complete		Fragment		Small		Blade		Bladelet		Chippage		Chunk					
	N	M	N	M	N	M	N	M	N	M	N	M	N	M	N	M	N	M	N	M
**Flint**	1	4.9	2	11.8	2	1.1	6	9.1	–	–	3	0.7	20	4.0	1	1.1	–	–	35	32.8
**Quartzite**	–	–	–	–	–	–	–	–	–	–	–	–	–	–	–	–	–	–	–	–
**Limestone**	–	–	–	–	–	–	–	–	–	–	–	–	–	–	–	–	–	–	–	–
**Quartz**	–	–	–	–	–	–	–	–	–	–	–	–	–	–	–	–	–	–	–	–
**TOTAL**	1	4.9	2	11.8	2	1.1	6	9.1	–	–	3	0.7	20	4.0	1	1.1	–	–	35	32.8

Table S4.25. ADB Gravettian (OH13). Classification of cores and bladelets. Bladelet counts include both retouched and unretouched blanks. IL3 items included

Cores	N
"burin"	1
**TOTAL**	1

Bladelets extracted from	N
"burin"	2
other	1
**TOTAL**	3

Burin types	N
dihedral-déjété	1
**TOTAL**	**1**

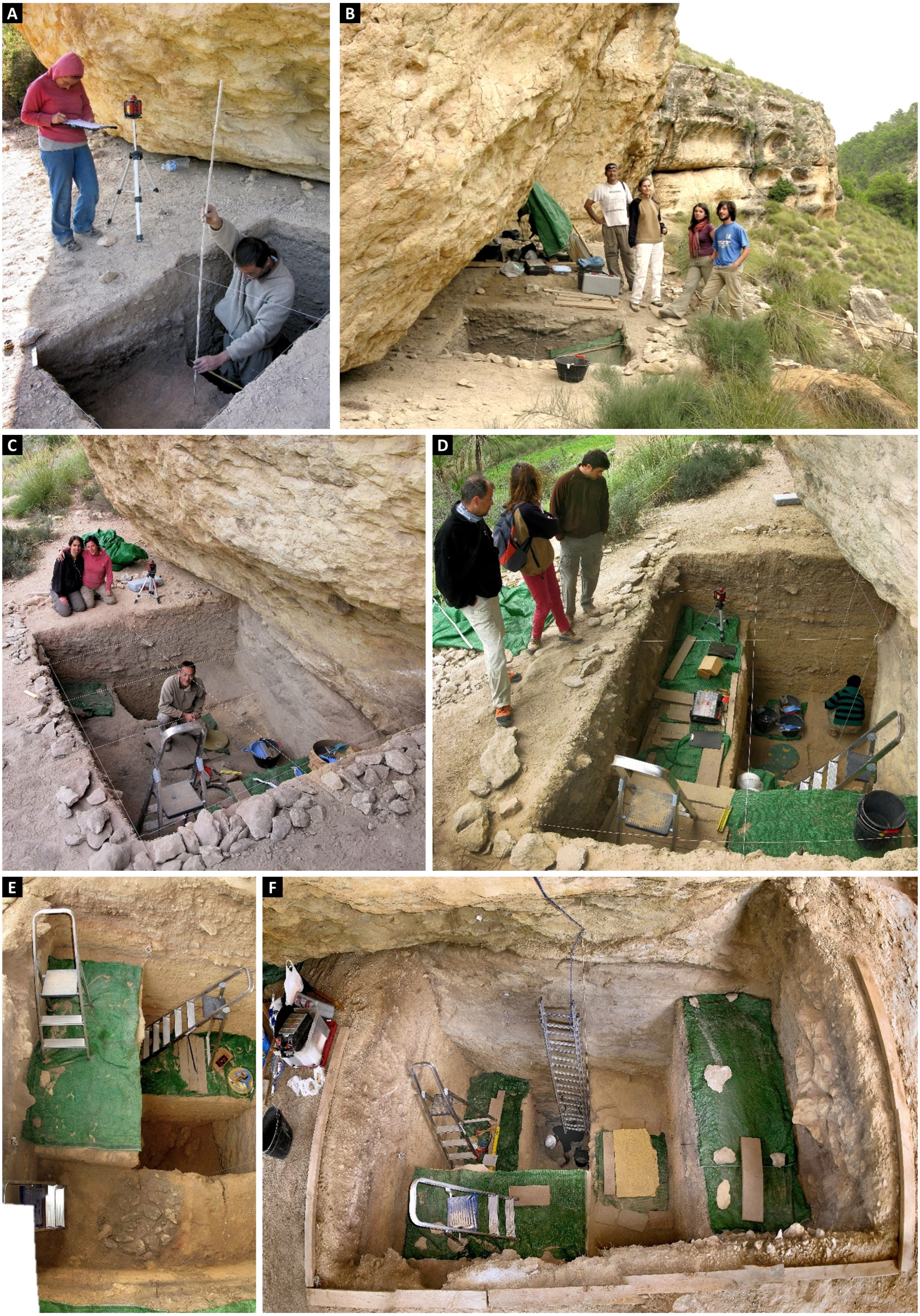

Fig. S4.1. La Boja. The excavation's progress. A. Initial testing (April 5, 2008). **B.** At the end of the 2008 field season (September 27). **C.** At the end of the 2009 field season (April 17). **D.** At the end of the 2010 field season (April 17). **E.** At the end of the 2012 field season (April 5). F. At the end of the Spring 2013 field season (April 11).

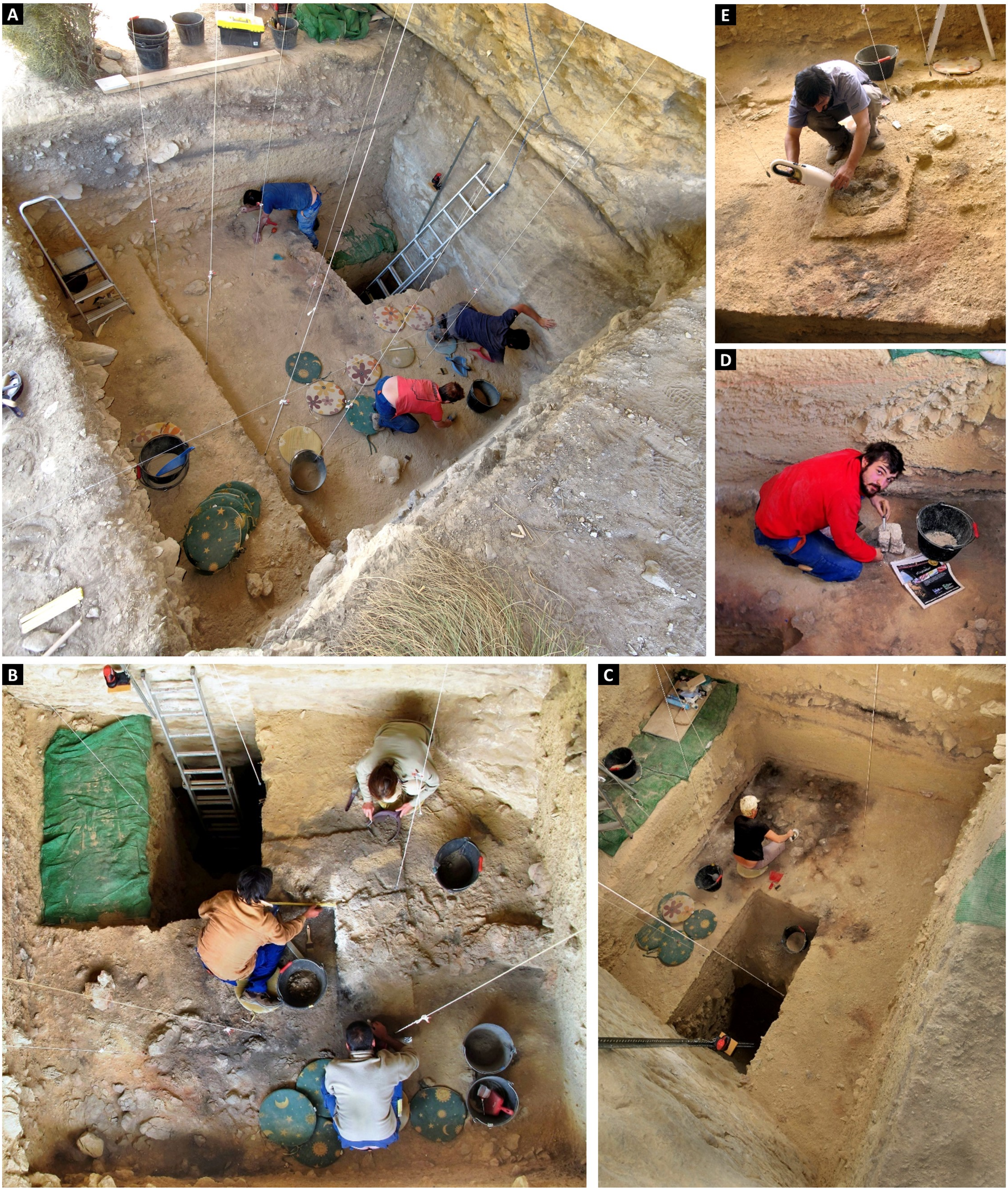

Fig. S4.2. La Boja. The 2014 field season. A. Preparing the A15 décapage (OH7) for recording (April 23). **B.** Excavating the A22 spit (OH10) (May 15). **C.** Preparing the A42 décapage (OH16) for recording (October 11). **D.** Cutting a sample for soil micromorphological analysis (from a sediment column in the middle of grid unit U4 representing the stacked-up hearth features of OH16 and the upper part of OH17) (October 14). **E.** Preparing the hearth found in spit A12 (OH5) of grid units T/3-4 for recording (April 17).

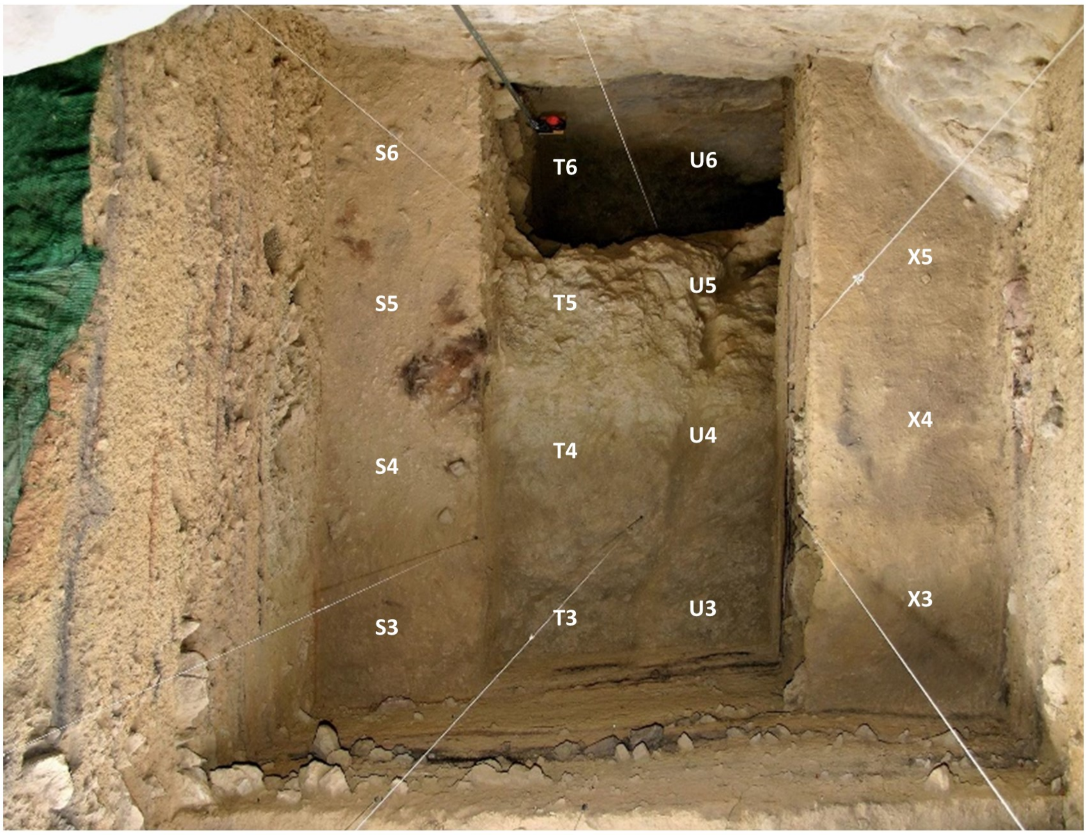

Fig. S4.3. La Boja. The 2014 excavations. Zenithal view of the situation of grid units S-X/3-6 of the excavation trench at the end of the field season (November 22). The surface of the large collapsed boulder that separates the Aurignacian from the Mousterian deposit is visible in T-U/3-5. Originally, it extended into row 6 but, in 2013, it was sectioned along the T-U6>5 cross-section so that the excavation could proceed down to bedrock.

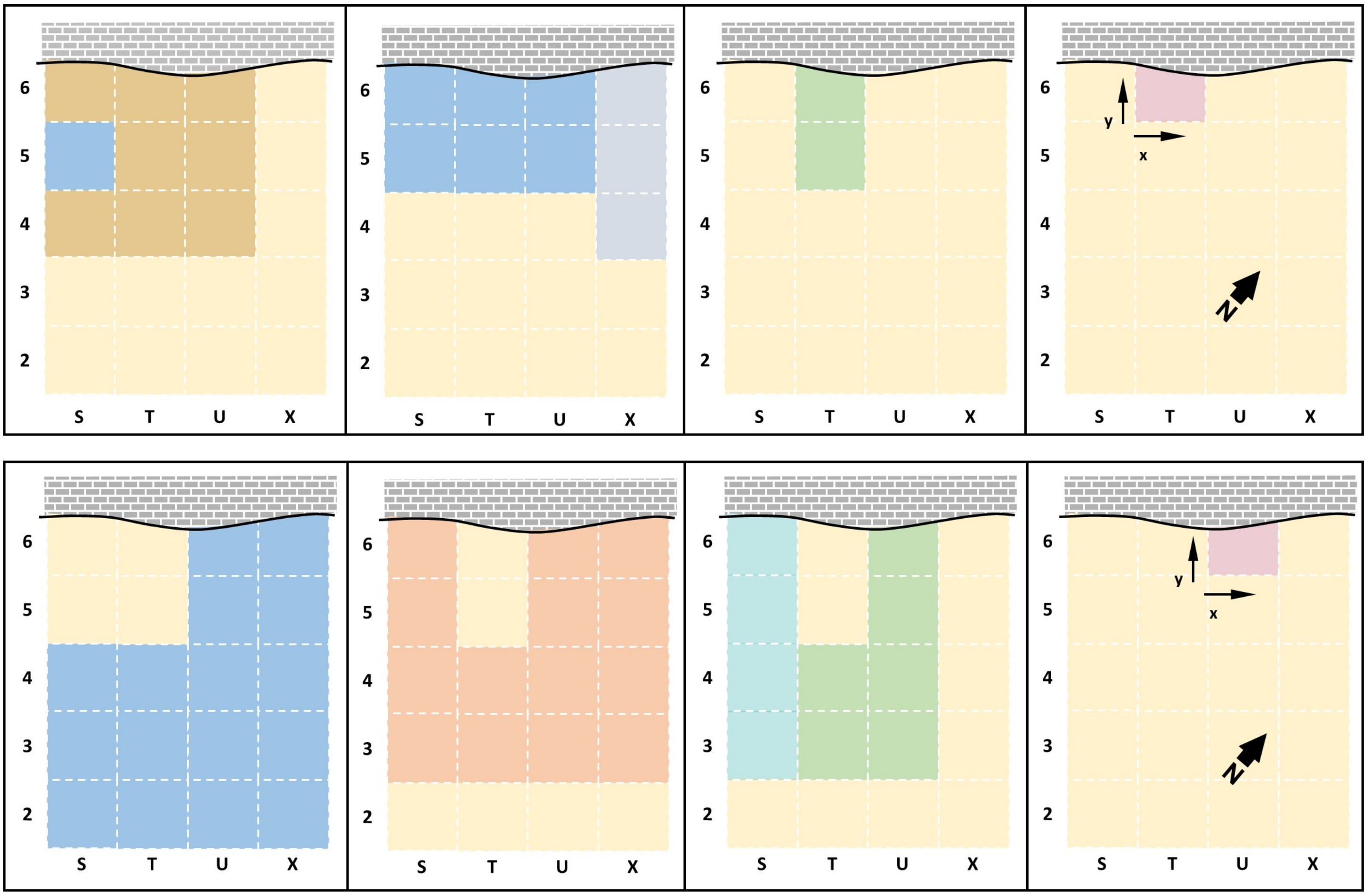

Fig. S4.4. La Boja. Top row: grid units excavated in the initial testing phase. Far left. 2008, down to the Upper Magdalenian in S-U/4-6, and to the Solutrean in S5. **Middle left.** 2009-2010, down to the base of the Holocene deposit in X/4-6, and down to the Solutrean or the Solutreo-gravettian in S-U/5-6. **Middle right.** 2012, down to the Aurignacian in T/5-6. **Far right.** April 2013, down to bedrock in T6. **Bottom row: grid units area-excavated in the August-September 2013 and March-November 2014 field seasons. Far left.** Down to the top of the Solutreo-gravettian. **Middle left.** Down to the base of the Middle Gravettian. **Middle right.** Down to the middle of the Aurignacian in S/3-6, and to its base in T/3-4 and U/3-6. **Far right.** Down to the Mousterian and bedrock.

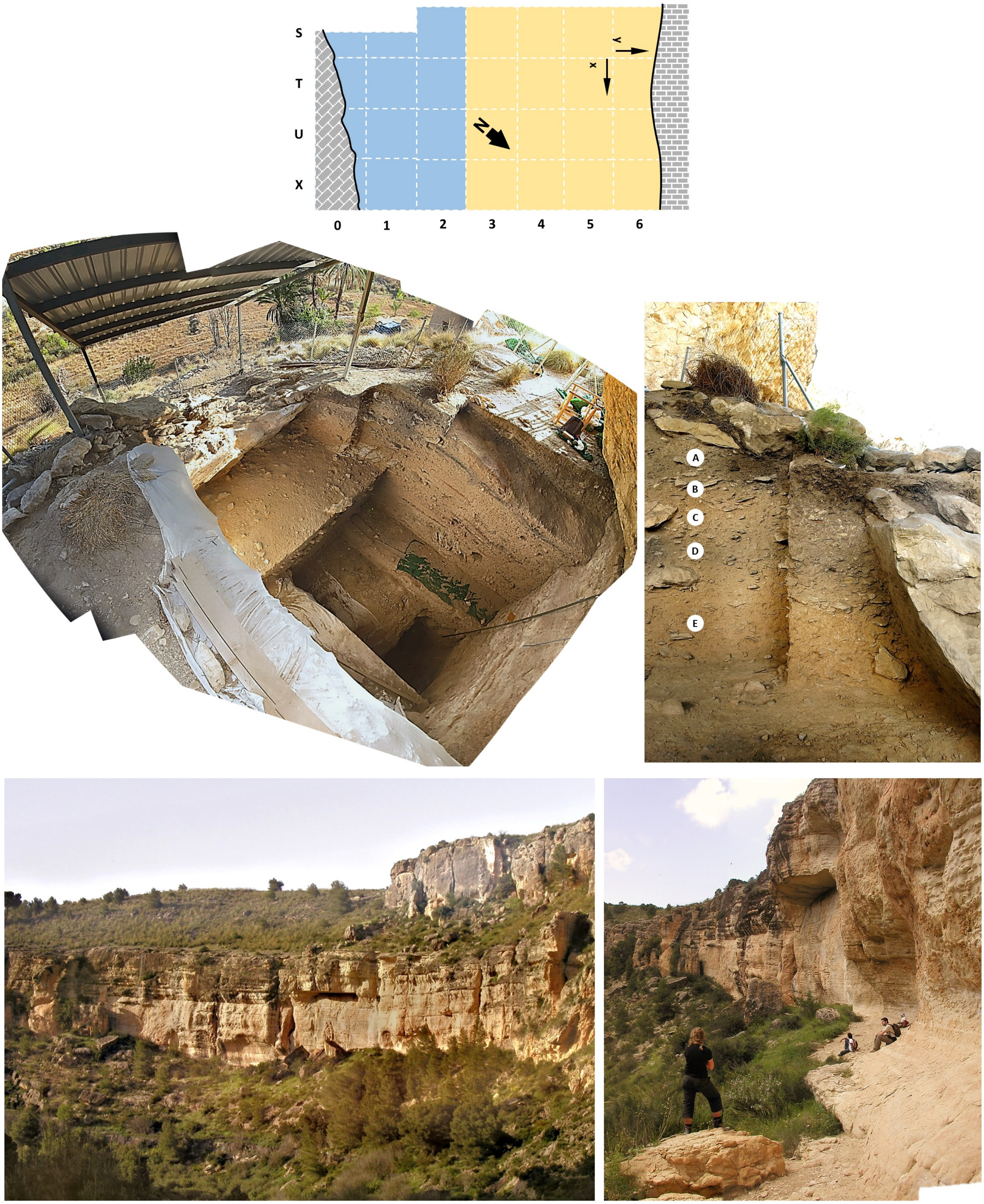

Fig. S4.5. La Boja. The 2016 field season. Top. The extended site grid, with indication of the units excavated down to the Middle Solutrean in 2016 (S-X/0-2). **Middle left.** Overview at the end of the season; note the cave-like environment, whereby sediment accumulates between the back wall and the steeply inward-tilted, largely flat, lower surface of the massive slab making for the site's outward boundary. **Middle right.** Cross-section in rows 0 and 1 during the excavation of OH12 in the SE corner of the trench; note the deep penetration along the outer wall of a lens of the dark, Upper Magdalenian layer D ("wall effect"). **Bottom.** The archeologically fertile but partially emptied rock-shelter of Corral de las Avispas, ca.500 m upstream from La Boja in a panoramic overview from SW (**left**) and an on-site view to North (**right**) (April 24, 2007); under the parsimonious hypothesis that La Boja's outer wall is a collapsed section of the overhang, a present-day analogue for the site's original configuration is provided by the Corral de las Avispas and its extant, flat-bottomed roof.

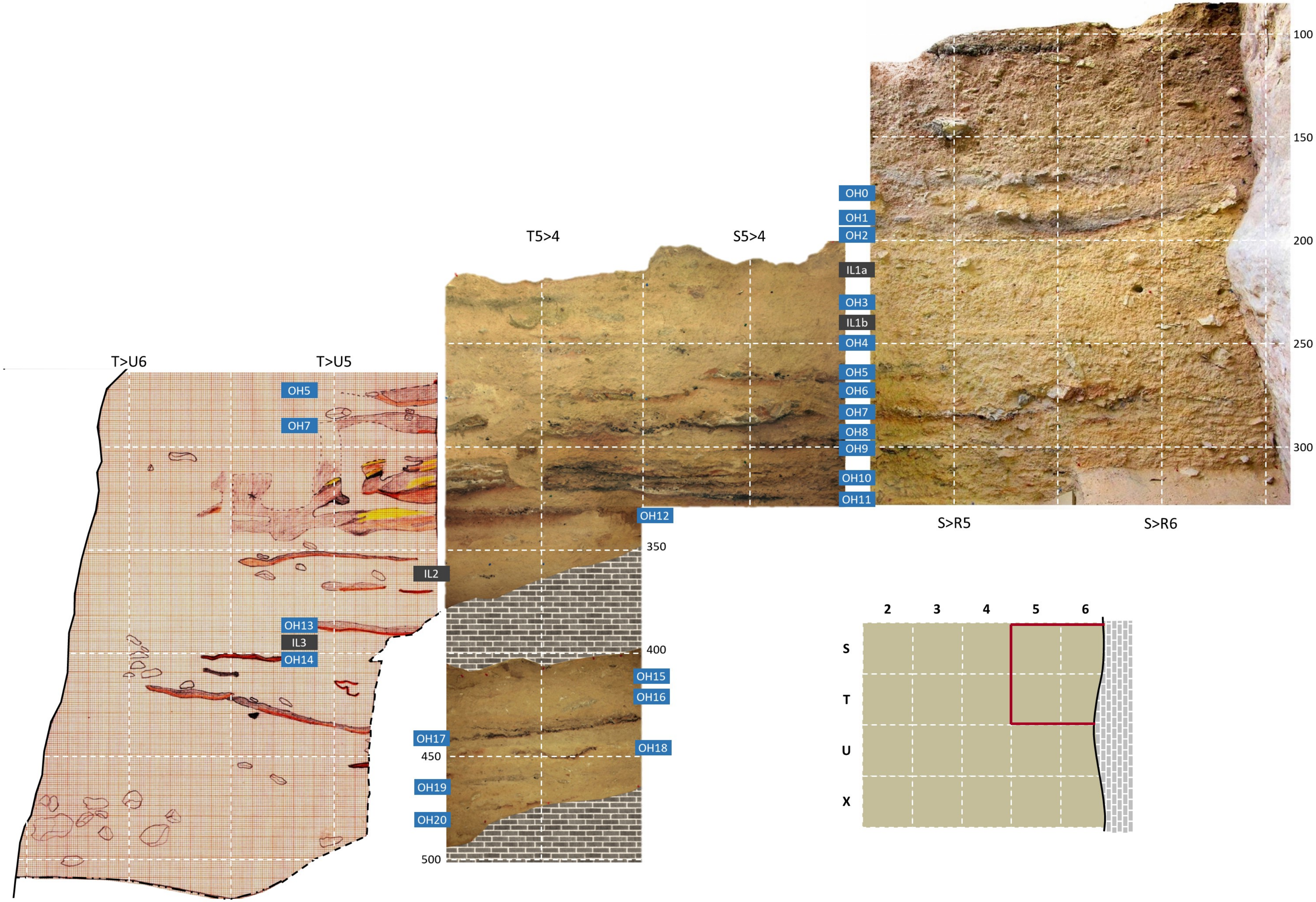

Fig. S4.6. La Boja. Stratigraphic cross-sections as recorded at the end of the 2012 field season. Orthorectified photomosaics and drawing (original 1:10). Elevations are in cm below datum. The position of the cross-sections in the grid is indicated in plan view.

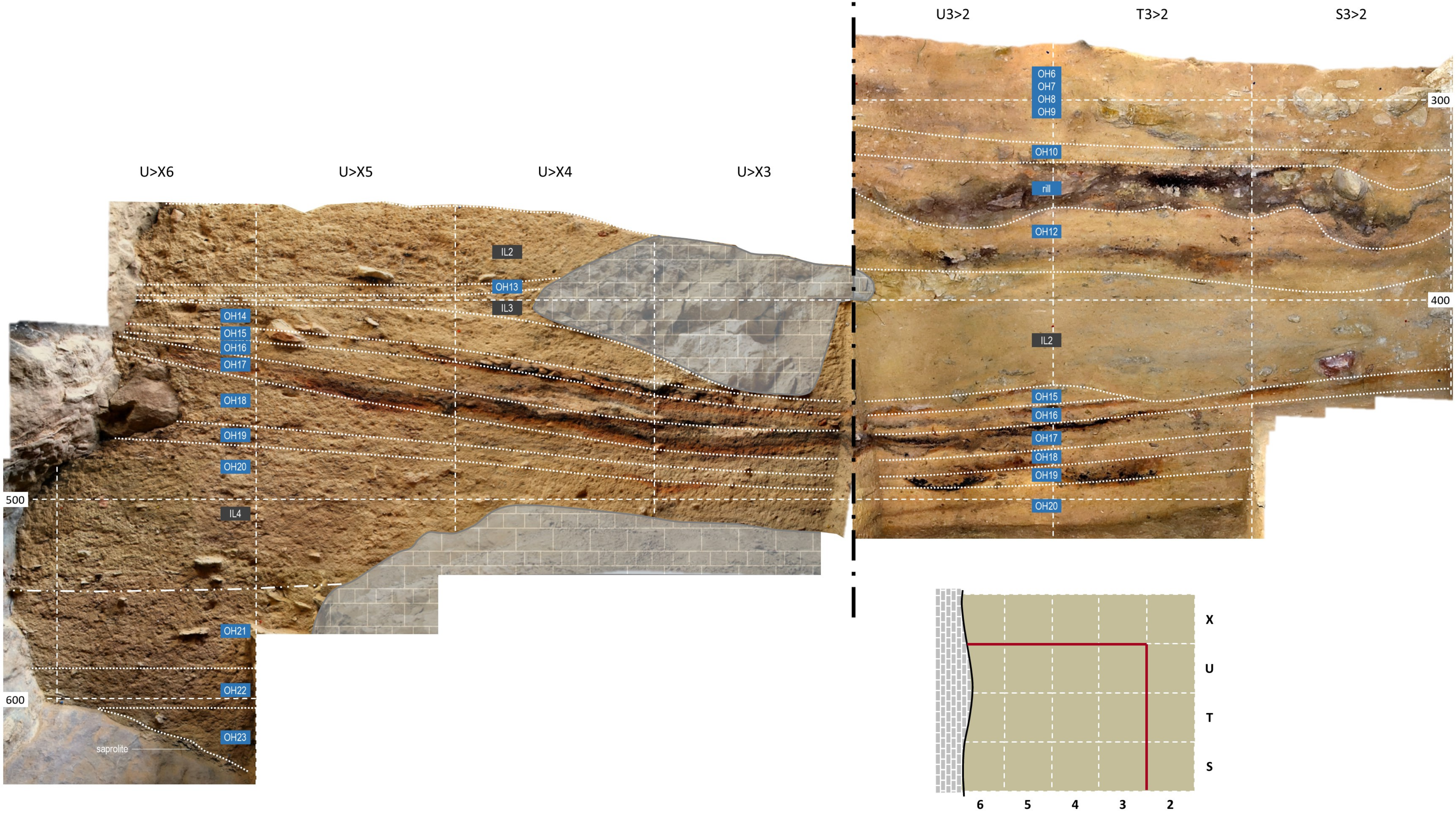

Fig. S4.7. La Boja. Stratigraphic cross-sections in the deeper trenches as recorded at the end of the 2014 field season. Orthorectified photomosaics. Elevations are in cm below datum. The position of the cross-sections in the grid is indicated in plan view.

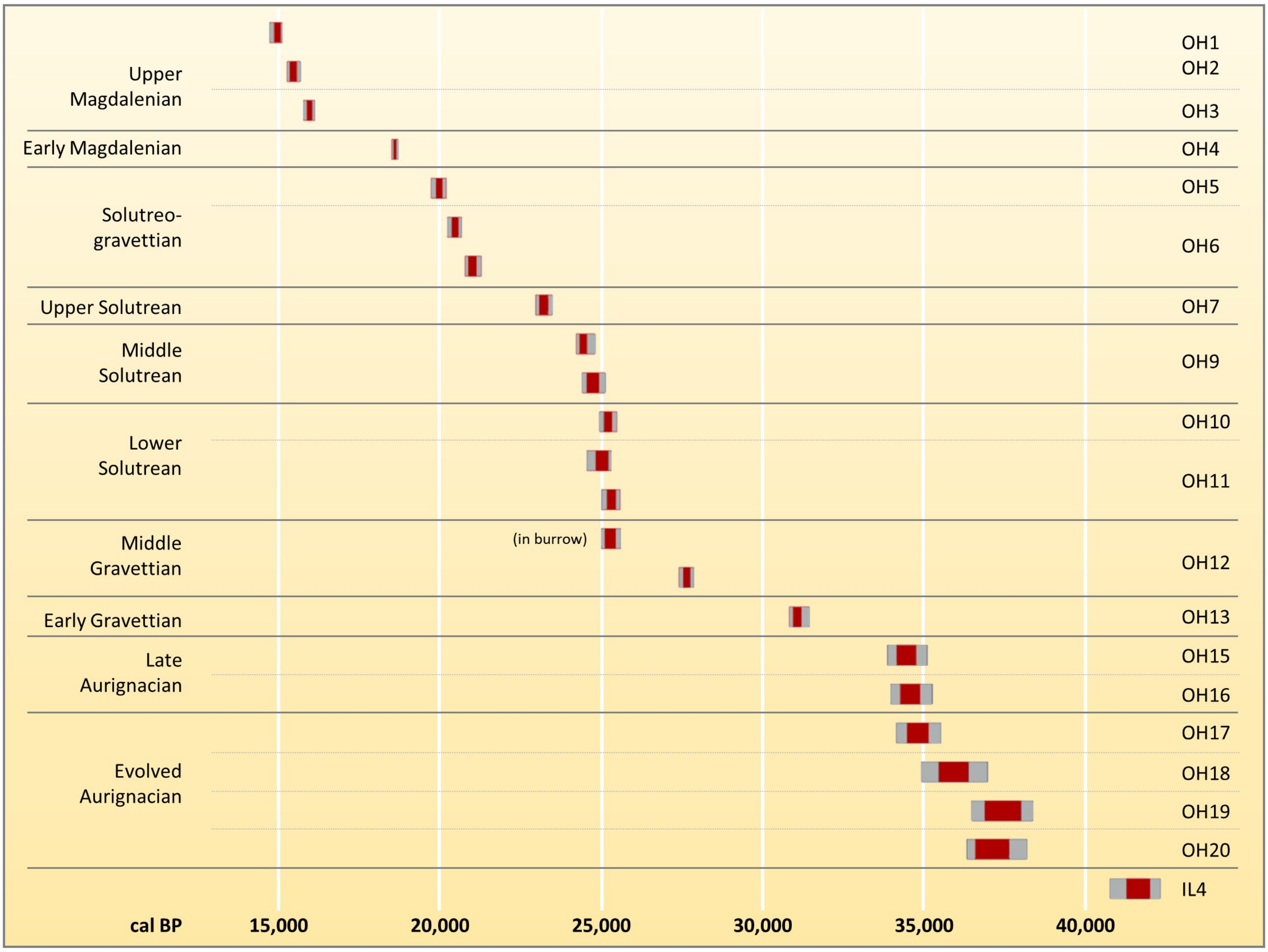

Fig. S4.8. La Boja. Plot of the 95.4% probability intervals of calibrated radiocarbon dates for the Upper Paleolithic sequence. Results on humic acids have been disregarded. Except for the date obtained on a sample from the testing phase, collected in an area where, after widening of the trench, the sediment was recognised as burrow fill, the dates are all in consistent stratigraphic order.

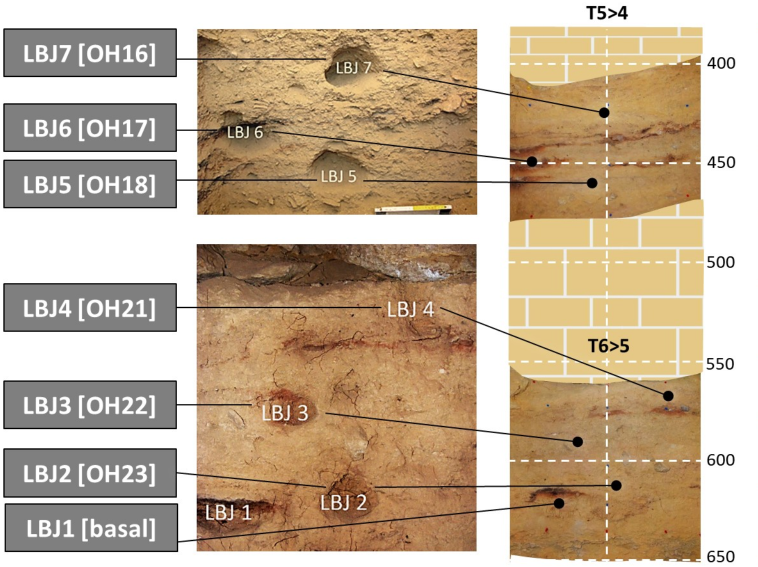

	Occupation Horizon	Sample Lab #	Age (ka)	95.4% probability interval (ka)
LBJ6	OH17	C-L3906	32.6±1.9	28.8-36.4
LBJ5	OH18	C-L3905	35.8±2.8	30.2-41.4
LBJ4	OH21	C-L3904	51.5±4.5	42.5-60.5
LBJ3	OH22	C-L3903	36.0±3.9	28.2-43.8
LBJ2	OH23	C-L3902	49.3±3.7	41.9-56.7
LBJ1	basal	C-L3901	57.7±3.2	51.3-64.1

Fig. S4.9. La Boja. Stratigraphic provenience of the OSL samples and age of the measured ones. The dates are quartz multiple-grain results, expressed as the arithmetic mean of all measurements for each sample. Standard deviations are given at 1σ. The sediment samples were collected in April 2014 from exposed cross-sections. The photographic positioning of the samples is linked to an orthorectified photomosaic of those cross-sections as previously recorded (at the end of the field season of the Spring of 2013). Sample LBJ3 is an outlier. The 95.4% probability intervals of the other results agree with those obtained by radiocarbon for the same horizons.

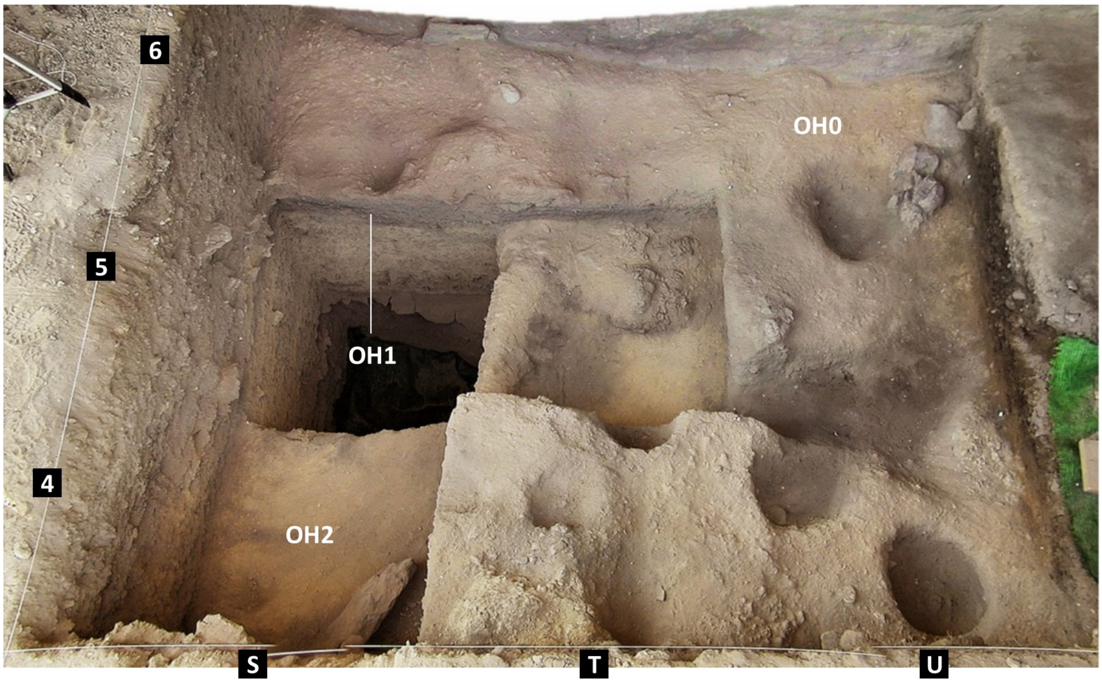

Fig. S4.10. La Boja. Bioturbation features at the interface between the Pleistocene sequence and the Holocene soil (2009 field season). The interface, which corresponds to the top of the OH0 occupation horizon, is exposed in grid units S-U/6, U5 and T-U/4, while the surface of OH2 is exposed in S4 and T5 (S5 had been taken another meter deeper during the previous season); the dark band constituting OH1 is well apparent in the S-T5>6 cross-section. The negative features — filled with an organic matter-rich, dark sediment containing abundant land snail shell and charcoal — relate to bioturbation (namely, burrowing), not human activity.

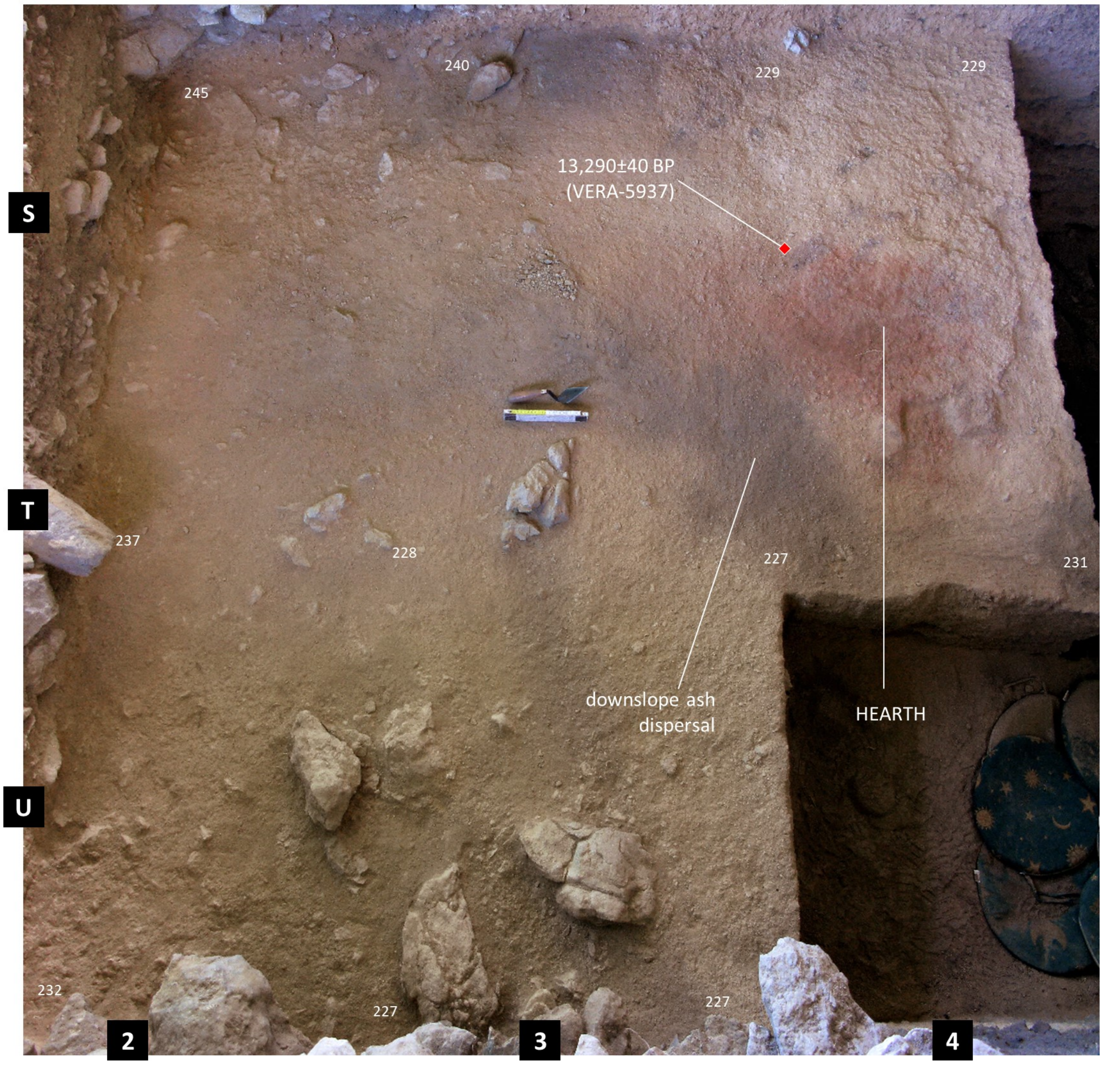

Fig. S4.11. La Boja. The A6 décapage of 2014 (base of OH3). The associated lithics are undiagnostic, but the radiocarbon date indicates an Upper Magdalenian age for this occupation. Elevations are in cm below datum.

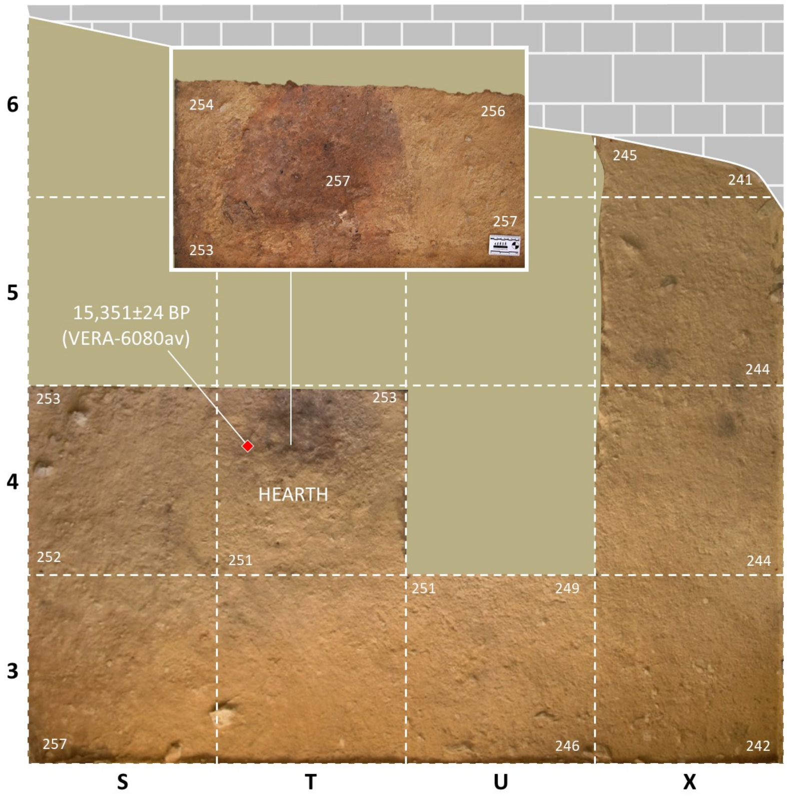

Fig. S4.12. La Boja. The A9 décapage of 2014 (base of OH4). Ortohrectified plan view; the inset is a non-rectified zenithal view over the fire-reddened base of the excavated hearth feature. The associated lithics and the radiocarbon dated sample whose provenience is indicated establish an Early Magdalenian age for this occupation. Elevations are in cm below datum.

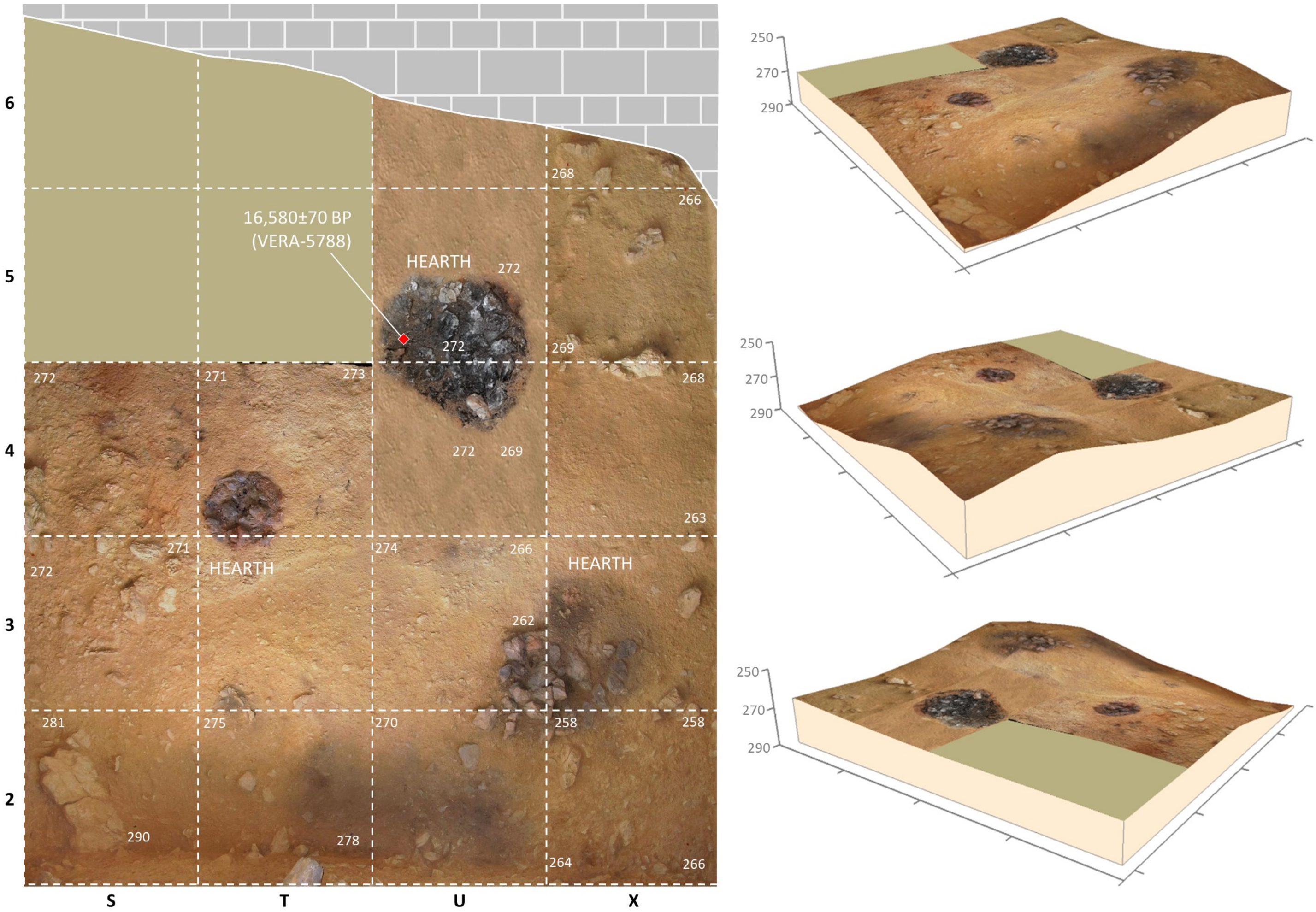

Fig. S4.13. La Boja. The A12 décapage of 2014 (base of OH5). Left. Ortohrectified plan view (the image for U/4-6 is from the equivalent spit of the 2012 field season). **Right.** 3D oblique views (elevation ×2). Elevations are in cm below datum. The associated lithics and the radiocarbon dated sample whose provenience is indicated establish a Solutreo-gravettian age for this occupation.

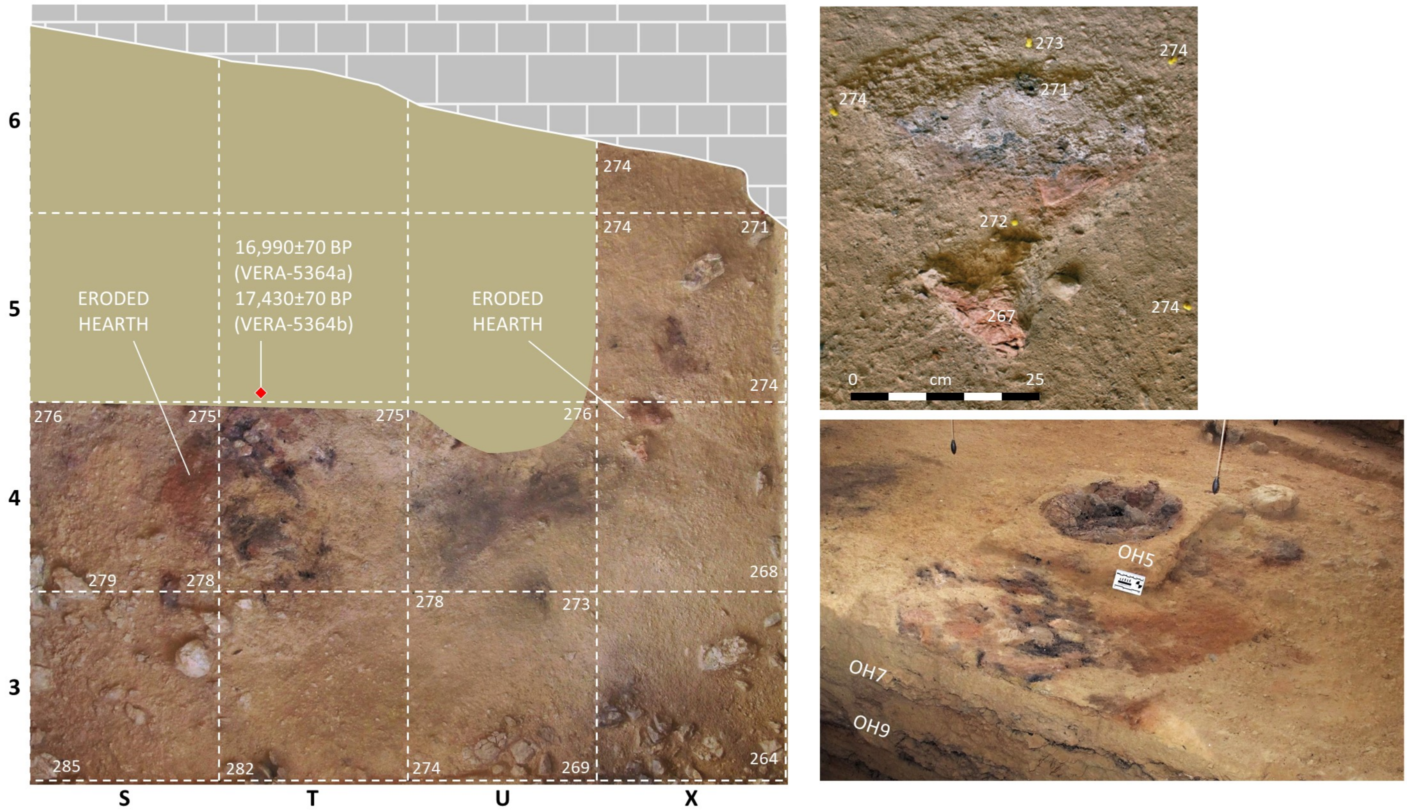

Fig. S4.14. La Boja. The A13 décapage of 2014 (base of OH6). Left. Orthorectified plan view. **Top right.** Orthorectified plan view of hearth cut by erosion half-way through the A12 décapage in X/4-5. **Bottom right.** Oblique view, from behind the S-T5>4 cross-section, of the stratigraphic position of the eroded hearth in S-T/4 and the OH6 surface it belongs to, relative to over- and underlying horizons. Elevations are in cm below datum. The associated lithics are undiagnostic. The radiocarbon dates indicate that this horizon is a palimpsest of two different, albeit close in time, Solutreo-gravettian occupation lenses.

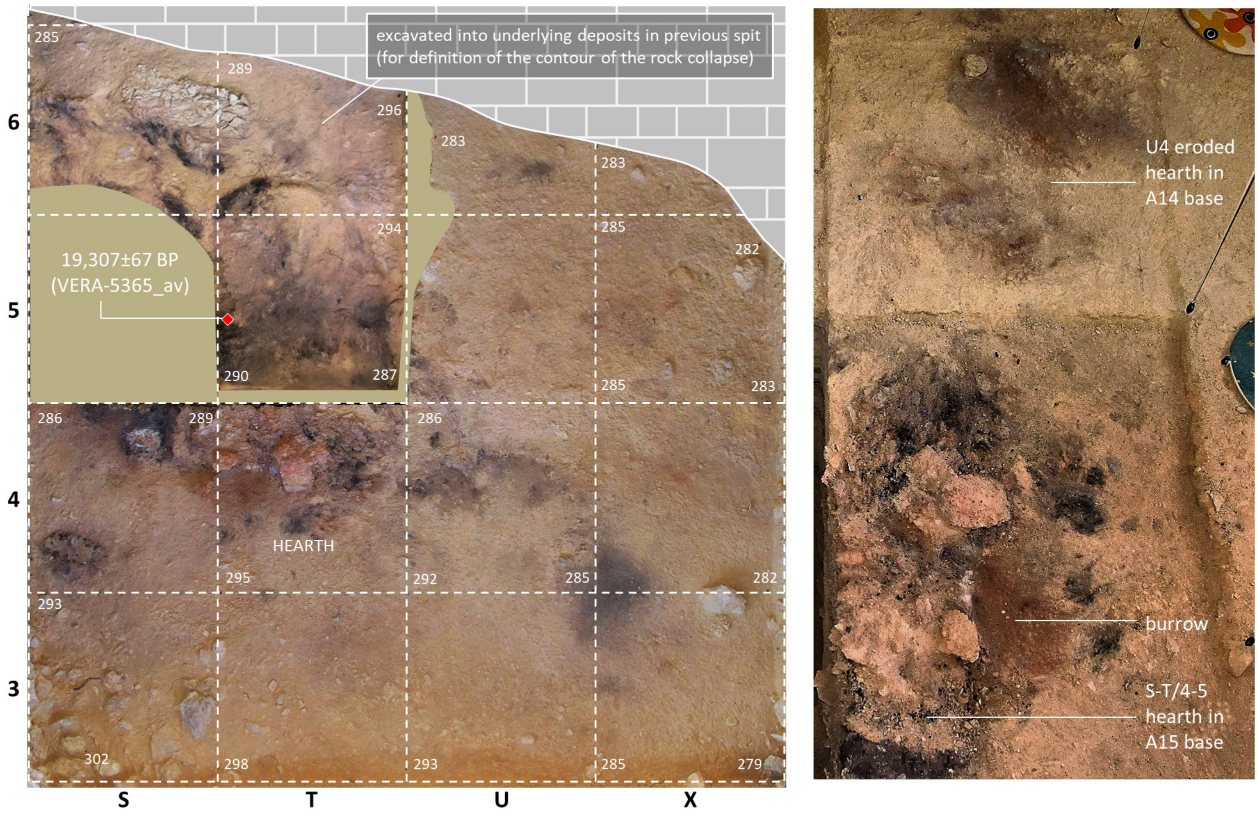

Fig. S4.15. La Boja. The A15 décapage of 2014 (middle of OH7). Left. Orthorectified plan view; the image for S-T/5-6 is from the equivalent spit of the 2010 field season. Elevations are in cm below datum. **Right.** Oblique view over the main hearth of OH7 during the A15 décapage in S-U/4; note, in U4, at this stage still showing the surface of A14, the eroded hearth belonging to the uppermost occupation lens of OH7. The associated lithics and the radiocarbon dated sample whose provenience is indicated establish an Upper Solutrean age for this occupation.

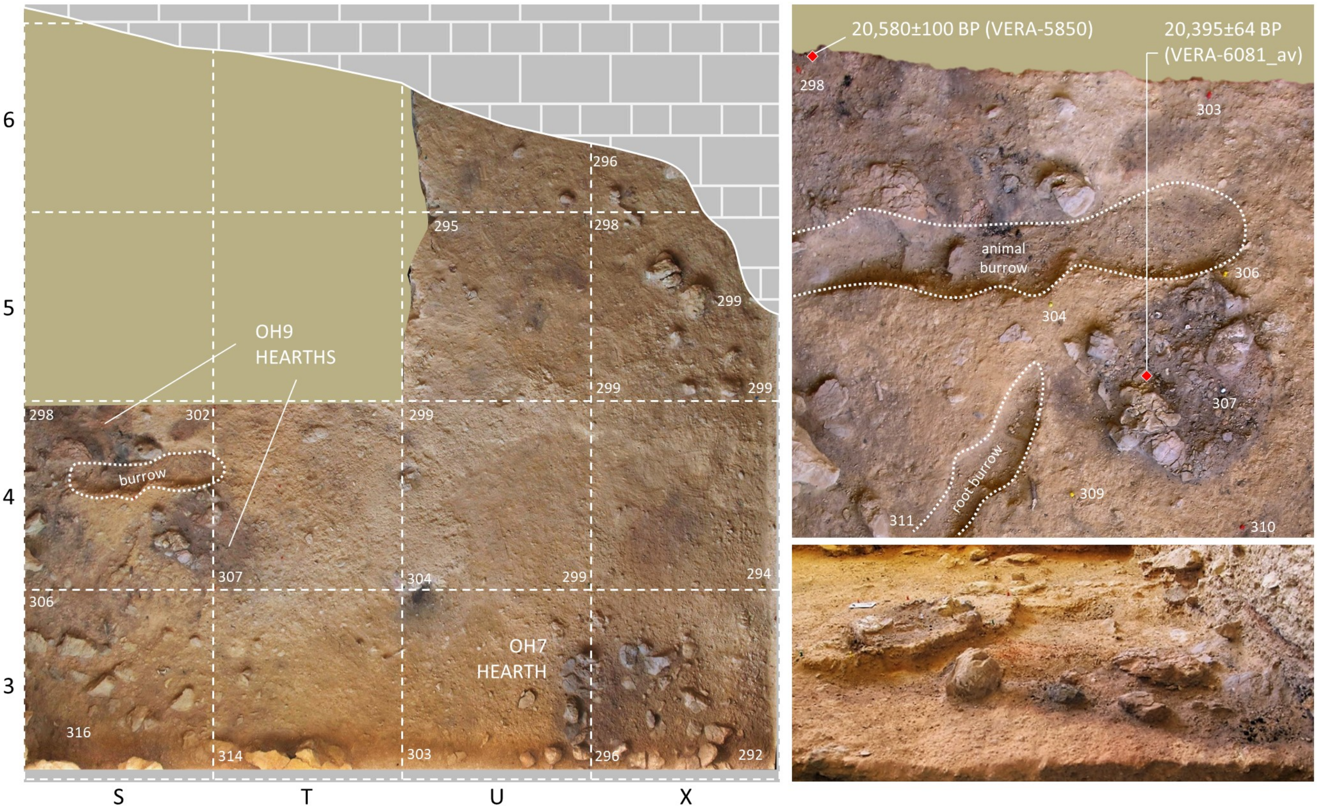

Fig. S4.16. La Boja. The A17 décapage of 2014 (OH7/OH9). Left. Orthorectified plan view of A17middle. In S-T/4, the outcropping hearths denote the OH9 paleo-surface. OH8 is a thin lens present in S-T/5-6 only. Elevations are in cm below datum. **Top right.** Orthorectified plan view of S4 and T4-West at A17base. **Bottom right.** Oblique view, from behind the S5>4 cross-section, during the excavation of A17base, of the relationship between the two OH9 hearths; although topographically higher, the hearth in S/4-5 stratigraphically underlies that in S-T/4. The associated lithics and the radiocarbon dated samples whose provenience is indicated establish a Middle Solutrean age for the OH9 occupation.

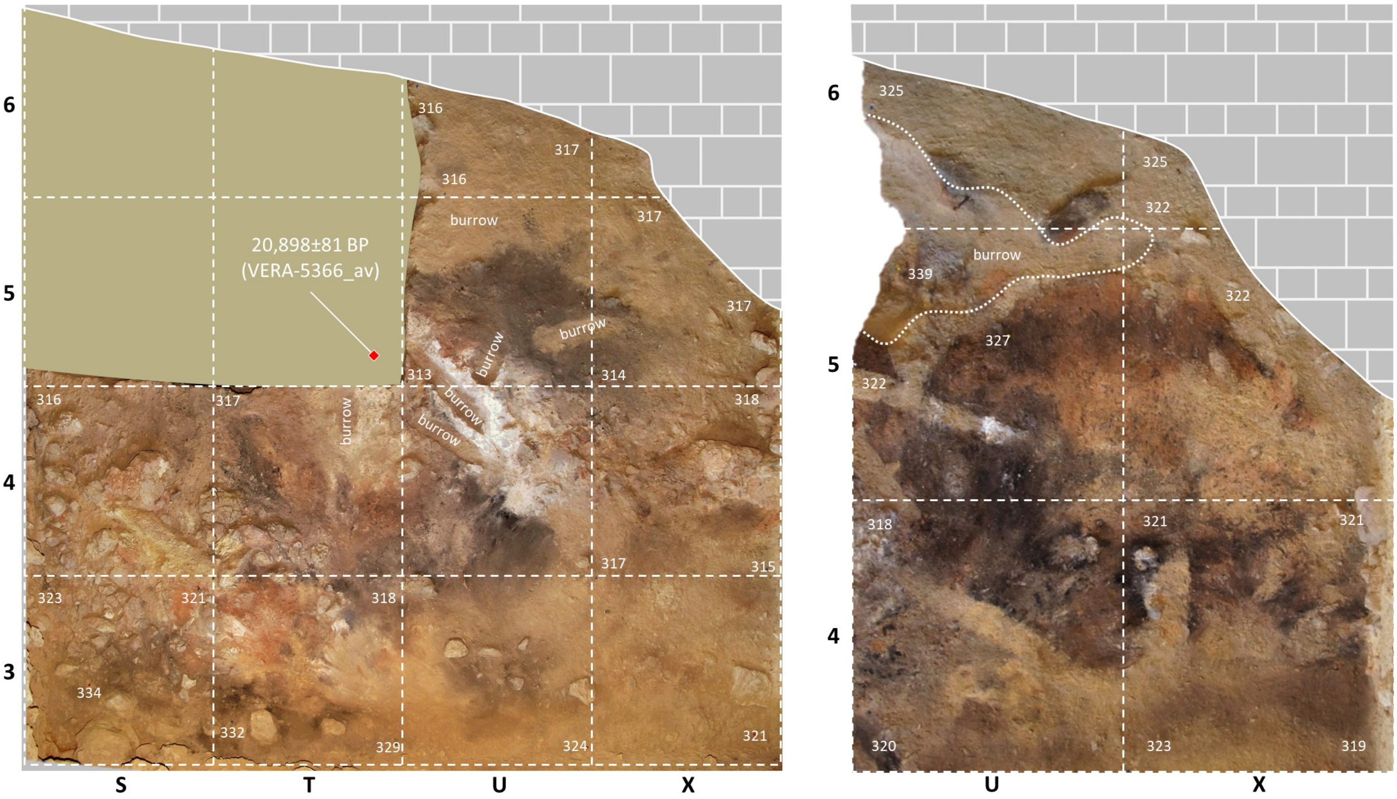

Fig. S4.17. La Boja. The A21 and A22 décapages of 2014 (OH10). Left. Orthorectified plan view of A21, which corresponds to the base of the upper occupation lens within OH10; note the numerous burrows. R**ight.** Orthorectified plan view of A22 in U-X/4-6, which corresponds to the basal occupation lens within OH10, defined by the palaeosurface upon which the large, ash-capped hearth outcropping in A21 was lit . Elevations are in cm below datum. The associated lithics and the radiocarbon dated sample whose provenience is indicated establish a Lower Solutrean age for this occupation horizon.

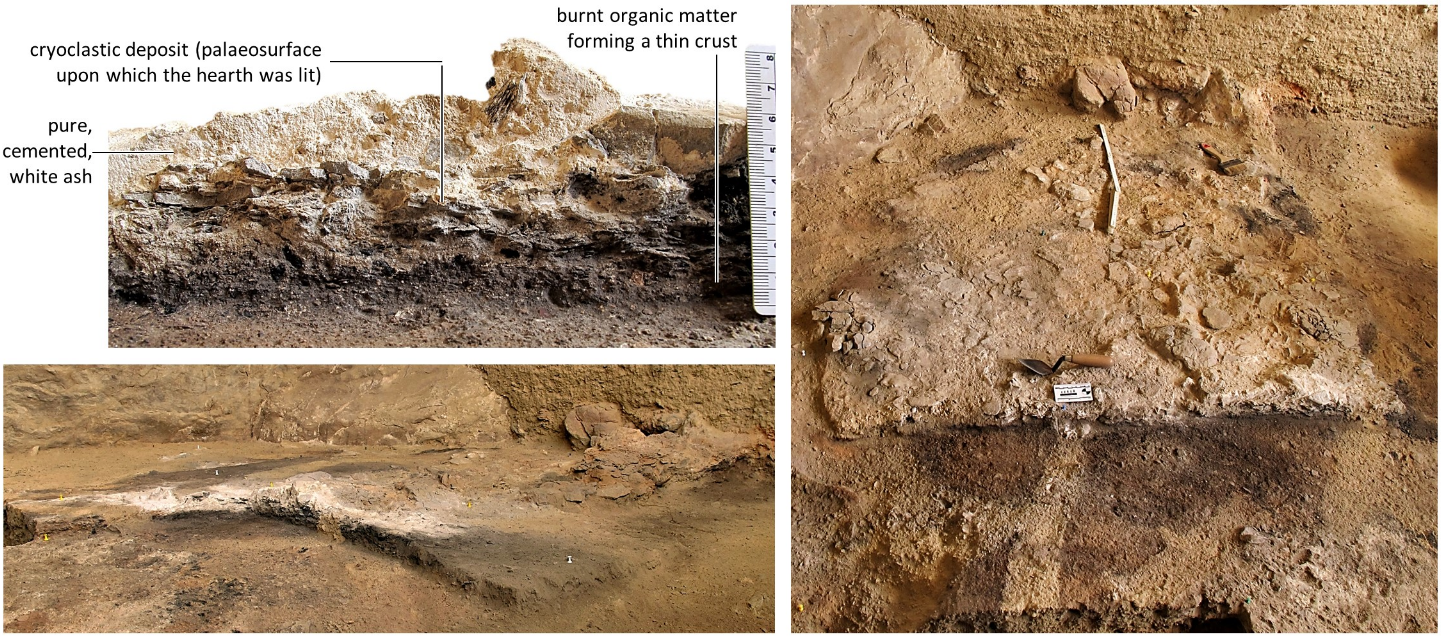

Fig. S4.18. La Boja. Excavation of the hearth at the base of OH10 (décapage A22). Bottom left. Tangential view from T4 at the end of the first step (excavation of a pie-cut to assess the feature's internal micro-stratigraphy). **Top left.** Microstratigraphic detail. **Right.** Oblique overview taken from behind the T>U5 cross-section when the feature had already been cut in half; note the fire-reddened palaeosurface and the burrows that traverse it.

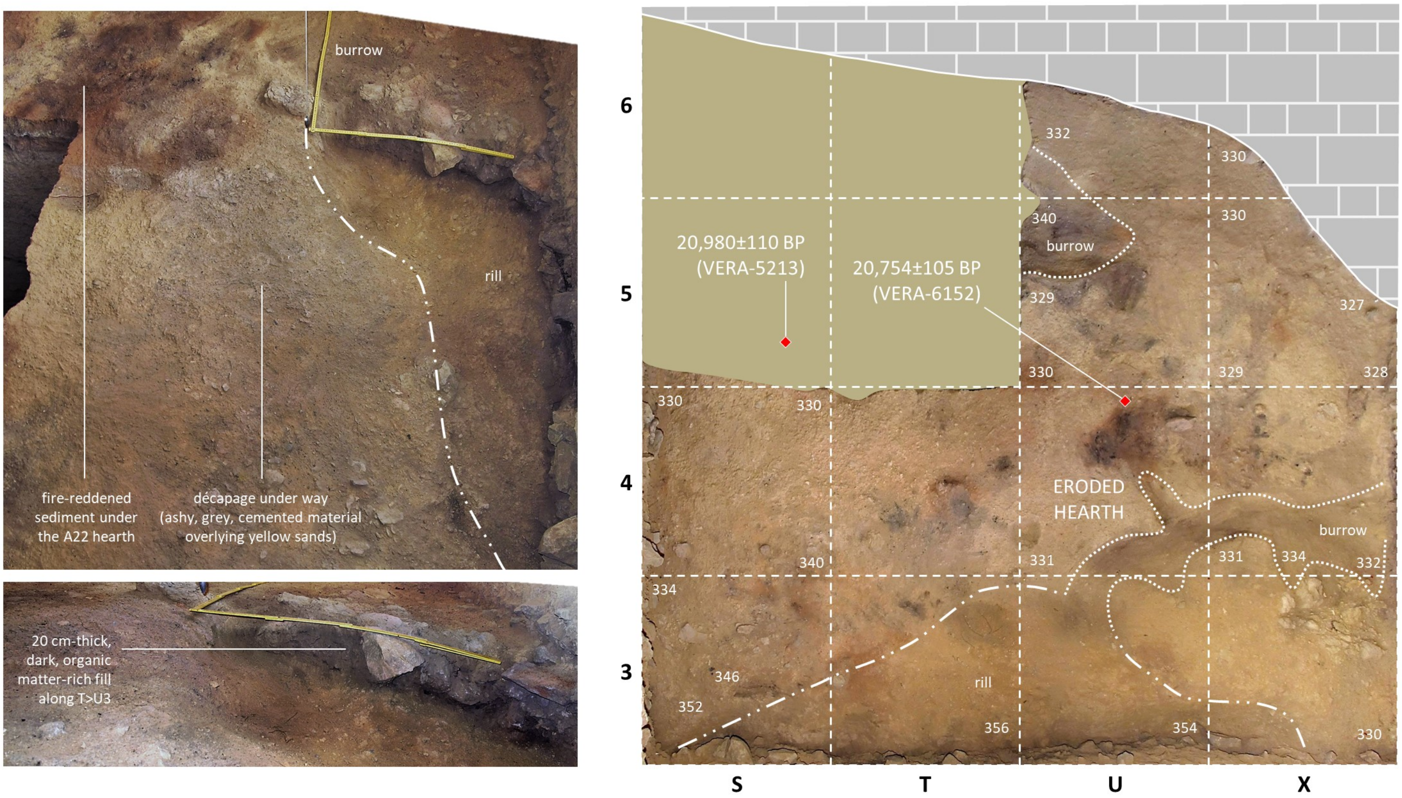

Fig. S4.19. La Boja. Excavation of OH11 (décapages A23-A25). Top left. Oblique view from the western edge of the trench over the excavation of A23. **Bottom left.** Detail cross-section view of the fill of the major erosional feature detected in row 3. **Right.** Orthorectified plan view of A24. Elevations are in cm below datum. The associated lithics and the radiocarbon-dated samples whose provenience is indicated establish a Lower Solutrean age for this occupation horizon.

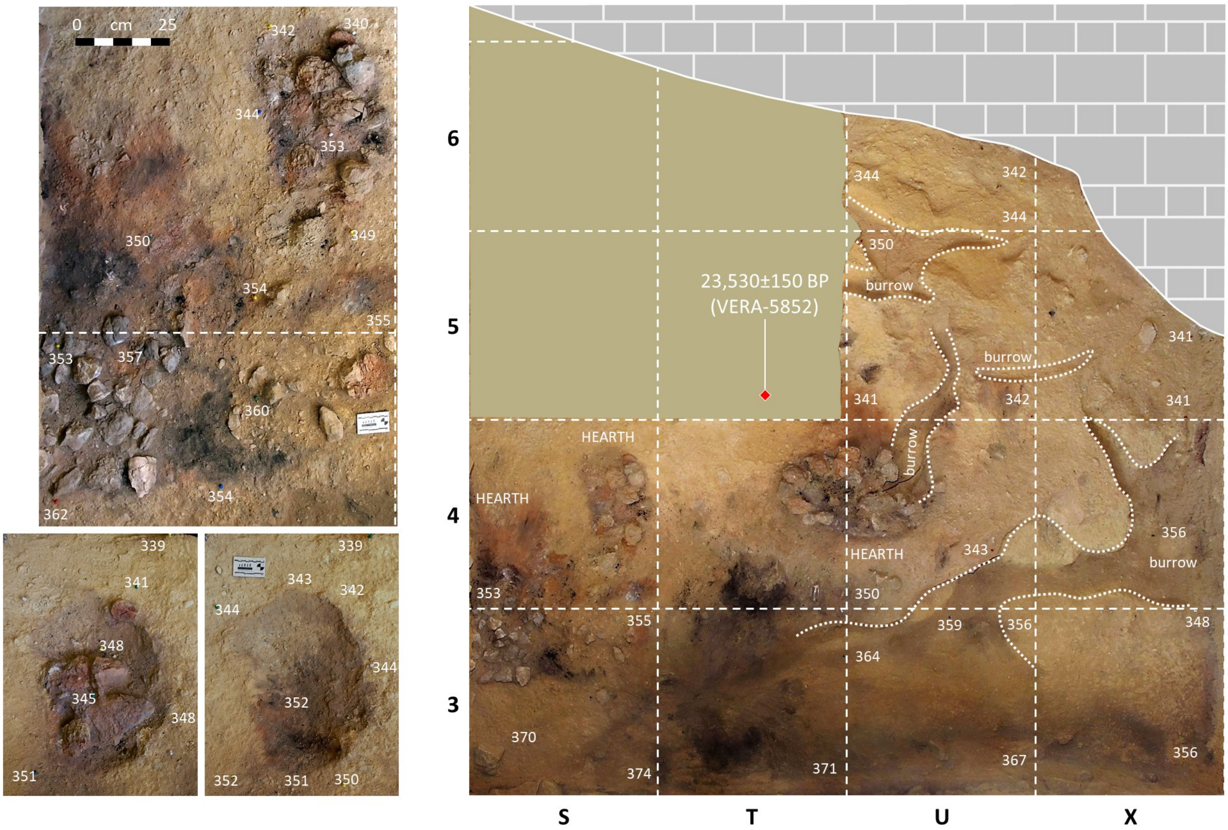

Fig. S4.20. La Boja. Upper palaeosurface (décapage A27 of 2014) within OH12. Right. Orthorectified plan view of A27. **Top left.** Orthorectified close-up view of the hearths in S/3-4. **Bottom left.** Orthorectified plan views of the two steps in the excavation of the stone-filled, subsurface basin featured by the hearth in S4NE. Elevations are in cm below datum. The associated lithics and the radiocarbon-dated sample from the ash- and charcoal-rich lens covering the hearth in T-U/4 (excavated as décapage A26), whose provenience is indicated, establish a Middle Gravettian age for this occupation horizon.

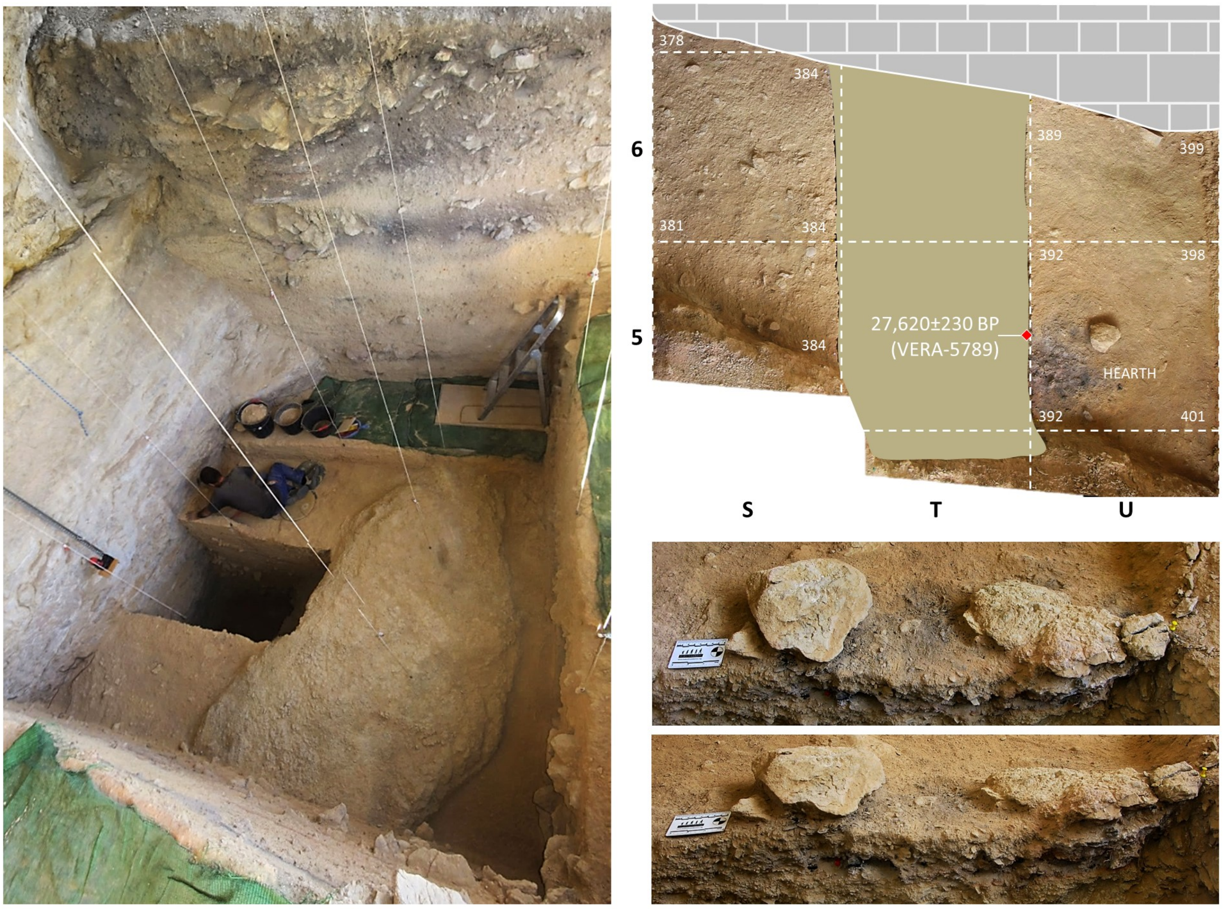

Fig. S4.21. La Boja. Late Aurignacian to Early Gravettian roof collapse and OH13 occupation. Left. The IL2 deposit under excavation prior to demolition of the fallen slab. **Top right.** Orthorectified plan view of décapage A36 (OH13). Elevations are in cm below datum. **Bottom right.** The OH13 hearth in T-U/5 overviewed from behind the T>U5 cross-section prior to exposure of the burnt lens under the stones. The radiocarbon-dated sample whose provenience is indicated establishes an Early Gravettian age for this horizon.

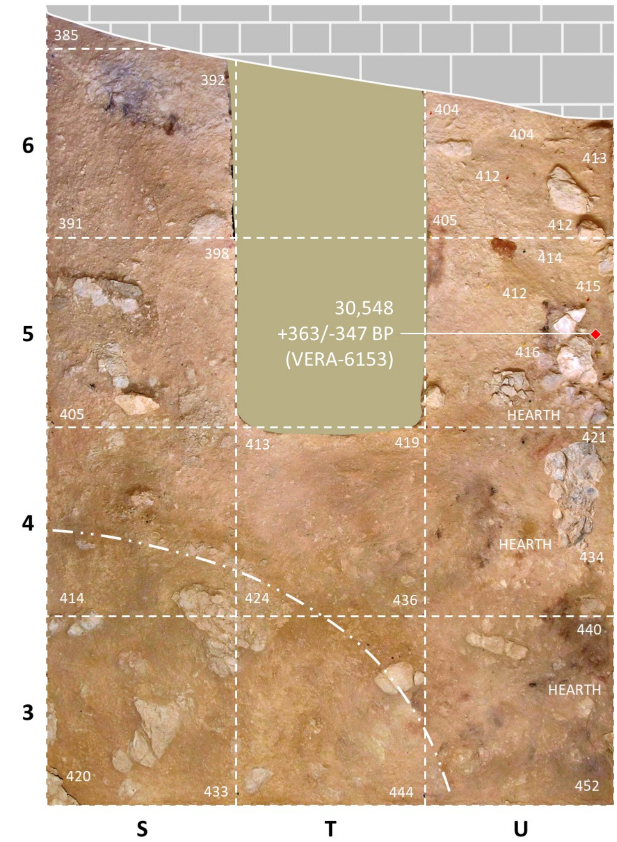

Fig. S4.22. La Boja. The A40top décapage of 2014 (OH15). This palaeosurface corresponds to the most recent occupation lens found in the upper part of the Aurignacian sequence. The overlying A39 sediments form the undated OH14 horizon, which yielded a microgravette. Disaggregated remnants of the collapse that sealed the Aurignacian are apparent in the SW corner of the trench. Orthorectified plan view. Elevations are in cm below datum.

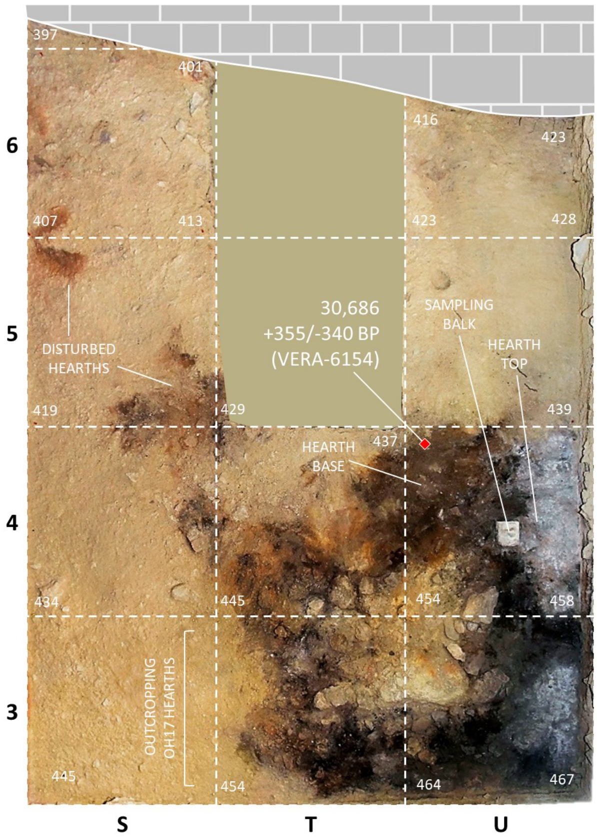

Fig. S4.23. La Boja. The A41 décapage of 2014 (OH16). First of two décapages into the basal occupation lens found in the upper part of the Aurignacian sequence. The thin deposit of silts seen around the SW corner of U4 was left unexcavated to document the microstratigraphic pattern; it separates this lens from the features defining the surface of the lower part of the Aurignacian sequence (OH17). Orthorectified plan views. Elevations are in cm below datum.

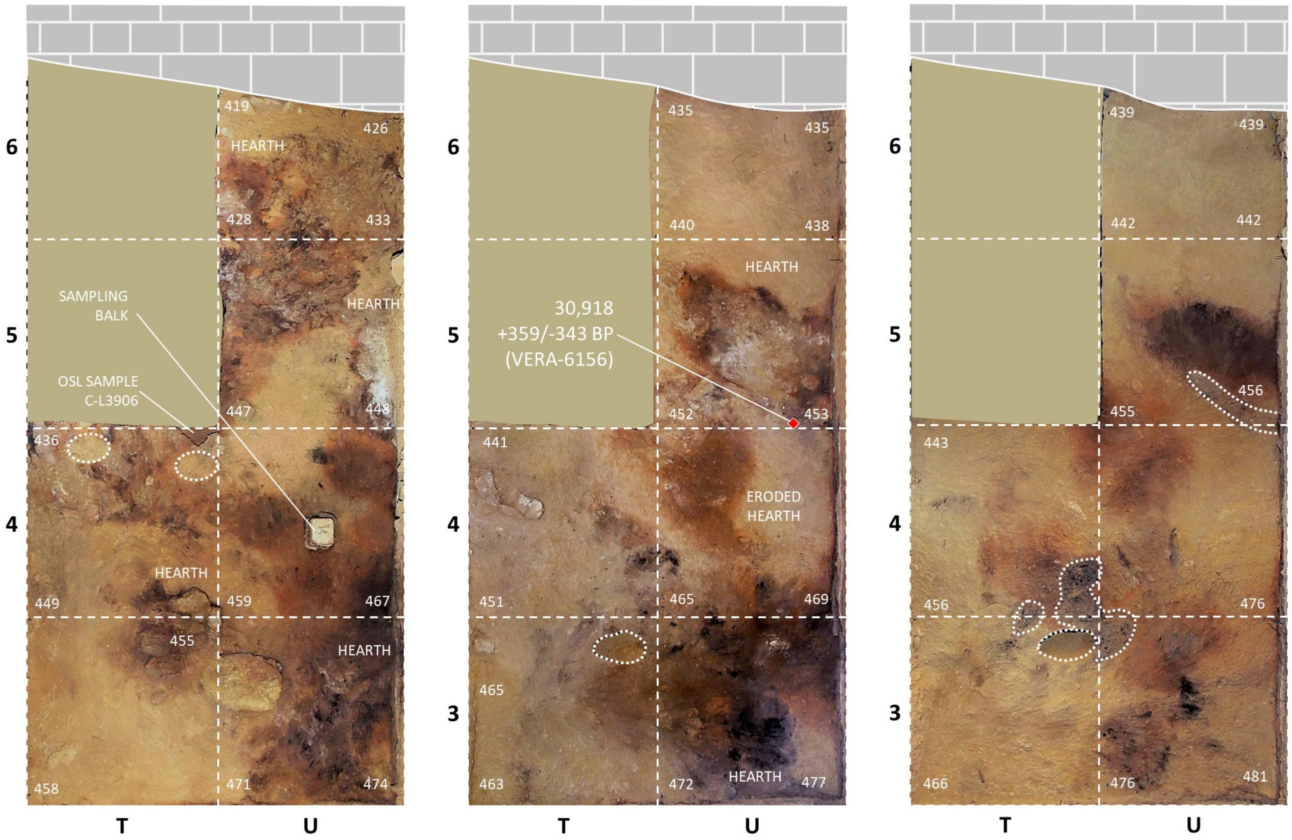

Fig. S4.24. La Boja. The three-décapage, 2014 excavation of OH17. Left. A43. Middle. A44. Right. A45. Elevations are in cm below datum. Burrowed areas are indicated by dotted contour lines. Note, in A45, the sediment reddening caused by the fire features present in A43-A44; nearly sterile, A45 neatly separated OH17 from the underlying OH18 horizon.

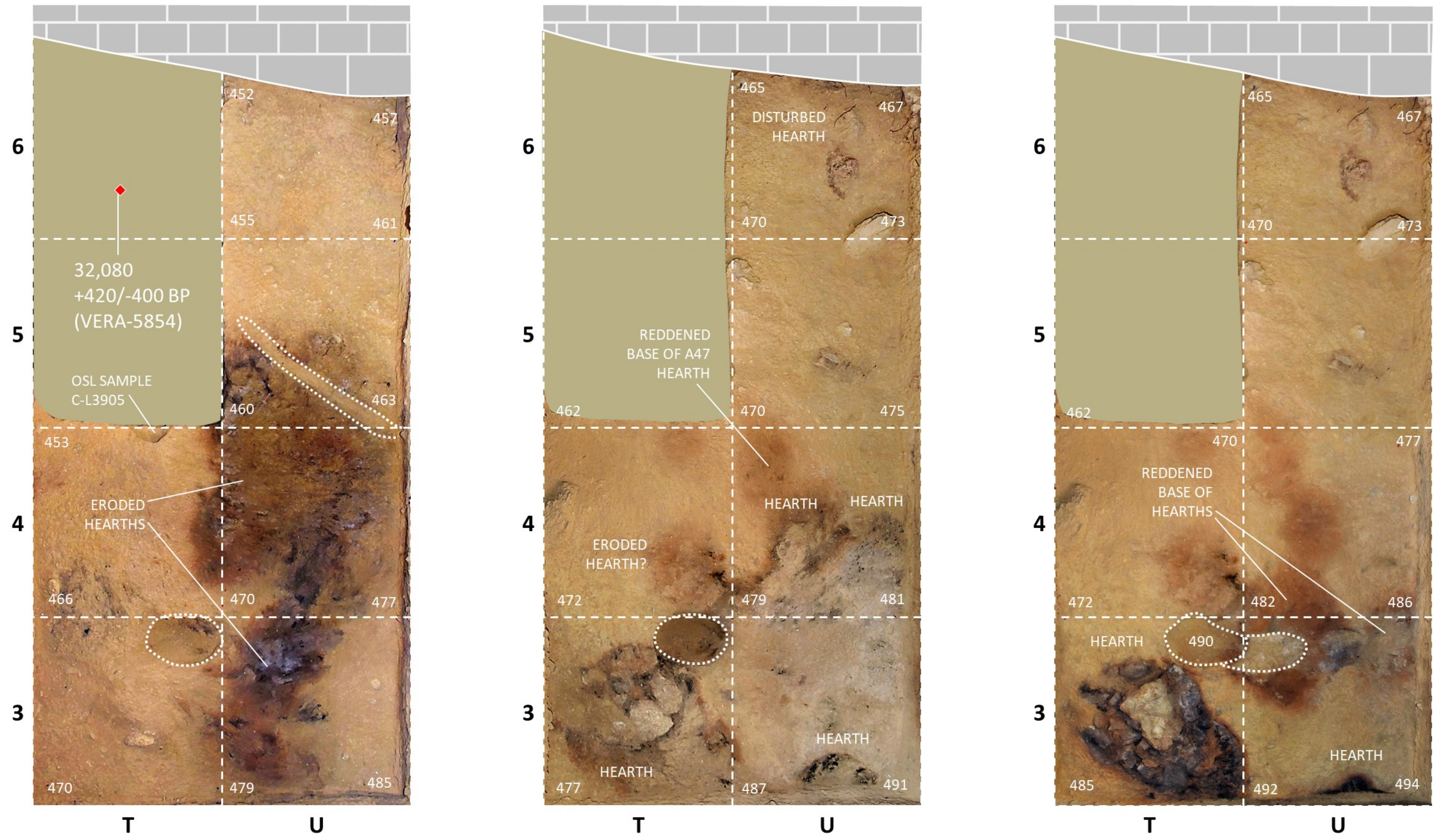

Fig. S4.25. La Boja. The 2014 excavation of OH18 and OH19. Left. The A46 décapage (OH18). **Middle.** Half-way through the A48 décapage (OH19). **Right.** The A48 décapage (OH19) completed in T-U/3-4. Orthorectified plan views. Elevations are in cm below datum. Burrowed areas are indicated by dotted contour lines. While OH18 only yielded eroded fire features, those in OH19 were well preserved; even though subdivided in two décapages (A47 and A48), OH19 corresponds to a single occupation lens.

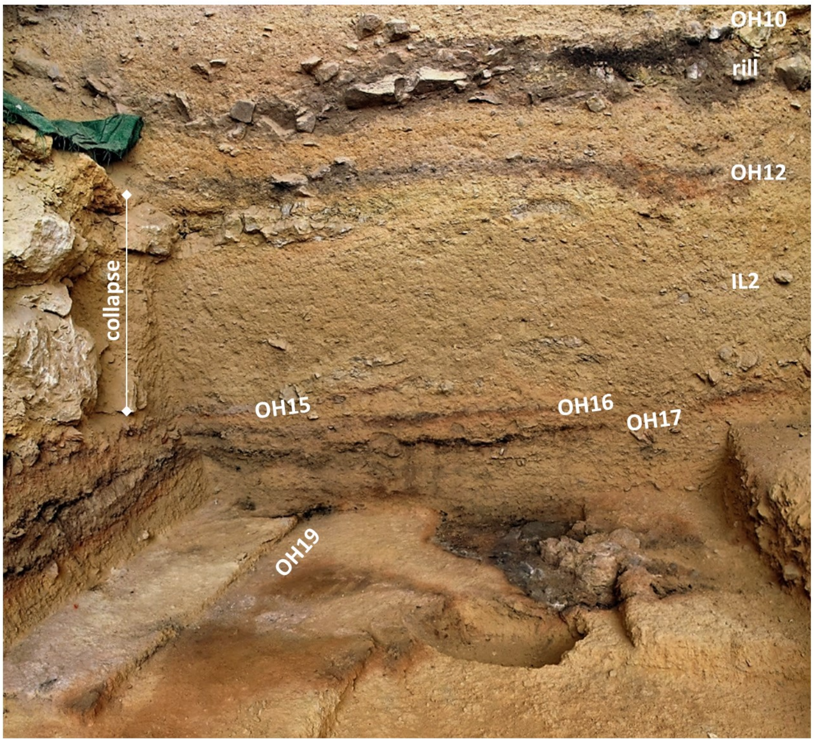

Fig. S4.26. La Boja. The A48 décapage in progress. To examine microstratigraphy, a stepping approach was followed. In the S-U3>2 cross-section, note the position of OH19 relative to overlying horizons, namely those whose position is denoted by fire lenses.

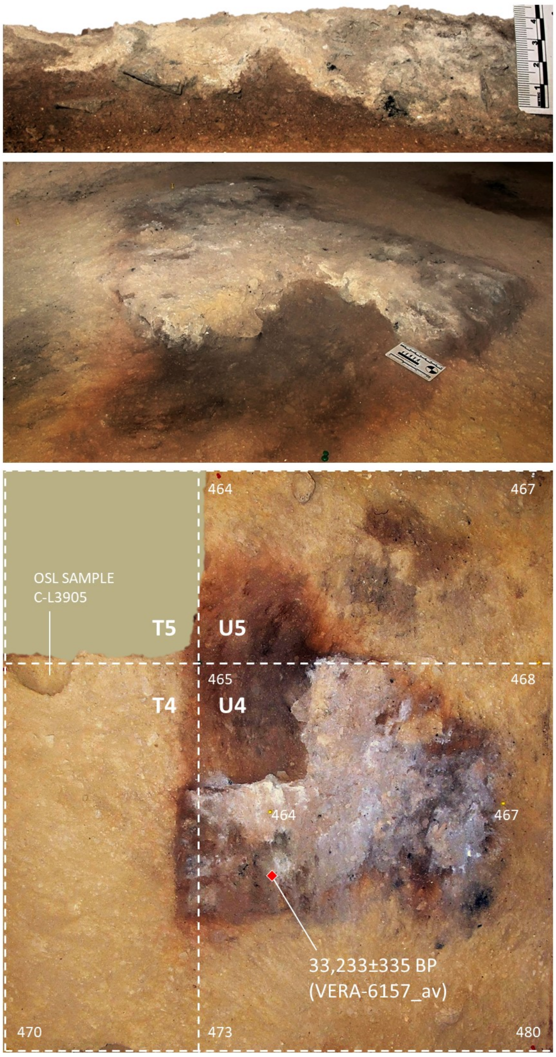

Fig. S4.27. La Boja. The hearth in spit A47 (after a pie-slice had been cut to expose microstratigraphy). Top. Detail of cross-section through the feature. **Middle.** Oblique view, from the SE corner of T5. **Bottom.** Orthorectified plan view. Elevations are in cm below datum.

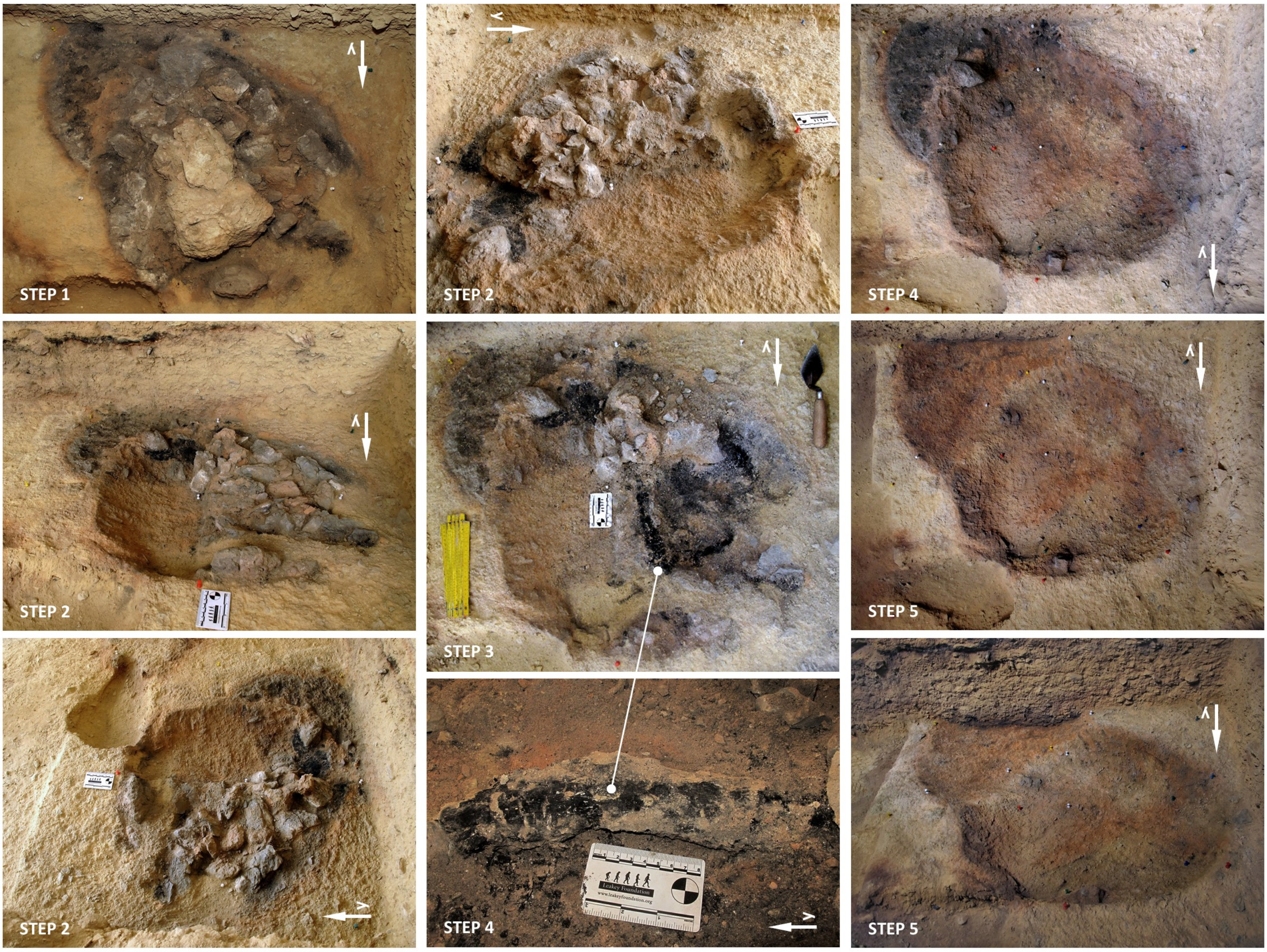

Fig. S4.28 La Boja. The double hearth of OH19 in T3. Step 1. At the end of the A48 décapage**. Step 2.** The eastern half of the larger hearth excavated to the bottom of its stone- and charcoal-filled basin**. Step 3.** Removal of the larger hearth's stone cap completed. **Step 4.** The larger hearth's western half excavated to the base of the fill; note the presence of carbonized wood fragments up to 25 cm-long and 5 cm-thick. **Step 5.** Excavation of the fill of the earlier, only charcoal-filled hearth cut by the larger, stone-filled feature.

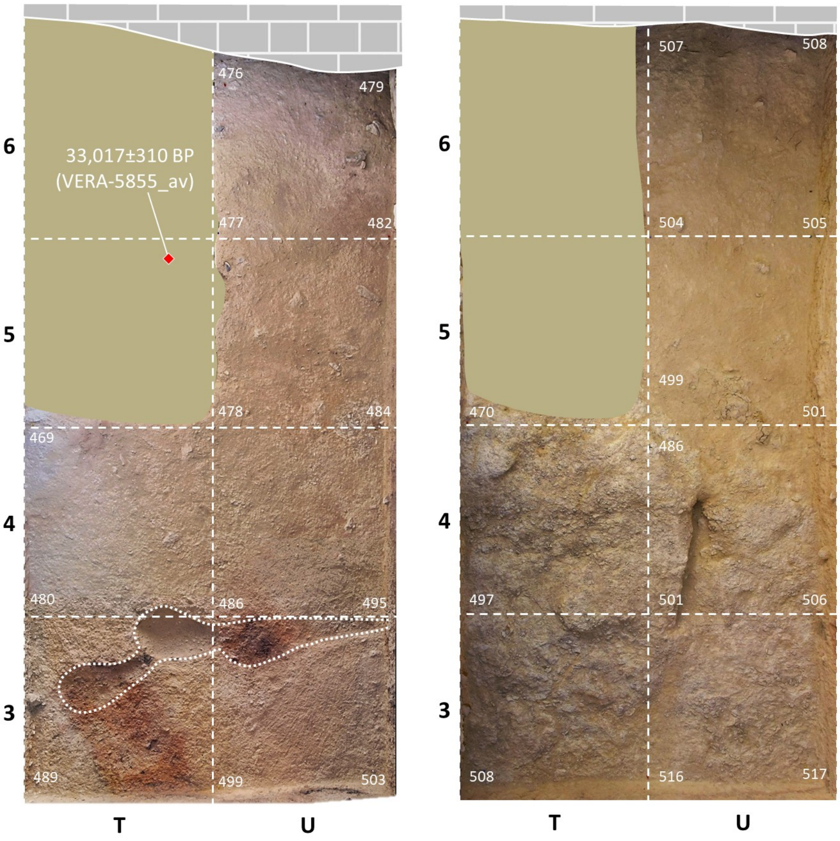

Fig. S4.29. La Boja. The OH20 occupation and the IL4 deposit. Left. The A49 décapage. Note, outcropping in the western half of T4, the large slab collapsed on top of the basal Mousterian. A49 is the uppermost spit of the two into which the excavation of the OH20 horizon was subdivided. **Right.** The A51 décapage. The upper side of the collapse is now exposed, and the sediment surface behind it is the base of this first spit into the loose and disturbed sediments that make up the IL4 horizon. Orthorectified plan views. Elevations are in cm below datum.

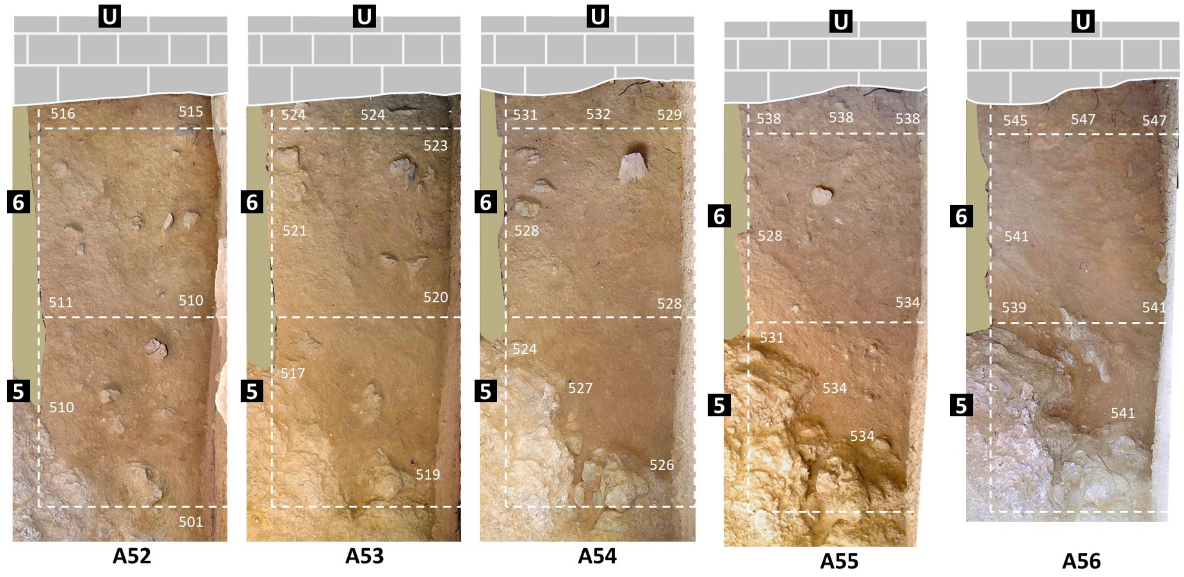

Fig. S4.30. La Boja. The IL4 deposit. The vertically oriented stones bespeak of the disturbance undergone by the sediments accumulated behind the boulder prior to the stabilization of a ground surface in OH20 times. The A56 décapage corresponds to the interface with the Middle Paleolithic. This décapage exposed a lens of finer, siltier sediments that make for a poorly defined stratigraphic boundary. Orthorectified plan views. Elevations are in cm below datum.

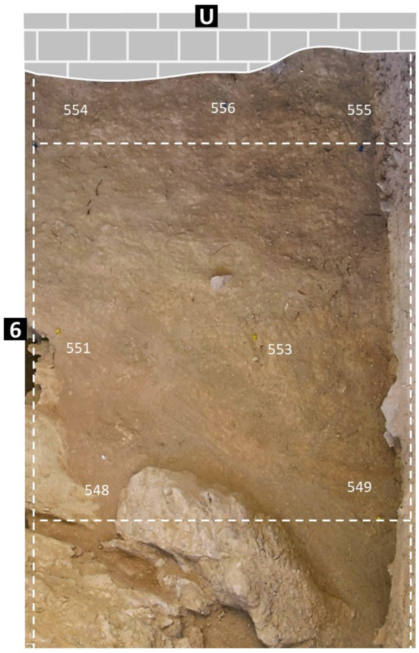

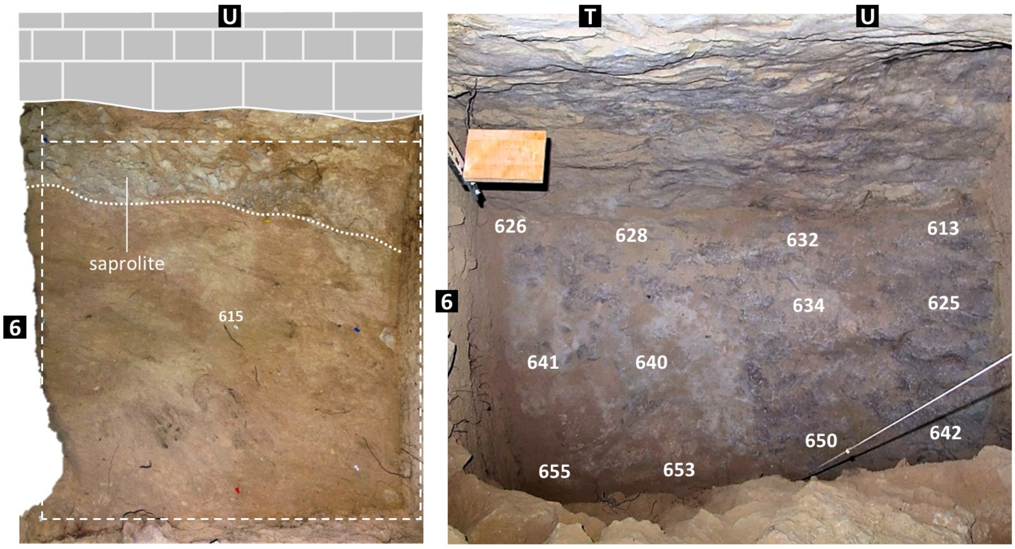

Fig. S4.31. La Boja. The Middle Paleolithic deposit. Left. The A57 décapage, base of the first spit in OH21. **Middle.** The A67 décapage within OH23. **Right**. Bedrock exposed. Left and middle are orthorectified plan views. Elevations are in cm below datum.

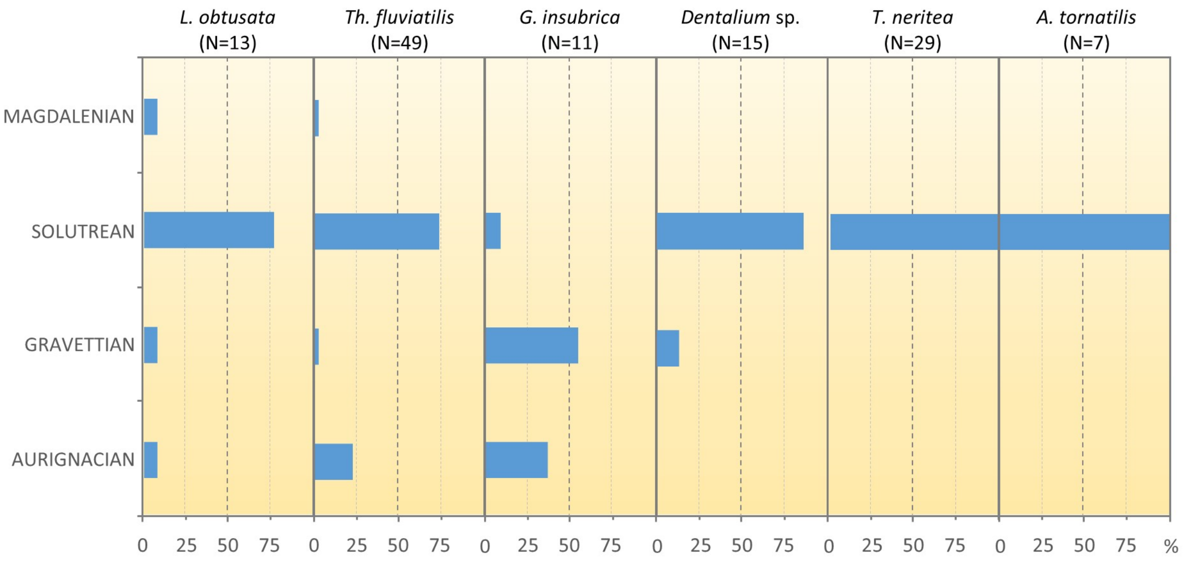

Fig. S4.32. La Boja. Shell bead percentage graph. Distribution of marine and fluviatile shell taxa represented by more than five specimens that, given the perforations present in most, can be securely interpreted as beads. The Solutrean category subsumes the Solutreo-gravettian.

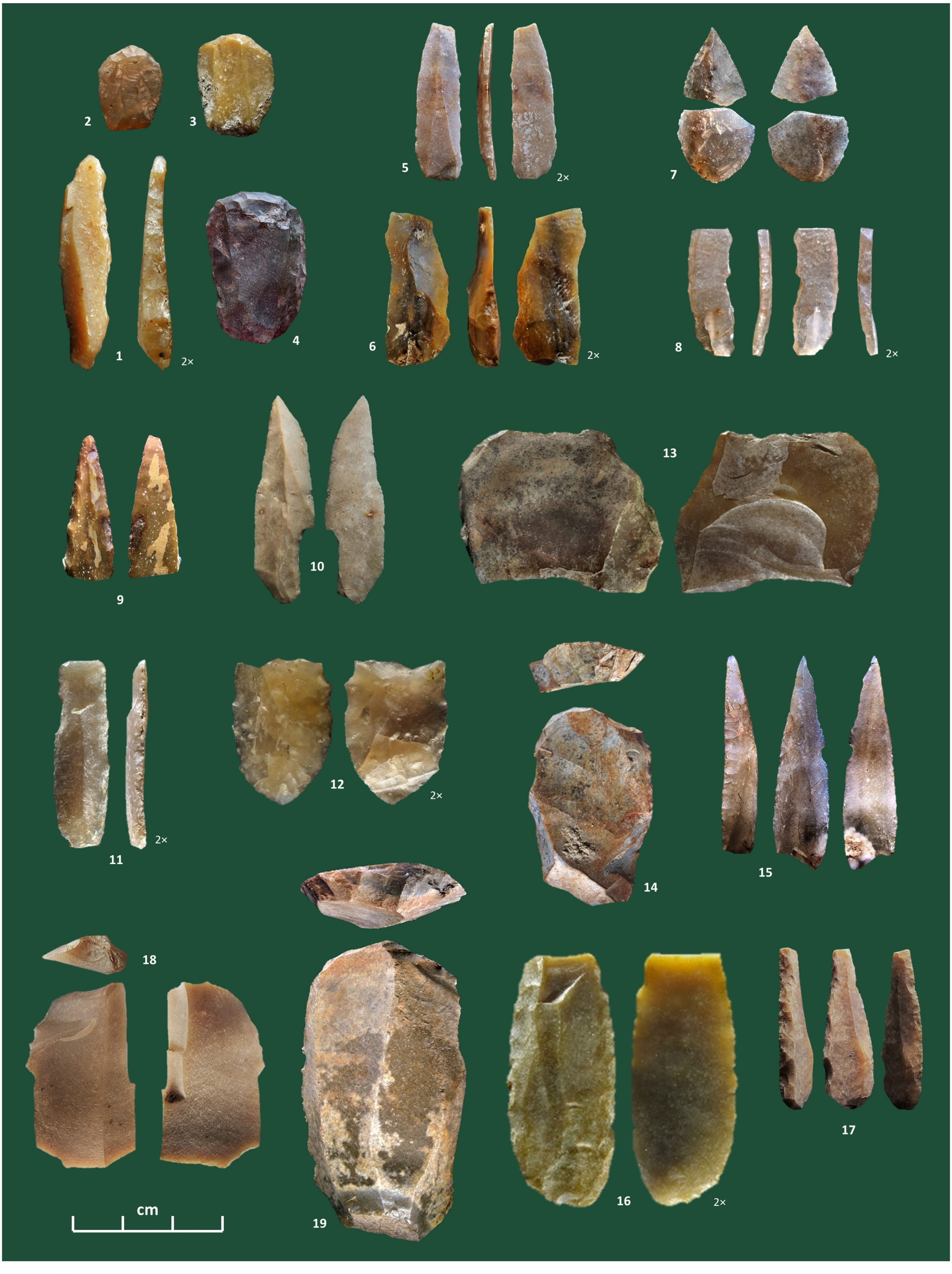

Fig. S4.33. La Boja. Mid- and Later Upper Paleolithic. Upper Magdalenian. 1. backed bladelet; 2-4. endscrapers. **Early Magdalenian.** 5-6. marginally backed bladelets. **Solutreo-Gravettian.** 7. unifacial foliate; 8. double-backed bladelet. **Upper Solutrean.** 9-10. shouldered points; 11. impact-fractured backed bladelet. 12. willow-leaf (?) fragment. **Middle Solutrean.** 13. splintered piece; **Lower Solutrean.** 14. ochred endscraper; 15-17. unifacial points. **Middle Gravettian.** 18. “burin” on truncation; 19. endscraper.

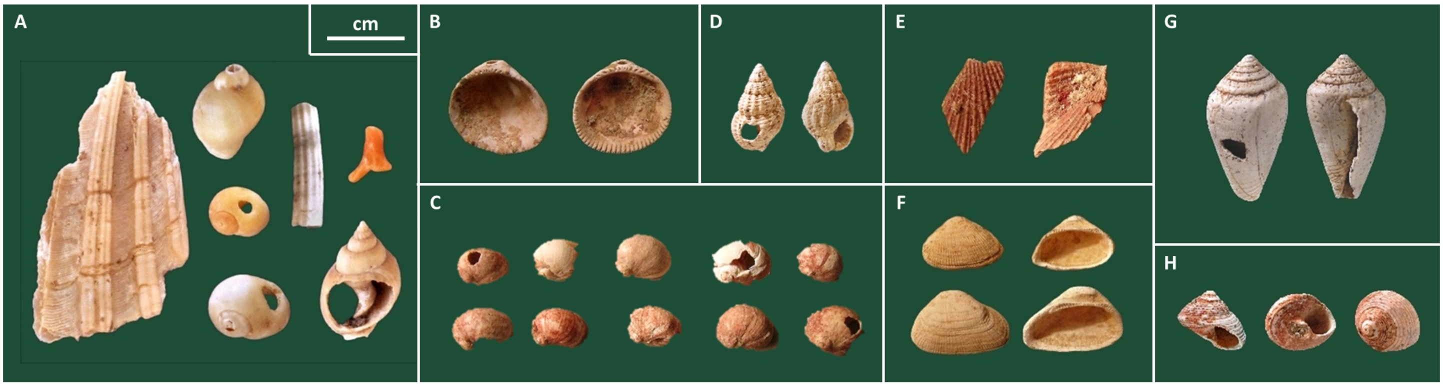

Fig. S4.34. La Boja. Items of ornamental and/or utilitarian function of marine or fluviatile origin. A. Solutrean. *Pecten jacobaeus*, *Nucella lapillus*, *Littorina obtusata*, *Dentalium* sp., *Tritia mutabilis*, *Coralium rubrum*. **B. Middle Gravettian.** umbo-perforated *Glycymeris insubrica.* **C-H. Evolved Aurignacian. C.** cluster of ochre-stained, perforated and unperforated *Theodoxus fluviatilis* found in grid unit U4, horizon OH19. **D.** *Tritia incrassata*. **E.** ochre-stained *Mimachlamys* sp. fragments. **F.** unperforated *Striarca lactea*. **G.** perforated *Conus ventricosus*. **H.** unperforated, ochre-stained *Gibbula* sp.

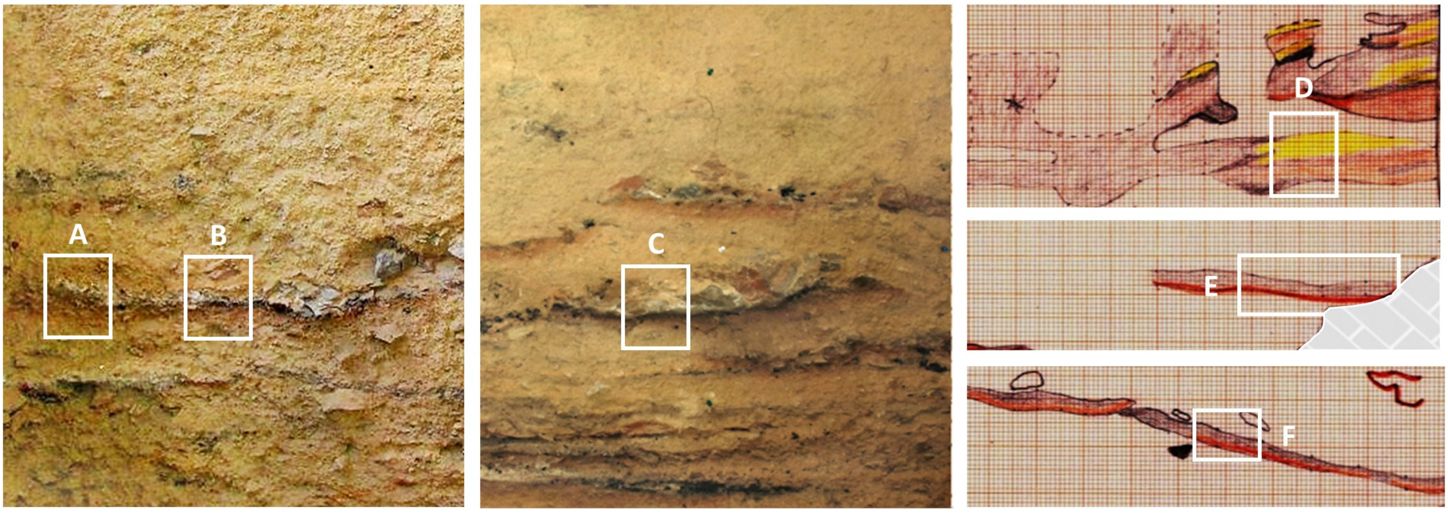

Lens	Column A [OH7]		Column B [OH7]		Column C [OH7]		Column D [OH12]		Column E [OH13]		Column F [mid-OH17]	
	Sample	N/g	Sample	N/g	Sample	N/g	Sample	N/g	Sample	N/g	Sample	N/g
above							254	10,069	230	114,452	202	36,783
white	039	1,980,926	044	2,050,897	103	731,656	251	1,421,136	228	1,382,820		
black	038	123,349	043	1,392,834	102	342,432	250	63,919	229	181,724		
red	037	57,734	042	12,113							201	9720
below									219	0	198	59,043

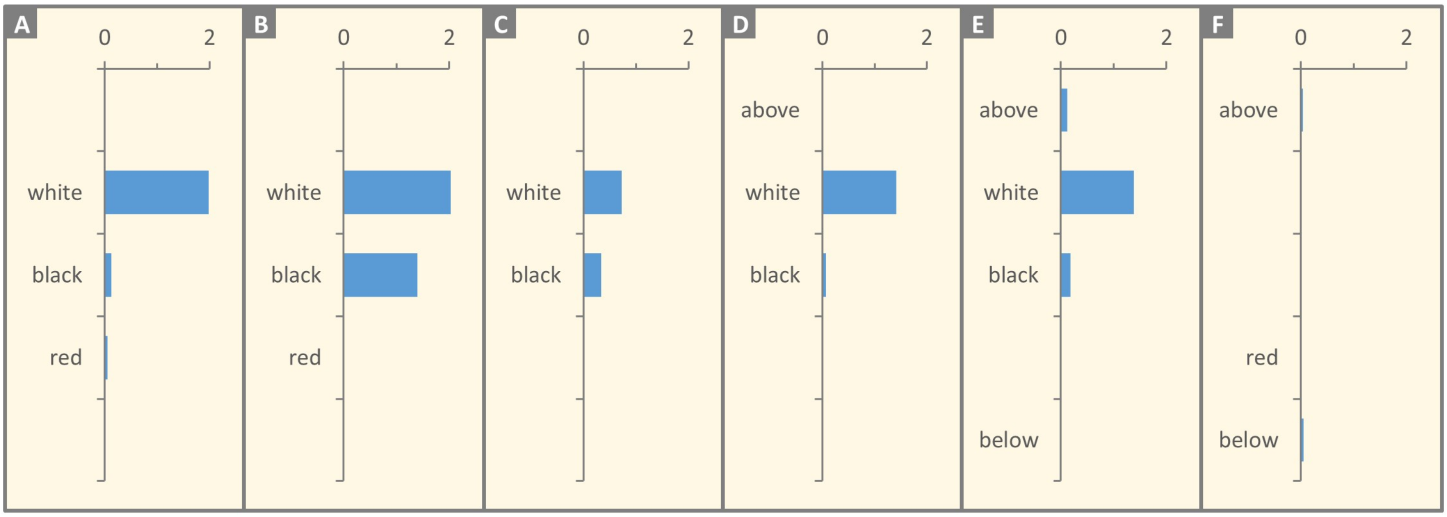

Fig. S4.35. La Boja. The phytolith component of fire-related lenses. Top. Position of selected sampling columns in the 2012 cross-sections (see Fig. S4.6). **Middle.** Examples of the vertical variation of concentrations (per gram of sediment) across the sequence of lenses making-up each occupation horizon; note that, upon analysis, some ash was found to be included in sample 230. **Bottom.** Plot of the concentrations (in millions per gram); note the strict correlation of the peaks with the white (ash) lenses, and the zero or near zero values found in the matrix (including the fire-reddened lenses at and below the ground surface upon which fire was lit).

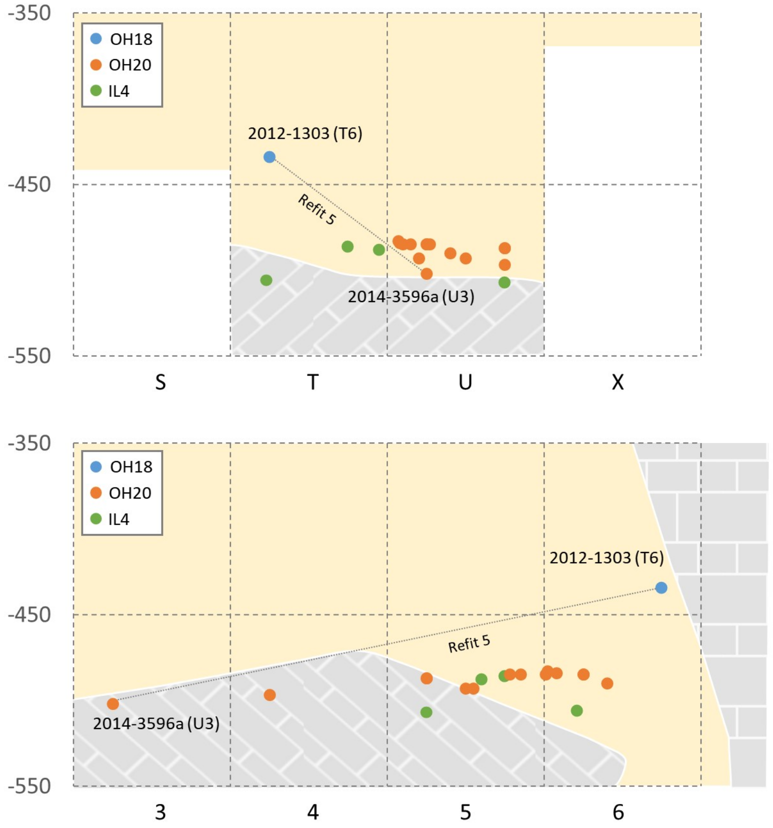

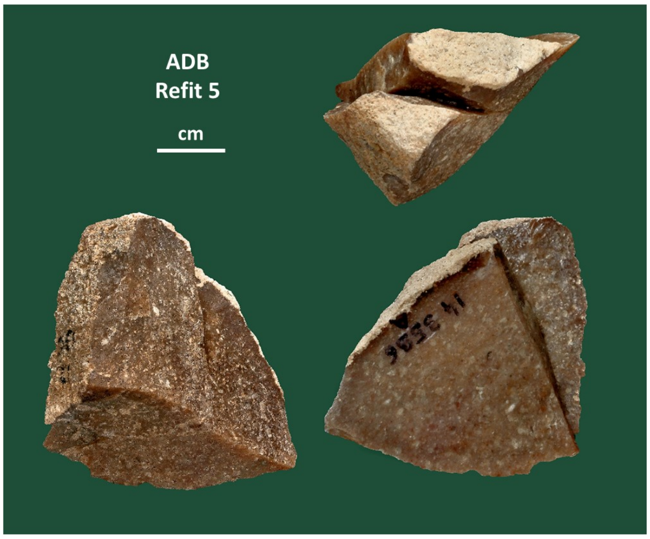

Fig. S4.36. La Boja. Above: Refit 5. **Left:** projection of piece-plotted items in the refits linking OH20 with IL4 (top, over the x-axis; bottom, over the y-axis); the outlines of back wall and collapsed boulder follow the U>T and 5>6 planes, and elevations are in cm below datum. The overlap between the OH20 and IL4 point clouds relates to the dip of the stratification to SE, induced by the geometry of the boulder's upside.

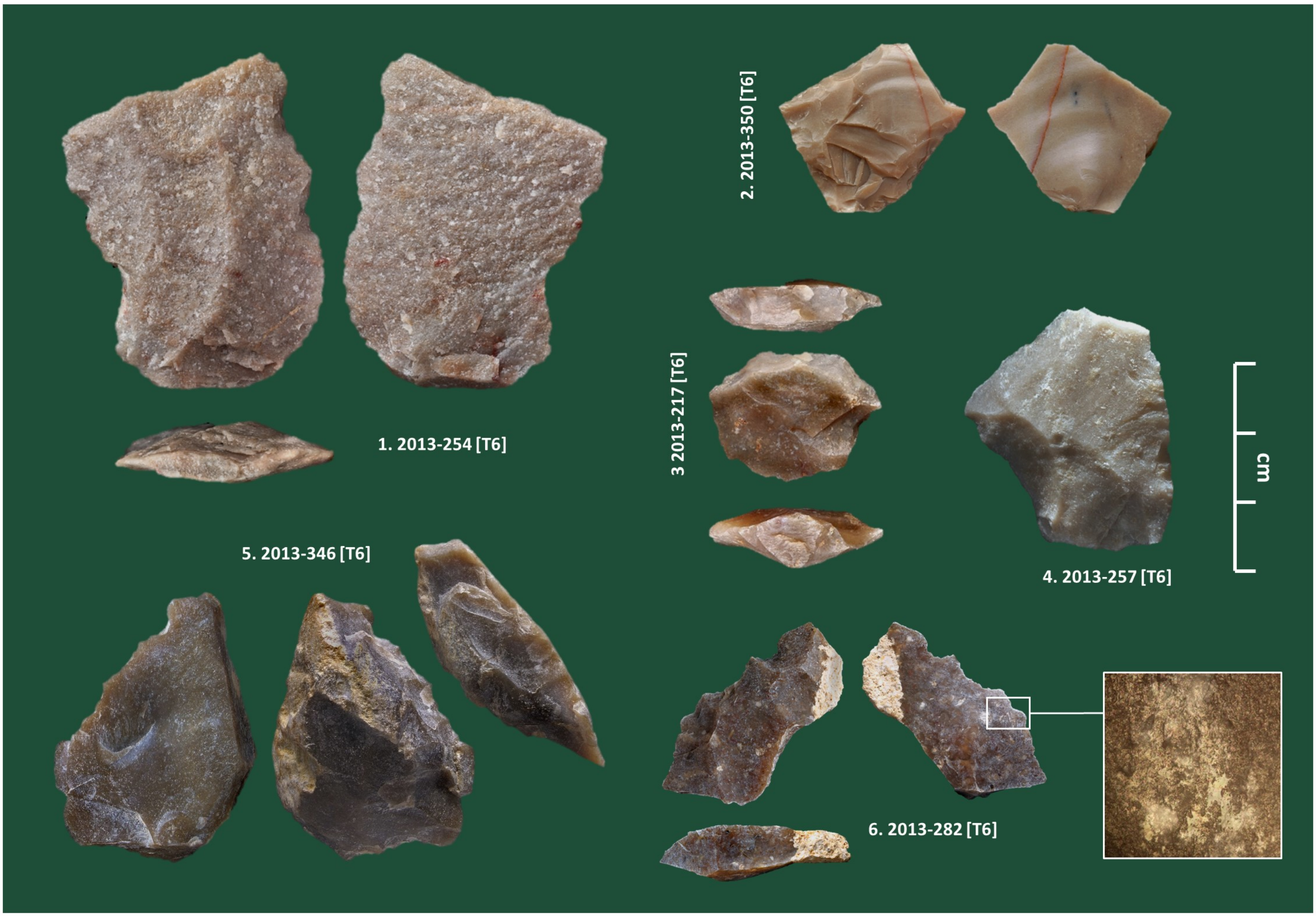

Fig. S4.37. La Boja Middle Paleolithic: formal stone tools from OH22 and OH23. 1. denticulate with distal break, quartzite; **2.** convergent sidescraper (with distal break); **3.** transversal sidescraper; **4.** sidescraper; **5.** denticulated sidescraper; **6.** denticulate. Item 2013-217 is from OH22, all the others are from OH23. The low-density, low-shine and diffuse polish seen on the 2013-282 denticulate are characteristic of use on wood.

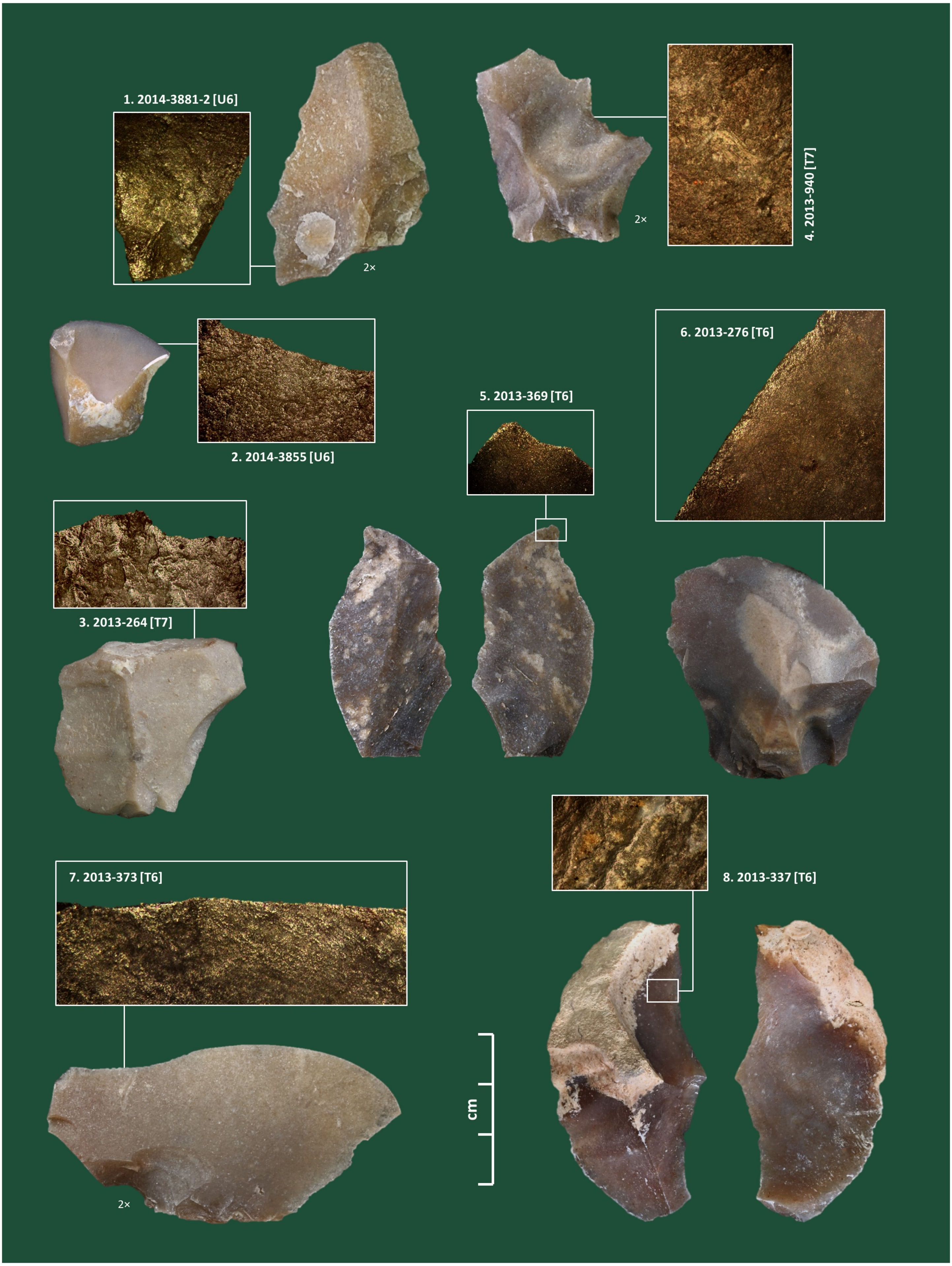

Fig. S4.38. La Boja Middle Paleolithic: use-wear evidence. 1-3, 6-7. unretouched blanks; **4.** notch; **5, 8.** edge-modified or edge-worn flakes. Use-wear was recorded at ×200 for nos. 2-7, and at ×400 for nos. 1 and 8. No. 8 is from OH22, all the others are from OH23. The "on bone" wear of no. 8 is little developed, suggesting that it was acquired through occasional contact in the course of a defleshing task. The attenuated, low-density, low-shining polish found on all the other pieces denotes use as wood-working tools.

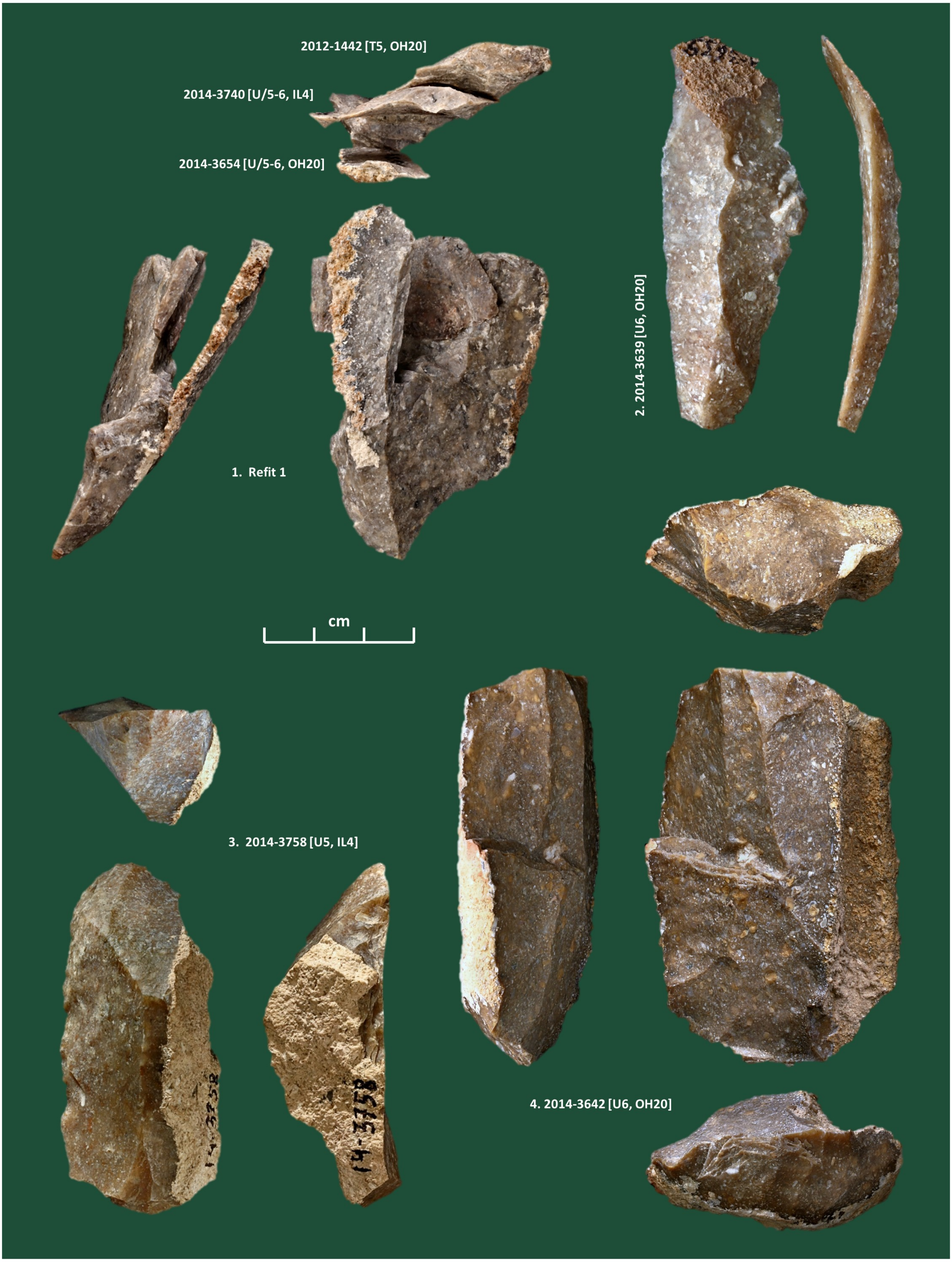

Fig. S4.39. La Boja. Blade technology of the Evolved Aurignacian in OH20/IL4. 1. production sequence aborted after a series of hinge fractures (nodule G); **2.** blank with broken-off butt; **3.** carinated “scraper” set-up on a thick, cortical blank; **4.** single platform prismatic core aborted after a series of hinge fractures; note the preparation of both platform and bottom.

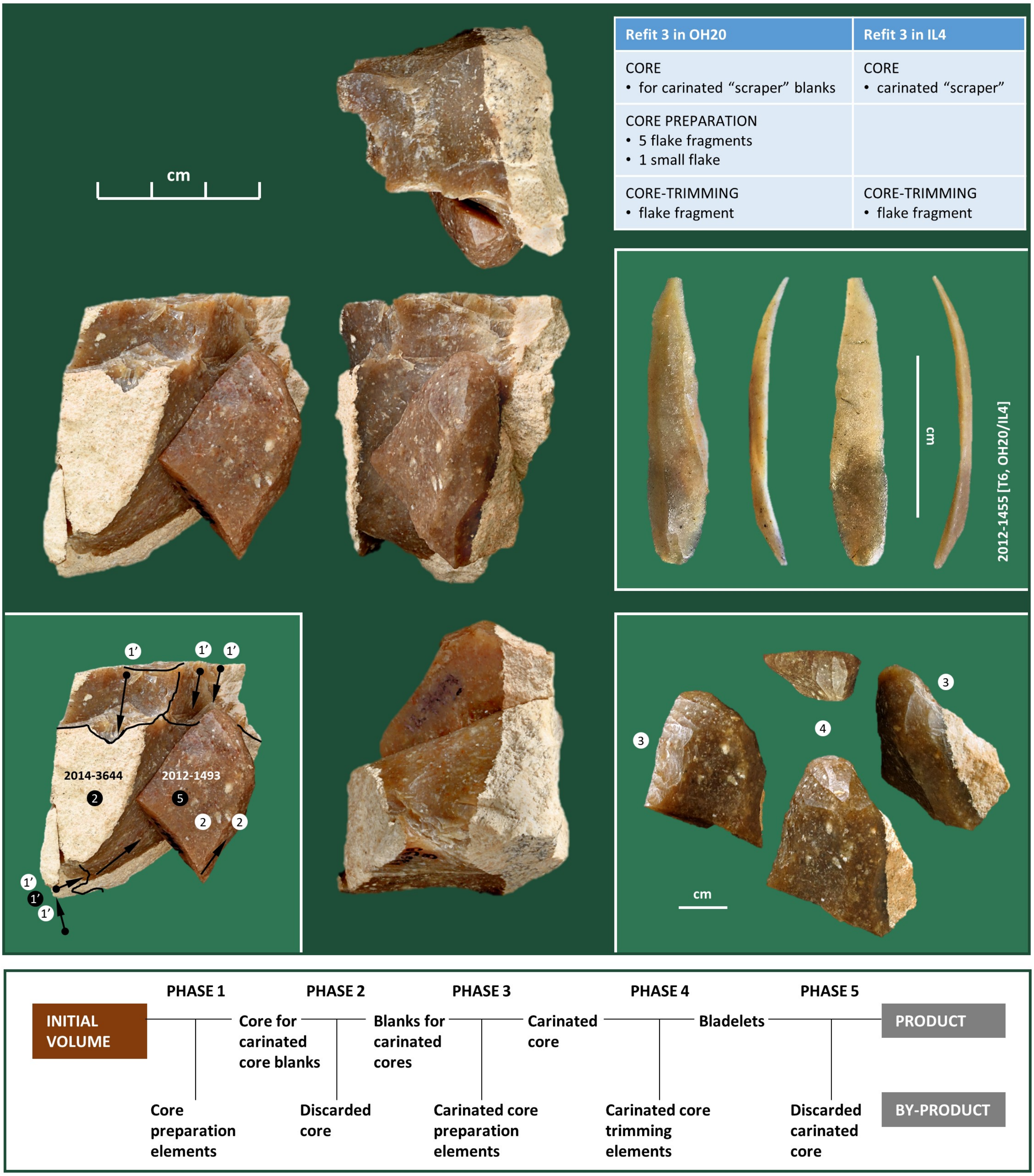

Refit 3 in OH20	Refit 3 in IL4
CORE<br>• for carinated "scraper" blanks	CORE<br>• carinated "scraper"
CORE PREPARATION<br>• 5 flake fragments<br>• 1 small flake	
CORE-TRIMMING<br>• flake fragment	CORE-TRIMMING<br>• flake fragment

Fig. S4.40. La Boja. The carinated "scraper" reduction scheme of the Evolved Aurignacian in OH20/IL4 as documented by Refit 3.3 of nodule J. The table inset provides the stratigraphic distribution of the different elements making up the three refitting units (3.1, 3.2 and 3.3) assembled with items from nodule J. The black circles denote phases represented by refitted elements, the white circles denote phases represented by the removal scars observed in refitted elements. Phase 1′ concerns the attempted (but failed, or aborted) recycling for another round of blank production of what remained of the initial volume (item 2014-3644; OH20) after the extraction of a series of thick blades to be set-up as carinated/nosed "scrapers," of which one (item 2012-1493; IL4) has been refitted back; of the material produced in this last round, only a core-trimming element could be refitted back (item 2014-3599b; OH20) on the initial volume. The bladelet 2012-1455 does not belong to nodule J but is illustrated here as a typical example of the reduction sequence's intended end-product. Note that the carinated "scraper" 2014-3758 (Fig. S4.39, no. 3) also belongs in nodule J; although it was not possible to include it in Refit 3 at this stage, the corresponding blank was in all likelihood extracted during phase 2 of the reduction of the same initial volume.

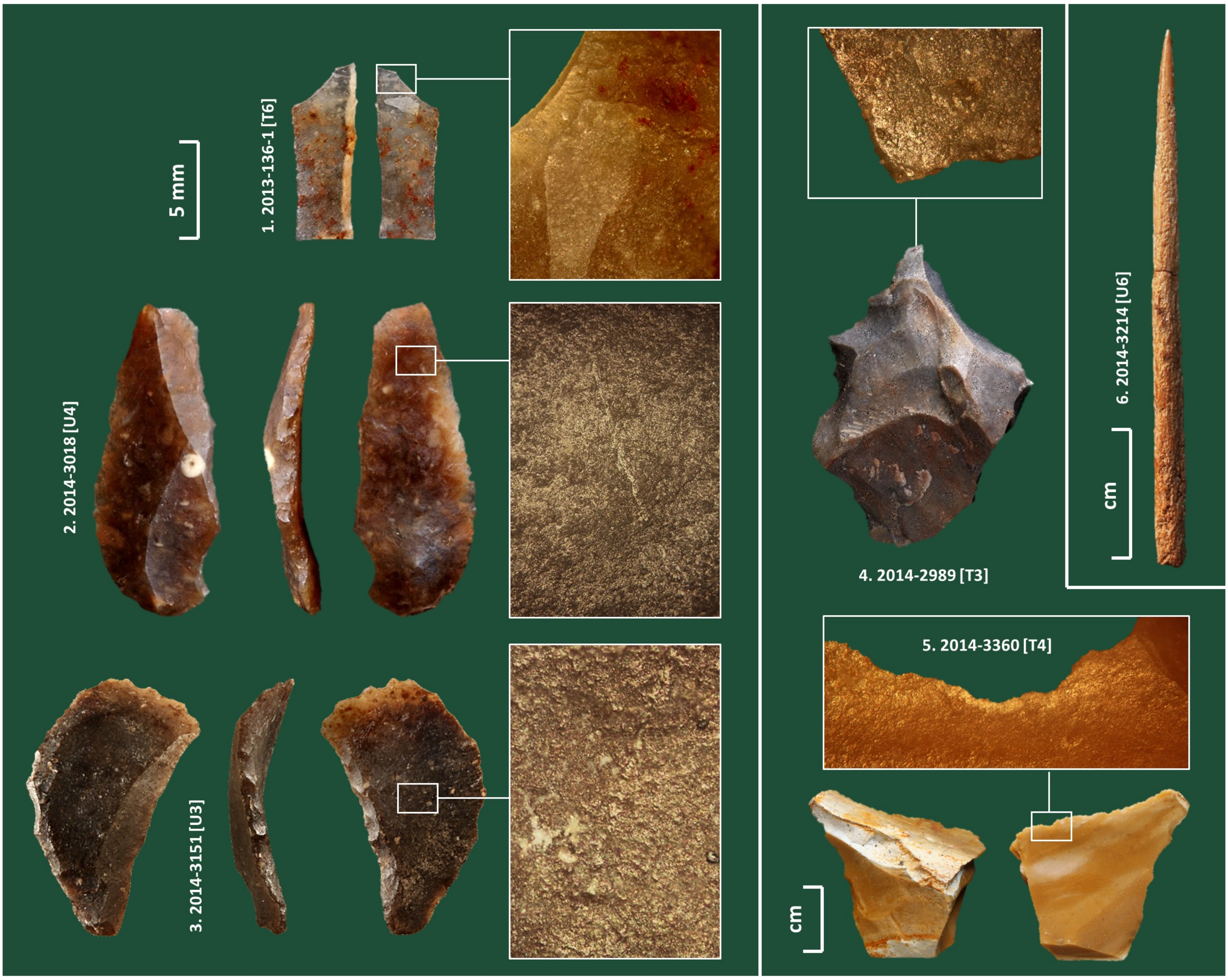

Fig. S4.41. La Boja. Aurignacian use-wear evidence. 1-3. microliths used as projectile elements (1. Areeiro [micro-Dufour] bladelet, IL4; 2. marginally backed bladelet, OH16; 3. Roc-de-Combe bladelet, OH17); **4.** perforator used on wood (OH16); **5.** edge-modified or edge-worn flake used on hard animal tissue (OH20). **6.** fine bone point with basal break (needle?) (OH17). Use-wear was recorded at ×100 for nos. 1 and 5, and at ×200 for nos. 2-4. In no. 5, the polish is dense but limited to the working edge and it is impossible to ascertain whether it was used on bone or antler; no. 6 attests the use, discard and, possibly on-site manufacture of bone tools. In no. 4, the break of the tip is characteristically wear-derived, and may have entailed loss of the area of the tool most intensively used; the polish apparent at the very edge of the break suggests use on wood. The striations apparent on the ventral surface of nos. 2-3 are diagnostic of impact, as is the mechanical break apparent in no. 1.

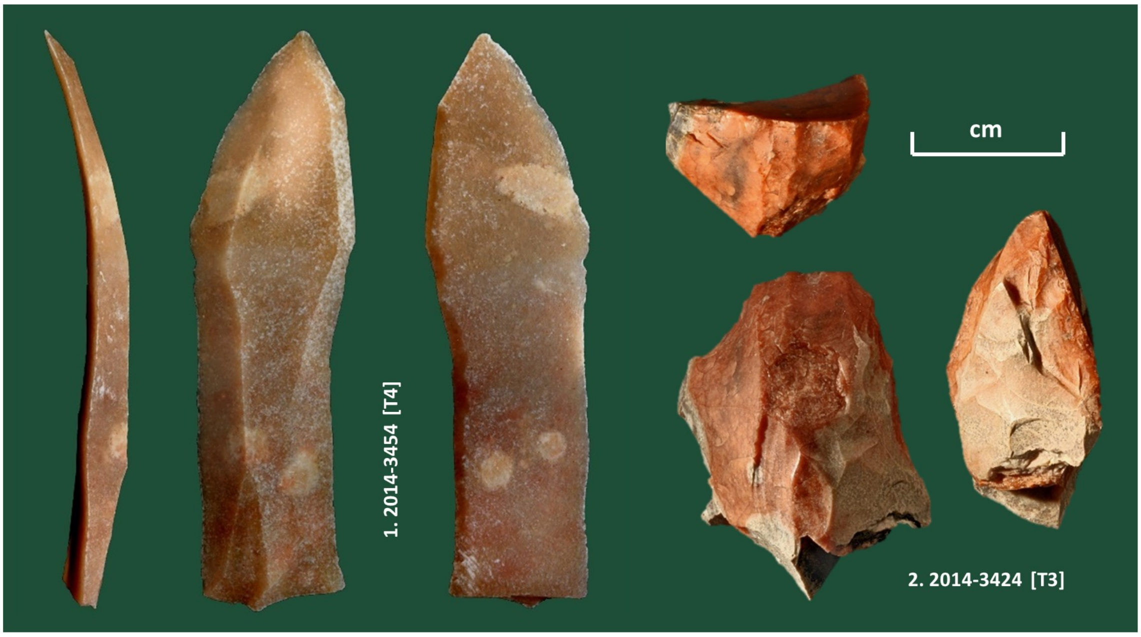

Fig. S4.42. La Boja. Size variation in the Evolved Aurignacian carinated "scraper"/ microlith technology. 1. large Dufour bladelet with proximal break from OH20 whose original length must have been >40 mm; **2.** exhausted, burnt nosed scraper retrieved inside the OH19 double hearth excavated in square T3, in which the scar left by the largest of the last bladelet extractions is 10.9 mm-long.

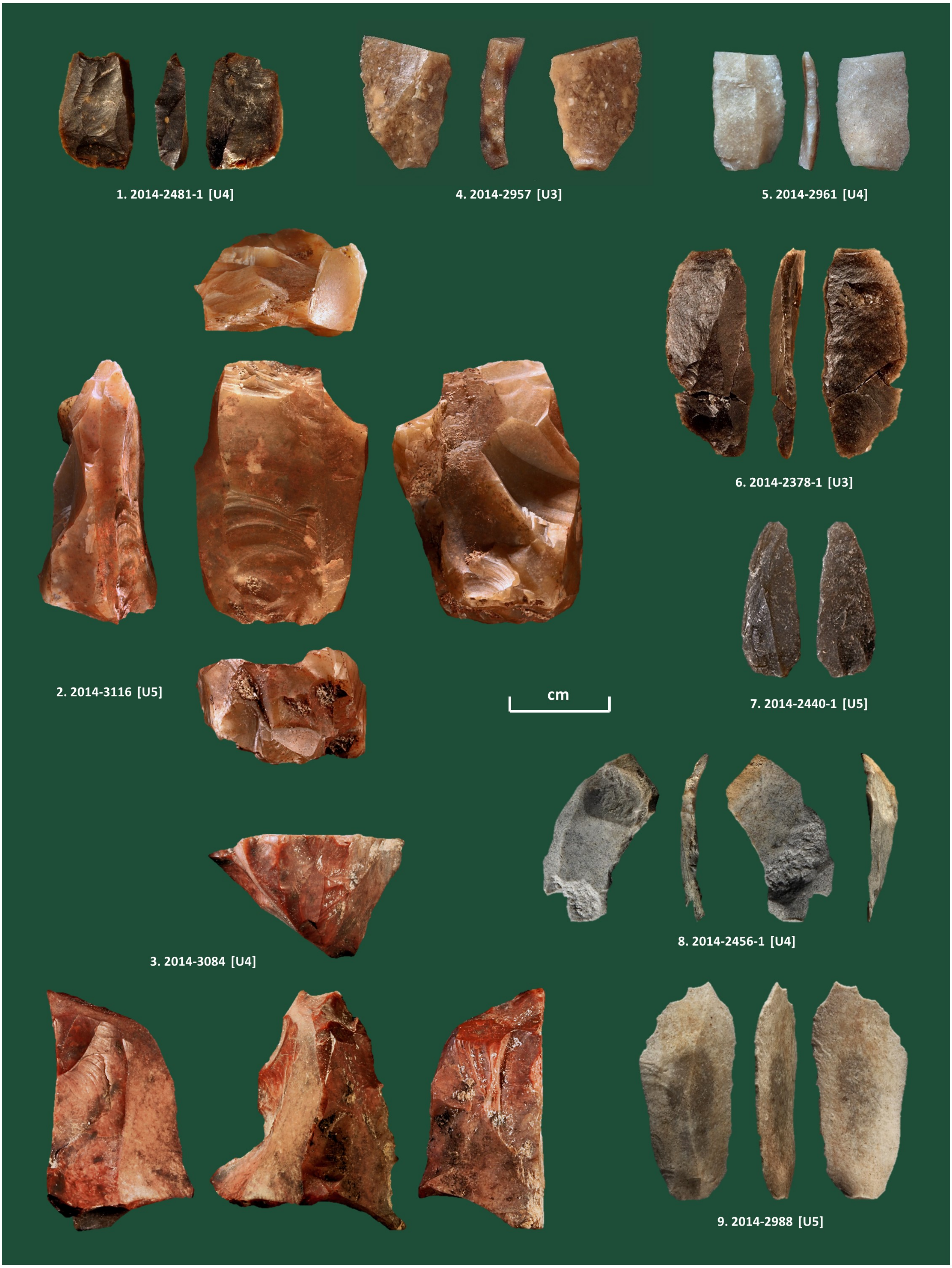

Fig. S4.43. La Boja. Stone tools from the Late Aurignacian (OH15-OH16) and the upper part of the Evolved Aurignacian sequence (OH17). 1-2. splintered pieces/bipolar cores. **3.** nosed "scraper;" **4-5.** proximal fragments of short-backed bladelets. **6-9.** Dufour bladelets. Nos. 4-6 are from OH15, nos. 1 and 7-9 are from OH16, nos. 2-3 are from OH17. Note that no. 8 is made on a blank extracted from a splintered piece/bipolar core.
